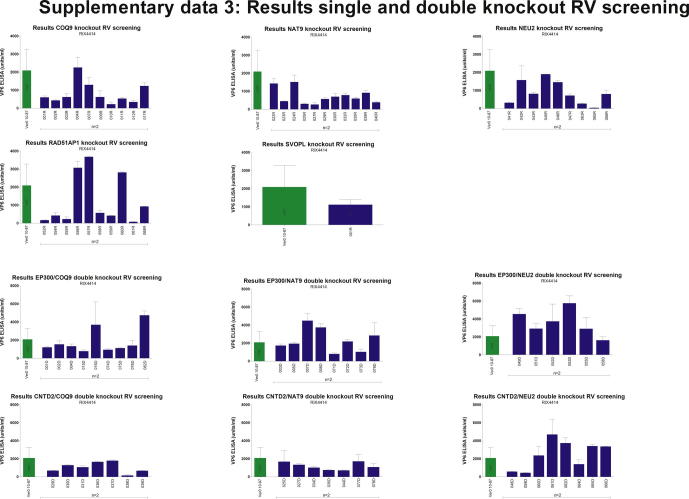# Enhancing viral vaccine production using engineered knockout vero cell lines – A second look

**DOI:** 10.1016/j.vaccine.2018.03.010

**Published:** 2018-04-12

**Authors:** F. Hoeksema, J. Karpilow, A. Luitjens, F. Lagerwerf, M. Havenga, M. Groothuizen, G. Gillissen, A.A.C. Lemckert, B. Jiang, R.A. Tripp, C. Yallop

**Affiliations:** a Batavia Biosciences, Leiden, The Netherlands; b Independent Researcher, Athens, GA, USA; c Division of Viral Diseases, National Center for Immunization and Respiratory Diseases, Centers for Disease Control and Prevention, Atlanta, GA, USA; d Department of Infectious Diseases, College of Veterinary Medicine, University of Georgia, Athens, GA, USA

**Keywords:** Vero cell, Cell substrate, Chlorocebus sabaeus, Manufacturing cell line, Gene editing, CRISPR, Gene knockout, Vaccine production, Poliovirus, Rotavirus, Microcarriers, Poliomyelitis, Costs and cost analysis, Virus cultivation, RNA viruses, Reoviridae, Picornaviridae

## Abstract

The global adoption of vaccines to combat disease is hampered by the high cost of vaccine manufacturing. The work described herein follows two previous publications (van der Sanden et al., 2016; Wu et al., 2017) that report a strategy to enhance poliovirus and rotavirus vaccine production through genetic modification of the Vero cell lines used in large-scale vaccine manufacturing. CRISPR/Cas9 gene editing tools were used to knockout Vero target genes previously shown to play a role in polio- and rotavirus production. Subsequently, small-scale models of current industry manufacturing systems were developed and adopted to assess the increases in polio- and rotavirus output by multiple stable knockout cell lines. Unlike previous studies, the Vero knockout cell lines failed to achieve desired target yield increases. These findings suggest that additional research will be required before implementing the genetically engineered Vero cell lines in the manufacturing process for polio- and rotavirus vaccines to be able to supply vaccines at reduced prices.

## 1 Introduction

Vaccines have a profound impact on global health, preventing illness, death, and improving the quality of life across the globe. However, the current costs of vaccine manufacturing and distribution often prevent the poorest segments of the world’s population from accessing these critical medicines. To address this problem, the identification and adoption of new technologies that lower costs and make vaccines affordable is an important objective.

Vaccine manufacturing processes are typically low yielding and production for global distribution regularly requires large and expensive manufacturing facilities that result in high vaccine prices and impede developing countries from initiating and/or expanding in-country manufacturing capabilities. To address this, new manufacturing technologies are being explored, including the development of optimized cell culture media, novel bioreactor designs that boost virus production by increasing cell densities, and innovative purification resins and membranes that result in higher recoveries and shorter process times (Barrett et al., [3]; Jacquemart et al., [4]; Tapia et al., [5]_,_ Rajendran et al., [6]). Another area for exploration is the engineering of manufacturing cell lines to improve virus propagation and vaccine yield. Viral vaccines are manufactured on a range of mammalian cell substrates including Vero, MRC-5, PER.C6 that are capable of supporting propagation and production of the vaccine virus strain. These cell substrates are a critical factor in the manufacturing process as they determine to a large extent overall vaccine yield. In general, approved manufacturing cell lines have remained unchanged for vaccine production. Several manufacturers have approached production and pricing challenges by attempting to modify the properties of the cells used to grow the virus. In some cases, adherent cells can be transitioned into suspension growth, thereby increasing virus production through increase in cell densities (Sanders et al., [7]). In other instances, clonal selection, the selection of a (sub)-clonal population within a parental cell population, has been used to improve the manufacturing properties of a cell substrate. Specifically, studies have shown that within a homogeneous population of cells, variants demonstrating improved vaccine production can be selected (Davies et al., [8]). In such cases, care must be taken in the screening process to ensure that the selected cell populations do not contain any traits that may negatively impact on the manufacturing process.

While clonal selection offers a proven opportunity to enhance virus yields, the underlying molecular basis for a cell’s improved properties remains unclear and the long-term stability of clones with enhanced traits remains a key challenge (Hou et al., [9]; Feng et al., [10]). To address these issues, researchers in both academic and industrial settings have begun to combine the vast wealth of knowledge generated during the genomics revolution with a new generation of synthetic biology tools (e.g., RNAi, and CRISPR/Cas9 gene editing). While such modified cell lines will need to undergo extensive testing to address questions regarding (1) genetic stability, and (2) the compatibility of modified cell line traits (e.g. doubling time, cell viability) with current vaccine manufacturing processes, this amalgam of technologies may enhance the production of both vaccines and biotherapeutic molecules.

The work presented here is a follow-up to a study performed by Van der Sanden et al. in 2016 [1]. In that report a genome-wide RNA interference (RNAi) screen identified multiple host gene knockdown events that enhanced the production of Sabin and wild type poliovirus (PV). These gene knockdown-mediated increases were dramatic, with 20- to 60-fold increases in viral titers observed in two unrelated cell lines (Vero and Hep-2C). Moreover, the overall effects (i) varied with virus serotype, (ii) were demonstrated to exhibit additive properties, and in some cases, (iii) facilitated the production of closely related viruses (e.g., EV-71). Importantly, the authors created stable Vero knockout cell lines of the top gene candidates using clustered regularly interspaced short palindromic repeat (CRISPR; Ran et al., [11]) technology and, using plaque assays, demonstrated that stable KO clones could dramatically improve PV vaccine strain production. In a separate study that examined gene targets that enhanced rotavirus (RV) production, the same group recently reported that 7- to 18-fold increases could be achieved through knockout of a single Vero cell host gene (Wu et al. [2]). With the reported dramatic yield increases for multiple viral vaccines, these discoveries could address the challenges currently facing governments and vaccine manufacturers.

In this manuscript, we investigated this approach further by evaluating the gene targets identified in the van der Sanden and Wu publications to determine whether stably engineered single and double knockout cell lines with greatly increased viral production of PV and RV could be created in the WHO 10-87 GMP Vero cell line currently employed in industry. Focusing attention on these vaccines is essential to address the economic and disease burden these two pathogens impose on the developing world. While significant progress has been made towards eradication of polio since the introduction of the Sabin live oral polio vaccine (OPV) in the 1950′s and the Salk inactivated polio vaccine (IPV) in the 1960′s, complete eradication requires that OPV, because it can result in rare cases of vaccine associated paralytic poliomyelitis (VAPP), be phased out and replaced with IPV. Such a change has a challenging price and supply impact. OPV is typically sold for less than $0.20 per dose (compared to IPV, which is sold in different price tiers based on the financial resources of the country; from under $1 per dose for the Global Alliance for Vaccines and Immunization (GAVI) countries up to $2.40 per dose for middle-income countries^(web 1, web 2)^. In addition, since IPV is an inactivated virus formulation, it requires nearly ten times greater amount of viral antigen to achieve equivalent levels of protection. Phasing out the OPV vaccine in favor of IPV would thus require a considerable increase in virus production capacity. For this reason, development of new cell lines, as well as new manufacturing technologies, enabling increased IPV production at reduced costs are paramount for achieving the global health goal of eradicating PV.

Similar issues surround RV infections and vaccines. RV infections have remained the most common cause of severe gastroenteritis among children under 5 years of age, leading to an estimated 215,000 deaths per year and millions of hospitalizations (Atherly et al., [12]; Tate et al. [13]). As almost all RV-related deaths occur in less developed countries where access to medical care is limited, the RV pathogen places an enormous burden on the healthcare resources of economically-strained geographies (Rheingans et al. [14]; Rheingans et al. [15]). Introduction of the Rotarix vaccine (GSK, Belgium) and RotaTeq vaccine (Merck, USA) have shown that immunization can significantly reduce RV-related hospitalizations in developed and developing countries (Leshem et al. [16]). However, the current RV vaccines are costly. Prices in developed countries such as the US and EU range from $50-$100 per dose and even in these countries, price has been cited as a barrier to the introduction of the vaccine with, for example, the UK, France and Germany delaying introduction of the vaccine into their childhood vaccination campaigns^(web 3)^.

Both Rotarix and RotaTeq are made available at reduced prices to low- and middle-income countries. For example GAVI prices in 2016 were $2–3.50 per dose^(web 4)^ while for PAHO in 2014 it was $5.50–$6.50 per dose. Despite this however, lower prices would be more conducive to the widespread adoption and use of these vaccines in poorer countries. New RV vaccines are coming on to the market, for example RotaVac (Bharat Biotech, India) and RotaSiil (Serum Institute of India) are in late stages of clinical development, while inactivated RV vaccines are also under development (Wang et al. [17]), which may improve vaccine pricing for the future through reduced manufacturing and infrastructure costs^(web 5)^. Regardless of the formulation, there is clearly a need for new technologies that increase the production of more affordable RV vaccines.

Engineered Vero cell lines capable of greatly increased production of IPV and RV vaccines would have an enormous impact on global health. In this study, we describe our efforts to generate single knock-out cell lines from the WHO Vero 10–87 cell line, capable of enhancing the production of PV and RV vaccines. In parallel, we summarize a novel study designed to combine the best knockout targets from the van der Sanden and Wu studies to create a double-knockout Vero GMP cell bank capable of enhancing production of both viral vaccines. For each program, a target titer amplification goal of 30-fold or greater was set, based on the previous publications indicating such a yield increase should be achievable (Van der Sanden et al. [1], Wu et al. [2]). In addition, a 30-fold increase in production can significantly alter the cost and capacity of current vaccine manufacturing platforms thereby making vaccines accessible and affordable to a greater portion of the global population. Small-scale models of the current industry manufacturing systems were developed and adopted to assess the increase in output by knockout cell lines. These procedures allowed for the testing of lead clones that could proceed rapidly into GMP Master Cell Bank manufacturing to dramatically increase vaccine supply at significantly reduced prices.

## 2 Results

### 2.1 Creation of single and double knockout clones for polio and rotavirus vaccine production

The PV and RV programs focused on isolating high performance single and double knockout Vero cell clones for genes identified in the van der Sanden and Wu publications. Below describes how the knockout generation and characterization was approached in the current study, as well compares and contrasts our techniques and results with those of previous groups (Van der Sanden et al. [1]; Wu et al. [2]).

For single gene knockouts dedicated to PV production, candidate genes included ZNF205, EP300, CNTD2, GCGR, and SEC61G (Van der Sanden et al. [1]). Single gene knockout candidates selected for the RV program included NEU2, NAT9, COQ9, SVOPL, and RAD51AP1 (Wu et al. [2]). For the double knockout experiments that combined individual targets for PV and RV production into a single clone, either EP300 or CNTD2 (PV enhancement) was combined with either NEU2, NAT9, or COQ9 (RV enhancement). As the first step in creating candidate gene knockouts in the WHO Vero 10-87 cell line, gRNA sequences were designed to the recently published Chlorocebus sabaeus orthologs (Osada et al. [18]). This approach differs from that adopted by van der Sanden et al. who designed targeting constructs to the human gene sequences. For our studies, four different gRNA sequences having at least three base pair mismatches to similar sequences were identified and synthesized for each target (Sigma Aldrich). In cases where unique gRNA sequences could not be designed based on these desired restrictions, gRNA sequences with two base pair mismatches were used. Each targeting sequence was subsequently assessed for activity by determining the percentage of insertions/deletions (in/dels) as result of the non-homologous-end-joining repair of CRISPR/Cas9-induced double strand breaks. The gRNAs with highest activity for each gene were selected for the knockout clone generation (see Table 1). For individual knockouts, synthetic gRNA and Cas9 protein were transfected into Vero 10-87 cells and clones were isolated by limiting dilution. For the generation of double knockouts, a mixture of Cas9 protein and individual gRNAs targeting each gene were sequentially introduced into cells, and prospective double knockouts were selected from the final pool. Following the isolation of individual clones, genomic DNA was extracted and next generation sequencing (NGS) was used to identify clones exhibiting NHEJ-induced frameshifts in each of the target gene copies. In cases where NGS could not provide genotypic information on all of the gene copies, such as the case with large deletions or multiple alleles having the same mutation, additional molecular analyses were performed. Supplementary data 1 shows molecular characterization of each of the total 116 knockout clones. Of those 116 clones, three clones were encountered in which molecular characterization was not fully conclusive (clones 012P, 007D and 044D); wild-type alleles were confirmed absent, but exact characterization (e.g. size determination of the deletion) was not confirmed for all in/dels. Of the 116 clones screened, one final cell bank showed a mixture of two clones of the same knockout (clone 008P).

**Table 1:** Host Genes Targeted for Single and Double Knockout Programs. Table lists the genes targeted by CRISPR/Cas9 mediated knockout for enhanced PV, RV and PV/RV (double knockout) production. Additionally, NCBI reference numbers, gene copy number in Vero, the exon targeted for knockout, the gRNA sequence employed for CRISPR/Cas9 knockout, and the NHEJ-activity (percentage of in/dels) identified in the CRISPR/Cas9 treated clones are provided. The number of confirmed knockout clones that were subsequently screened for the enhanced viral production phenotype are indicated as well.

Virus	Target genes	NCBI reference	Copy #	Exon targeted	gRNA sequence	In/Dels (%)	# of Clones screened for PV and RV production
PV	EP300	103,223,371	2	1	ccctctcggcgtccgccagcga	45	11
	CNTD2	103,234,699	2	1	cctctctttaggcgctgagtcc	57	11
	ZNF205	103,227,194	2	2	cccctaagtcacggctctaagg	10	–
	GCGR	103,243,730	3	4	ccgccaataccacggccaacat	65	8
	SEC61G	103,226,057	3	1	ccaagtcggcagtttgtaaagg	59	–
RV	NEU2	103,218,098	2	1	aggagagcgtgttccagtcggg	26	9
	NAT9	103,243,075	3	2	gtacttgtaccctacacctcgg	95	11
	COQ9	103,233,060	2	2	ccctggtgccacgtgccttcca	93	10
	SVOPL	103,226,981	4	4	ggctgacagatatggccgctgg	36	1
	RAD51AP1	103,218,421	2	3	gaaatccagaacaacaccaagg	55	10
PV + RV	EP300/NEU2	–	–	–	–	–	6
	EP300/NAT9	–	–	–	–	–	8
	EP300/COQ9	–	–	–	–	–	9
	CNTD2/NEU2	–	–	–	–	–	8
	CNTD2/NAT9	–	–	–	–	–	7
	CNTD2/COQ9	–	–	–	–	–	7

Multiple clones containing single gene knockouts were isolated for three of the five gene targets (EP300, CNTD2, and GCGR) for the PV program. An average population doubling time (pdt) of 27.6 ± 9.5 h was observed for each of the knockout clones, a value that was more variable, but comparable to the original parental cell line (24.2 ± 3.7 h). In contrast, despite analysis of greater than 1000 potential knockout clones, homologous knockout clones were not obtained for either ZNF205 or SEC61G. Additional attempts to create complete knockouts of ZNF205 by repeated transfection of the gRNA/Cas9 synthetics into heterozygote (ZNF205 +/−) clones also failed to generate a homologous clone for the ZNF205 gene.

For the rotavirus program, multiple single gene knockout clones were isolated for four out of the five target genes (NEU2, NAT9, COQ9, and RAD51AP1). For the SVOPL gene, only a single homologous knockout clone was isolated from >1000 clones assessed for each target gene copy by next generation sequencing. As was observed for single knockout clones for PV production, doubling times for the RV single gene knockout clones was similar to that observed in the parental line (24.1 ± 3.9 h). Finally, for the double knockout program, multiple clones containing knockouts of both target genes were isolated (Table 1). Doubling times (28.0 ± 9.1 h) were comparable yet more variable than those observed in the parental line.

### 2.2 Baseline performance of vero 10-87 and analysis of knockout clones for PV production

**Fig. 1:**
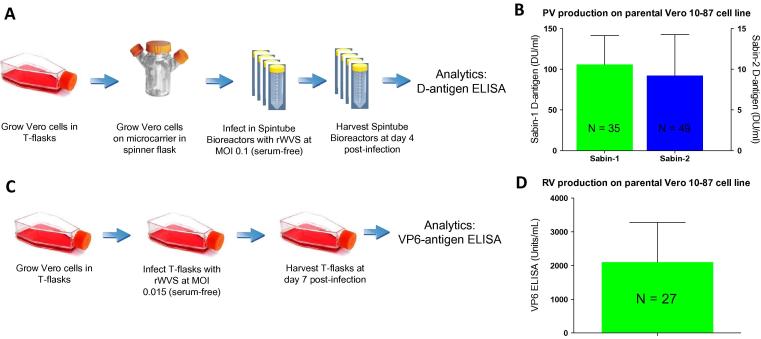
Virus production and baseline determination. A. Workflow for PV studies. WHO Vero 10-87 parental cells grown in T-flasks were transferred to spinner flasks and grown on microcarriers. Subsequently, microcarrier-associated cells were transferred to spintubes and infected with Sabin-1 or Sabin-2 poliovirus (MOI = 0.1). Four days-post infection, viral supernatants were collected and D-antigen levels were assessed by ELISA. (B) Baseline PV Studies with Vero 10-87. Bar graph shows the D-antigen production levels (in DU/ml) for Sabin 1 (green) and Sabin 2 (blue) over the course of 35 and 37 runs, respectively. Y-axis (left) represents D-antigen levels for Sabin-1; Y-axis (right) depicts D-antigen levels for Sabin-2. (C) Workflow for RV Studies. Vero 10-87 cells grown in T-flasks were infected with the RIX4414 vaccine strain of human RV at an MOI of 0.015. Flasks were incubated for 7 days prior to assessing VP6-antigen levels by ELISA. (D) Baseline RV Studies with Vero 10-87. Bar graph shows the VP6-antigen production levels (units/ml) over the course of 27 runs.

Prior to screening the knockout cell lines for PV antigen production, extensive baseline studies were performed on the Vero 10-87 parental cells using (i) Sabin-1 and Sabin-2 vaccine strains as the infectious agents, (ii) a downscaled manufacturing format for cell line screening (microcarrier/tubespin format; Bakker et al., [19]; de Jesus et al., [20]), and (iii) a D-antigen ELISA that included reference standards as the means of assessing viral yields (Fig. 1A). Results from these experiments were combined with additional control data generated from at least two Vero 10-87 cell control experiments, included in the evaluation of each clone screen. The compiled control data shows that production levels of 106 ± 36 DU/ml (n = 35) and 9.2 DU/ml ± 5.0 (n = 49) were achieved for Sabin-1 and Sabin-2 respectively in the WHO-10-87 control cell line (Fig. 1B). Internal ELISA controls showed an assay variability of 12% for the D-antigen ELISA, suggesting that biological factors contributed to the overall variability observed in the screen. Importantly, control study results demonstrated that the protocols developed for in-house screening of knockout clones gave Sabin-1 and Sabin-2 yields that were comparable to those achieved in industrial manufacturing platforms (Bakker et al. [19]).

**Fig. 2:**
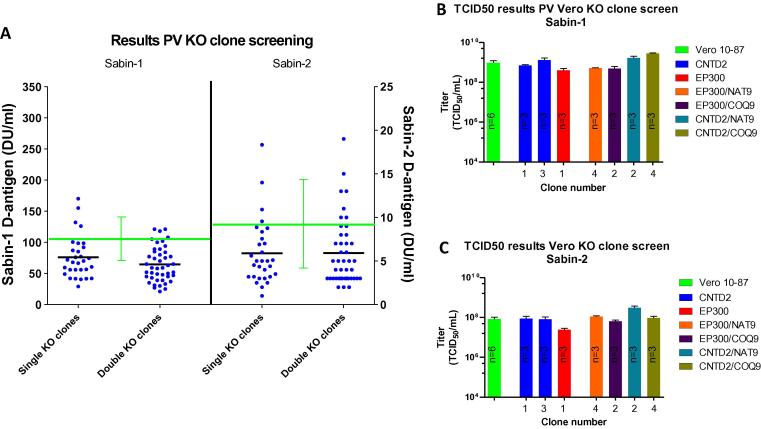
PV Production in single and double KO Clones. (A) Dot plot depicting the D-antigen ELISA results of single and double knockout clones for Sabin-1 and Sabin-2 poliovirus. Y-axis (left) shows the production levels (DU/ml) of single and double knockout clones with Sabin-1. Y-axis (right) shows the equivalent production levels of single and double knockout clones with Sabin-2. Each clone was screened in duplicate for PV production and each dot represents the average results of the two replicates. Green lines depict average production levels by the Vero 10-87 parental cell line. Black lines depict the average D-antigen levels generated by the single and double knockout clones. (B) Sabin-1 TCID_50_ results for the Vero 10-87 parental cell line (green) as well as a subset of single and double knockout clones. (C) Sabin-2 TCID_50_ results for the Vero 10-87 parental cell line (green) as well as a subset of single and double knockout clones.

The Vero 10-87 knockout clones for PV production were assessed using the procedures described above (Fig. 2A), with D-antigen ELISA used to evaluate clone versus parental production. Overall, we observed that the average results obtained in the knockout clones were lower than average results obtained in the parental cell line. The highest producing clones were within the range of productivity obtained by the Vero parental cells and showed (maximally) a ∼2-fold increase compared to the average observed for Vero 10-87. In addition to assessing volumetric yields, the specific productivity of each clone was also determined by taking into consideration the cell density at the time of infection. The results using this method of analysis also failed to show knockout clones yielding significantly higher PV D-antigen yields than the Vero 10-87 counterpart (data not shown). Supplementary data 2 shows the D-antigen results of each of the 75 single and double knockout clones screened in duplicate for Sabin-1 and Sabin-2 replication.

The original van der Sanden publication predominantly used plaque and TCID_50_ assays as a means to quantitate PV production. To determine whether our clones exhibited infectious titer improvements using these assays, a subset of the knockout clones developed here were assessed for their ability to enhance production of infectious PV particle. As shown in Fig. 2B and C, the TCID_50_ results for our knockout clones were comparable to the Vero parental cells, thereby confirming the results of the D-antigen ELISA. In addition, three knockout clones (EP300 (1) and CNTD2 (2) targeted) and the parental Vero WHO 10-87 cells were tested at the laboratory of the original study (Van der Sanden et al. [1]) using a plaque assay. As was observed previously, those knockout clones showed a maximum increase of 3-fold compared to the Vero WHO 10-87 parental (data not shown).

A follow-up capability analysis on the ELISA data determined that for all single and double knockout clones tested for PV antigen, the probability of attaining the desired production increases of 30-fold was 0%. Similarly, the probability of achieving a 10-fold increase was also found to be highly unlikely (0.03% for Sabin-1, and 0% for Sabin-2). As none of the single or double knockout clones showed a sufficient increase in PV production to warrant transition into manufacturing platforms, follow-up bioreactor performance experiments could not be justified. To investigate whether assay differences could be the source of the discrepancies between our findings and those of van der Sanden et al. [1], we tested PV supernatants (Sabin-1) generated from the original Vero cell line as well as two of the original knockout cell lines (ZNF205 and CNTD2 single gene knockouts) described in the 2016 publication. In this follow-up, the supernatants of all three cell lines were tested in the source laboratory and the knockout clones were reported to show a maximum increase in PV production of 5- to 9-fold over the parental Vero cells; substantially lower than originally reported. When the knockout supernatants were analyzed in our laboratory, they were observed to exhibit minor increases of ∼2-fold over the titers observed in the parental cell line (data not shown).

### 2.3 Analysis of WHO Vero 10-87 sub-clones for PV production

**Fig. 3:**
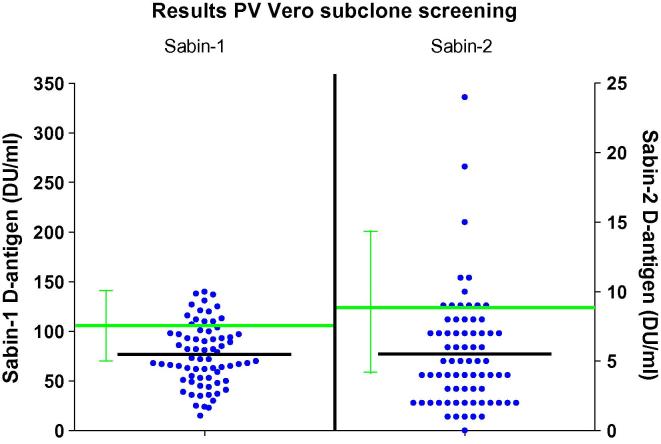
PV Production in Vero sub-clones. Dot plot depicting the D-antigen ELISA results of 72 sub-clones for Sabin-1 and Sabin-2 poliovirus. Y-axis (left) shows the production levels (DU/ml) of the sub-clones with Sabin-1. Y-axis (right) shows the equivalent production levels of the sub-clones with Sabin-2. Green lines depict average production levels by the Vero 10-87 parental cell line. Black lines depict the average D-antigen levels generated by the sub-clones.

The 2 to 3-fold increase in Sabin-1 and Sabin-2 PV replication was observed in only a fraction of the knockout clones developed in this study. The absence of consistency across the collection precipitated the question of whether the limited improvements were related to CRISPR-mediated gene knockout or, alternatively, clonal variability. To try to identify the impact of possible clonal variation in the absence of CRISPR-mediated gene modifications, 72 sub-clones of the Vero 10-87 parental cell line were created and screened for PV production using the same microcarrier-based platform as employed in the Vero knockout studies. As was the case in the knockout cell line study, the average titer across the collection of 72 sub-clones was lower than that of the parental line (Fig. 3). Moreover, as was the case in the knockout cell line studies, the highest producing sub-clones (i) were within the range of productivity of the parental cells, (ii) showed (maximally) a 2- to 3-fold increase, and (iii) were observed at a frequency that was comparable with the occurrence of high-producing clones generated in the knockout cell line program. In the knockout data-set, 10.8% of clones showed higher Sabin-1 production than parental average, whereas this was the case for 19.4% of the sub-clones. Similarly, for Sabin-2 production, 14.9% of knockout clones were higher than average Vero parental; in comparison, 16.7% of sub-clones showed higher yields than the Vero parental cells. Applying a threshold criterion of >1.5-fold increase of clone versus average Vero parental shows that 6.8% of knockout clones meet this criterion (for either Sabin-1 or Sabin-2), while 4.2% of sub-clones fulfill the criterion. While these findings do not exclude the possibility that targeted gene knockout contributed to PV production in a subset of the KO clones, the similarities between the two data sets is consistent with the conclusion that in this study, knockout of the target genes did not increase PV production and that individual KO clones exhibiting limited (2- to 3-fold) increases in PV titers were likely the result of clonal variability.

### 2.4 Baseline performance of Vero 10-87 and analysis of knockout clones for RV production

**Fig. 4:**
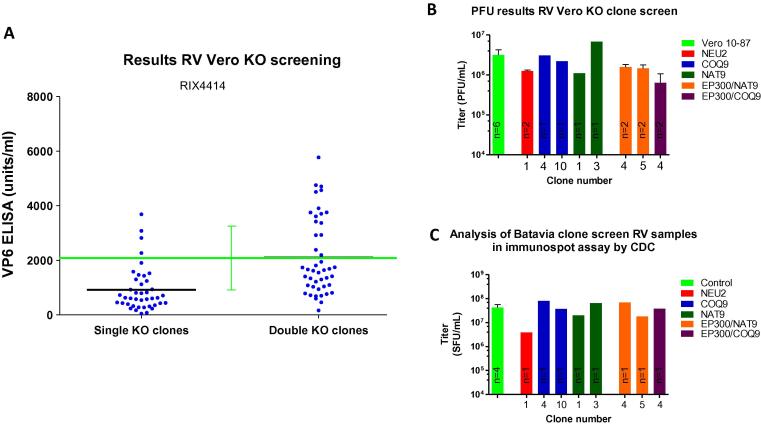
RV Production in single and double knockout Clones. (A) Dot plot depicting the VP6-antigen ELISA results of single and double knockout clones for RIX4414 RV. Y-axis indicates the production levels (units/ml) of single and double knockout clones. Each clone was screened in duplicate for PV production and each dot represents the average results of the two replicates. Green line depicts average VP6 production levels by the Vero 10-87 parental cell line. Black lines depict the average VP6-antigen levels of single and double knockout clones. (B) Infectious RV titers based on plaque forming unit (PFU) assay. Results are presented for the Vero 10-87 parental cell line (green) as well as a collection of single and double knockout clones. **C.** RV titers based on immunospot assay. Results are presented for the Vero 10-87 parental cell line (green) and a subset of single and double knockout clones.

RV studies were initiated by assessing the ability of the Vero 10-87 parental cells to foster RV replication in the downscale platform, developed in T-flasks as representative downscaled format of cell factories, which are typically applied for RV vaccine production (Fig. 1C)^(web 6)^. To achieve this, parental cells grown in T-flasks were infected with a live-attenuated RIX4414 G1P [8] human RV vaccine strain (Rotarix®) and assessed using a VP6-antigen ELISA (Fig. 1D). As was the case in our PV studies, data from RV baseline studies were combined with at least two control experiments on parental Vero performed during the knockout clone assessment. Fig. 1D shows that in the downscaled manufacturing platform developed for this study, RIX4414 VP6-antigen yields in WHO Vero 10-87 cells were 2098 ± 1178 U/ml (at n = 27). As reported above, parental cell doubling time under these conditions was found to be 24.2 ± 3.7 h. As these values are comparable to those obtained in industry, assessment of the knockout clone performance followed. The ability of single and double knockout clones to increase RV production are shown in Fig. 4. As was observed in the PV studies, the highest VP-6 antigen producing RV clones showed productivities that were within the range of the parental Vero cells, with a maximum increase of 3-fold (clone versus parental average) being observed for a double knockout clone (CNTD2/COQ9). Supplementary data 3 shows the VP6-antigen results of each of the 86 single and double knockout clones screened in duplicate for RIX4414 replication. To confirm the VP6-antigen ELISA studies, a plaque assay that measured infectious particles was performed. These results confirmed that knockouts in the prescribed genes did not result in increased RV plaque production over what was observed in the Vero 10-87 cell line (Fig. 4B). These conclusions were further supported by an immunospot assay (Fig. 4C) executed in a separate laboratory. Based on these results, a capability analysis was performed on the ELISA data to calculate the probability of obtaining increased RV production using the knockout clones generated by the described methods. It was determined that for all single and double knockout clones for RV production the probability of achieving the targeted 30-fold production increase was 0%. Similarly, the probability of increasing RV production by 10-fold was 0% for the single knockout clones and 0.06% for the highest producing double knockout clone. Based on these findings, scale-up experiments with the knockout cell lines were not performed.

## 3 Discussion

The work presented here follows on two promising studies (published in 2016 and 2017) that described how knockouts of individual Vero cell host genes enhanced the production of viruses included in current PV and RV vaccines. Our study was designed to rapidly transition this new technology into vaccine manufacturing with the goal of dramatically increasing cell line productivity, increasing global supply and decreasing Cost of Goods (CoGs) for these two important vaccines. Lead clones, identified by a screen performed in a downscaled manufacturing format would then, after performance confirmation in bioreactor (for PV) and cell factory (for RV) formats, be expanded under GMP to create Master Cell Banks that would be available to vaccine manufacturers and researchers alike.

Our approach towards attaining these goals began with generation of host gene knockouts in the WHO Vero 10-87 cell line. Using CRISPR designs that targeted the predicted Vero cell orthologs of the selected gene targets, we successfully isolated and verified by next generation sequencing (NGS) the gene knockout status of multiple target genes. Subsequently, downscale manufacturing platforms for both PV and RV were created and baseline performance studies of the parental Vero 10-87 cell line for PV and RV vaccine strains were achieved through rigorous testing (35–37 control runs for Sabin-1 and -2; 27 control runs for RIX4414). Average PV D-antigen yields obtained on the Vero parental cells using the downscaled manufacturing format were similar to those described by Bakker et al. [19] for Sabin-1 propagation (120 ± 9.42 DU/ml) and approximately 2.5-fold lower for Sabin-2 (24.5 ± 1.29 DU/ml) (Bakker et al. [19]).

Surprisingly, after assessing the performance of multiple confirmed knockout clones using D-antigen (PV) and VP6-antigen (RV) ELISA to measure viral output, we concluded that none of the clones showed production increases of 20- to 80-fold as previously reported in van der Sanden et al. [1] or 7- to 18-fold as reported by Wu et al. [2]. Indeed, no substantial increases in virus yield were demonstrated for any of the knockout clones generated. Follow up studies using assays that measure live virus production (plaque assay and TCID_50_) and immunoblotting confirmed these observations. We therefore conclude that the procedures and cell lines described herein were not able to recapitulate and build on the results published in previous reports (Van der Sanden et al. [1]; Wu et al. [2]) and that a lead clone capable of significantly increased PV and RV production capabilities and decreased vaccine costs was not identified. Indeed, a similar range of PV production was obtained with sub-clones of the Vero 10-87 cells (without gene knockouts) suggests the absence of any impact of the knockout gene targets in this study.

The principal issue now is to better understand the differences between the results described in this study and those published in the previous studies for the gene knockout results (Van der Sanden et al. [1]; Wu et al. [2]). Certainly, there are any number of factors that could explain the dissimilarities. First, while the cell lines used in this study and previous reports were both Vero-derived, the history of these two cultures are unquestionably different. The Vero 10-87 cell line used in our study was established by the WHO for vaccine manufacturing purposes. In contrast, the Vero culture employed by van der Sanden and Wu has been maintained in an academic setting for research purposes. It is well documented that cell lines derived from the same source can deviate dramatically over time, including cell lines frequently used in industry like Vero and CHO. Dahodwala et al. [21] showed that five CHO cell cultures derived from the same parental line exhibited significantly different growth responses in the presence of native and recombinant insulin and Davies and colleagues [8] have shown that CHOK1SV clones derived from a single in-house population can exhibit heritable variations in multiple traits during subculture. Given these findings, it is conceivable that the knockout of target genes in the van der Sanden/Wu Vero isolates might yield different results from similar gene modifications in the 10-87 cell line used in this study. Similarly, the origin and generation of the virus seed stocks applied for the screening of the cell lines in the different studies may also have a role in the differences in results obtained. RNA viruses have an intrinsic capacity for genetic modification potentially leading to evolving levels of fitness. Moreover, the results described in this paper on two PV vaccine strains and one RV vaccine strain do not rule out that better replication enhancement can be obtained when testing different viral strains, as exemplified by differences in viral transcript enhancement observed in a knockout Vero cell line tested for three different RV strains by Wu et al.

Another explanation for the disparate results may lie in how the knockout clones were generated in each study. In our work, the Chlorocebus sabaeus genomic sequence was imported for gRNA design and individual synthetic gRNA sequences were transfected into Vero 10-87 cells together with CRISPR/Cas9 protein to reduce the risks of off-target effects resulting from, e.g., extended exposure to multiple gene targeting reagents. At the time of the Van der Sanden et al. publication, the Chlorocebus sabaeus genome was unpublished and for that reason, guide strand RNAs were designed to the human target gene sequences. In addition, in contrast to the approach adopted here, van der Sanden et al. [1] simultaneously introduced four separate gRNA sequences targeting an individual gene into Vero cell cultures along with a plasmid that encoded CRISPR/Cas9 function. This combination of variables i.e., simultaneous introduction of multiple gRNA sequences and long-term CRISPR/Cas9 expression, might have resulted in enhanced off-target effects that could augment in cell productivity in unknown ways.

Other aspects of the workflow should also be considered as possible contributors to the differences between our results and those of van der Sanden and Wu. In our workflow, prospective clones isolated through limiting dilution were sequenced and only confirmed knockouts were then tested with PV and RV strains for increases in titer. In this fashion, the knockout status was confirmed first and the question of whether the knockouts exhibit increased virus production followed. In van der Sanden et al. [1] and Wu et al. [2], clones isolated by FACS were first tested for the ability to generate high viral titers, and subsequently, clones that generated high titers were then sequenced to examine the status of the target gene locus. This latter approach raises two closely linked questions: (1) do all high-performing PV and RV clones isolated by Van der Sanden and Wu show disruptions of the target gene and, by extension, (2) if the protocol was performed in the absence of gene targeting reagents (i.e. by simple sub-cloning of the parental cell line), would high performing clones with similar yield increases as obtained in the knockout clones still have been isolated, as described in the present study. Certainly, understanding the nuances of these protocol differences might provide insights into the importance of the protocols in creating high-performance knockout cell lines and the contributions that the gene targets make to RV and PV replication. Another factor that might explain some of the discrepancies between the reports is related to the status of the target gene in knockout clones. The original work reported by van der Sanden and Wu demonstrated that knockdown of target genes using RNAi technology resulted in increased production of PV and RV respectively. It is possible that phenotypes induced by transcriptional suppression (i.e., RNAi-mediated knockdown) and genetic deletion (i.e., CRISPR/Cas9 knockout) are not the same. In such a situation, one may imagine that RNAi induced gene knock-down may result in a phenotype with increased virus production while a gene knockout does not. Our results with ZNF205 may underscore these differences. As reported here, we were unable to generate verifiable homozygous knockouts of ZNF205 for enhanced PV production, even after a second transfection of ZNF205 gRNA-CRISPR/Cas9 targeting reagents was performed on ZNF205 +/− heterozygotes. This result is in contrast to the van der Sanden et al. article that reports a ZNF205 knockout clone enhancing PV production by greater than 50-fold. In the work reported here, the status of both target gene alleles was confirmed with next-generation sequencing. In van der Sanden et al., the knockout status of one allele, obtained by Sanger sequencing, is reported. The Sanger technique verifies that at least one allele was knocked out, but does not eliminate the possibility of the presence of an intact wild-type allele, i.e., a heterozygous state. Certainly, if ZNF205 knockouts are lethal and the van der Sanden ZNF205 clone was a heterozygote, it might simultaneously explain our inability to isolate homozygous knockouts for this gene and support the van der Sanden report that ZNF205 targeting by CRISPR/Cas9 mimics results found with RNAi knockdown technology.

The type of assay applied in the different studies may also account for the differences in results obtained. For example, Wu et al. reported enhanced RV replication in a Vero knockout cell line using qPCR, whereas in this study we used the VP6-antigen ELISA to assess RV replication. Moreover, the impact that biological and assay variability plays on clone analysis should be considered as a potential source of the differences reported in the three studies as well. In the previous study, the researchers predominantly used a plaque assay to quantitate their findings on PV replication and reported the knockout cell line enhancements as fold-increases over the Vero controls performed in each experiment (Van der Sanden et al. [1]). As exemplified by the work presented here, biological variability can greatly impact the assessment of clone performance and a large number of control assays measuring viral titers must be performed to accurately quantitate the baseline performance of the Vero 10-87 cell line to which all subsequent knockout cell line studies are compared. In the absence of this approach, variability within the clone screen set-up may mistakenly lead to the conclusion of having identified a high producer cell clone. In support of this premise we observed that when the Van der Sanden clones were re-tested with the objective to compare potential assay differences with the current study, the initial reported increases of 20- to >80-fold were not reproduced; a maximum 9-fold increase was observed in two repeats of the experiments. Cell line instability, e.g. as result of potential heterozygous knockout state as described above, may also have contributed to this inability to reproduce the initial reported significant PV yield increases.

Based on these findings, several steps are proposed to further investigate the technology and its potential application for generating high yielding, industrially suitable cell lines that can have a major impact on the cost of vaccines. The first priority would be to investigate whether the reported differences can be explained by dissimilar cellular states induced by gene knockdown and knockout. As described above, the van der Sanden and Wu studies both demonstrated that knockdown of target genes using RNAi led to increases in yields for both PV and RV. Such an approach employed synthetic siRNA pools that typically provide silencing of the target gene transcript, leaving (potentially) <30% of the gene’s function remaining. In contrast, our knockout cell lines, generated using CRISPR/Cas9 technology and validated by next-generation sequencing, eliminate gene function but failed to exhibit increases in viral titers. One explanation for the observed discrepancies is that knockdown and knockout of target genes leads to two different cellular states; one which supports increased viral replication (knockdown) and the second which provides no additional enhancements (knockout). To tackle this hypothesis, a side-by-side comparison that further investigates the knockout state of our cell lines and those from the van der Sanden/Wu collection using next generation sequencing and quantitative Western blot analysis, might provide clues that explain the current discrepancies. In the circumstance that gene KD would result in increased virus propagation while gene knockout would not, then further characterization of gene function would be required in order to generate a suitably engineered cell line that could be used for vaccine manufacturing.

A second program that might provide insights into reported disparities delves into the differences in the two original parental cell lines. It is conceivable that the Vero cells used in the van der Sanden and Wu studies possess a somewhat different genetic background than the Vero 10-87 cell line; one that can be augmented by KD or knockout to further enhance PV and RV production whereas the same genetic knockout or KD in the WHO Vero 10-87 cell line would not result in this response. Repeating the original RNAi knock-down experiments in both the van der Sanden/Wu Vero cell line as well as the Vero 10-87 cell line using industry-accepted metrics (e.g., D-antigen and VP6-antigen ELISAs) and extensive baseline studies could rapidly determine whether the two Vero subcultures respond equivalently to target gene knockdown. Certainly, if it was determined that gene knockout or knockdown would have very different effects based on the genotype of the target cell line, this would represent a major step in our understanding of Vero-based cell substrates for bioproduction.

Finally, several experiments could be performed to determine whether (i) the workflow, or (ii) assay variability contributed to the observed discrepancies. Repeating the original van der Sanden/Wu FACS-based workflow with additional controls that explore whether enhanced production clones can be isolated in the absence of CRISPR/Cas9 gene-targeting reagents would distinguish between contributions made by targeted gene knockout and pre-existing variability present in the cell population. Likewise, a side-by- side performance analysis of the knockout cell lines from all three reports that includes extensive parental baseline studies using multiple assays and controlled viral seed stocks could determine the contributions that biological and assay variability make to these three programs. Certainly, if one or more of the proposed studies reveal that stable, 30-fold (or greater) increases in virus production can be achieved, new cell substrates could be rapidly developed to address global vaccine challenges.

## 4 Materials and methods

### 4.1 Virus and Vero stocks

The two Sabin poliovirus strains used in this study (PV1, 01/528; PV2, 01/530) were obtained from the National Institutes of Biological Standards and Controls (NIBSC). For all RV experiments, a live-attenuated RIX4414 G1P [8] human RV vaccine strain (Rotarix®, a trademark of the GlaxoSmithKline) was used (O’Ryan, 2007 [22]). The RIX4414 strain was obtained via the ECACC (deposited under ECACC accession number 99081301). The WHO Vero 10-87 cell line (Knezevic et al. [23]) was obtained from the ECACC (deposited under ECACC accession number 88020401) at passage number (pn) 134. A research cell bank at pn 138 was generated and used as starting material for clone generation. The MA104 cells (Whitaker and Hayward [24]) used in the RV plaque assay were obtained from the ECCACC as well (accession number 85102918) at pn 11. A research cell bank at pn 18 was generated and used for the plaque assay.

Vials containing 3 × 10_6_ cells of the parental cell line, WHO Vero 10-87 and the Vero 10-87 cell lines containing knockouts in individual genes were thawed into T175 flasks containing of 30 mL DMEM (Gibco) with 10% FBS (Gibco). Media was refreshed on day 1 and cultures were incubated at 37 °C, 10% CO_2_ for an additional 3–4 days. Subsequently, the flasks were washed with D-PBS (Gibco), trypsinized with TrypLE Select (Gibco) and passaged at a cell density of 7500–12,000 cells/cm^2^. Vero sub-clones were generated by seeding Vero 10-87 parental cells at 0.1 cells/well in 96-well plates (Greiner). Cells of clonal origin were subsequently scaled up, banked (at pn 143) and used for a PV production screen.

### 4.2 Vero knockout clone generation

Vero knockout clone generation was outsourced to the Sigma-Aldrich Cell Design Studio. Briefly, the WHO Vero 10-87 cell line (pn 139) was transferred to Sigma Aldrich and cultured in DMEM + 10% FBS (Sigma Aldrich). Subsequently, genomic DNA was purified and copy number analysis was performed by digital droplet PCR (ddPCR) using primers and probes designed to each gene target. The reference genes and corresponding NCBI gene ID used for this analysis include: GUSB (Gene ID: 103246571), TERT (Gene ID: 103214928), RPP30 (Gene ID: 103216241), and EGFR (Gene ID: 103226055). CRISPR/Cas9 was used to induce frameshifts within target genes and thereby create knockout clones for each of the target genes. For the majority of the targets, gRNAs were designed using Sigma-Aldrich’s proprietary design algorithm that prioritizes targeting sequences at early positions in the reading frame. Designs had at least 3 bp mismatch to any other location in the genome, but in 4 cases (gene targets SEC61G, COQ9, SVOPL and RAD61AP1), 3 bp mismatches could not be achieved and designs with 2 bp mismatches were selected. For ZNF205 and EP300, the gRNA designs used in the original Van der Sanden study (2016) were employed. For CNTD2, a modification of the original Van der Sanden gRNA that (i) overlapped with the original design, (ii) eliminated base pair mismatches with the Vero target gene, and (iii) exhibited increased cutting efficiency, was employed. An overview of gRNAs used in this study is presented in Table 1. Transfections with GFP-containing plasmids were performed as transfection efficiency controls.

To generate pools of prospective single knockout cell lines, synthetic guide RNA (Sigma-Aldrich) and wild-type Cas9 recombinant protein (PNA Bio) were introduced into Vero cells by nucleofection (Lonza) and individual clones were isolated via limiting dilution (0.6 cell/well concentration in 96-well half-area plates). To produce double knockout pools, cells were nucleofected with Cas9 protein and gRNA targeting the first gene and, following a ∼48 h incubation, Cas9 protein and gRNA targeting the second gene was transfected. For both single and double knockout clone isolation, plates were scanned using a CloneSelect Imager (Molecular Devices) and wells containing more than one colony were eliminated. Individual colonies were expanded to approximately 50% confluency before being transferred and expanded to 80% confluency in conventional 96-well plates. Cultures were then replica plated whereupon half of the material was used to further expand each clone while the remaining cells were processed to prepare genomic DNA for next generation sequencing (NGS). Three filters were applied to NGS data before clones were considered for PV and RV replication studies. These included: (i) 1000x sequence coverage, (ii) observation of the insertion/deletion with allelic fractions complying to copy number determined for gene target, and (iii) confirmation of the original sequence analysis in the final cell bank. Based on these criteria, heterozygous clones or clones having in-frame mutations were eliminated. In cases where NGS could not provide genotypic information on all of the gene copies, such as the case with large deletions or multiple alleles having the same mutation, additional molecular analyses were performed. These include: (i) direct sequencing of PCR amplicon to sequence and visualize chromatogram for presence of heterozygous small nucleotide polymorphisms (SNPs), (ii) agarose gel electrophoresis to separate and detect different sizes of PCR amplicons, (iii) fragment length analysis to measure exact length of the PCR amplicon up to 1 kb; (iv) topo cloning and sequencing to allow sequencing up to 2 kb; and (v) long range PCR amplification (∼3kb) to allow detection of large deletions by visualization on agarose gel and subsequently isolation and sequencing of fragments. Cells of clonal origin were subsequently scaled up, banked (typically at pn 152) and used for the PV and RV production screen.

### 4.3 Poliovirus infection

To screen cell lines for PV production, cells were scaled up to four T175 flasks. Flasks containing either the Vero 10-87 parental cell line, sub-clone or a Vero 10-87 cell line containing one or more knockouts of target genes were grown to >80% confluency. The cultures were then washed (D-PBS) and trypsinized with TrypLE Select, followed by 5 min of centrifugation at 300*g*. Spinner flasks (Corning, 3152) were then seeded with a mixture of 0.5*g* Cytodex 3 (GE Healthcare) and 30 k cells/cm_2_ cells in DMEM + 10%FBS. The culture conditions for the spinner cultures was: 50 mL working volume, 37 °C, 10% CO_2_ and 50 RPM stirring. On day 1 following inoculation, 50 mL of DMEM, 10% FBS was added and the stirring speed was increased to 60 RPM. After 4 days of culture, a 10 mL sample was taken from each spinner flask and a cell count was performed to calculate the amount and volume of virus required for infection at a multiplicity of infection (MOI) of 0.1. For the remainder of the culture, the Cytodex 3 carriers were separated (gravity) and washed (2×) by replacing 70 ml of the culture with DMEM without FBS. The culture was then aliquoted into 4 Spintubes (TPP, 20 mL per tube) whereupon two (2) tubes were infected with Sabin 1 poliovirus and two tubes were infected with Sabin 2 PV at an MOI of 0.1. Parallel experiments were done with the Vero 10-87 parental cell line as a control. The Spintubes were then incubated at 34 °C, 10% CO_2_ at a shaking speed of 170 RPM. After 4 days of infection, the Spintubes were placed at ≤ −65 °C for at least 16 h, thawed and aliquoted for further analysis.

### 4.4 Rotavirus infection

The parental and knockout cell lines were grown in three, (3) T75 flasks. One flask was sacrificed for a cell count to calculate the amount and volume of virus required for an MOI of 0.015. The remaining 2 flasks were washed twice with D-PBS, after which 12.5 mL DMEM with 10 µg/mL Trypsin (Gibco) was added to each flask. The flasks were then infected with RIX4414 strain of RV (MOI = 0.015) and incubated at 37 °C with 10% CO_2_. After 7 days, the flasks were placed at ≤−65 °C for at least 16 h, thawed and aliquoted for further analysis.

### 4.5 D-antigen ELISA

The amount of poliovirus D-antigen units (DU) in cultures was quantified using a sandwich ELISA, and compared to an international DU reference standard (Poliomyelitis vaccine (inactivated) BRP, EDQM). Briefly, a strain-specific anti-Polio virus rabbit polyclonal antibody (in-house) diluted in bi-carbonate buffer was coated overnight at 2–8 °C on 96-wells plates. The next day, plates were washed 4 times (0.05% Tween-20 in PBS) to remove unbound antibodies and blocked for 60–90 min with blocking buffer (PBS with 1% BSA and 2% rabbit serum). Freeze-thaw supernatants from control and experimental studies (singular; 8 serial 2-fold dilutions) and reference standard (EDQM, in duplicate; 8 serial 2-fold dilutions) were added and incubated at 36 °C for 1.5 h. Blocking buffer only was used as a negative control. Plates were then washed as described before and a biotinylated, strain-specific anti-Polio virus rabbit polyclonal antibody (in-house) was added for 1 h at 36 °C to create the immunological sandwich complex. Additional washes were performed to remove unbound materials before horseradish peroxidase (HRP)-labeled extravidin peroxidase conjugate (Sigma-Aldrich) was bound to the immobilized biotin through a 30 min incubation for at 36 °C. Final washes preceded the addition of the HRP substrate, TMB, and reactions were halted with 1 N H_2_SO_4_. Absorption was measured at 450 nm and background (630 nm) values were subtracted. Sample concentration was expressed in D-Antigen units/ml (DU/ml), derived from the international standard with concentrations of 320 and 67 DU/ml for Sabin-1 and Sabin-2, respectively (EDQM reference, P2160000, batch 2).

### 4.5 VP6-antigen ELISA

RV was quantified by measuring Viral Protein 6 (VP6) levels by sandwich ELISA according to manufacturer’s instructions (RIDASCREEN Rotavirus ELISA kit, R-Biopharm AG). Assay results were compared to a reference standard (Rotarix, NDC58160-851-10, GSK). Briefly, a 7 × 3-fold serial dilution in diluent was first performed on samples in a 96-well plate. In parallel, an 8 × 1.5-fold serial dilution was performed in 2 independent replicates on the reference standards. Each RV reference and test sample was then pipetted into the microwell plate pre-coated with a primary anti-RV antibody, immediately followed by the addition of a biotinylated monoclonal anti-rotavirus antibody (kit content) and incubated for 1 h at room temperature. Plates were then washed (wash buffer (kit content), 3 times) and incubated with a Streptavidin poly-HRP peroxidase conjugate for 30 min at room temperature. Following the incubation, plates underwent a final wash and the immobilized biotin was detected by adding the peroxidase substrate, TMB. The reaction was stopped using 1 N H_2_SO_4_. Absorption and background were measured at 450 nm and 630 nm, respectively, using a ELx808, BioTek plate reader. The resulting delta OD is proportional to the concentration of RV found in the samples. Sample potency was expressed in ELISA units/mL (EU/ml), derived from the Rotarix standard.

### 4.6 TCID_50_ Assay

Infectious PV virus titers were calculated using a 50% Tissue Culture Infective Dose (TCID_50_) assay. Prior to infection Vero cells were plated at a density of 5.1 × 10^3^ cells per well (96-wells plate, MEM medium supplemented with 10% non-heat inactivated FBS (Gibco) plus 8 mM L-Glutamine (Gibco) and incubated at 32.5 °C, 5% CO_2_. Virus supernatant samples were pre-diluted (depending on the expected titer) followed by a 3-fold serial dilution in 8 replicates in a 96-well plate containing 150 µL of MEM medium supplemented with 5% non-heat inactivated FBS and 4 mM L-Glutamine. Vero cell cultures were then infected with 100 µL of each serial dilution within 4 h of seeding and incubated at 32.5 °C, 5% CO_2_. Cytopathic effect (CPE) was scored seven (7) days post-infection and TCID_50_ titer was determined using the Spearman-Karber method (Ramakrishnan [25]).

### 4.7 PFU assay

Infectious RV titers were determined using a plaque forming units (PFU) assay. MA104 cells were plated at a density of 5x10^5^ cells per well in a 6-wells plate and incubated for 3 days. RV (RIX4414) samples were prepared by duplicate, 5-fold serial dilutions after activation with 45 μg/mL trypsin. MA104 cells were infected with 1 mL diluted RV for 2 h and subsequently overlayed with 2% agarose containing 11 μg/mL trypsin. Plaques formed within an incubation period of 4 days. Cells were (i) fixed with a 5% glutaraldehyde solution, and (ii) stained with 0.5% crystal violet, in order to count plaques and quantitate the RV titers.

### 4.8 Immunospot assay

Monolayer MA104 cells in 96-well microtiter plates (Costar) were infected with serially 10-fold dilutions of RV in triplicate and incubated at 37 °C in (5% CO_2_, 18 h). After incubation, the cells were fixed with formaldehyde for 20 min at 4 °C and then air dried. The plates were treated with diluted rabbit hyperimmune serum to RV (CD10) in PBS containing 1% skim milk for 1 h. HRP-conjugated goat anti-rabbit immunoglobulin G (IgG) was added for 1 h, and the plates were then incubated with TrueBlue solution supplemented with H_2_O_2_ for 10 min. The titers of individual RV samples (SFU/ml) were determined by counting the number of blue dots in the well using ImmunoCapture 6.5 and CTL BioSpot Analyzer 5.0.

### 4.9 Capability analysis

In the capability analysis, the results for each clone are compared to the results of the parental Vero. For this analysis, all data was log10-transformed. The mean and standard deviations were calculated per clone and for the parental Vero separately. The difference between the log10-transformed data of each clone and the parental clone is calculated. The upper specification limit (USL) was set at 3000% production compared to the parental Vero, meaning that the clone would be considered a substantial producer per the pre-set acceptance criteria of 30 times increase of a clone compared to the parental Vero. Therefore, the capability analysis was performed using log10(3000/100) to find the percentage of the distribution that is above the USL. When this percentage is above 10%, there is a chance that the corresponding clone is a “high producer” and should be further investigated. The mean and standard error of the mean of this log10 difference is used to calculate the mean production and 95% confidence interval of that production for each specific clone compared to the parental Vero.

## Figures and Tables

**Supplementary data 2:**
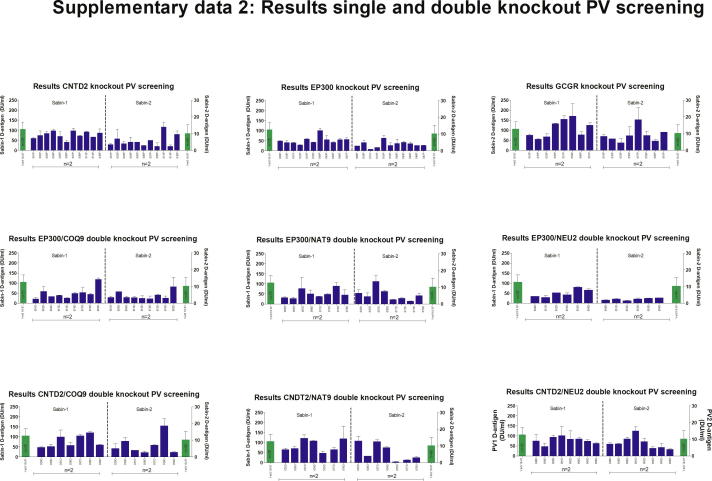


**Supplementary data 3:**