# Supplementary material for: Enhancing viral vaccine production using engineered knockout vero cell lines – A second look
Source: Vaccine. 2018 Apr 12;36(16):2093–103. doi: 10.1016/j.vaccine.2018.03.010 (PMC5890396; doi:10.1016/j.vaccine.2018.03.010)
Supplement: Supplementary data 1 [file mmc1.pdf]

PV single knockout clones

CNTD2 single knockout clones

Clone ID	In/dels
001P	-110/-62
002P	-32/+1
003P	-28/-17
004P	-25/+1
005P	-13/+1
006P	-11/-1
007P	-1/+1
008P	-1/-14/-62
013P	+1/+1
014P	-11/-1160
039P	-13/-5

EP300 single knockout clones

Clone ID	In/dels
009P	-47/-16
010P	-14/-11
011P	-7/+25
012P	-31
015P	-25/+1
016P	-17/-68
040P	-2/+2
043P	-16/+1
044P	-41/-19
046P	-2/-1
047P	-1/-2

GCGR single knockout clones

Clone ID	In/dels
018P	+1/+35/+32
035P	-1/-10/-11
053P	-5/-1/-488
056P	-16/-2/-341
057P	+1/+4/-467
059P	+4/-11/+1
060P	-1/-1/-17
061P	-11/-16/-1

Clone 001P (-110/-62)

NGS 1, fragment analysis, NGS 2 (cell bank)

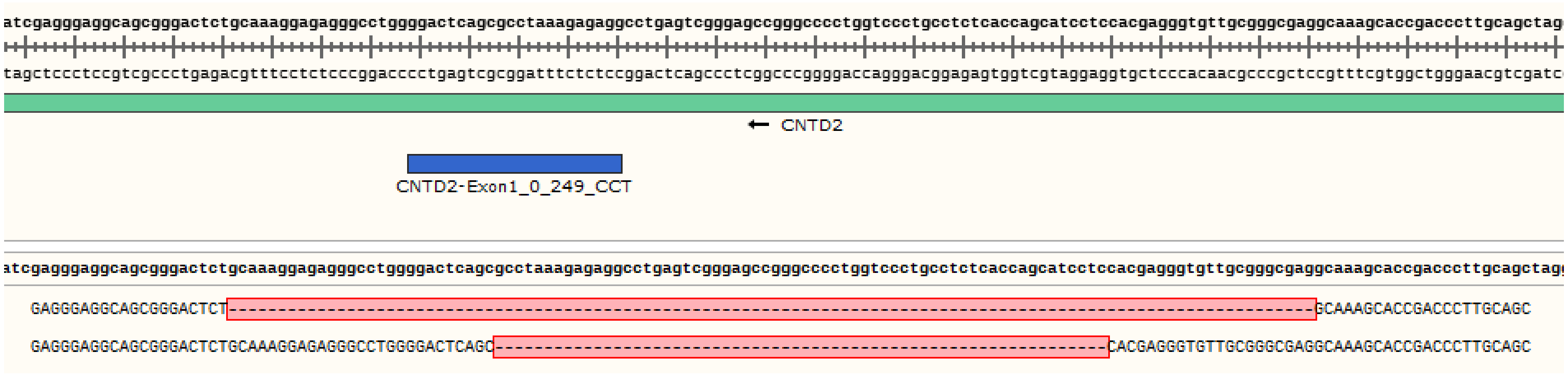

Name	Total # reads	# wt reads(%)	#1-Indel	#1-Reads(%)	#2-Indel	#2-Reads(%)
CNTD2 4-B5	3929	1 (0.0%)	-110	2621 (66.7%)	-62	1306 (33.2%)

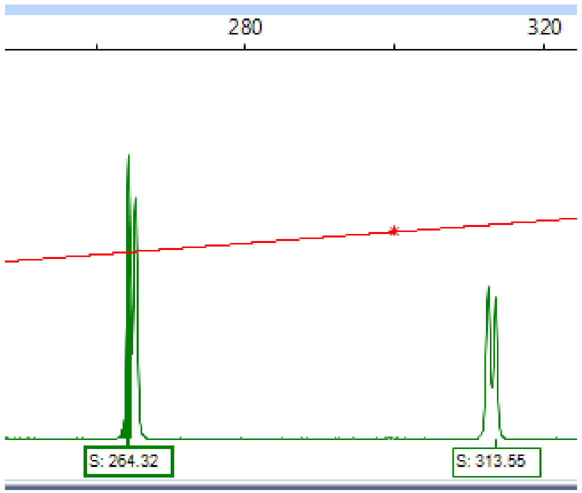

Clone ID	Total # reads	# wt reads(%)	#1-Indel	#1-Reads(%)	#2-Indel	#2-Reads(%)
CNTD2 4-B5	1862	0 (0.0%)	-110	1026 (55.1%)	-62	814 (43.7%)

Clone 002P (-32/+1)

NGS 1, fragment analysis, NGS 2 (cell bank)

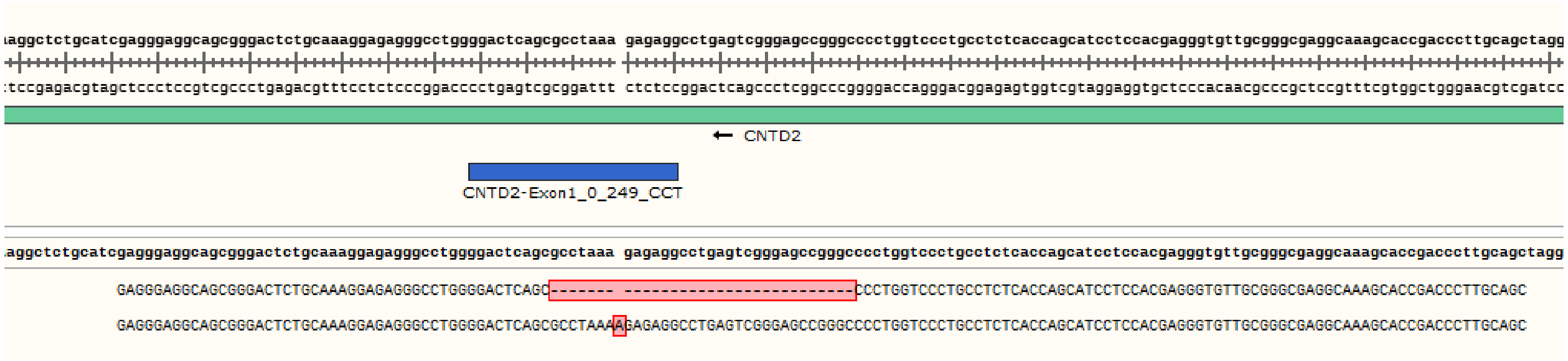

Name	Total # reads	# wt reads(%)	#1-Indel	#1-Reads(%)	#2-Indel	#2-Reads(%)
CNTD2 4-B2	2434	1 (0.0%)	-32	1395 (57.3%)	1	1035 (42.5%)

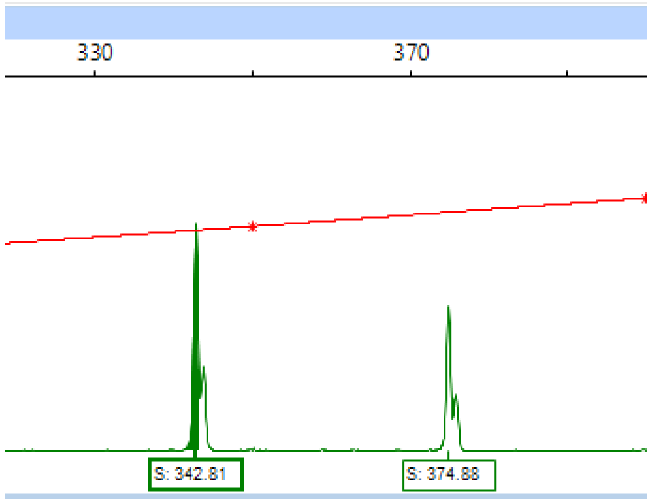

Clone ID	Batavia name	Total # reads	# wt reads(%)	#1-Indel	#1-Reads(%)	#2-Indel	#2-Reads(%)
CNTD2 4-B2	002P	1287	0 (0.0%)	-32	734 (57.0%)	1	527 (40.9%)

Clone 003P (-28/-17)

NGS 1, fragment analysis, NGS 2 (cell bank)

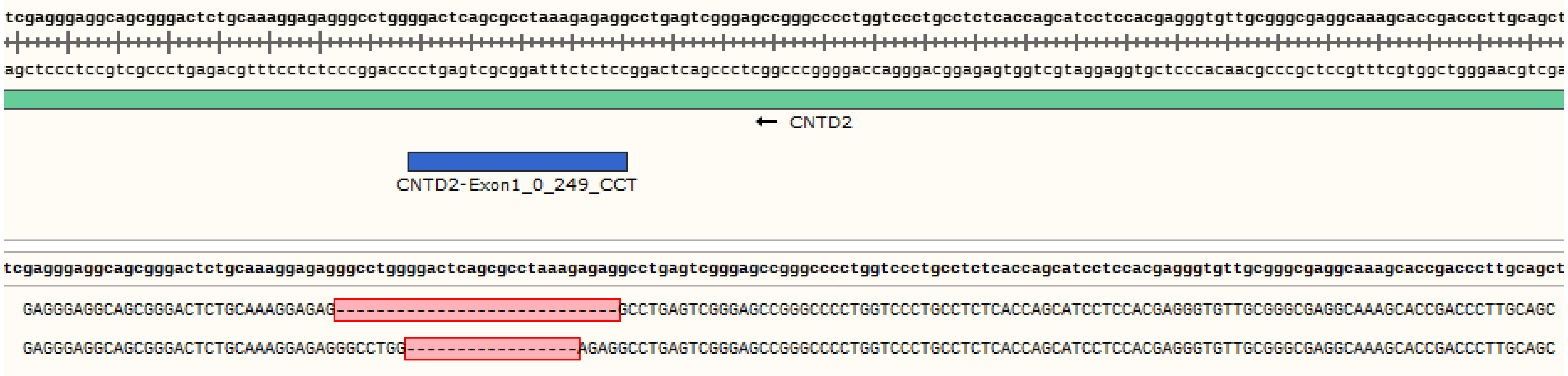

Name	Total # reads	# wt reads(%)	#1-Indel	#1-Reads(%)	#2-Indel	#2-Reads(%)
CNTD2 4-G9	3453	0 (0.0%)	-28	1877 (54.4%)	-17	1571 (45.5%)

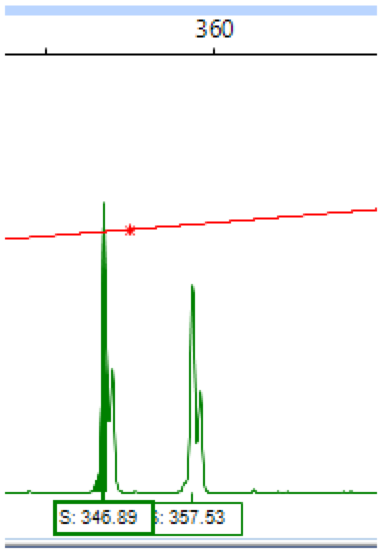

Clone ID	Total # reads	# wt reads(%)	#1-Indel	#1-Reads(%)	#2-Indel	#2-Reads(%)
CNTD2 4-G9	1581	0 (0.0%)	-28	776 (49.1%)	-17	770 (48.7%)

Clone 004P (-25/+1)

NGS 1, fragment analysis, NGS 2 (cell bank)

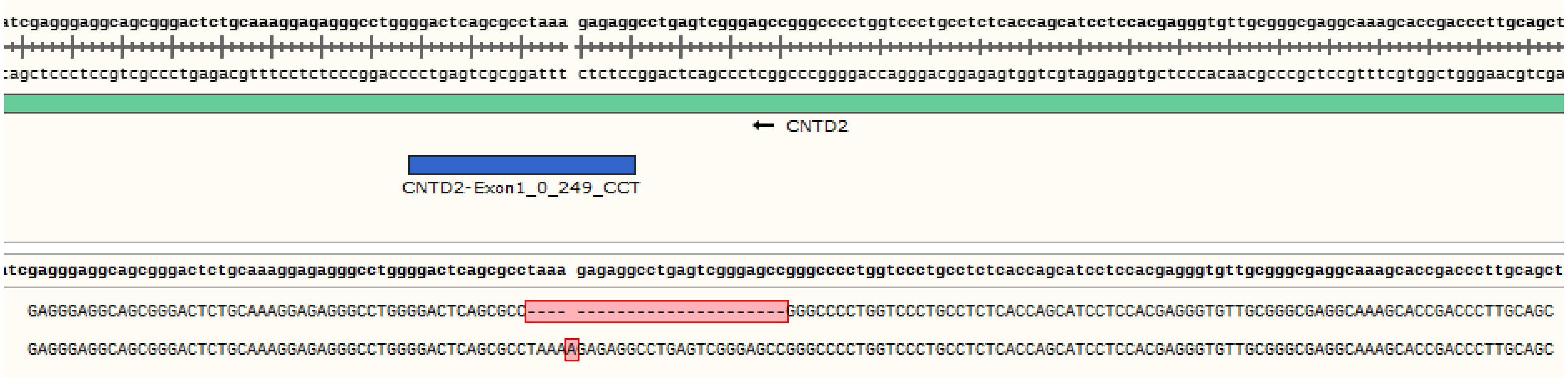

Name	Total # reads	# wt reads(%)	#1-Indel	#1-Reads(%)	#2-Indel	#2-Reads(%)
CNTD2 4-F1	2464	0 (0.0%)	-25	1356 (55.0%)	1	1108 (45.0%)

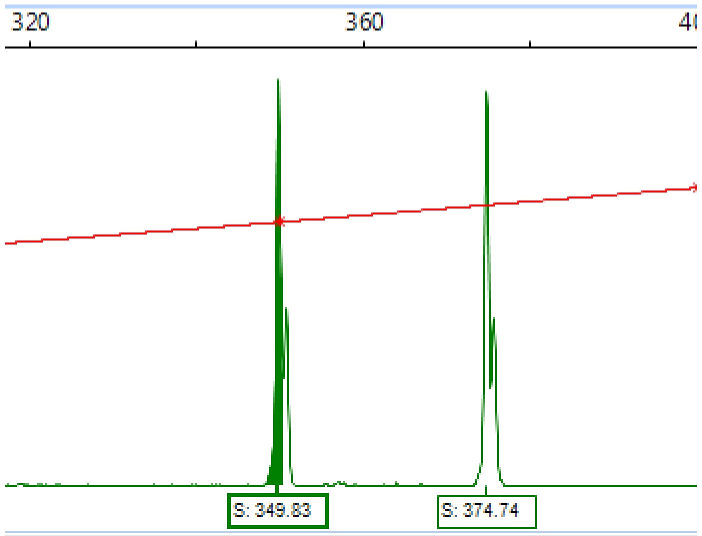

Clone ID	Total # reads	# wt reads(%)	#1-Indel	#1-Reads(%)	#2-Indel	#2-Reads(%)
CNTD2 4-F1	1580	0 (0.0%)	-25	867 (54.9%)	1	669 (42.3%)

Clone 005P (-13/+1)

NGS 1, fragment analysis, NGS 2 (cell bank)

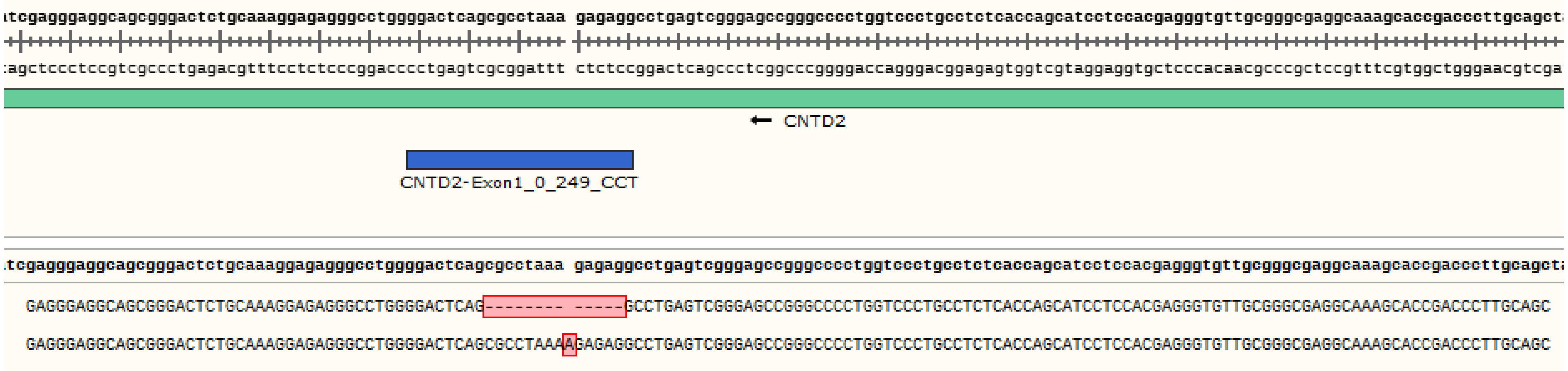

Name	Total # reads	# wt reads(%)	#1-Indel	#1-Reads(%)	#2-Indel	#2-Reads(%)
CNTD2 4-C11	2488	1 (0.0%)	-13	1434 (57.6%)	1	1052 (42.3%)

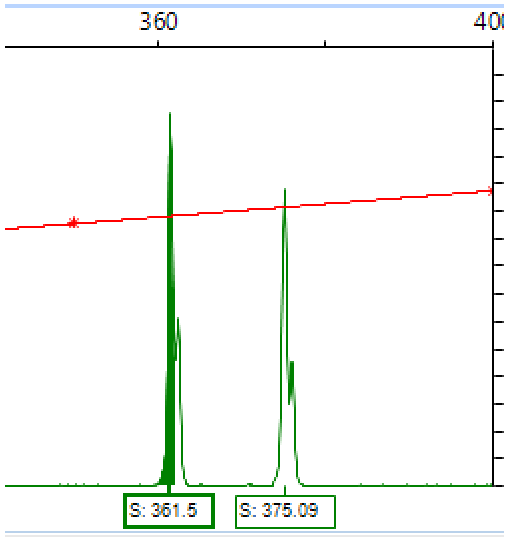

Clone ID	Total # reads	# wt reads(%)	#1-Indel	#1-Reads(%)	#2-Indel	#2-Reads(%)
CNTD2 4-C11	826	0 (0.0%)	1	469 (56.8%)	-13	321 (38.9%)

Clone 006P (-11/-1)

NGS 1, fragment analysis, NGS 2 (cell bank)

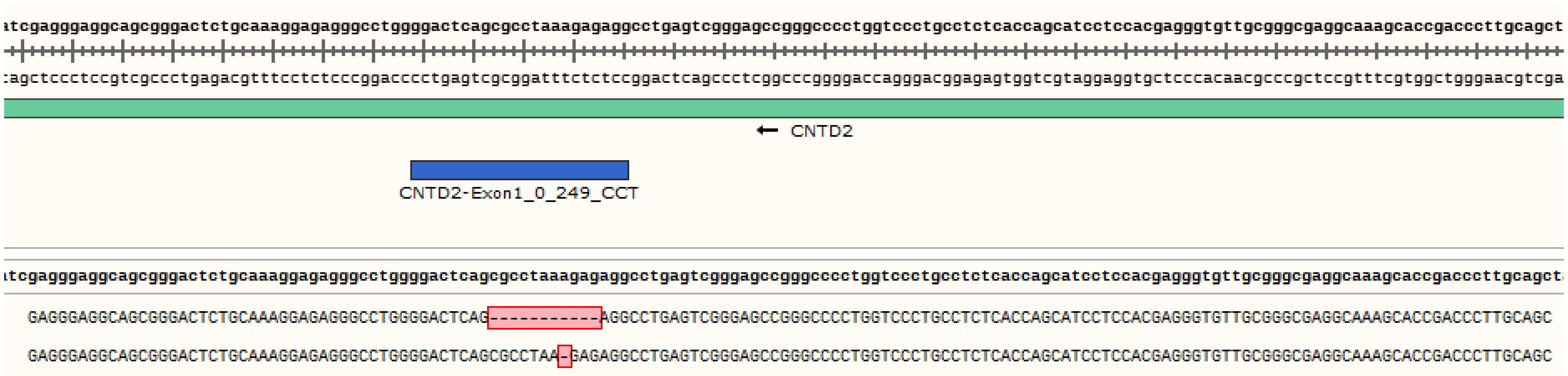

Name	Total # reads	# wt reads(%)	#1-Indel	#1-Reads(%)	#2-Indel	#2-Reads(%)
CNTD2 4-B11	2767	0 (0.0%)	-11	1588 (57.4%)	-1	1174 (42.4%)

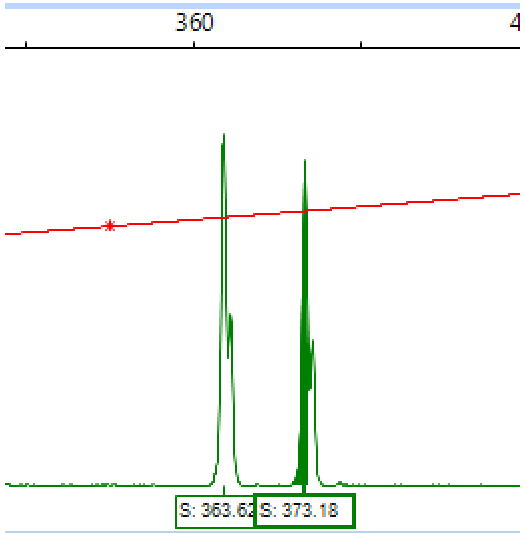

Clone ID	Total # reads	# wt reads(%)	#1-Indel	#1-Reads(%)	#2-Indel	#2-Reads(%)
CNTD2 4-B11	1149	0 (0.0%)	-11	562 (48.9%)	-1	545 (47.4%)

Clone 007P (-1/+1)

NGS 1, fragment analysis, NGS 2 (cell bank)

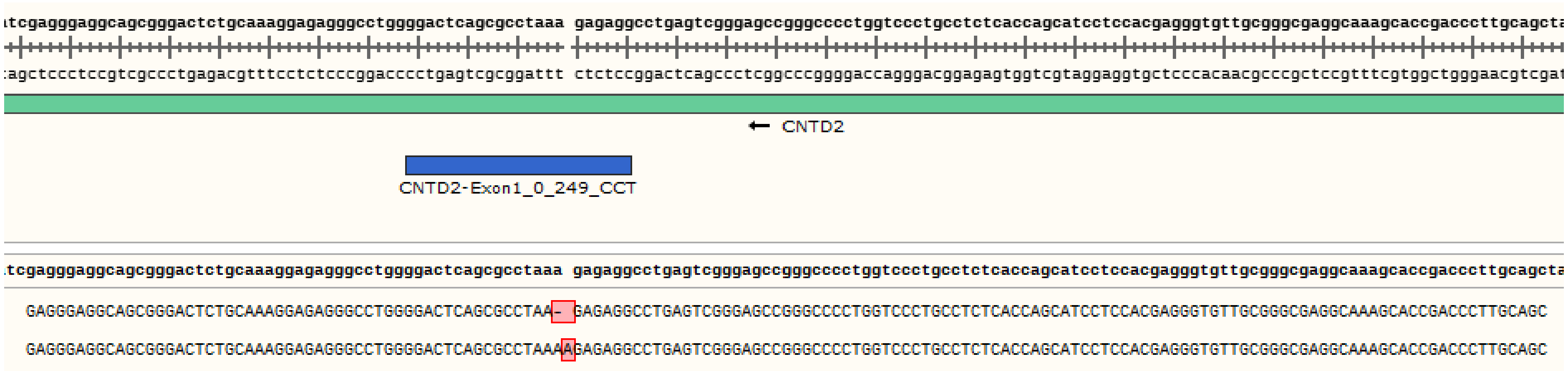

Name	Total # reads	# wt reads(%)	#1-Indel	#1-Reads(%)	#2-Indel	#2-Reads(%)
CNTD2 4-G12	908	0 (0.0%)	-1	475 (52.3%)	1	429 (47.2%)

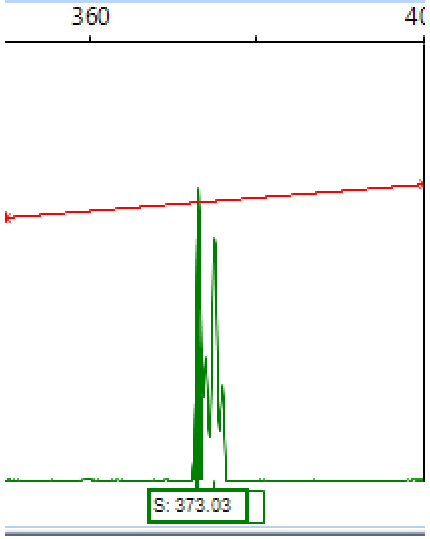

Clone ID	Total # reads	# wt reads(%)	#1-Indel	#1-Reads(%)	#2-Indel	#2-Reads(%)
CNTD2 4-G12	979	0 (0.0%)	1	482 (49.2%)	-1	469 (47.9%)

Clone 008P (-1/-14/-62)

NGS 1, NGS2 (cell bank) revealed contamination with other CNTD2 knockout clone

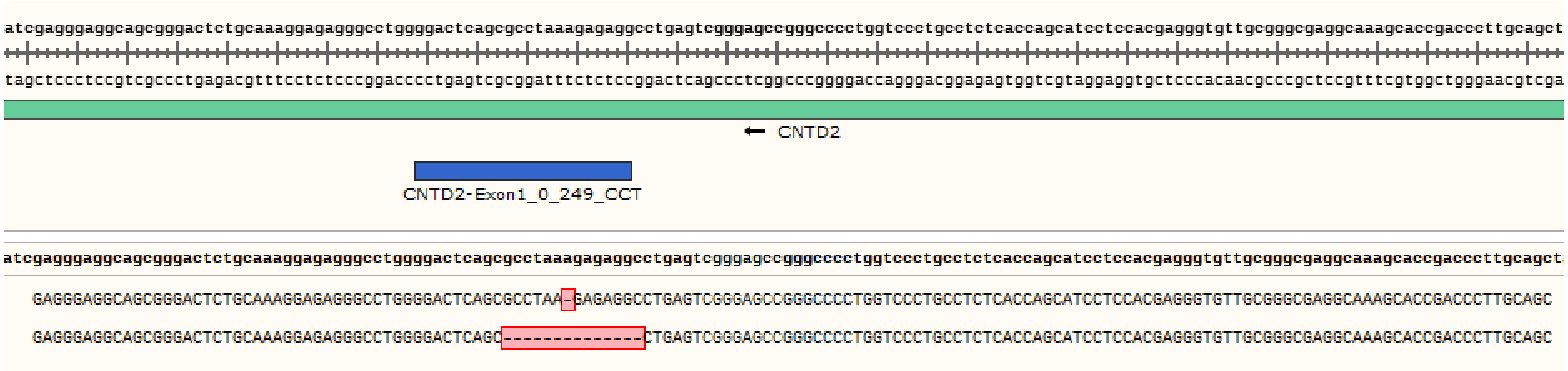

Name	Total # reads	# wt reads(%)	#1-Indel	#1-Reads(%)	#2-Indel	#2-Reads(%)
CNTD2 4-C5	1682	1 (0.1%)	-1	869 (51.7%)	-14	808 (48.0%)

Clone ID	Clone ID	Total # reads	# wt reads(%)	#1-Indel	#1-Reads(%)	#2-Indel	#2-Reads(%)	#3-Indel	#3-Reads(%)
CNTD2 4-C5	008P	773	0 (0.0%)	-62	633 (81.9%)	-14	70 (9.1%)	-1	55 (7.1%)

Clone 013P (+1/+1)

NGS 1, sequence, fragment analysis, NGS 2 (cell bank)

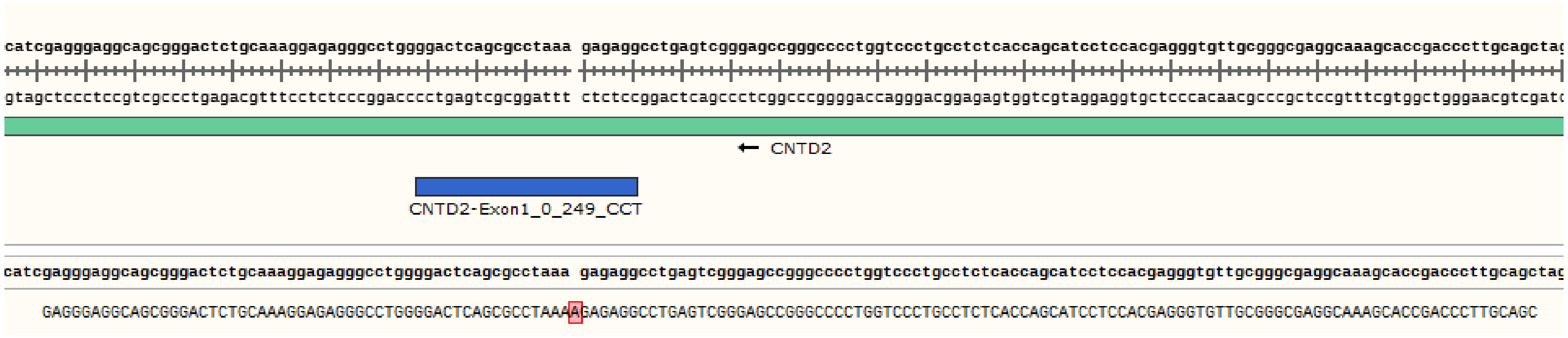

Name	Total # reads	# wt reads(%)	#1-Indel	#1-Reads(%)	#2-Indel	#2-Reads(%)
CNTD2 4-C2	2010	2 (0.1%)	1	2002 (99.6%)	0	6 (0.3%)

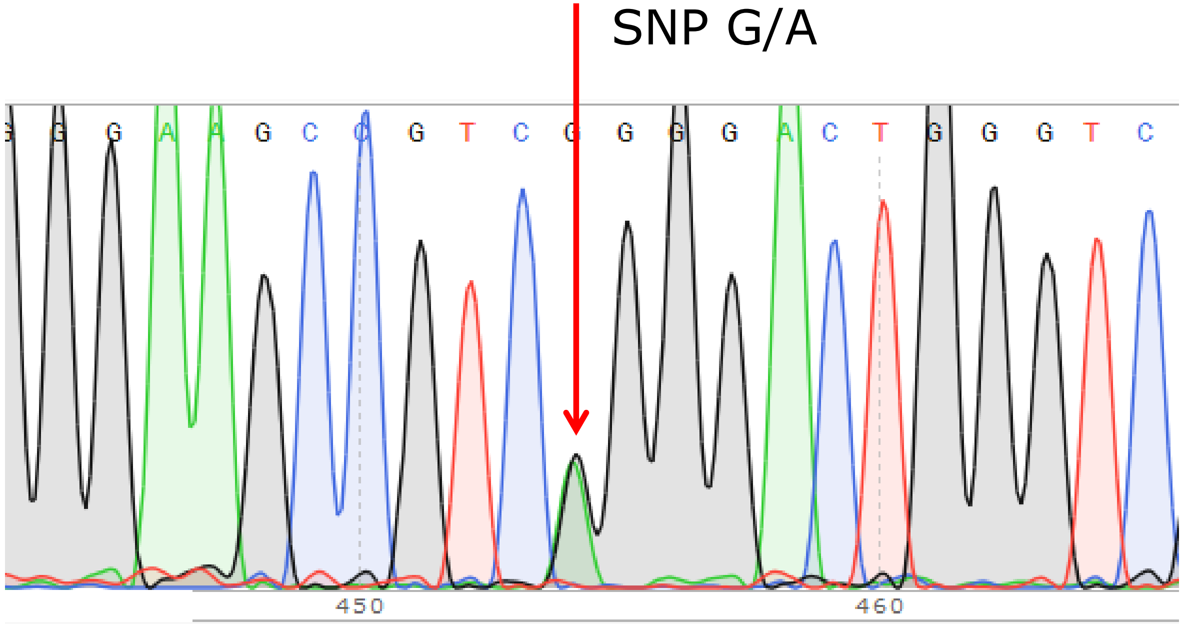

Clone 013P (+1/+1)

NGS 1, sequence, fragment analysis, NGS 2 (cell bank)

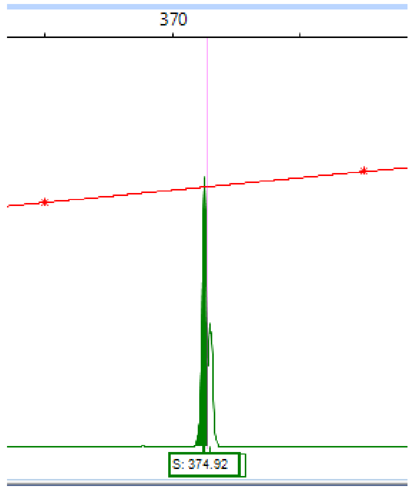

Clone ID	Total # reads	# wt reads(%)	#1-Indel	#1-Reads(%)	#2-Indel	#2-Reads(%)
CNTD2 4-C2	1189	2 (0.2%)	1	1167 (98.1%)	0	20 (1.7%)

Clone 014P (-11/-1160)

NGS 1, sequence, fragment analysis, NGS 2 (cell bank)

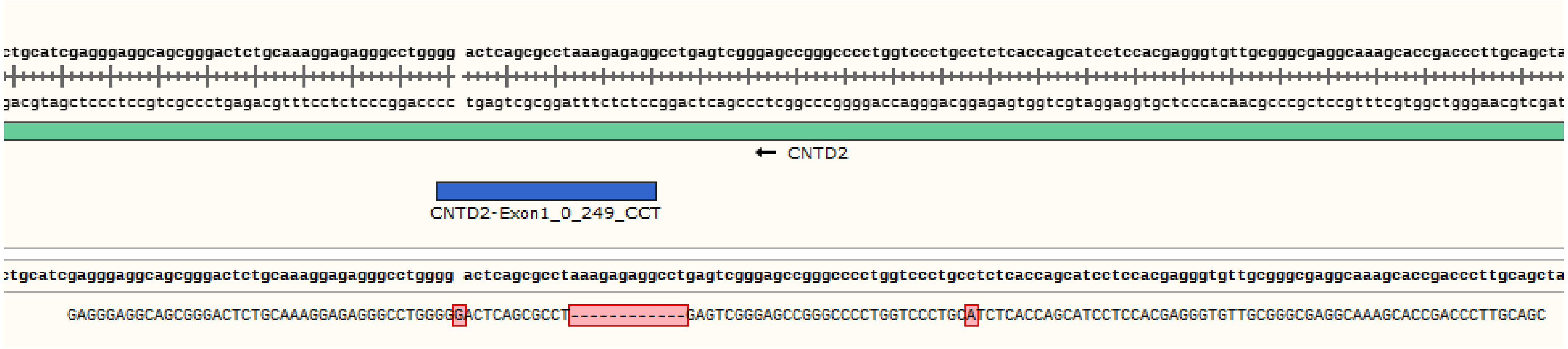

Name	Total # reads	# wt reads(%)	#1-Indel	#1-Reads(%)	#2-Indel	#2-Reads(%)
CNTD2 4-F9	1855	0 (0.0%)	-11	1849 (99.7%)	-12	4 (0.2%)

Clone 014P (-11/-1160)

NGS 1, sequence, fragment analysis, NGS 2 (cell bank)

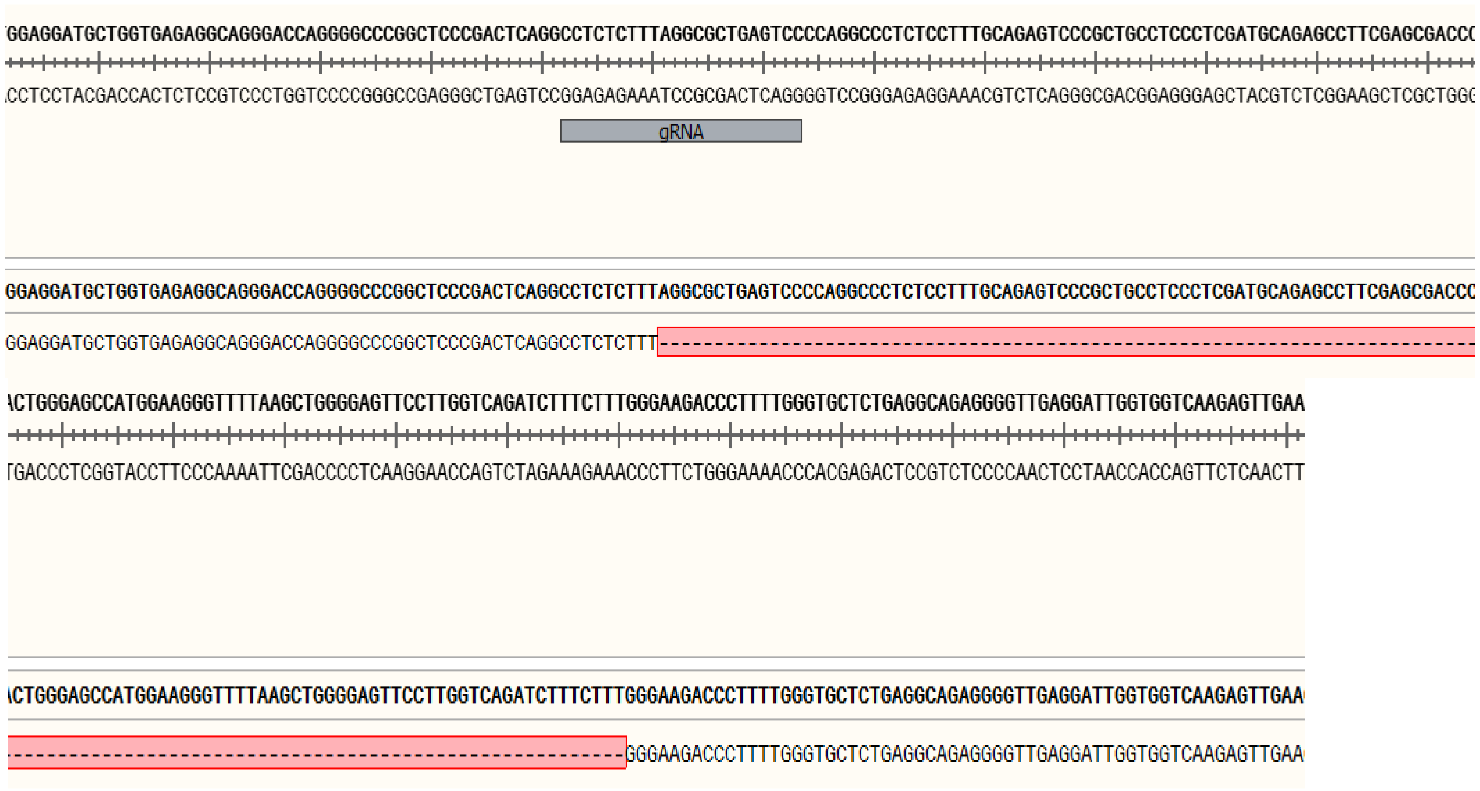

Deleted sequence -1160bp:

AGGCGCTGAGTCCCCAGGCCCTCTCCTTTGCAGAGTCCCGCTGCCTCCCTCGATGCAGAGCCTTCGAGCGACCCAGTCCCCGACGGCTTCCCCTCGGGCCCCAGTG
TCTCCCCAAGACGCCTGGAGAGGCCGCCGGGACTGGAGGAGGCGCTGAGCTCGCTGGGGCTGCAGGGAGAACTCGAGTACGCCGGGGACATCTTCGCCGAAGCC
ATGGTGAGCCGACCCGCTGCCTGCCCTGGTGAGGCCGACGCCAGCCCCCCACCCAGCACCCACTCAACACCACCTCTGAACTCCCACCGCTCCAGCCTCTCTCCTCC
CCATCACTGGCGGCCGGCGTTCCTTCTCTGCAACGTCCAGGCTGTTGTTTTGTTTTCACAGAGCACCGTTTCTACCGCTGGGAATGCCCTTTCCCACCGTCCCTGGGC
TCTTTACAGCCCCTCCTGGGGTGTTCGTGGTCCCCCTACTTATCCTCCACATTTATTCAACAAATATTTAATGGTTTAATAGCAAATGTTTTAAGCGCTGGAGATGCTGC
AGTGCTGGCCTCGTTCTGGGGAGATGAGCAAACACATAGGACAGTTAGGGAAAGGCCATCCACACCACAGCGTTGCCCTCGCCAGCCTTCAGACATCCCTGTGAGA
GGGGTGCTGTGGCAGGAAGACTGAAGGGAGGAGTTTATTTAACGTCTTGCCAGCGACTCTTCTGGGCCAGGATCTCTGTTAGGACCTGAGAGACACCTGTGTTCTGG
GCAGAAGTCCCTGCCCTCCTGGGACTGGCAGCCTAGCGGCCAAATAGACAAAGCACACAGGGAGACATCTTCAGAGCGAAAATGCTGAGACAAAGAGCTAGTGATC
GGGTGGGAGGAGTATAGATCCAGTCATGGAGATGTTAGTTGAATGACAGAAGGGCCATGCAAGGAGTTGGGGAAGGACCCTCTCTGTAGAAGAAATAGGAAGTGCA
AAGGAGCTGGGGTAGGAATGGGCTTGATATAGTCTAGGAGCCAAGAAGAAGGTGGCTGGGGCTGATGTGTGGGTTGTCGGGGGAGAGAGGGAAGAGGTGATGTT
GAAGCAGAGCCTGAAAGAAATTAGAGTTTTCTCCAAGGGTACTGGGAGCCATGGAAGGGTTTTAAGCTGGGGAGTTCCTTGGTCAGATCTTTCTTT

Clone 014P (-11/-1160)

NGS 1, sequence, fragment analysis, NGS 2 (cell bank)

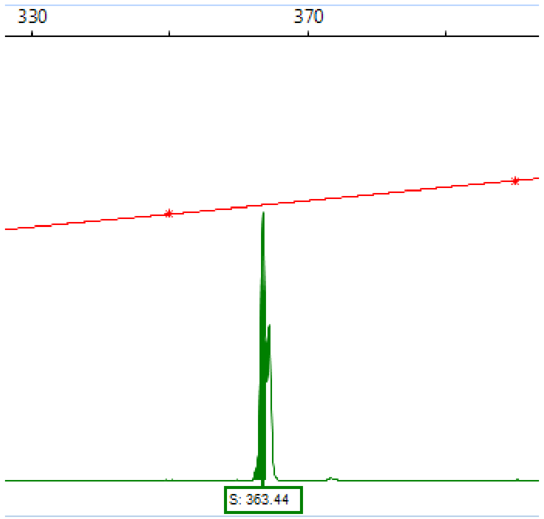

Clone ID	Total # reads	# wt reads(%)	#1-Indel	#1-Reads(%)	#2-Indel	#2-Reads(%)
CNTD2 4-F9	1589	0 (0.0%)	-11	1557 (98.0%)	-12	27 (1.7%)

Clone 039P (-13/-5)

NGS 1, NGS 2 (cell bank)

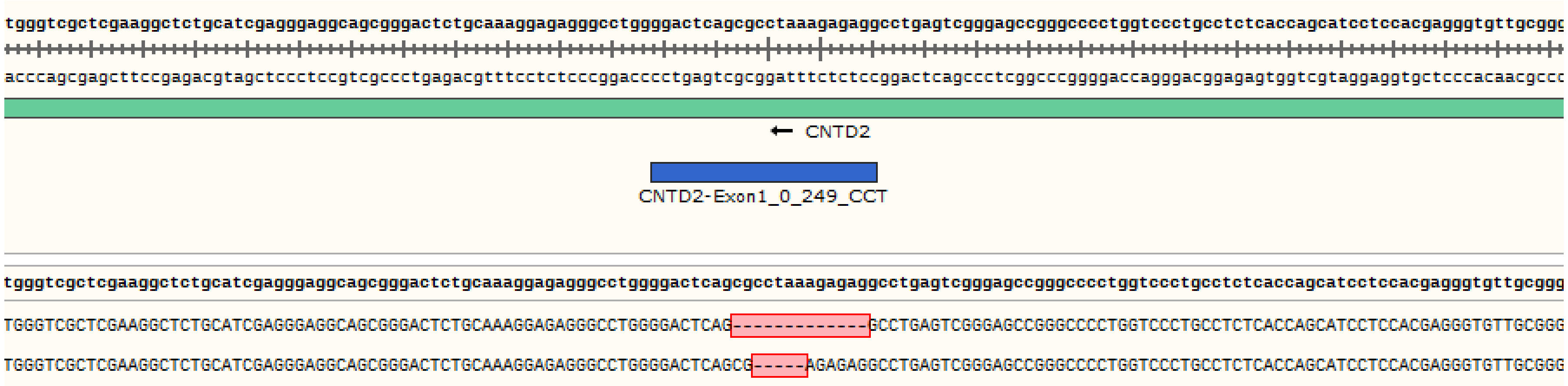

Name	Batavia name	Total # reads	# wt reads(%)	#1-Indel	#1-Reads(%)	#2-Indel	#2-Reads(%)
CNTD2 from CNT/NEU 1E6 data CNTD2		1536	0 (0.0%)	-13	829 (54.0%)	-5	678 (44.1%)
CNTD2 from CNT/NEU 1E6 data NEU2	039P	1074	995 (92.6%)	0	1055 (98.2%)	-1	17 (1.6%)

Clone ID	Clone ID	Total # reads	# wt reads(%)	#1-Indel	#1-Reads(%)	#2-Indel	#2-Reads(%)
CNTD2 from CNT/NEU 1E6 data CNTD2	039P	14322	0 (0.0%)	-13	7816 (54.6%)	-5	6214 (43.4%)
CNTD2 from CNT/NEU 1E6 data NEU2	039P	4912	4578 (93.2%)	0	4828 (98.3%)	-1	73 (1.5%)

Clone 009P (-47/-16)

NGS 1, fragment analysis, NGS 2 (cell bank)

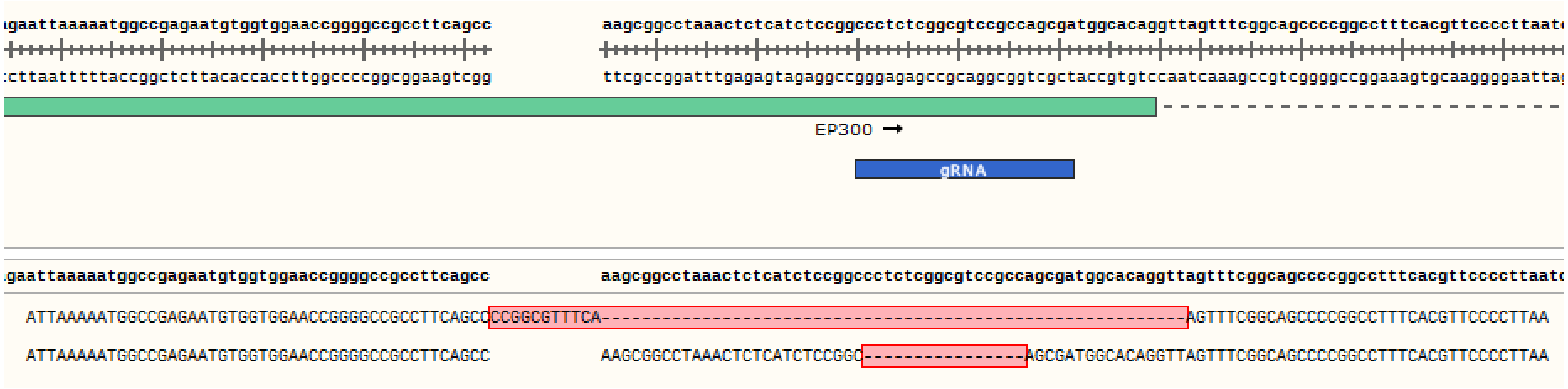

Name	Total # reads	# wt reads(%)	#1-Indel	#1-Reads(%)	#2-Indel	#2-Reads(%)
EP300 4-E12	878	0 (0.0%)	-47	500 (56.9%)	-16	367 (41.8%)

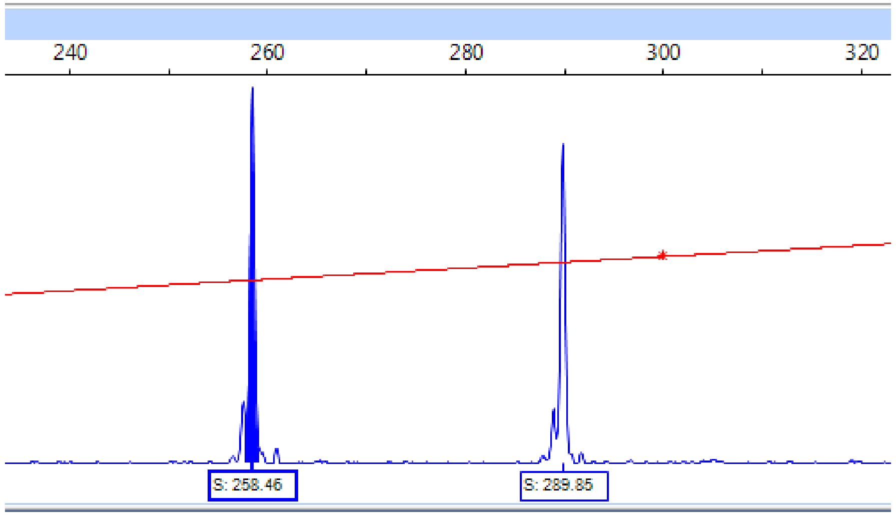

Clone ID	Total # reads	# wt reads(%)	#1-Indel	#1-Reads(%)	#2-Indel	#2-Reads(%)
EP300 4-E12	3029	5 (0.2%)	-47	1636 (54.0%)	-16	1376 (45.4%)

Clone 010P (-14/-11)

NGS 1, fragment analysis, NGS 2 (cell bank)

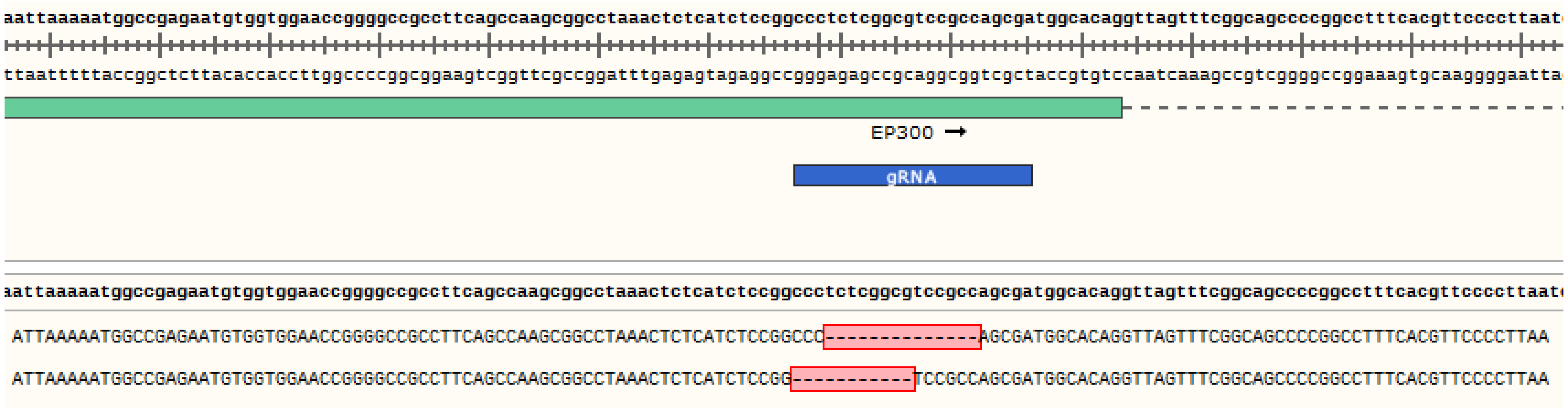

Name	Total # reads	# wt reads(%)	#1-Indel	#1-Reads(%)	#2-Indel	#2-Reads(%)
EP300 4-B6	3417	0 (0.0%)	-14	1771 (51.8%)	-11	1587 (46.4%)

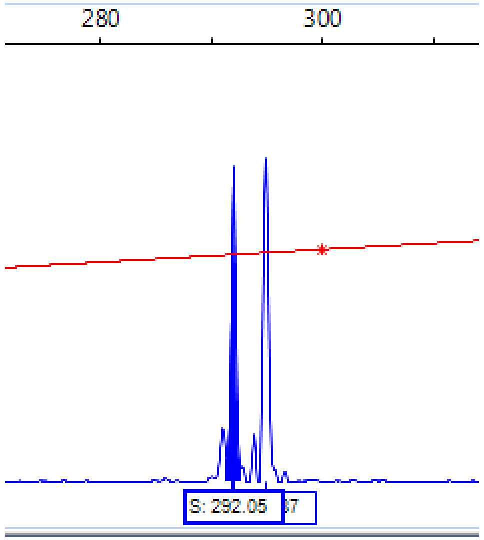

Clone ID	Total # reads	# wt reads(%)	#1-Indel	#1-Reads(%)	#2-Indel	#2-Reads(%)
EP300 4-B6	3037	1 (0.0%)	-14	1517 (50.0%)	-11	1492 (49.1%)

Clone 011P (-7/+25)

NGS 1, fragment analysis, NGS 2 (cell bank)

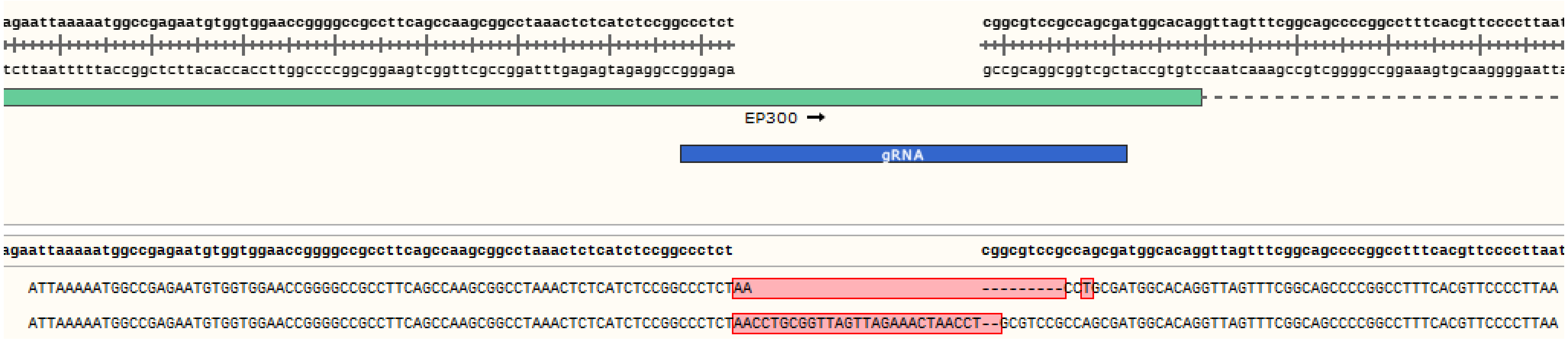

Name	Total # reads	# wt reads(%)	#1-Indel	#1-Reads(%)	#2-Indel	#2-Reads(%)
EP300 4-G12	772	0 (0.0%)	-7	458 (59.3%)	25	298 (38.6%)

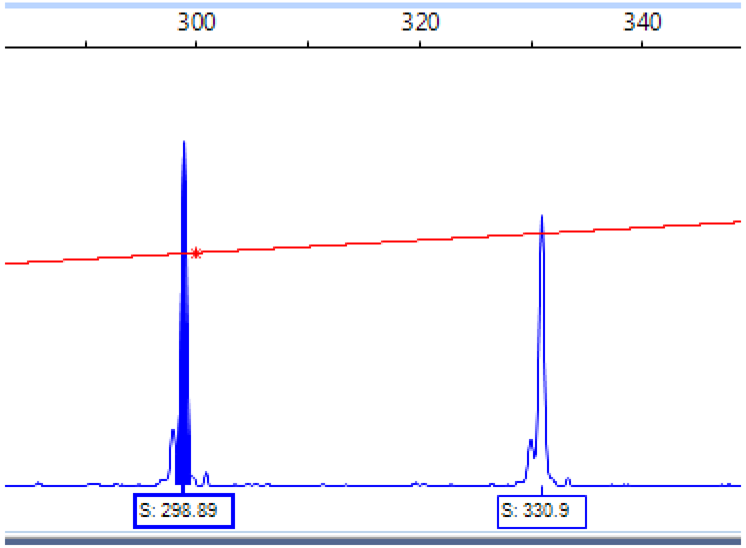

Clone ID	Total # reads	# wt reads(%)	#1-Indel	#1-Reads(%)	#2-Indel	#2-Reads(%)
EP300 4-G12	3832	4 (0.1%)	-7	2098 (54.7%)	25	1686 (44.0%)

Clone 012P (-31)

NGS 1, 3kb PCR, NGS 2 (cell bank)

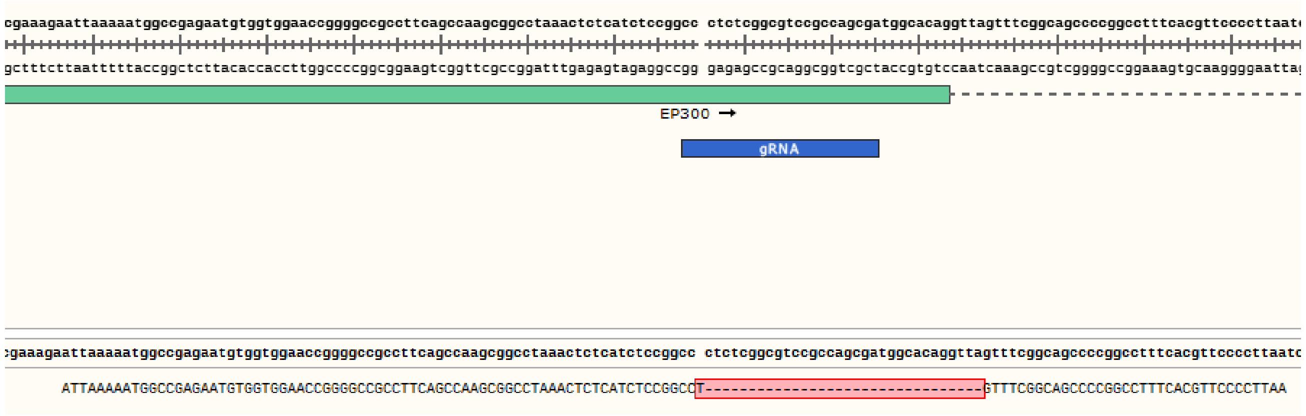

Name	Clone ID	Total # reads	# wt reads(%)	#1-Indel	#1-Reads(%)	#2-Indel	#2-Reads(%)
EP300 4-G10	012P	1803	1 (0.1%)	-31	1786 (99.1%)	-32	16 (0.9%)

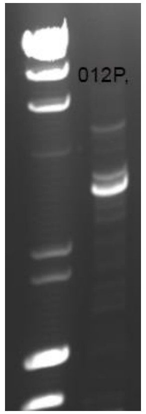

Clone ID	Clone ID	Total # reads	# wt reads(%)	#1-Indel	#1-Reads(%)	#2-Indel	#2-Reads(%)
EP300 4-G10	012P	3405	0 (0.0%)	-31	3364 (98.8%)	-32	36 (1.1%)

Clone 015P (-25/+1)

NGS 1, NGS 2 (cell bank)

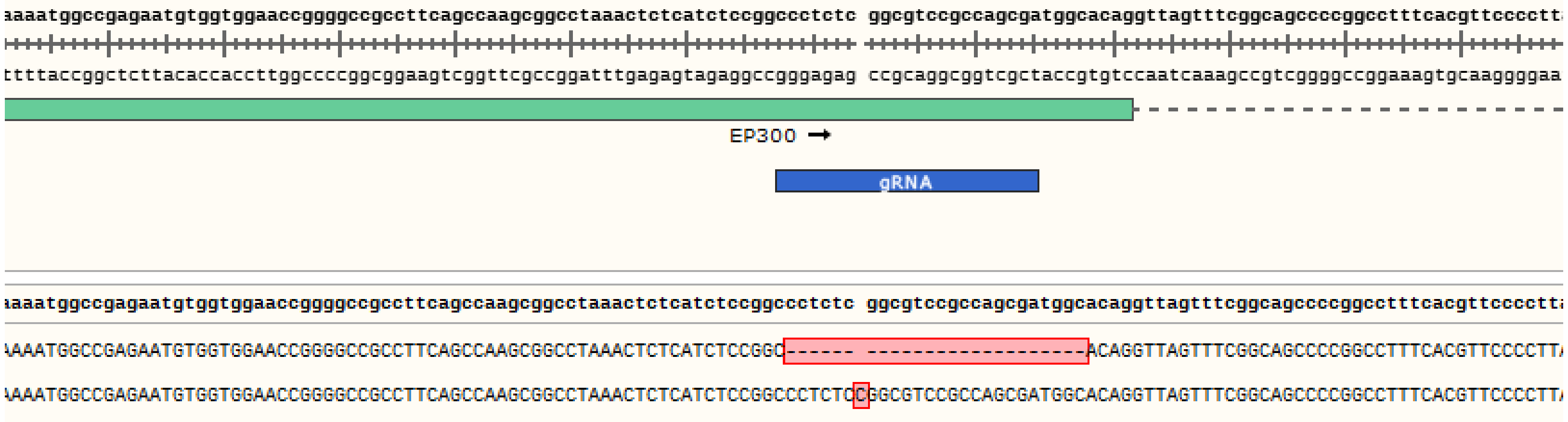

Name	Batavia name	Total # reads	# wt reads(%)	#1-Indel	#1-Reads(%)	#2-Indel	#2-Reads(%)
EP300 2D11	015P	2179	0 (0.0%)	-25	1162 (53.3%)	1	1006 (46.2%)

Clone ID	Batavia name	Total # reads	# wt reads(%)	#1-Indel	#1-Reads(%)	#2-Indel	#2-Reads(%)
EP300 2D11	015P	2420	0 (0.0%)	-25	1340 (55.4%)	1	1055 (43.6%)

Clone 016P (-17/-68)

NGS 1, topo sequence, NGS 2 (cell bank)

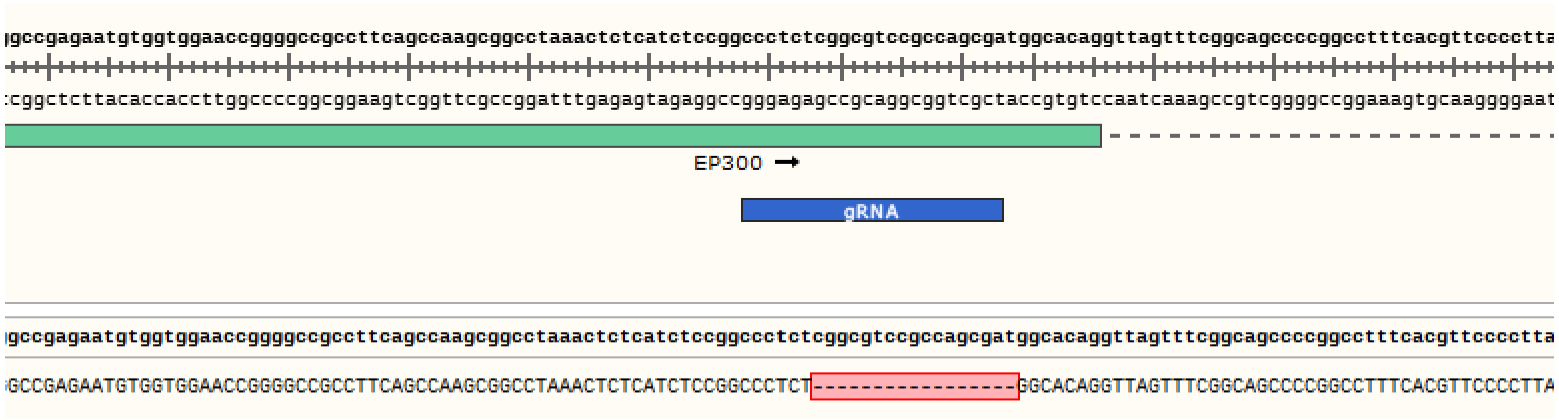

Name	Batavia name	Total # reads	# wt reads(%)	#1-Indel	#1-Reads(%)	#2-Indel	#2-Reads(%)
EP300 2D1	016P	1863	1 (0.1%)	-17	1853 (99.5%)	-18	9 (0.5%)

Topo -17/-68

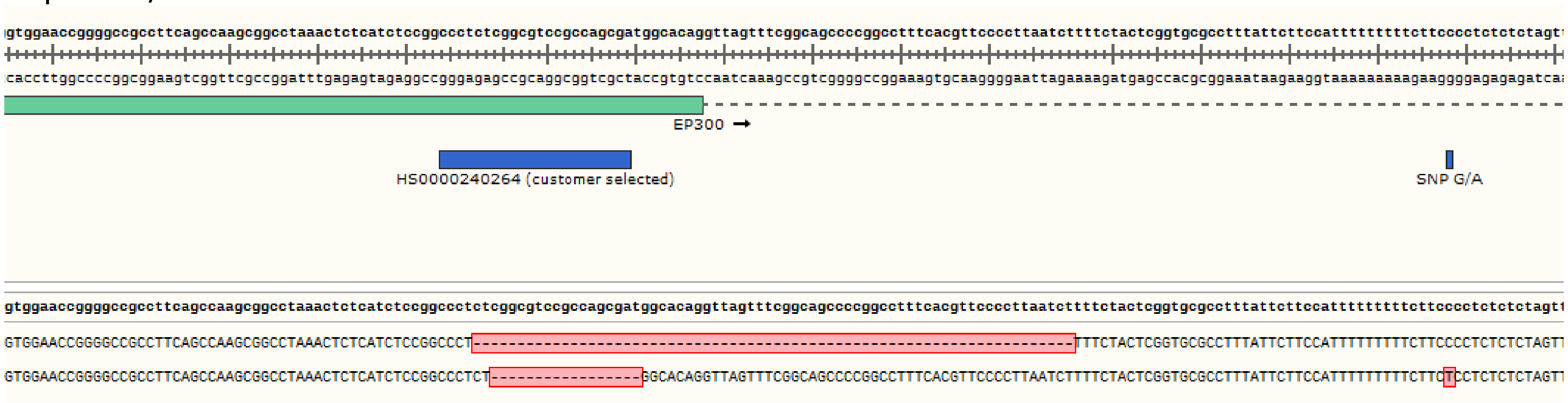

Clone ID	Batavia name	Total # reads	# wt reads(%)	#1-Indel	#1-Reads(%)	#2-Indel	#2-Reads(%)
EP300 2D1	016P	2932	3 (0.1%)	-68	1593 (54.3%)	-17	1304 (44.5%)

In NGS run 2 different primers were able to find -68bp

Clone 040P (-2/+2)

NGS 1, NGS 2 (cell bank)

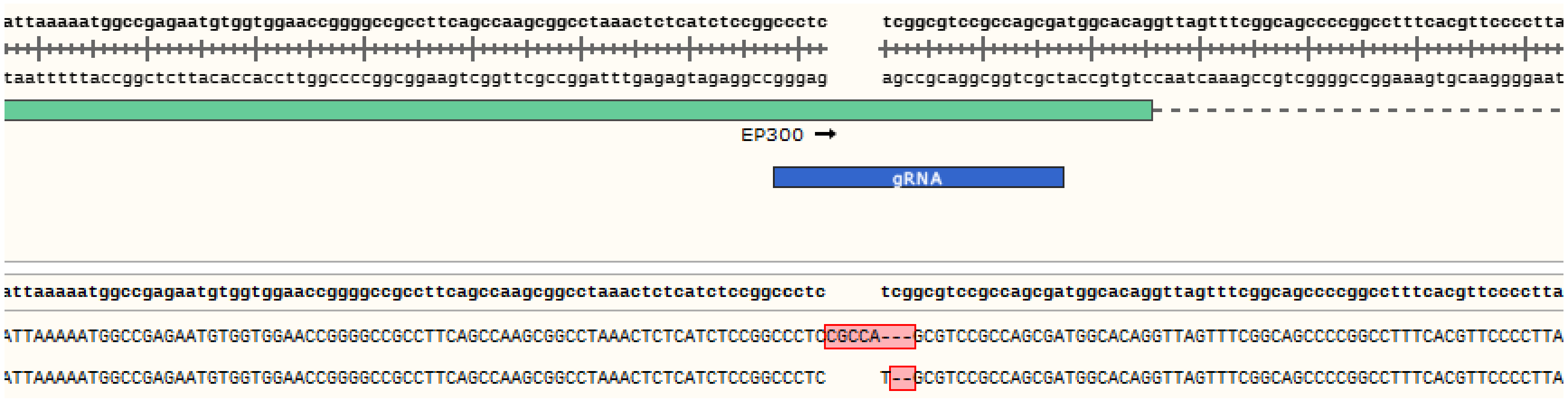

Name	Batavia name	Total # reads	# wt reads(%)	#1-Indel	#1-Reads(%)	#2-Indel	#2-Reads(%)
EP300 from EP/NEU 2C12 data EP300		3893	0 (0.0%)	2	1942 (49.9%)	-2	1921 (49.3%)
EP300 from EP/NEU 2C12 data NEU2	040P	1387	1279 (92.2%)	0	1358 (97.9%)	-1	25 (1.8%)

Clone ID	Clone ID	Total # reads	# wt reads(%)	#1-Indel	#1-Reads(%)	#2-Indel	#2-Reads(%)
EP300 from EP/NEU 2C12 data EP300		15294	0 (0.0%)	-2	7662 (50.1%)	2	7411 (48.5%)
EP300 from EP/NEU 2C12 data NEU2	040P	5529	5168 (93.5%)	0	5407 (97.8%)	-1	111 (2.0%)

Clone 043P (-16/+1)

NGS 1, NGS 2 (cell bank)

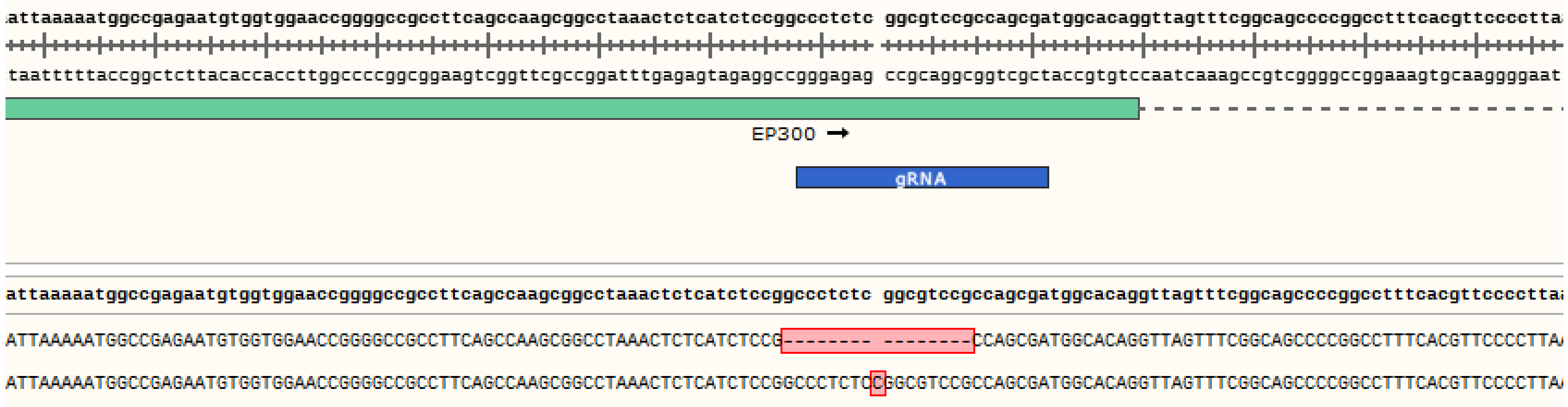

Name	Batavia name	Total # reads	# wt reads(%)	#1-Indel	#1-Reads(%)	#2-Indel	#2-Reads(%)
EP300 from EP/NEU 3C8 data EP300		3379	0 (0.0%)	-16	1747 (51.7%)	1	1611 (47.7%)
EP300 from EP/NEU 3C8 data NEU2	043P	1844	1727 (93.7%)	0	1817 (98.5%)	-1	21 (1.1%)

Clone ID	Clone ID	Total # reads	# wt reads(%)	#1-Indel	#1-Reads(%)	#2-Indel	#2-Reads(%)
EP300 from EP/NEU 3C8 data EP300		10496	1 (0.0%)	-16	5641 (53.7%)	1	4730 (45.1%)
EP300 from EP/NEU 3C8 data NEU2	043P	4302	4042 (94.0%)	0	4221 (98.1%)	-1	72 (1.7%)

Clone 044P (-41/-19)

NGS 1, NGS 2 (cell bank)

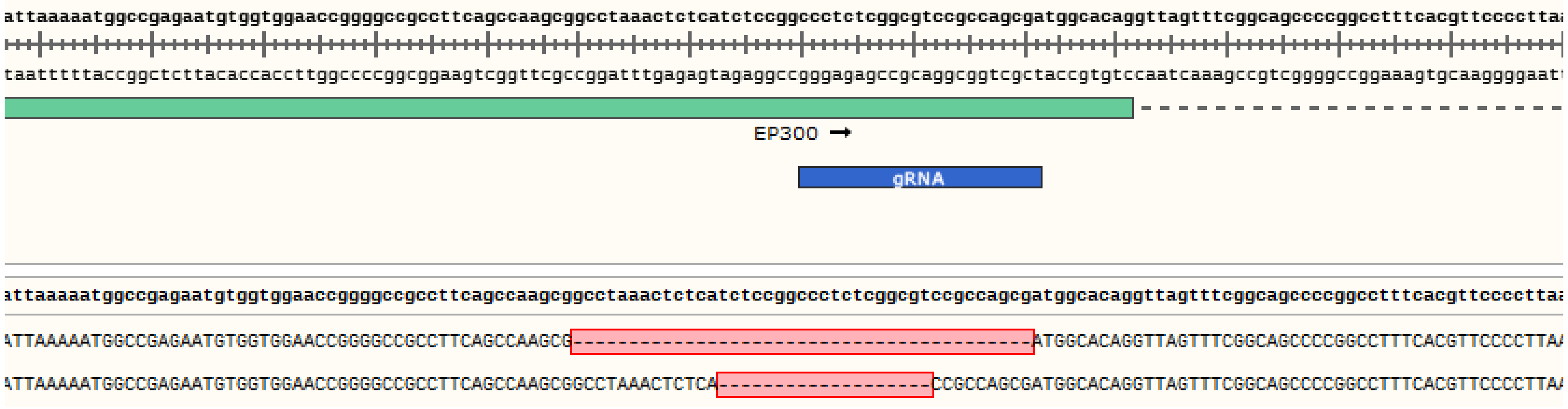

Name	Batavia name	Total # reads	# wt reads(%)	#1-Indel	#1-Reads(%)	#2-Indel	#2-Reads(%)
EP300 from EP/NEU 3D4 data EP300		3848	0 (0.0%)	-41	2131 (55.4%)	-19	1695 (44.0%)
EP300 from EP/NEU 3D4 data NEU2	044P	1758	1628 (92.6%)	0	1712 (97.4%)	-1	43 (2.4%)

Clone ID	Clone ID	Total # reads	# wt reads(%)	#1-Indel	#1-Reads(%)	#2-Indel	#2-Reads(%)
EP300 from EP/NEU 3D4 data EP300		10762	0 (0.0%)	-41	6226 (57.9%)	-19	4428 (41.1%)
EP300 from EP/NEU 3D4 data NEU2	044P	4906	4577 (93.3%)	0	4790 (97.6%)	-1	100 (2.0%)

Clone 046P (-2/-1)

NGS 1, NGS 2 (cell bank)

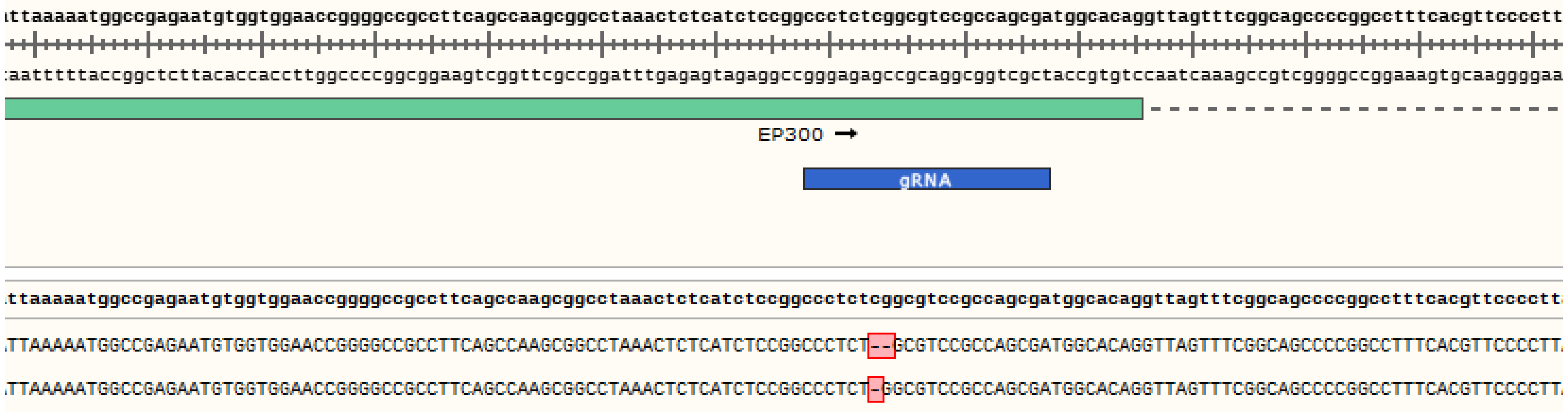

Name	Batavia name	Total # reads	# wt reads(%)	#1-Indel	#1-Reads(%)	#2-Indel	#2-Reads(%)
EP300 from EP/NEU 3E9 data EP300		1313	0 (0.0%)	-2	664 (50.6%)	-1	644 (49.0%)
EP300 from EP/NEU 3E9 data NEU2	046P	996	931 (93.5%)	0	980 (98.4%)	-1	15 (1.5%)

Clone ID	Clone ID	Total # reads	# wt reads(%)	#1-Indel	#1-Reads(%)	#2-Indel	#2-Reads(%)
EP300 from EP/NEU 3E9 data EP300		8331	0 (0.0%)	-1	4188 (50.3%)	-2	4070 (48.9%)
EP300 from EP/NEU 3E9 data NEU2	046P	4261	3932 (92.3%)	0	4168 (97.8%)	-1	79 (1.9%)

Clone 047P (-1/-2)

NGS 1, NGS 2 (cell bank)

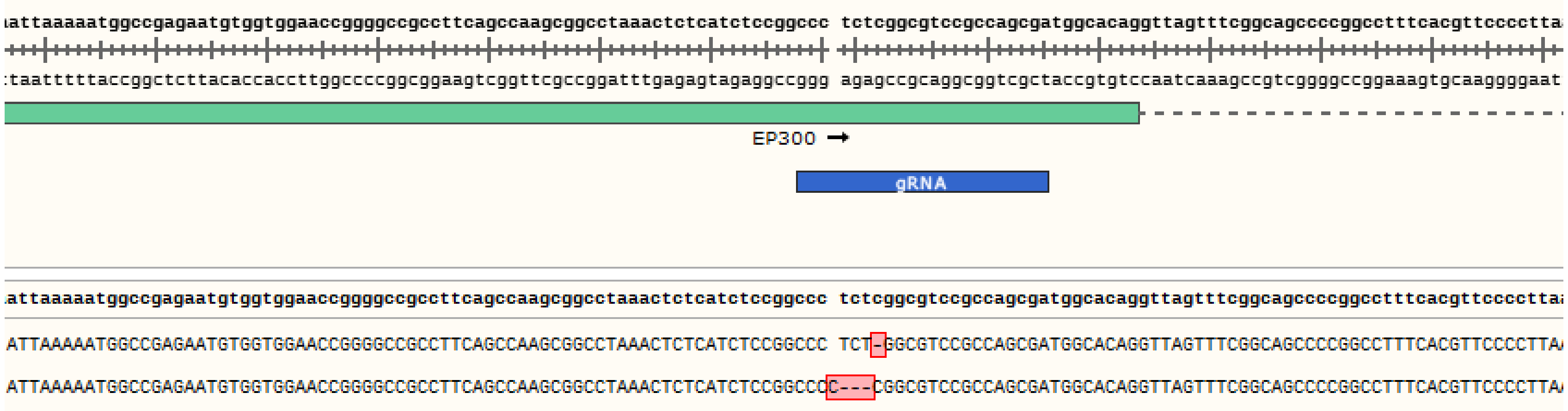

Name	Batavia name	Total # reads	# wt reads(%)	#1-Indel	#1-Reads(%)	#2-Indel	#2-Reads(%)
EP300 from EP/NEU 3H3 data EP300		2767	0 (0.0%)	-1	1470 (53.1%)	-2	1281 (46.3%)
EP300 from EP/NEU 3H3 data NEU2	047P	1814	1670 (92.1%)	0	1789 (98.6%)	-1	23 (1.3%)

Clone ID	Clone ID	Total # reads	# wt reads(%)	#1-Indel	#1-Reads(%)	#2-Indel	#2-Reads(%)
EP300 from EP/NEU 3H3 data EP300		12314	14 (0.1%)	-2	6128 (49.8%)	-1	6055 (49.2%)
EP300 from EP/NEU 3H3 data NEU2	047P	5706	5311 (93.1%)	0	5586 (97.9%)	-1	108 (1.9%)

Clone 018P (+1/+35/+32)

NGS 1, NGS 2 (cell bank)

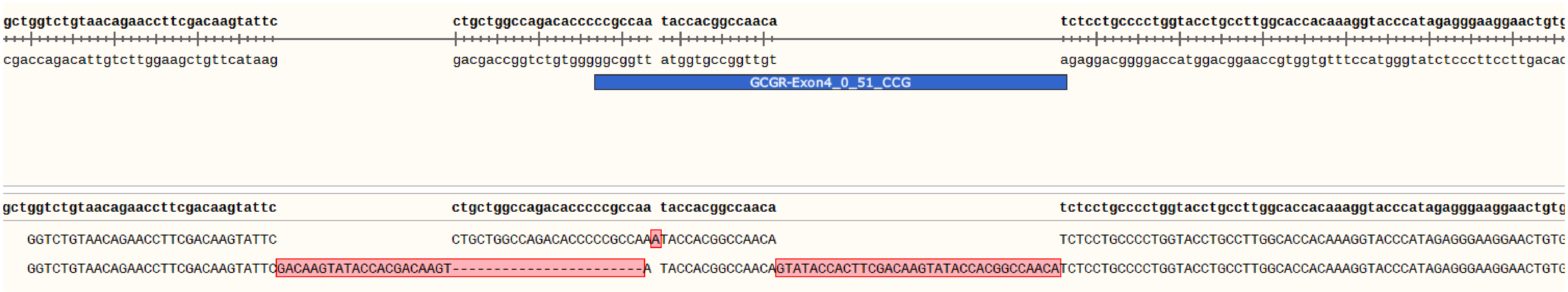

Name	Clone ID	Total # reads	# wt reads(%)	#1-Indel	#1-Reads(%)	#2-Indel	#2-Reads(%)	#3-Indel	#3-Reads(%)
GCGR 1H8	018P	2176	2 (0.1%)	1	840 (38.6%)	35	633 (29.1%)	32	492 (22.6%)

Clone ID	Clone ID	Total # reads	# wt reads(%)	#1-Indel	#1-Reads(%)	#2-Indel	#2-Reads(%)	#3-Indel	#3-Reads(%)
GCGR 1H8	018P	2200	0 (0.0%)	1	836 (38.0%)	35	646 (29.4%)	32	595 (27.0%)

Clone 035P (-1/-10/-11)

No data NGS 1, fragment analysis, topo sequencing, NGS 2 (cell bank)

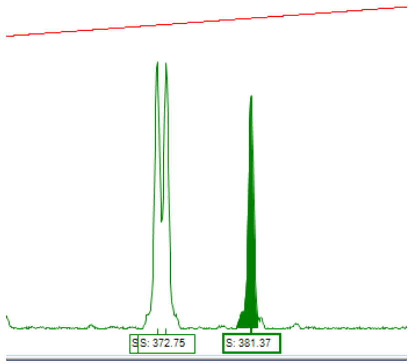

Topo sequencing -10/-11/-1

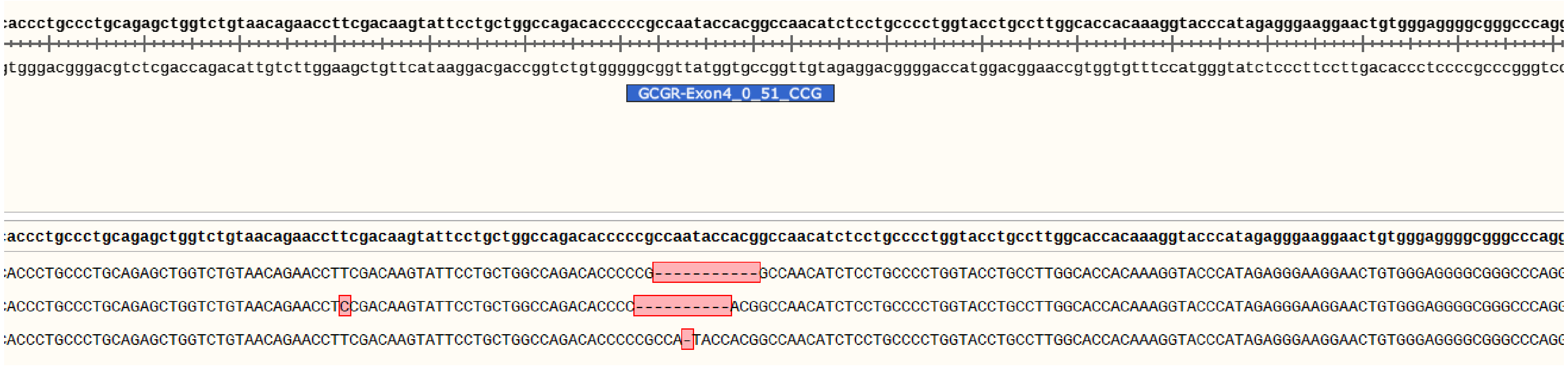

Clone ID	Clone ID	Total # reads	# wt reads(%)	#1-Indel	#1-Reads(%)	#2-Indel	#2-Reads(%)	#3-Indel	#3-Reads(%)
GCGR	035P	3431	0 (0.0%)	-11	1214 (35.4%)	-10	1131 (33.0%)	-1	965 (28.1%)

Clone 053P (-5/-1/-488)

NGS 1, agarose gel PCR, topo sequencing, NGS 2 (cell bank)

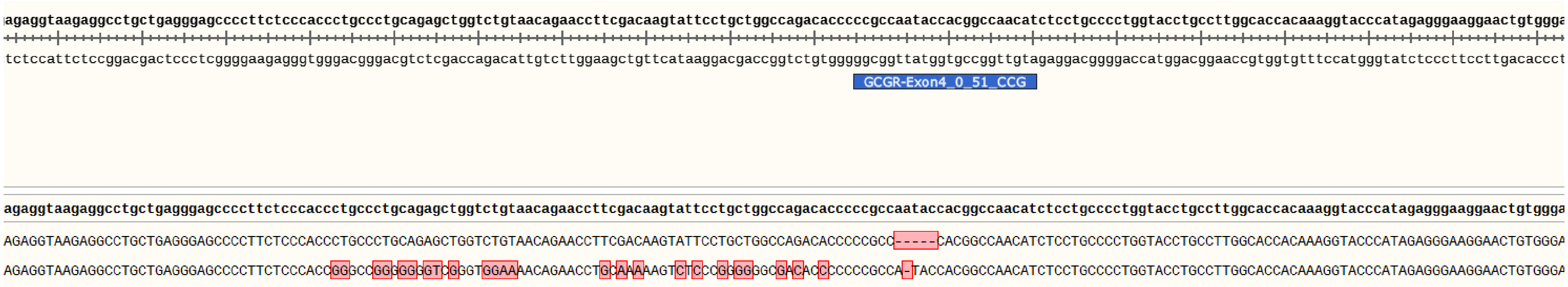

Name	Clone ID	Total # reads	# wt reads(%)	#1-Indel	#1-Reads(%)	#2-Indel	#2-Reads(%)	#3-Indel	#3-Reads(%)
GCGR 2D10	053P	254	0 (0.0%)	-5	122 (48.0%)	-1	69 (27.2%)	0	39 (15.4%)

Clone 053P (-5/-1/-488)

NGS 1, agarose gel PCR, topo sequencing, NGS 2 (cell bank)

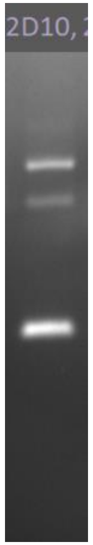

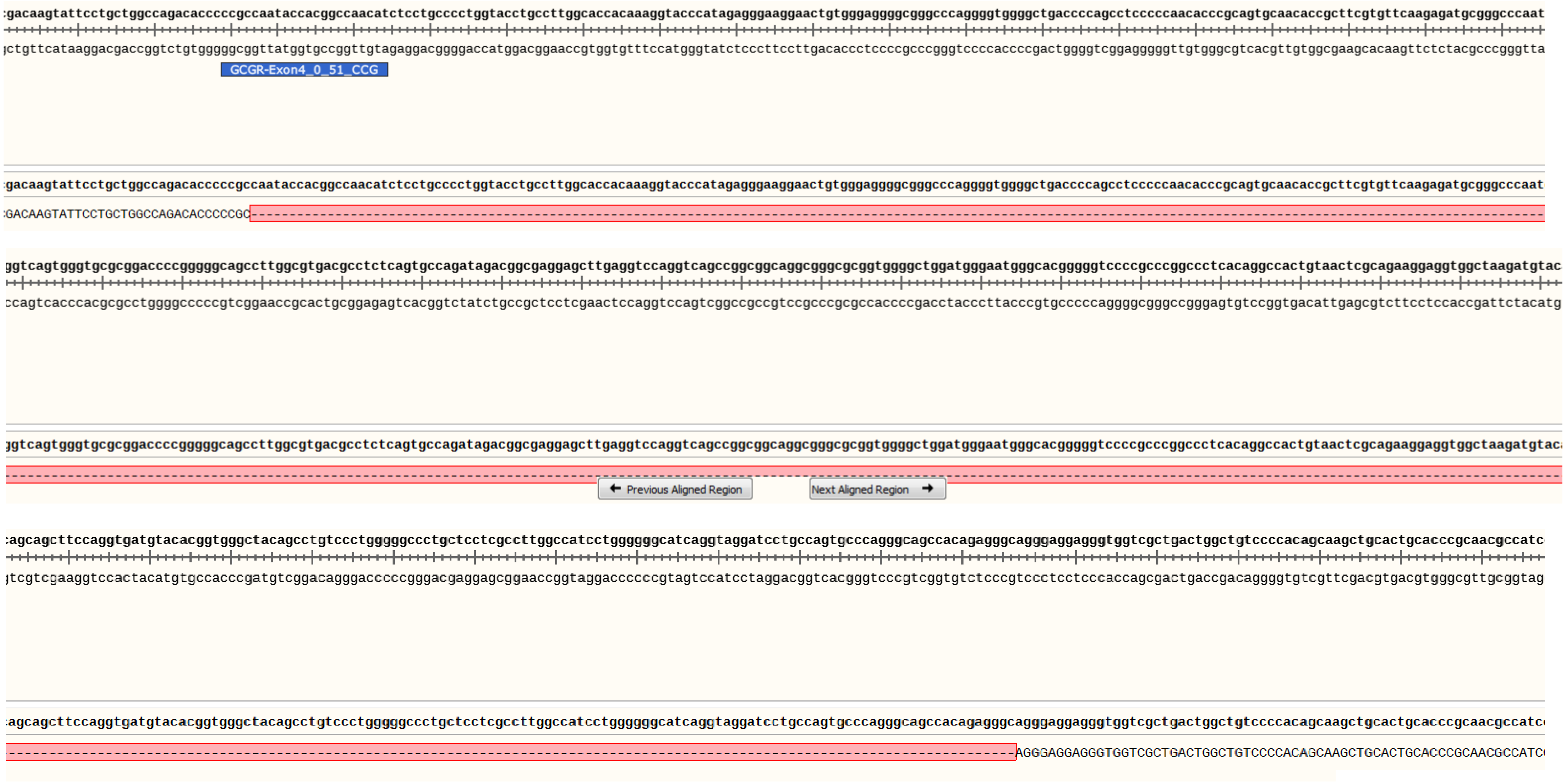

-488bp

Clone 053P (-5/-1/-488)

NGS 1, agarose gel PCR, topo sequencing, NGS 2 (cell bank)

Deleted sequence

caataccacggccaacatctcctgcccctggtacctgccttggcaccacaaaggtacccatagagggaaggaactgtgggaggggcgggcccagg
ggtggggctgaccccagcctcccccaacacccgcagtgcaacaccgcttcgtgttcaagagatgcgggcccaatggtcagtgggtgcgcggacccc
gggggcagccttggcgtgacgcctctcagtgccagatagacggcgaggagcttgaggtccaggtcagccggcggcaggcgggcgcggtggggct
ggatgggaatgggcacgggggtccccgcccggccctcacaggccactgtaactcgcagaaggaggtggctaagatgtacagcagcttccaggtg
atgtacacggtgggctacagcctgtccctgggggccctgctcctcgccttggccatcctgggggcatcaggtaggatcctgccagtgcccagggca
gccacagagggc

Clone ID	Clone ID	Total # reads	# wt reads(%)	#1-Indel	#1-Reads(%)	#2-Indel	#2-Reads(%)	#3-Indel	#3-Reads(%)
GCGR 2D10	053P	4173	0 (0.0%)	-5	2162 (51.8%)	-1	1760 (42.2%)	-6	126 (3.0%)

Clone 056P (-16/-2/-341)

NGS 1, agarose gel PCR, topo sequencing, NGS 2 (cell bank)

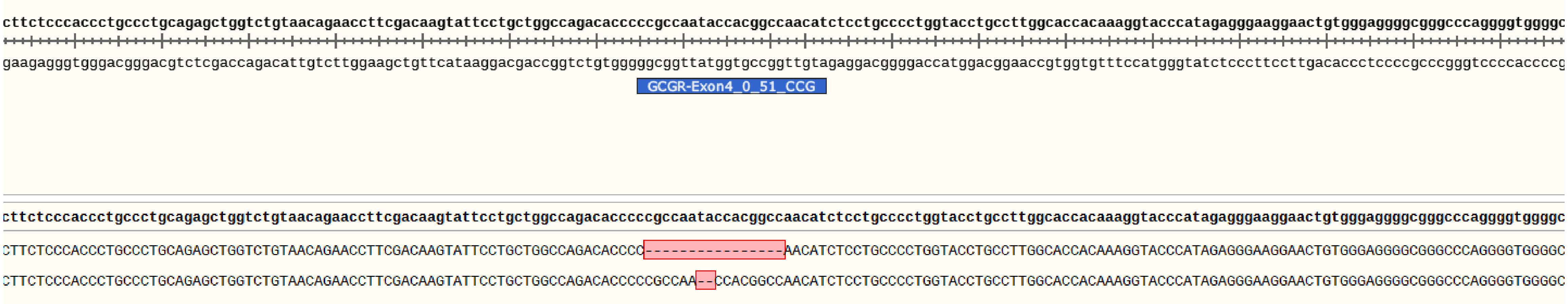

Name	Clone ID	Total # reads	# wt reads(%)	#1-Indel	#1-Reads(%)	#2-Indel	#2-Reads(%)	#3-Indel	#3-Reads(%)
GCGR 3B10	056P	336	0 (0.0%)	-16	177 (52.7%)	-2	139 (41.4%)	-17	12 (3.6%)

Clone 056P (-16/-2/-341)

NGS 1, agarose gel PCR, topo sequencing, NGS 2 (cell bank)

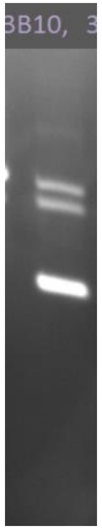

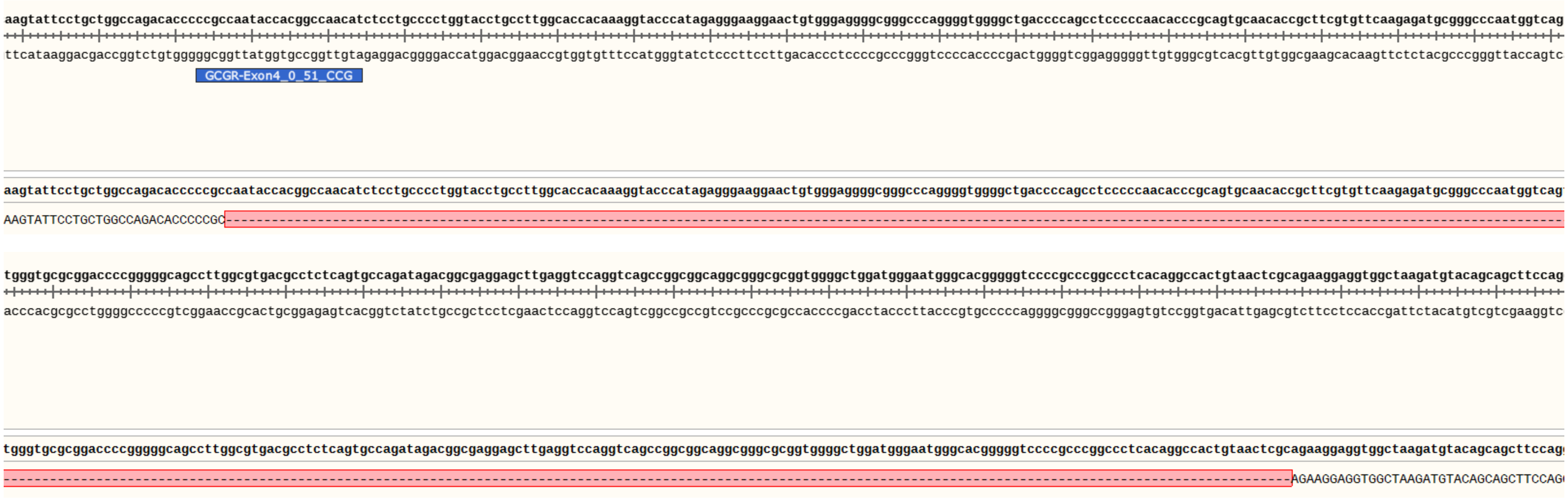

-341bp

Deleted sequence

caataccacggccaacatctcctgcccctggtacctgccttggcaccacaaaggtacccatagagggaaggaactgtgggaggggcgggcccaggggtg
gggctgaccccagcctcccccaacacccgcagtgcaacaccgcttcgtgttcaagagatgcgggcccaatggtcagtgggtgcgcggaccccgggggca
gccttggcgtgacgcctctcagtgccagatagacggcgaggagcttgaggtccaggtcagccggcggcaggcgggcgcggtggggctggatgggaatg
ggcacgggggtccccgcccggccctcacaggccactgtaactcgc

Clone ID	Clone ID	Total # reads	# wt reads(%)	#1-Indel	#1-Reads(%)	#2-Indel	#2-Reads(%)	#3-Indel	#3-Reads(%)
GCGR 3B10	056P	2875	0 (0.0%)	-16	1428 (49.7%)	-2	1288 (44.8%)	-17	74 (2.6%)

Clone 057P (+1/+4/-467)

NGS 1, agarose gel PCR, topo sequencing, NGS 2 (cell bank)

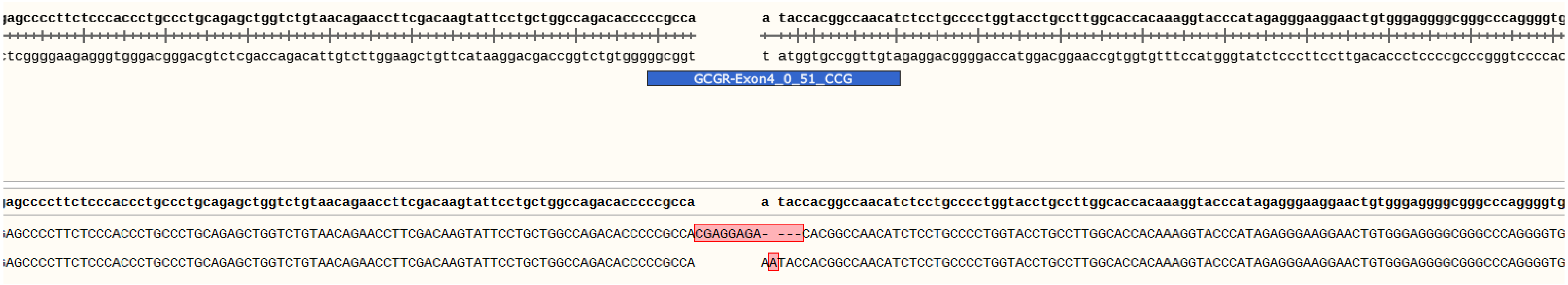

Name	Clone ID	Total # reads	# wt reads(%)	#1-Indel	#1-Reads(%)	#2-Indel	#2-Reads(%)	#3-Indel	#3-Reads(%)
GCGR 4F1	057P	1897	0 (0.0%)	4	979 (51.6%)	1	858 (45.2%)	3	25 (1.3%)

Clone 057P (+1/+4/-467)

NGS 1, agarose gel PCR, topo sequencing, NGS 2 (cell bank)

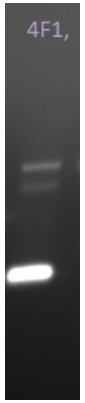

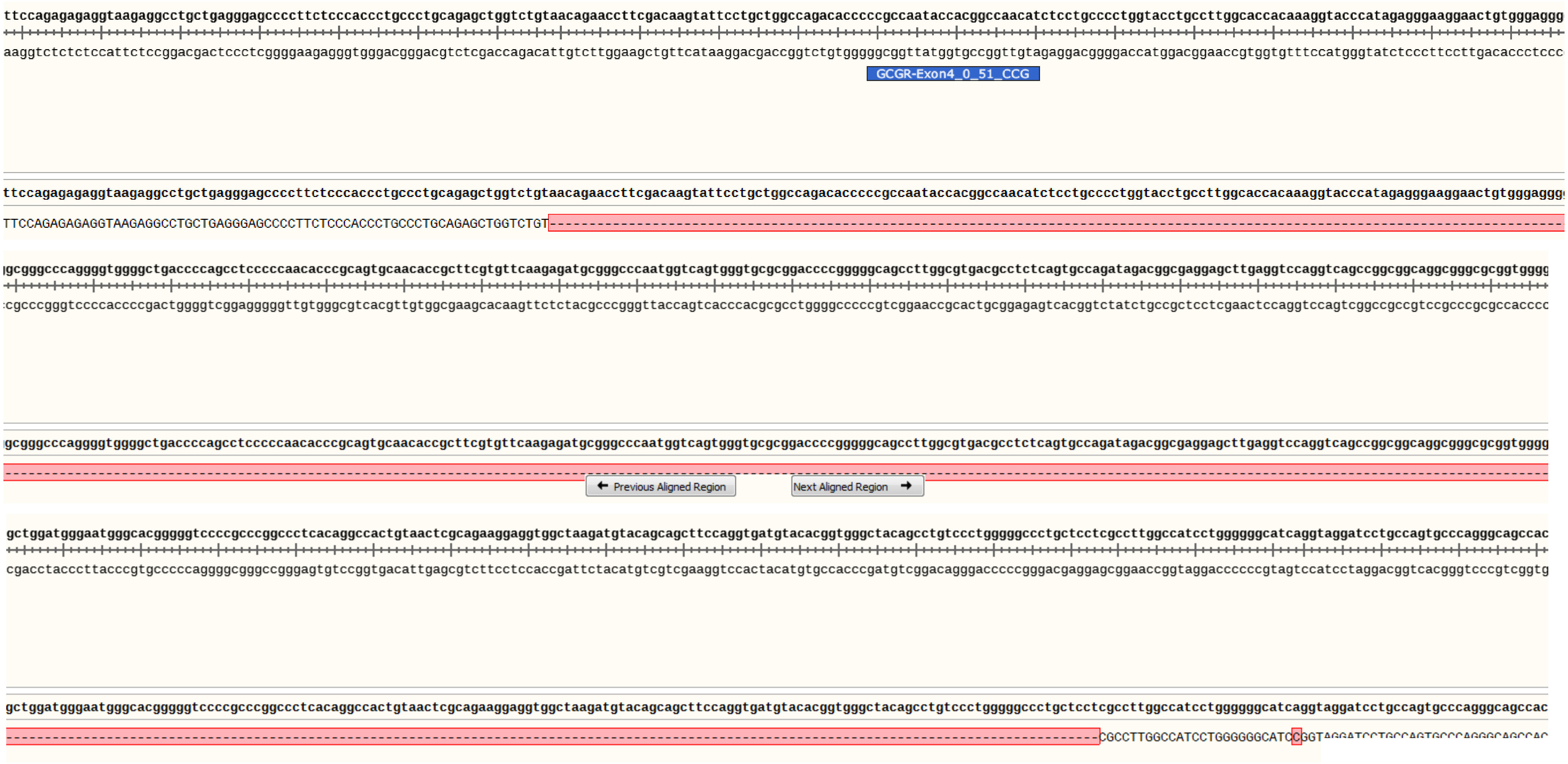

-467bp

Clone 057P (+1/+4/-467)

NGS 1, agarose gel PCR, topo sequencing, NGS 2 (cell bank)

Deleted sequence

aacagaaccttcgacaagtattcctgctggccagacacccccgccaataccacggccaacatctcctgcccctggtacctgccttggcac
cacaaaggtacccatagagggaaggaactgtgggagggcgggcccaggggtggggctgaccccagcctcccccaacacccgcag
tgcaacaccgcttcgtgttcaagagatgcgggcccaatggtcagtgggtgcgcggaccccgggggcagccttggcgtgacgcctctca
gtgccagatagacggcgaggagcttgaggtccaggtcagccggcggcaggcgggcgcggtggggctggatgggaatgggcacgg
gggtccccgcccggccctcacaggccactgtaactcgcagaaggaggtggctaagatgtacagcagcttccaggtgatgtacacggtg
ggctacagcctgtccctgggggccctgctcct

Clone ID	Clone ID	Total # reads	# wt reads(%)	#1-Indel	#1-Reads(%)	#2-Indel	#2-Reads(%)	#3-Indel	#3-Reads(%)
GCGR 4F1	057P	2313	1 (0.0%)	1	1172 (50.7%)	4	991 (42.8%)	3	61 (2.6%)

Clone 059P (+4/-11/+1)

NGS 1, NGS 2 (cell bank)

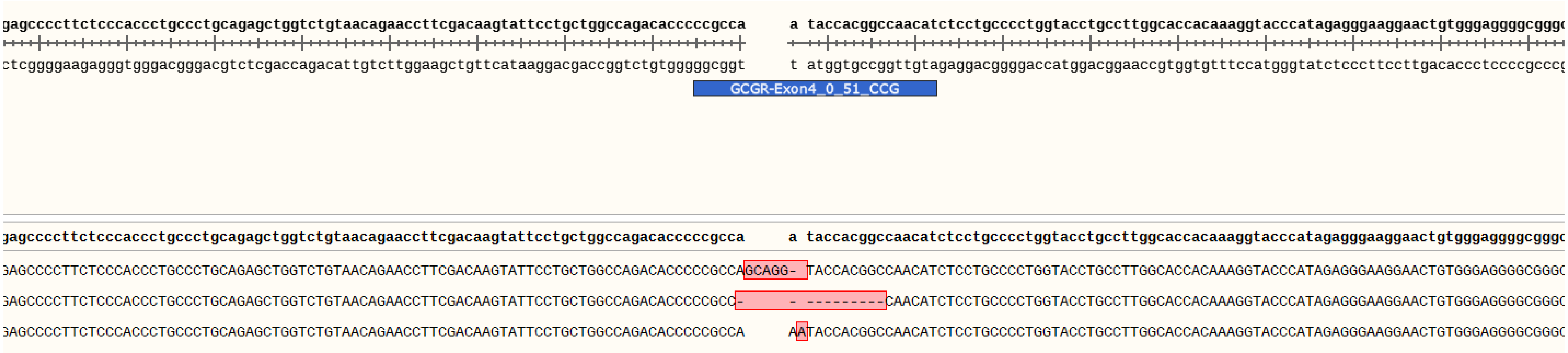

Name	Clone ID	Total # reads	# wt reads(%)	#1-Indel	#1-Reads(%)	#2-Indel	#2-Reads(%)	#3-Indel	#3-Reads(%)
GCGR 4D12	059P	2285	1 (0.0%)	4	845 (37.0%)	-11	682 (29.8%)	1	627 (27.4%)

Clone ID	Clone ID	Total # reads	# wt reads(%)	#1-Indel	#1-Reads(%)	#2-Indel	#2-Reads(%)	#3-Indel	#3-Reads(%)
GCGR 4D12	059P	3139	0 (0.0%)	4	1033 (32.9%)	-11	1013 (32.3%)	1	886 (28.2%)

Clone 060P (-1/-1/-17)

NGS 1, NGS 2 (cell bank)

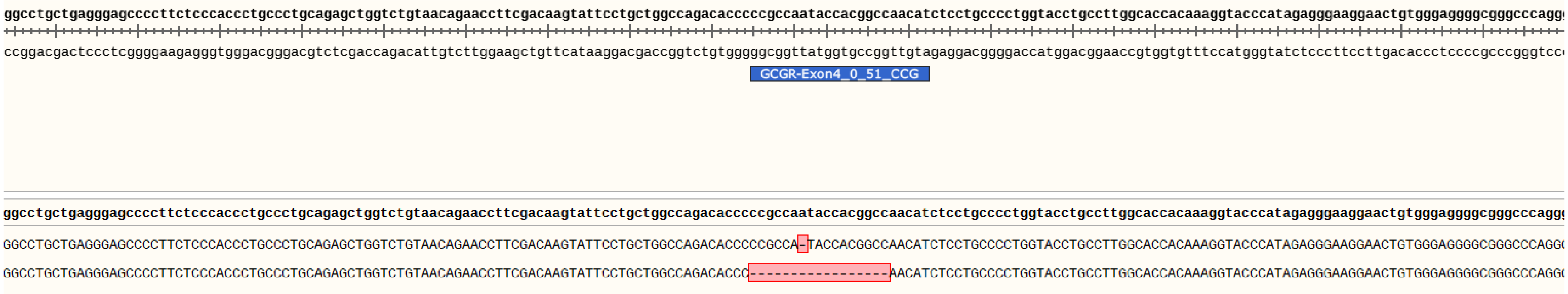

Name	Clone ID	Total # reads	# wt reads(%)	#1-Indel	#1-Reads(%)	#2-Indel	#2-Reads(%)	#3-Indel	#3-Reads(%)
GCGR 4G7	060P	2457	3 (0.1%)	-1	1398 (56.9%)	-17	902 (36.7%)	-2	75 (3.1%)

Clone ID	Clone ID	Total # reads	# wt reads(%)	#1-Indel	#1-Reads(%)	#2-Indel	#2-Reads(%)	#3-Indel	#3-Reads(%)
GCGR 4G7	060P	2595	0 (0.0%)	-1	1525 (58.8%)	-17	930 (35.8%)	-2	74 (2.9%)

Clone 061P (-11/-16/-1)

NGS 1, NGS 2 (cell bank)

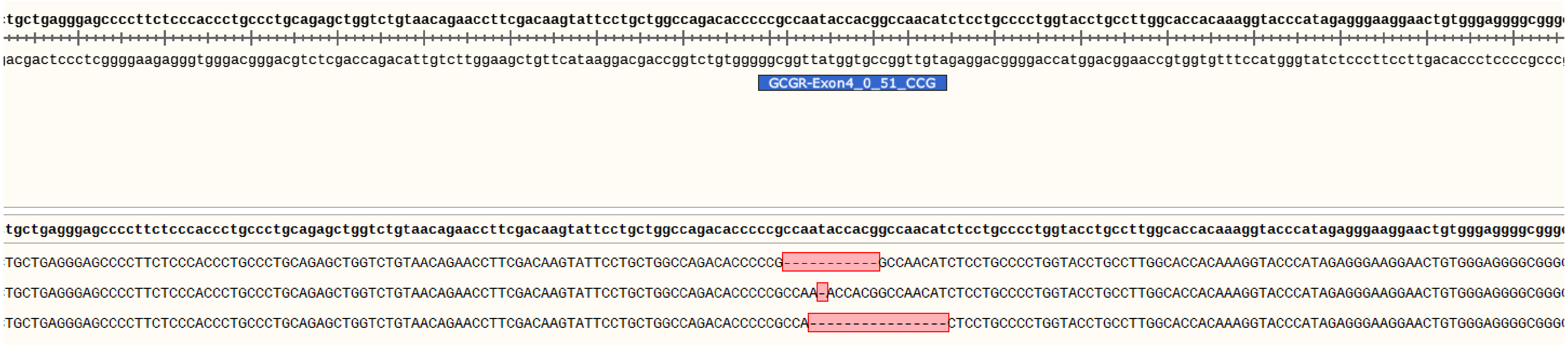

Name	Clone ID	Total # reads	# wt reads(%)	#1-Indel	#1-Reads(%)	#2-Indel	#2-Reads(%)	#3-Indel	#3-Reads(%)
GCGR 5E7	061P	3378	0 (0.0%)	-11	1248 (36.9%)	-16	1046 (31.0%)	-1	906 (26.8%)

Clone ID	Clone ID	Total # reads	# wt reads(%)	#1-Indel	#1-Reads(%)	#2-Indel	#2-Reads(%)	#3-Indel	#3-Reads(%)
GCGR 5E7	061P	3266	0 (0.0%)	-11	1111 (34.0%)	-16	1024 (31.4%)	-1	915 (28.0%)

RV single knockout clones

COQ9 single knockout clones

Clone ID	In/dels
001R	-23/+1
002R	-4/+1
003R	+1/-2
004R	-7/+1
007R	-8/+1
009R	-25/+1
010R	-19/+1
011R	-14/+1
012R	-23/-11
017R	-38/+1

NAT9 single knockout clones

Clone ID	Indels
022R	-11/-2/+1
023R	-7/-1/-2
024R	-16/-10/-1
025R	-14/-14/-35
027R	-26/-13/-10
029R	-101/-101/-1
030R	-2/+2/-1
032R	+1/+1/-1
035R	-7/+1/-13
039R	-2/-11/+1
040R	-16/-4/+1

NEU2 single knockout clones

Clone ID	Indels
041R	-4/+1
042R	-22/-698
043R	-4/-8
045R	+4/-134
046R	-2/-2
047R	-53/+1
063R	+1/-5
065R	-11/-17
066R	-17/-4

RAD51AP1 single knockout clones

Clone ID	In/dels
052R	-2/-11
054R	-2/-1
055R	-1/+1
056R	-10/+5
057R	-2/+1
058R	-59/+1
059R	-22/-11
060R	+1/+23
061R	-2/-32
068R	+1/-7

SVOPL single knockout clones

Clone ID	In/dels
051R	-23/-23/-5

Clone 001R (-23/+1)

NGS 1, fragment analysis, NGS 2 (cell bank)

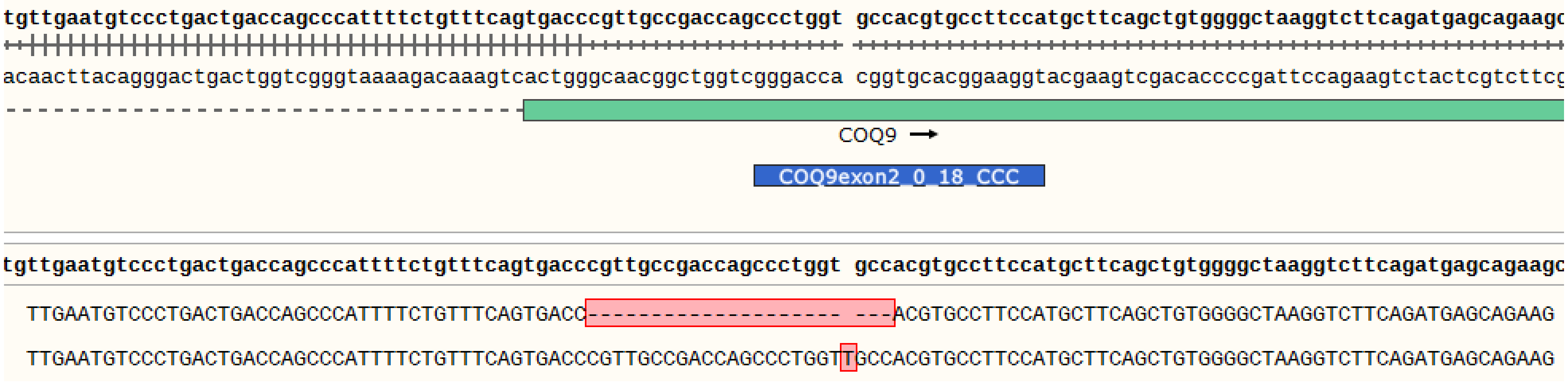

Name	Clone ID	Total # reads	# wt reads(%)	#1-Indel	#1-Reads(%)	#2-Indel	#2-Reads(%)
COQ9 1-B1	001R	2674	0 (0.0%)	-23	1806 (67.5%)	1	827 (30.9%)

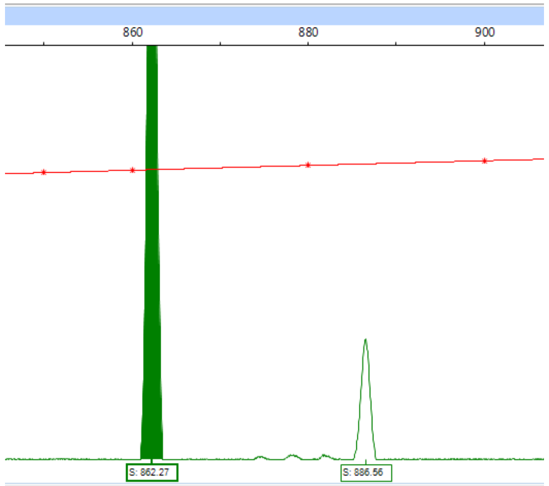

Clone ID	Clone ID	Total # reads	# wt reads(%)	#1-Indel	#1-Reads(%)	#2-Indel	#2-Reads(%)
COQ9 1-B1	001R	2662	0 (0.0%)	-23	2267 (85.2%)	1	302 (11.3%)

Clone 002R (-4/+1)

NGS 1, fragment analysis, NGS 2 (cell bank)

gttgaatgtccctgactgaccagcccatttctgtttcagtgacccgttgccgaccagccctggt gccacgtgccttccatgcttcagctgtggggctaaggtcttcagatgagcagaagc

caacttacagggactgactggtcgggtaaagacaaagtcactgggcaacggctggtcgggacca cggtgcacggaaggtacgaagtcgacaccccgattccagaagtctactcgtcttcg

COQ9 →

COQ9exon2_0_18_CCC

gttgaatgtccctgactgaccagcccatttctgtttcagtgacccgttgccgaccagccctggt gccacgtgccttccatgcttcagctgtggggctaaggtcttcagatgagcagaagc

TTGAATGTCCCTGACTGACCAGCCCATTTCTGTTTCAGTGACCCGTTGCCGACCAGCCCTGGT ----CGTGCCTTCCATGCTTCAGCTGTGGGGCTAAGGTCTTCAGATGAGCAGAAG

TTGAATGTCCCTGACTGACCAGCCCATTTCTGTTTCAGTGACCCGTTGCCGACCAGCCCTGGTTGCCACGTGCCTTCCATGCTTCAGCTGTGGGGCTAAGGTCTTCAGATGAGCAGAAG

Name	Clone ID	Total # reads	# wt reads(%)	#1-Indel	#1-Reads(%)	#2-Indel	#2-Reads(%)
COQ9 1-B11	002R	1564	0 (0.0%)	-4	834 (53.3%)	1	715 (45.7%)

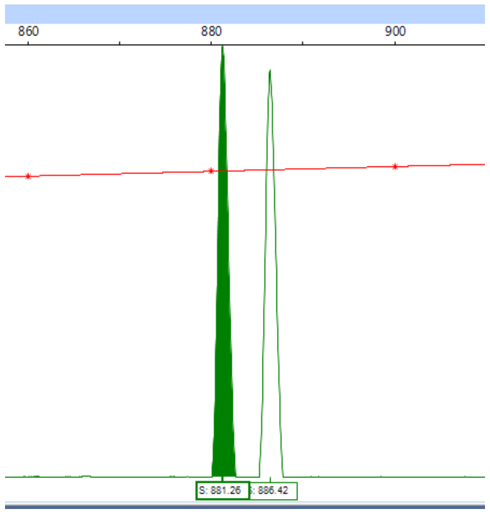

Clone ID	Clone ID	Total # reads	# wt reads(%)	#1-Indel	#1-Reads(%)	#2-Indel	#2-Reads(%)
COQ9 1-B11	002R	3302	0 (0.0%)	-4	1655 (50.1%)	1	1555 (47.1%)

Clone 003R (+1/-2)

NGS 1, fragment analysis, NGS 2 (cell bank)

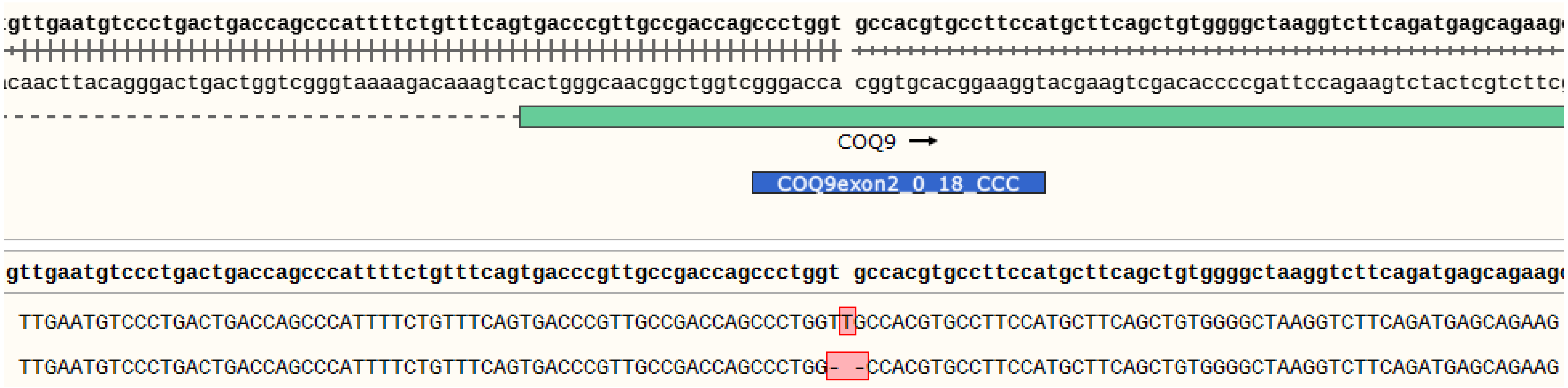

Name	Clone ID	Total # reads	# wt reads(%)	#1-Indel	#1-Reads(%)	#2-Indel	#2-Reads(%)
COQ9 1-B3	003R	2425	0 (0.0%)	1	1215 (50.1%)	-2	1166 (48.1%)

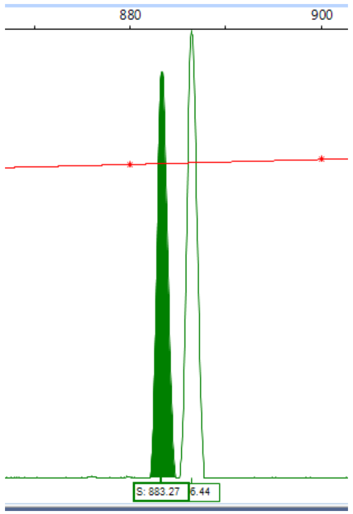

Clone ID	Clone ID	Total # reads	# wt reads(%)	#1-Indel	#1-Reads(%)	#2-Indel	#2-Reads(%)
COQ9 1-B3	003R	2976	0 (0.0%)	-2	1453 (48.8%)	1	1398 (47.0%)

Clone 004R (-7/+1)

NGS 1, fragment analysis, NGS 2 (cell bank)

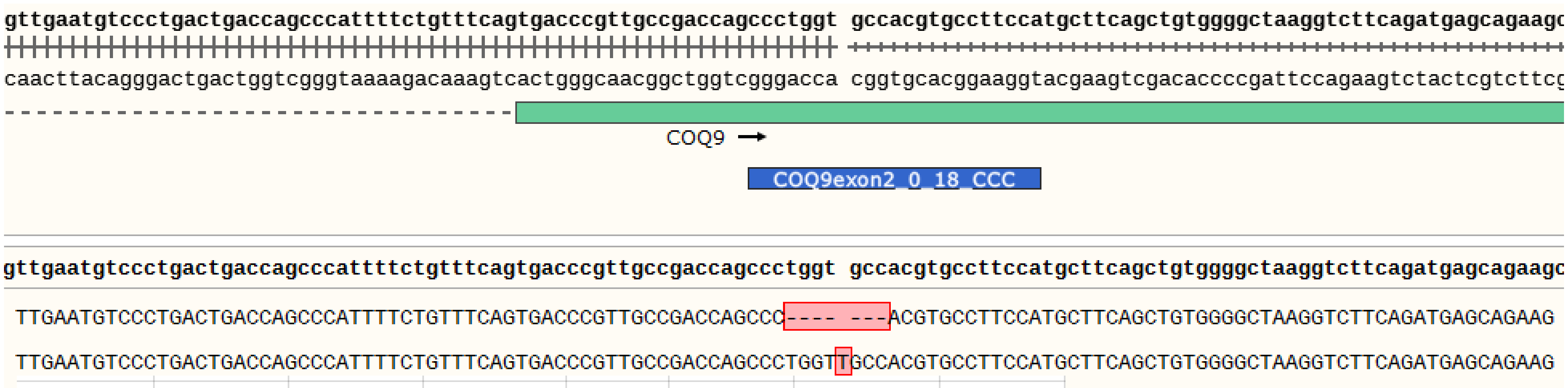

Name	Clone ID	Total # reads	# wt reads(%)	#1-Indel	#1-Reads(%)	#2-Indel	#2-Reads(%)
COQ9 1-B6	004R	2679	1 (0.0%)	-7	1397 (52.1%)	1	1236 (46.1%)

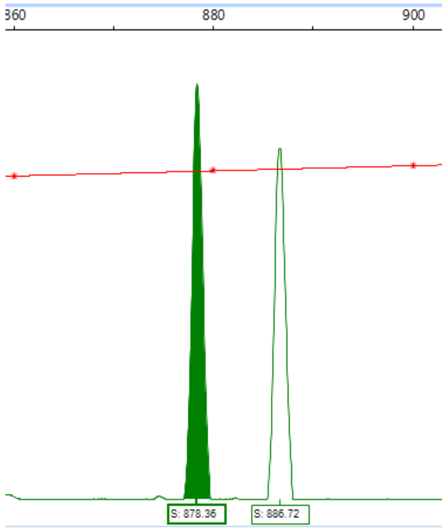

Clone ID	Clone ID	Total # reads	# wt reads(%)	#1-Indel	#1-Reads(%)	#2-Indel	#2-Reads(%)
COQ9 1-B6	004R	2023	0 (0.0%)	-7	1014 (50.1%)	1	923 (45.6%)

Clone 007R (-8/+1)

NGS 1, fragment analysis, NGS 2 (cell bank)

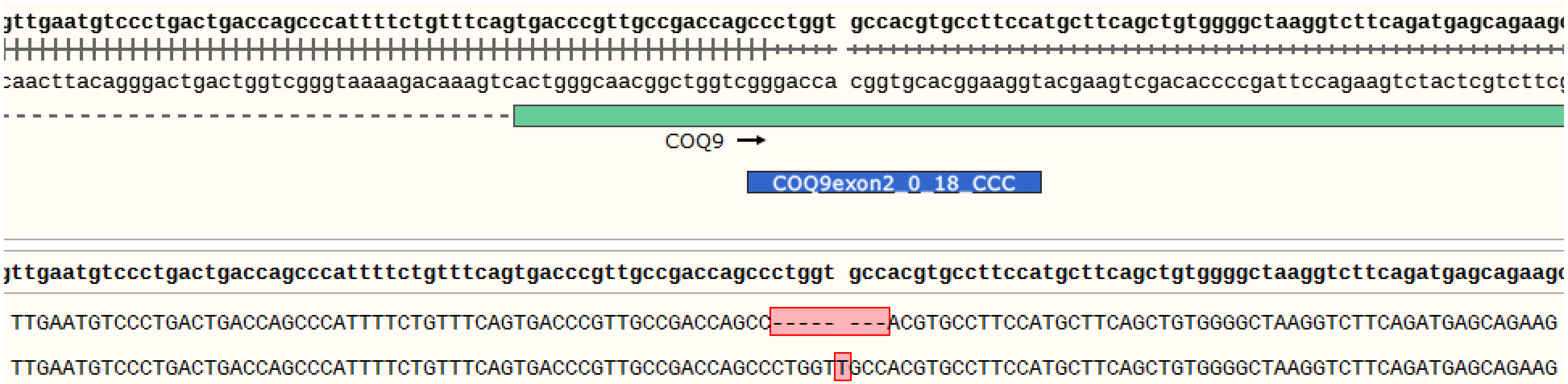

Name	Clone ID	Total # reads	# wt reads(%)	#1-Indel	#1-Reads(%)	#2-Indel	#2-Reads(%)
COQ9 1-D10	007R	1420	0 (0.0%)	-8	830 (58.5%)	1	568 (40.0%)

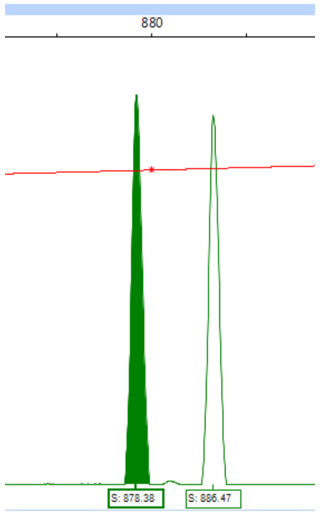

Clone ID	Clone ID	Total # reads	# wt reads(%)	#1-Indel	#1-Reads(%)	#2-Indel	#2-Reads(%)
COQ9 1-D10	007R	2556	0 (0.0%)	-8	1313 (51.4%)	1	1132 (44.3%)

Clone 009R (-25/+1)

NGS 1, fragment analysis, NGS 2 (cell bank)

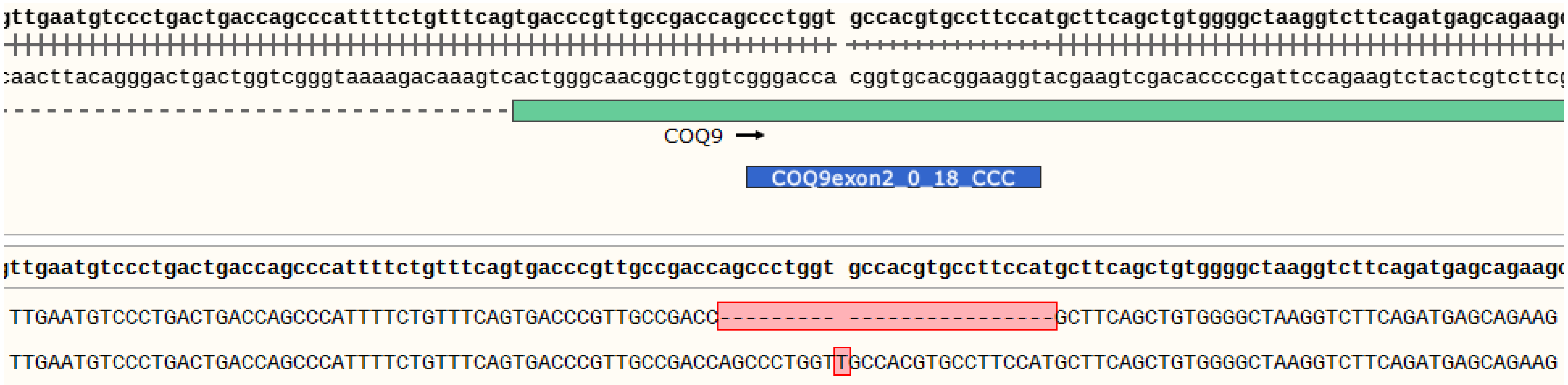

Name	Clone ID	Total # reads	# wt reads(%)	#1-Indel	#1-Reads(%)	#2-Indel	#2-Reads(%)
COQ9 1-E3	009R	1125	0 (0.0%)	-25	539 (47.9%)	1	527 (46.8%)

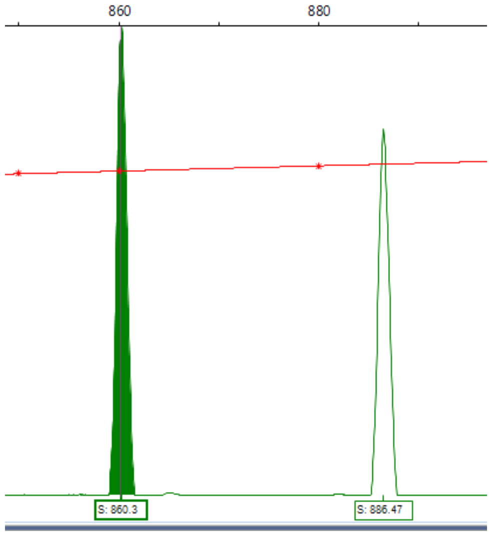

Clone ID	Clone ID	Total # reads	# wt reads(%)	#1-Indel	#1-Reads(%)	#2-Indel	#2-Reads(%)
COQ9 1-E3	009R	2485	0 (0.0%)	-25	1360 (54.7%)	1	1026 (41.3%)

Clone 010R (-19/+1)

NGS 1, fragment analysis, NGS 2 (cell bank)

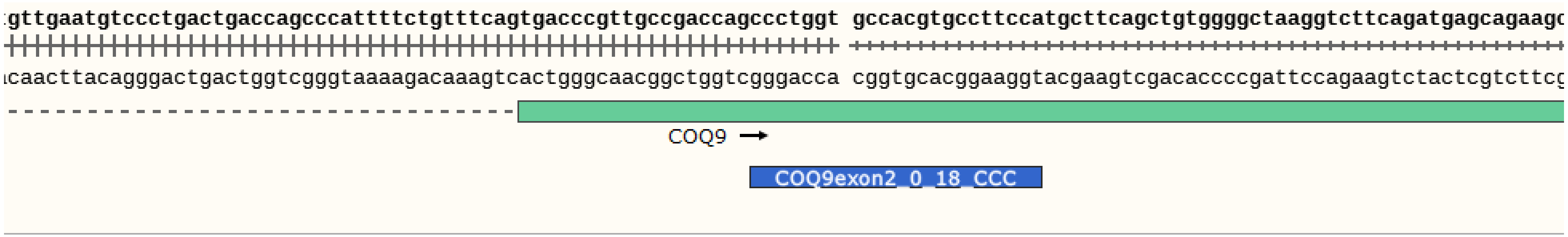


```
gttgaatgtccctgactgaccagcccattttctgtttcagtgacccgttgccgaccagccctggt gccacgtgccttccatgcttcagctgtggggctaaggtcttcagatgagcagaagc

TTGAATGTCCCTGACTGACCAGCCCATTTTCTGTTTCAGTGACCCGTTGCCGACC--------- ----------TTCCATGCTTCAGCTGTGGGGCTAAGGTCTTCAGATGAGCAGAAG

TTGAATGTCCCTGACTGACCAGCCCATTTTCTGTTTCAGTGACCCGTTGCCGACCAGCCCTGGTTGCCACGTGCCTTCCATGCTTCAGCTGTGGGGCTAAGGTCTTCAGATGAGCAGAAG
```

Name	Clone ID	Total # reads	# wt reads(%)	#1-Indel	#1-Reads(%)	#2-Indel	#2-Reads(%)
COQ9 1-E4	010R	2525	0 (0.0%)	-19	1424 (56.4%)	1	1049 (41.5%)

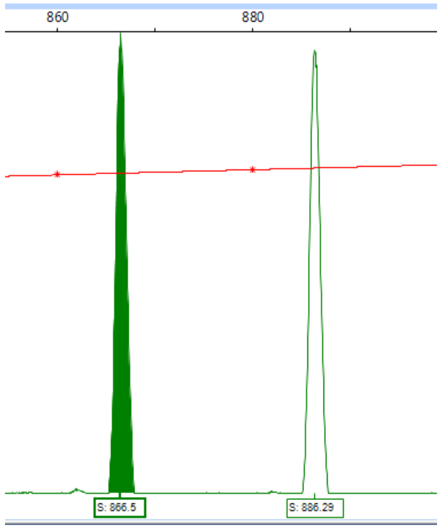

Clone ID	Clone ID	Total # reads	# wt reads(%)	#1-Indel	#1-Reads(%)	#2-Indel	#2-Reads(%)
COQ9 1-E4	010R	2280	0 (0.0%)	-19	1230 (53.9%)	1	950 (41.7%)

Clone 011R (-14/+1)

NGS 1, fragment analysis, NGS 2 (cell bank)

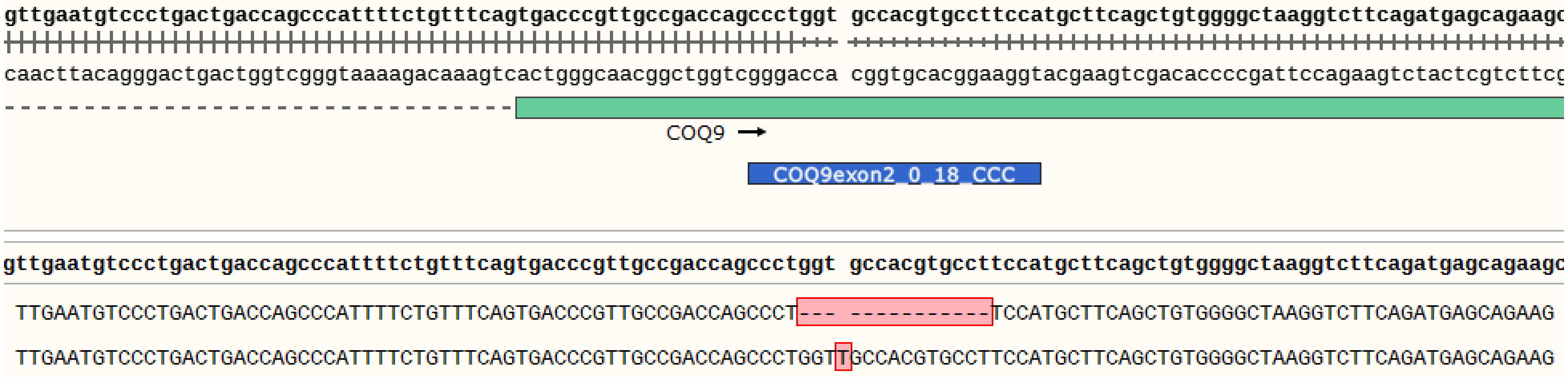

Name	Clone ID	Total # reads	# wt reads(%)	#1-Indel	#1-Reads(%)	#2-Indel	#2-Reads(%)
COQ9 1-E7	011R	2850	1 (0.0%)	-14	1497 (52.5%)	1	1277 (44.8%)

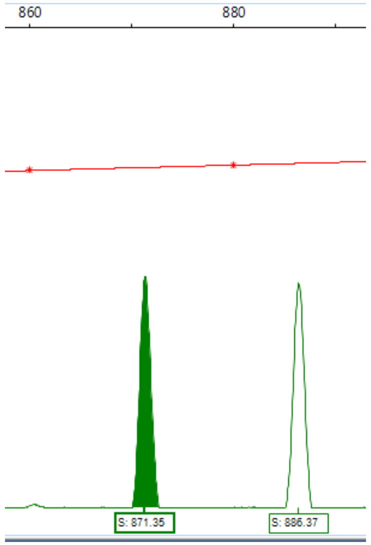

Clone ID	Clone ID	Total # reads	# wt reads(%)	#1-Indel	#1-Reads(%)	#2-Indel	#2-Reads(%)
COQ9 1-E7	011R	2524	0 (0.0%)	-14	1351 (53.5%)	1	1094 (43.3%)

Clone 012R (-23/-11)

NGS 1, fragment analysis, NGS 2 (cell bank)

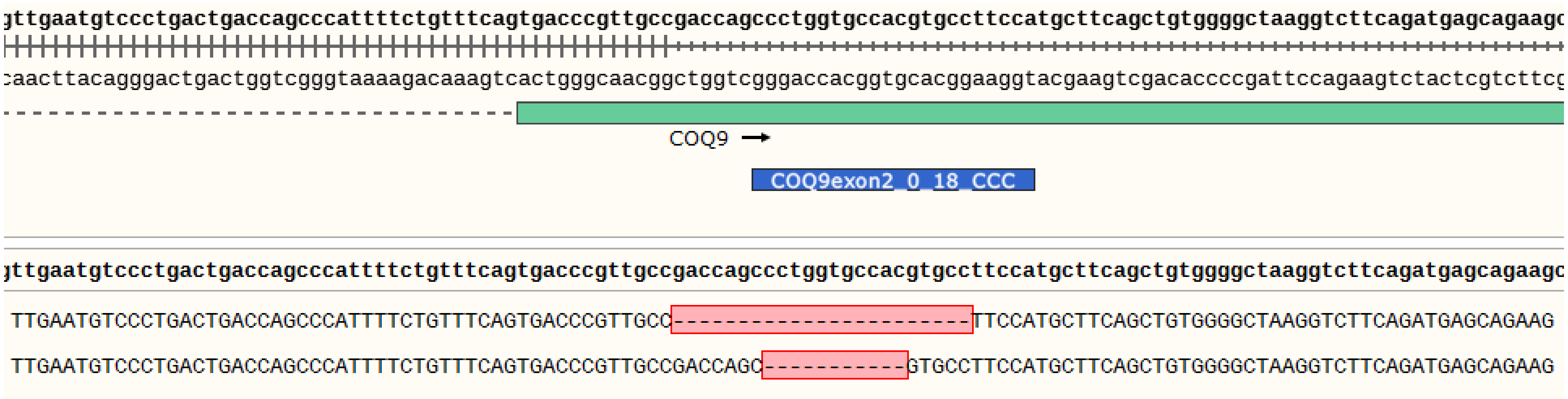

Name	Clone ID	Total # reads	# wt reads(%)	#1-Indel	#1-Reads(%)	#2-Indel	#2-Reads(%)
COQ9 1-F10	012R	2546	0 (0.0%)	-23	1357 (53.3%)	-11	1156 (45.4%)

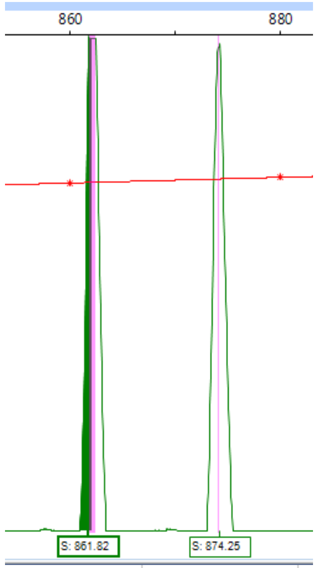

Clone ID	Clone ID	Total # reads	# wt reads(%)	#1-Indel	#1-Reads(%)	#2-Indel	#2-Reads(%)
COQ9 1-F10	012R	2595	0 (0.0%)	-23	1318 (50.8%)	-11	1172 (45.2%)

Clone 017R (-38/+1)

NGS 1, fragment analysis, NGS 2 (cell bank)

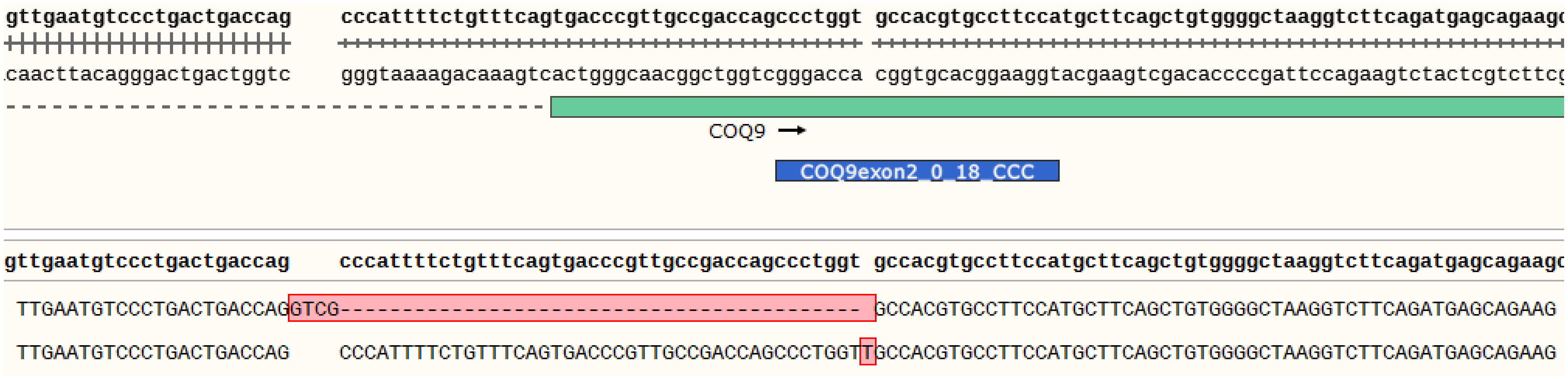

Name	Clone ID	Total # reads	# wt reads(%)	#1-Indel	#1-Reads(%)	#2-Indel	#2-Reads(%)
COQ9 2-C12	017R	1877	0 (0.0%)	-38	1093 (58.2%)	1	762 (40.6%)

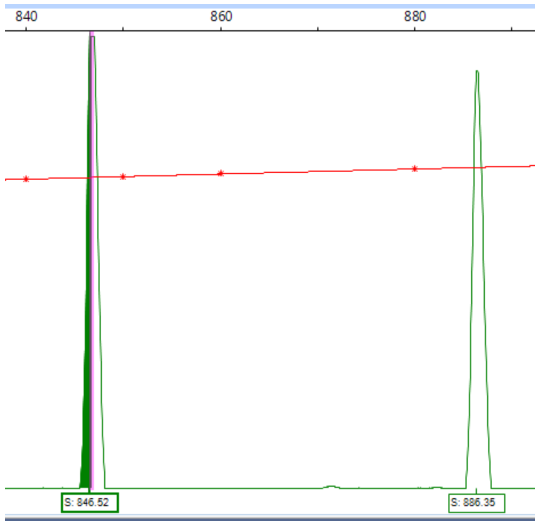

Clone ID	Clone ID	Total # reads	# wt reads(%)	#1-Indel	#1-Reads(%)	#2-Indel	#2-Reads(%)
COQ9 2-C12	017R	3371	0 (0.0%)	-38	1985 (58.9%)	1	1261 (37.4%)

Clone 022R (-11/-2/+1)

NGS 1, NGS 2 (cell bank)

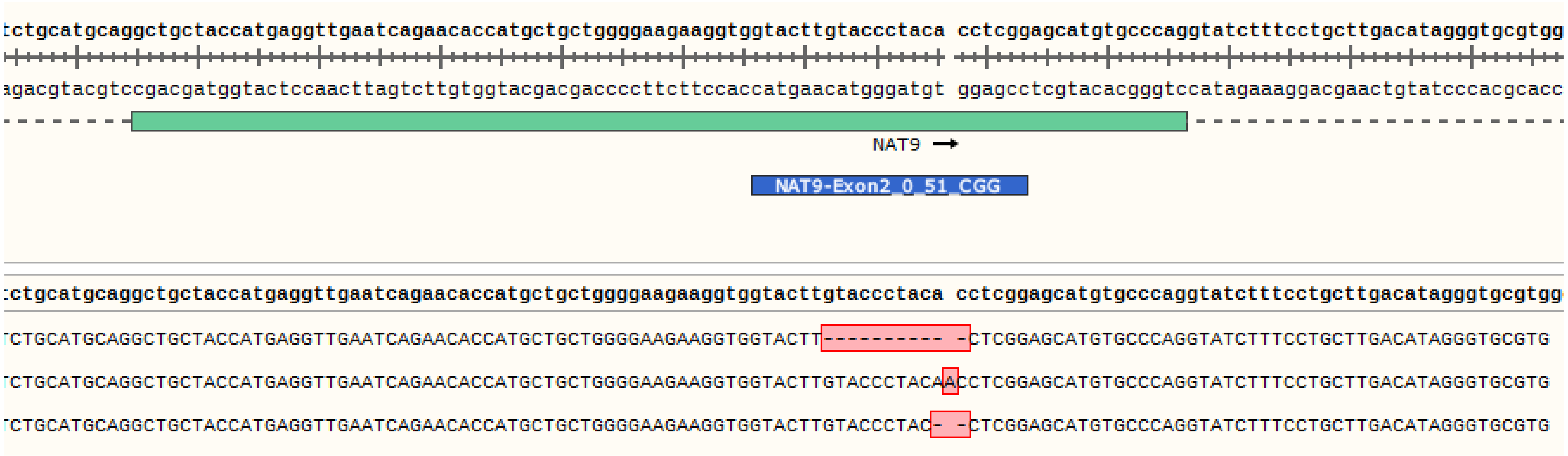

Name	Clone ID	Total # reads	# wt reads(%)	#1-Indel	#1-Reads(%)	#2-Indel	#2-Reads(%)	#3-Indel	#3-Reads(%)
NAT9 1-D11	022R	2193	0 (0.0%)	-11	790 (36.0%)	-2	700 (31.9%)	1	675 (30.8%)

Clone ID	Clone ID	Total # reads	# wt reads(%)	#1-Indel	#1-Reads(%)	#2-Indel	#2-Reads(%)	#3-Indel	#3-Reads(%)
NAT9 1-D11	022R	1421	0 (0.0%)	-2	478 (33.6%)	-11	464 (32.7%)	1	449 (31.6%)

Clone 023R (-7/-1/-2)

NGS 1, NGS 2 (cell bank)

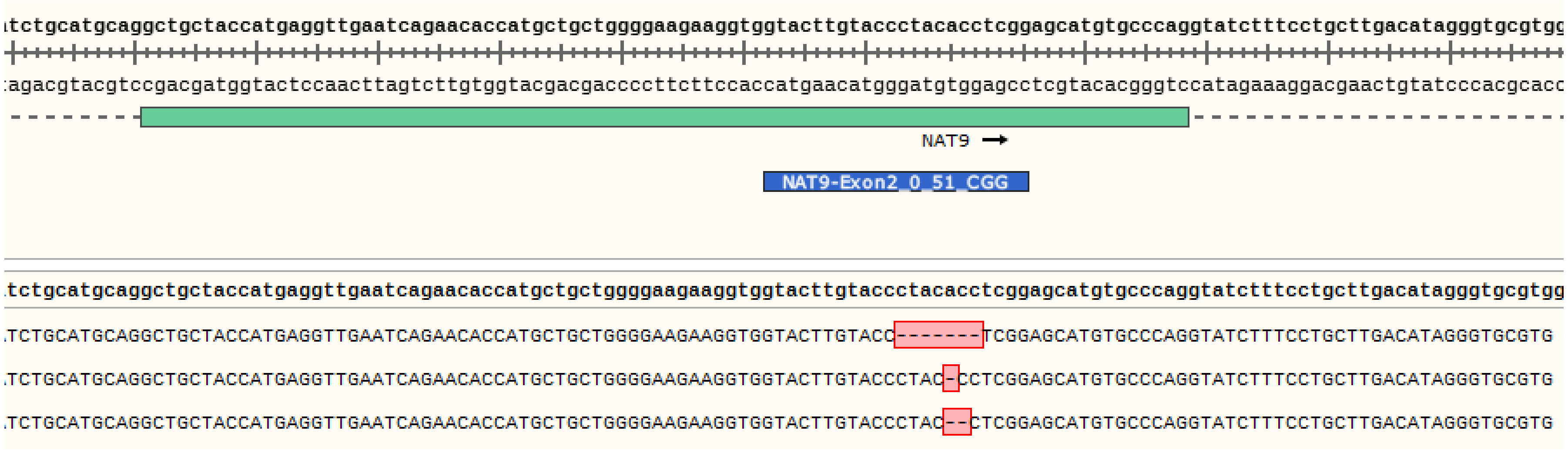

Name	Clone ID	Total # reads	# wt reads(%)	#1-Indel	#1-Reads(%)	#2-Indel	#2-Reads(%)	#3-Indel	#3-Reads(%)
NAT9 1-E10	023R	2126	0 (0.0%)	-7	738 (34.7%)	-1	715 (33.6%)	-2	647 (30.4%)

Clone ID	Clone ID	Total # reads	# wt reads(%)	#1-Indel	#1-Reads(%)	#2-Indel	#2-Reads(%)	#3-Indel	#3-Reads(%)
NAT9 1-E10	023R	1501	0 (0.0%)	-7	509 (33.9%)	-2	496 (33.0%)	-1	472 (31.4%)

Clone 024R (-16/-10/-1)

NGS 1, fragment analysis, NGS 2 (cell bank)

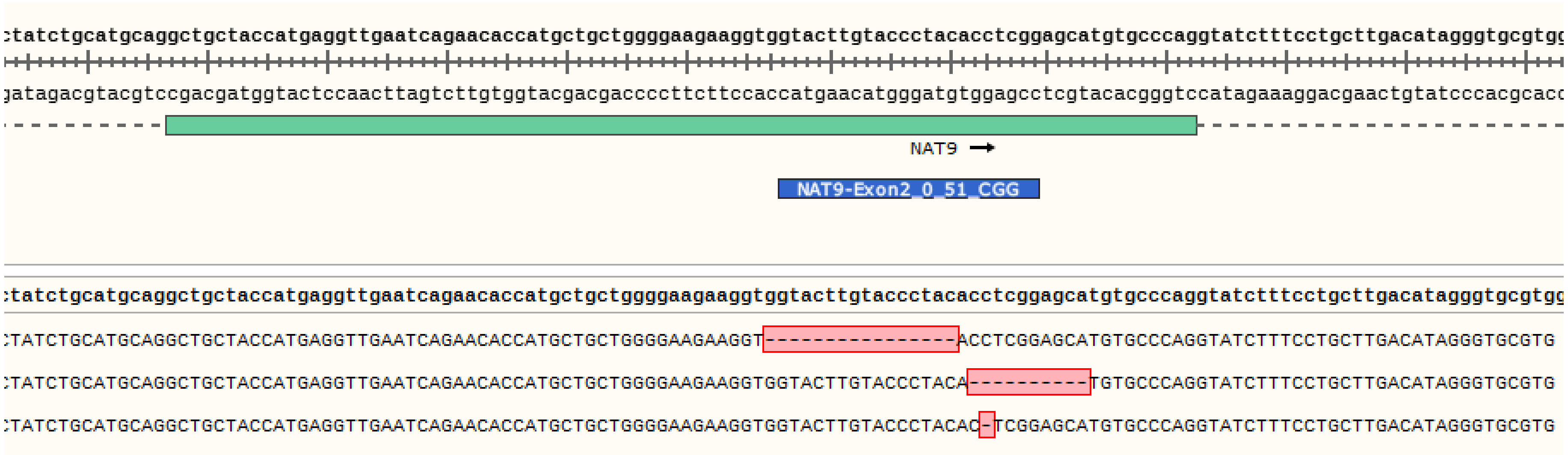

Name	Clone ID	Total # reads	# wt reads(%)	#1-Indel	#1-Reads(%)	#2-Indel	#2-Reads(%)	#3-Indel	#3-Reads(%)
NAT9 1-E2	024R	2738	0 (0.0%)	-16	1010 (36.9%)	-10	862 (31.5%)	-1	828 (30.2%)

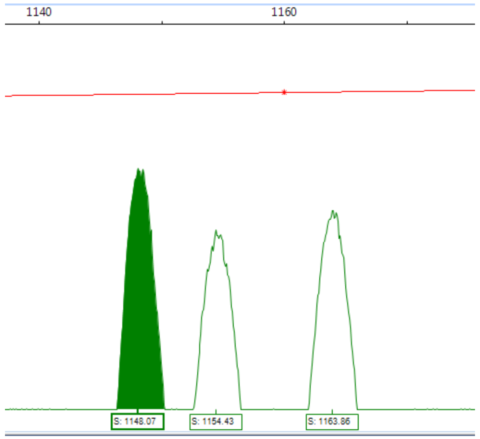

Clone ID	Clone ID	Total # reads	# wt reads(%)	#1-Indel	#1-Reads(%)	#2-Indel	#2-Reads(%)	#3-Indel	#3-Reads(%)
NAT9 1-E2	024R	1472	0 (0.0%)	-16	531 (36.1%)	-10	480 (32.6%)	-1	428 (29.1%)

Clone 025R (-14/-14/-35)

NGS 1, fragment analysis, NGS 2 (cell bank)

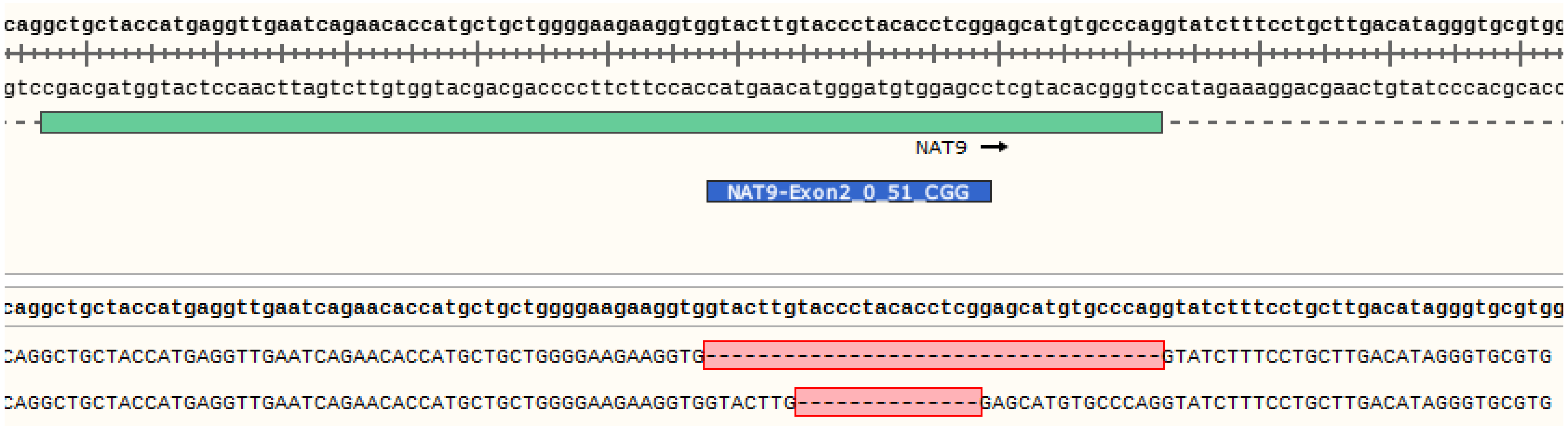

Name	Clone ID	Total # reads	# wt reads(%)	#1-Indel	#1-Reads(%)	#2-Indel	#2-Reads(%)	#3-Indel	#3-Reads(%)
NAT9 1-F2	025R	2599	1 (0.0%)	-14	1495 (57.5%)	-35	1080 (41.6%)	-15	13 (0.5%)

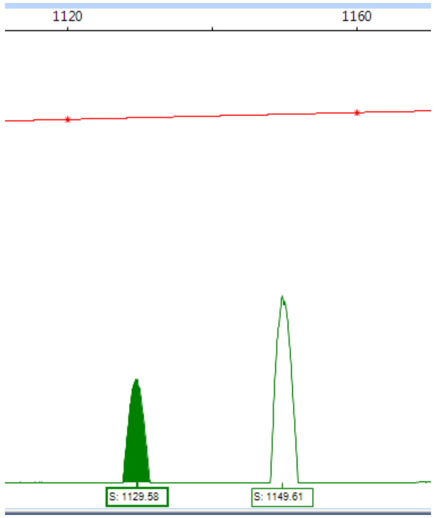

Clone ID	Clone ID	Total # reads	# wt reads(%)	#1-Indel	#1-Reads(%)	#2-Indel	#2-Reads(%)	#3-Indel	#3-Reads(%)
NAT9 1-F2	025R	1694	0 (0.0%)	-14	1005 (59.3%)	-35	648 (38.3%)	-15	27 (1.6%)

Clone 027R (-26/-13/-10)

NGS 1, NGS 2 (cell bank)

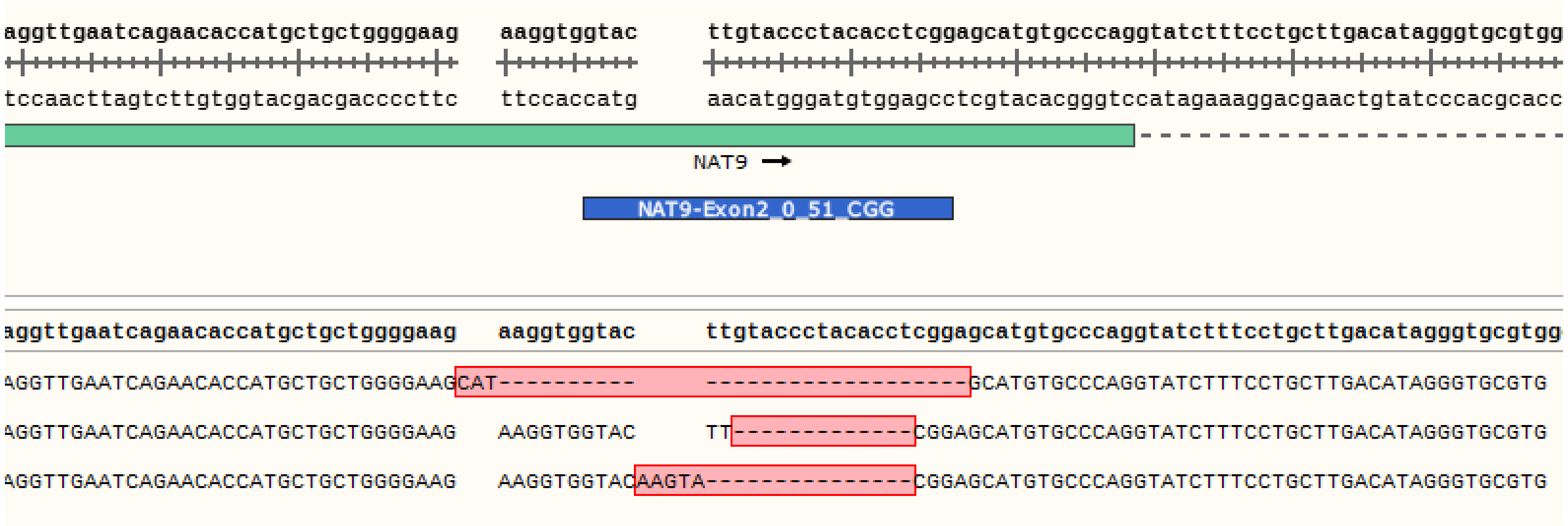

Name	Clone ID	Total # reads	# wt reads(%)	#1-Indel	#1-Reads(%)	#2-Indel	#2-Reads(%)	#3-Indel	#3-Reads(%)
NAT9 1-F8	027R	2698	0 (0.0%)	-26	1071 (39.7%)	-13	847 (31.4%)	-10	740 (27.4%)

Clone ID	Clone ID	Total # reads	# wt reads(%)	#1-Indel	#1-Reads(%)	#2-Indel	#2-Reads(%)	#3-Indel	#3-Reads(%)
NAT9 1-F8	027R	1734	0 (0.0%)	-26	666 (38.4%)	-13	539 (31.1%)	-10	490 (28.3%)

Clone 029R (-101/-101/-1)

NGS 1, NGS 2 (cell bank)

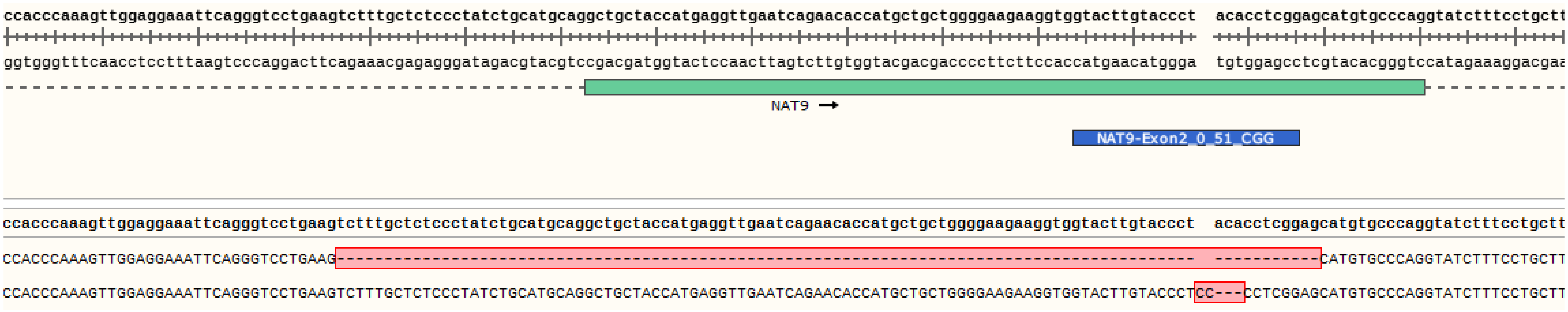

Name	Clone ID	Total # reads	# wt reads(%)	#1-Indel	#1-Reads(%)	#2-Indel	#2-Reads(%)	#3-Indel	#3-Reads(%)
NAT9 1-G6	029R	3242	0 (0.0%)	-101	2308 (71.2%)	-1	910 (28.1%)	-2	11 (0.3%)

Clone ID	Clone ID	Total # reads	# wt reads(%)	#1-Indel	#1-Reads(%)	#2-Indel	#2-Reads(%)	#3-Indel	#3-Reads(%)
NAT9 1-G6	029R	2235	0 (0.0%)	-101	1590 (71.1%)	-1	599 (26.8%)	-102	32 (1.4%)

Clone 030R (-2/+2/-1)

NGS 1, NGS 2 (cell bank)

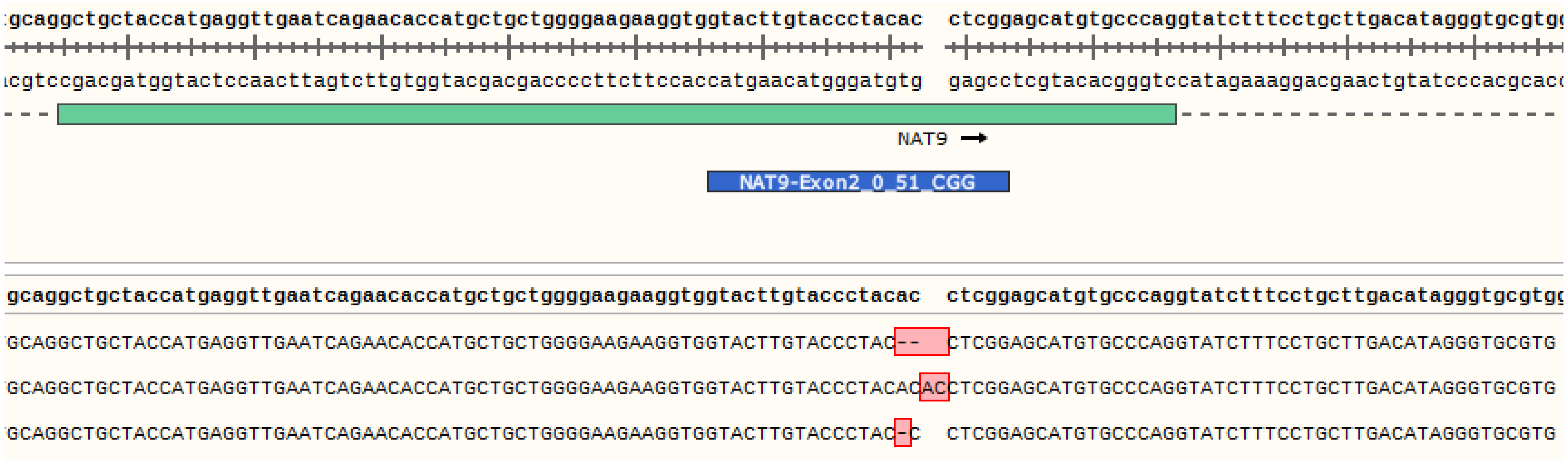

Name	Clone ID	Total # reads	# wt reads(%)	#1-Indel	#1-Reads(%)	#2-Indel	#2-Reads(%)	#3-Indel	#3-Reads(%)
NAT9 1-G9	030R	1988	0 (0.0%)	-2	722 (36.3%)	2	631 (31.7%)	-1	619 (31.1%)

Clone ID	Clone ID	Total # reads	# wt reads(%)	#1-Indel	#1-Reads(%)	#2-Indel	#2-Reads(%)	#3-Indel	#3-Reads(%)
NAT9 1-G9	030R	1303	4 (0.3%)	-2	470 (36.1%)	-1	430 (33.0%)	2	374 (28.7%)

Clone 032R (+1/+1/-1)

NGS 1, NGS 2 (cell bank)

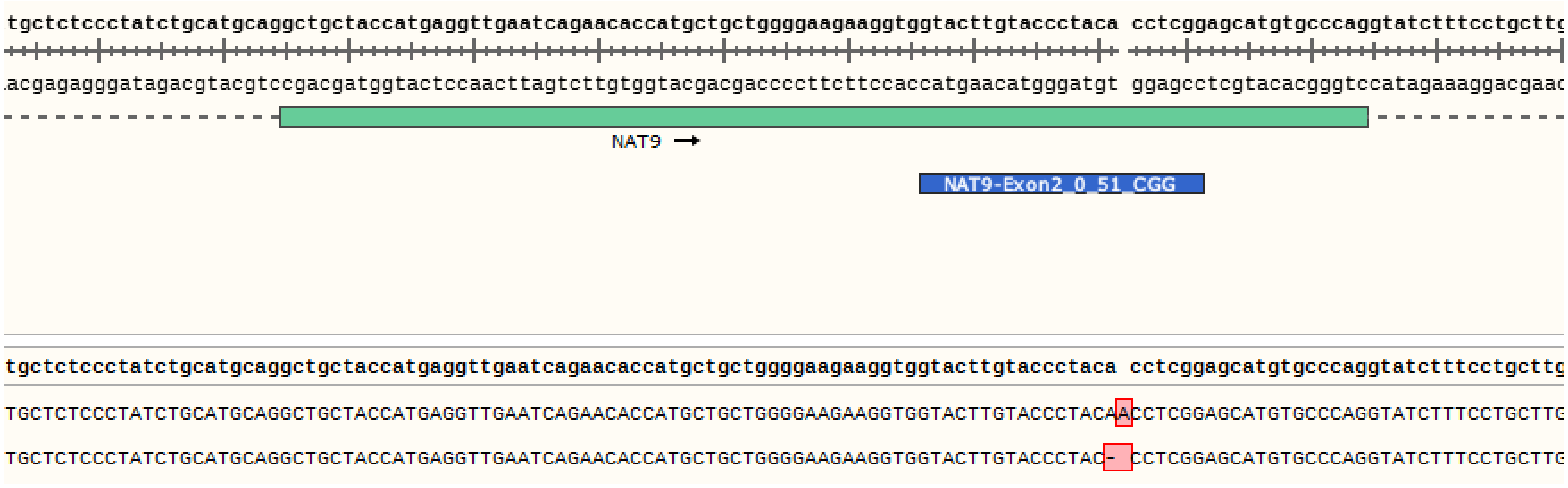

Name	Clone ID	Total # reads	# wt reads(%)	#1-Indel	#1-Reads(%)	#2-Indel	#2-Reads(%)	#3-Indel	#3-Reads(%)
NAT9 1-H5	032R	2638	0 (0.0%)	1	1609 (61.0%)	-1	995 (37.7%)	0	19 (0.7%)

Clone ID	Clone ID	Total # reads	# wt reads(%)	#1-Indel	#1-Reads(%)	#2-Indel	#2-Reads(%)	#3-Indel	#3-Reads(%)
NAT9 1-H5	032R	1669	0 (0.0%)	1	1079 (64.6%)	-1	554 (33.2%)	0	19 (1.1%)

Clone 035R (-7/+1/-13)

NGS 1, fragment analysis, NGS 2 (cell bank)

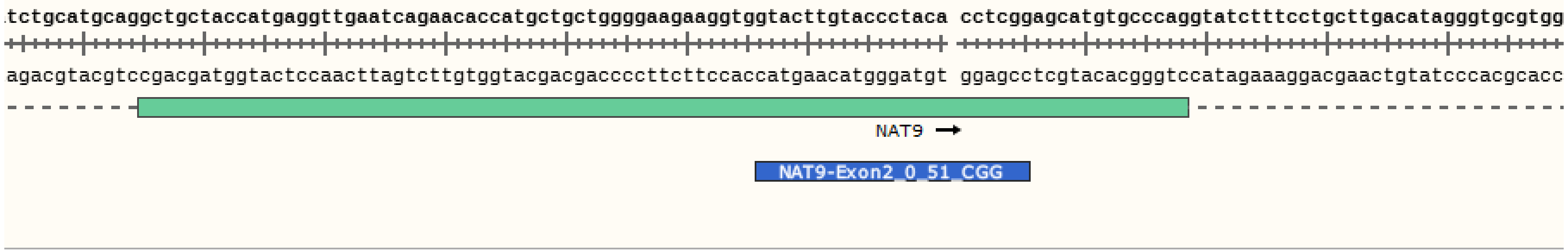


```
tctgcatgcaggctgctaccatgaggttgaatcagaacaccatgctgctggggaagaaggtggtacttgtaccctaca cctcggagcatgtgcccaggtatctttcctgcttgacatagggtgcgtgg
TCTGCATGCAGGCTGCTACCATGAGGTTGAATCAGAACACCATGCTGCTGGGGAAGAAGGTGGTACTTGTACC----- --TCGGAGCATGTGCCCAGGTATCTTTCCTGCTTGACATAGGGTGCGTG
TCTGCATGCAGGCTGCTACCATGAGGTTGAATCAGAACACCATGCTGCTGGGGAAGAAGGTGGTACTTGTACCCTACAACCTCGGAGCATGTGCCCAGGTATCTTTCCTGCTTGACATAGGGTGCGTG
TCTGCATGCAGGCTGCTACCATGAGGTTGAATCAGAACACCATGCTGCTGGGGAAGAAGGTGGTAC------------ -CTCGGAGCATGTGCCCAGGTATCTTTCCTGCTTGACATAGGGTGCGTG
```

Name	Clone ID	Total # reads	# wt reads(%)	#1-Indel	#1-Reads(%)	#2-Indel	#2-Reads(%)	#3-Indel	#3-Reads(%)
NAT9 2-B8	035R	582	0 (0.0%)	-7	227 (39.0%)	1	183 (31.4%)	-13	162 (27.8%)

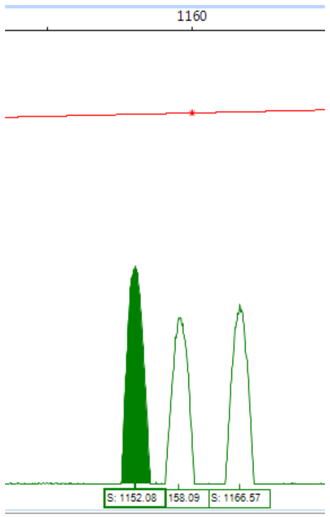

Clone ID	Clone ID	Total # reads	# wt reads(%)	#1-Indel	#1-Reads(%)	#2-Indel	#2-Reads(%)	#3-Indel	#3-Reads(%)
NAT9 2-B8	035R	1215	0 (0.0%)	-13	437 (36.0%)	-7	372 (30.6%)	1	371 (30.5%)

Clone 039R (-2/-11/+1)

NGS 1, NGS 2 (cell bank)

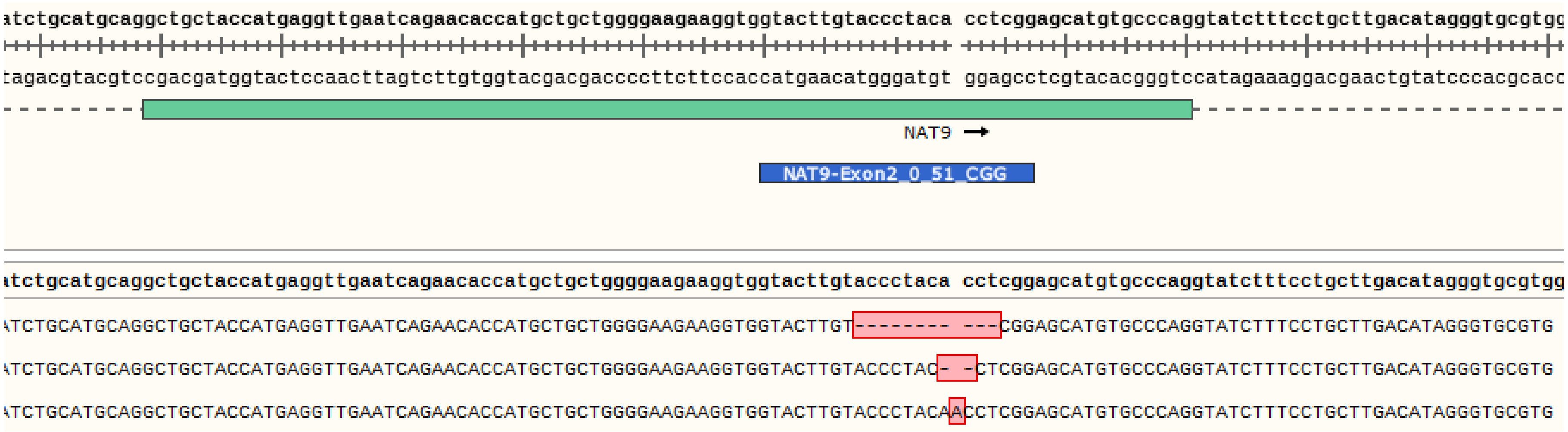

Name	Clone ID	Total # reads	# wt reads(%)	#1-Indel	#1-Reads(%)	#2-Indel	#2-Reads(%)	#3-Indel	#3-Reads(%)
NAT9 2-G4	039R	2342	0 (0.0%)	-2	799 (34.1%)	-11	768 (32.8%)	1	744 (31.8%)

Clone ID	Clone ID	Total # reads	# wt reads(%)	#1-Indel	#1-Reads(%)	#2-Indel	#2-Reads(%)	#3-Indel	#3-Reads(%)
NAT9 2-G4	039R	1495	0 (0.0%)	-2	507 (33.9%)	-11	487 (32.6%)	1	466 (31.2%)

Clone 040R (-16/-4/+1)

NGS 1, NGS 2 (cell bank)

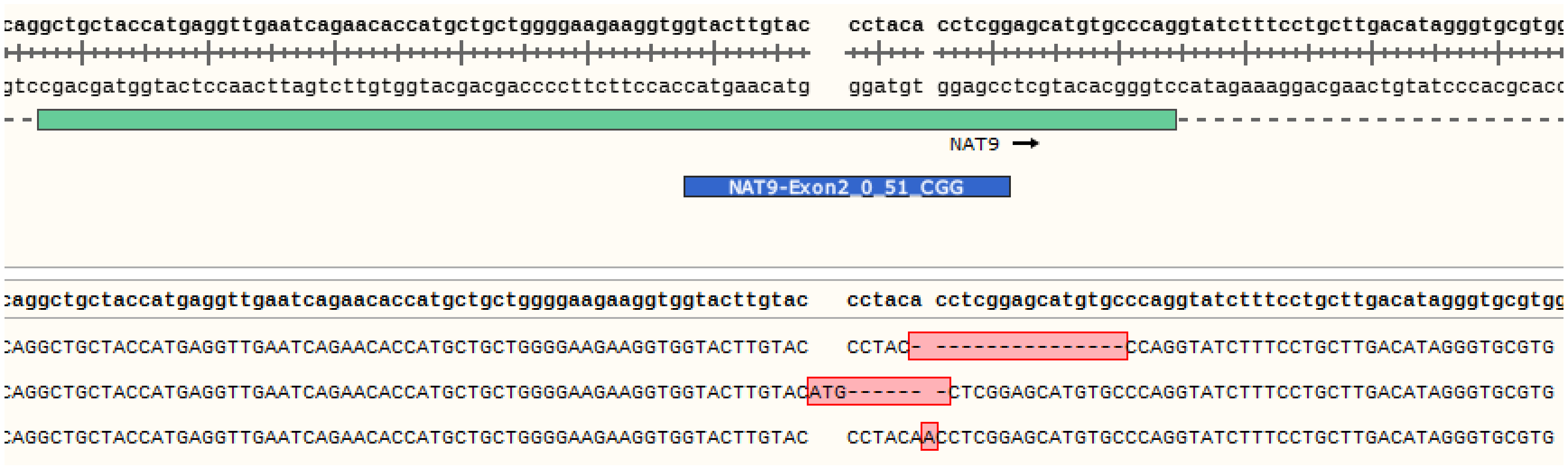

Name	Clone ID	Total # reads	# wt reads(%)	#1-Indel	#1-Reads(%)	#2-Indel	#2-Reads(%)	#3-Indel	#3-Reads(%)
NAT9 1-C10	040R	2606	0 (0.0%)	-16	931 (35.7%)	-4	873 (33.5%)	1	774 (29.7%)

Clone ID	Clone ID	Total # reads	# wt reads(%)	#1-Indel	#1-Reads(%)	#2-Indel	#2-Reads(%)	#3-Indel	#3-Reads(%)
NAT9 1-C10	040R	1550	0 (0.0%)	-16	606 (39.1%)	-4	475 (30.6%)	1	428 (27.6%)

Clone 041R (-4/+1)

NGS 1, fragment analysis, NGS 2 (cell bank)

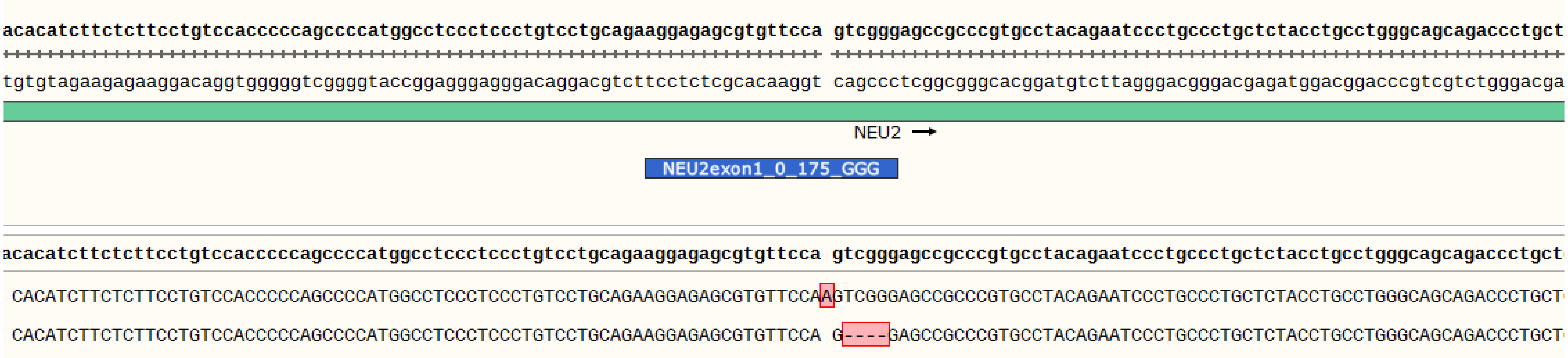

Name	Clone ID	Total # reads	# wt reads(%)	#1-Indel	#1-Reads(%)	#2-Indel	#2-Reads(%)
NEU2 1E11	041R	844	47 (5.6%)	-4	386 (45.7%)	1	383 (45.4%)

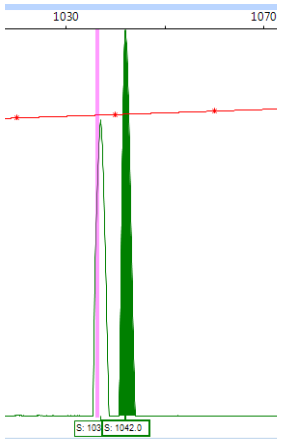

Clone ID	Clone ID	Total # reads	# wt reads(%)	#1-Indel	#1-Reads(%)	#2-Indel	#2-Reads(%)
NEU2 1E11	041R	728	0 (0.0%)	-4	381 (52.3%)	1	334 (45.9%)

Clone 042R (-22/-698)

NGS 1, 3kb PCR, topo sequencing, fragment analysis, NGS 2 (cell bank)

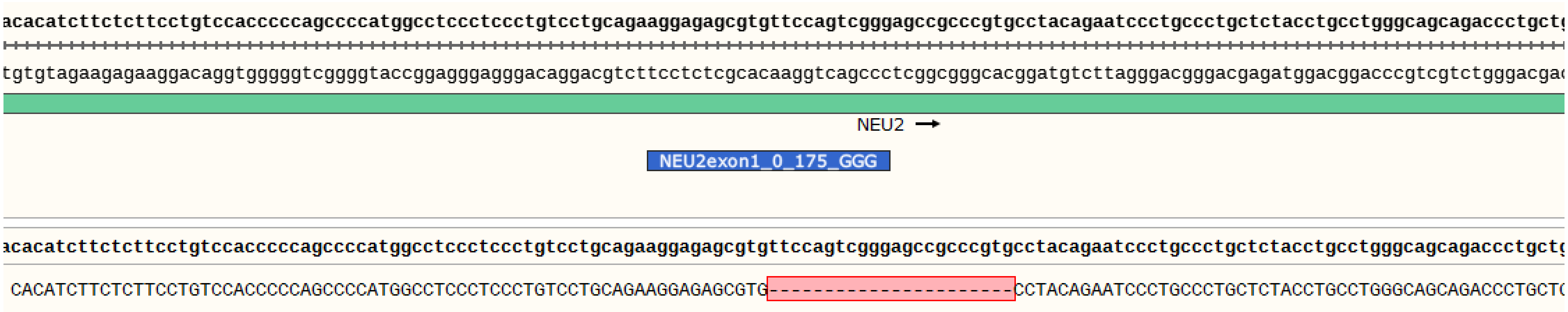

Name	Clone ID	Total # reads	# wt reads(%)	#1-Indel	#1-Reads(%)	#2-Indel	#2-Reads(%)
NEU2 1G8	042R	1389	0 (0.0%)	-22	1371 (98.7%)	-23	16 (1.2%)

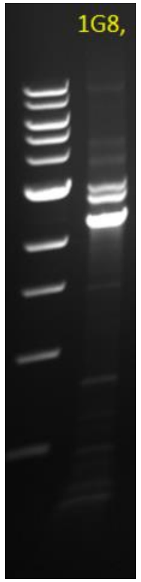

Clone 042R (-22/-698)

NGS 1, 3kb PCR, topo sequencing, fragment analysis, NGS 2 (cell bank)

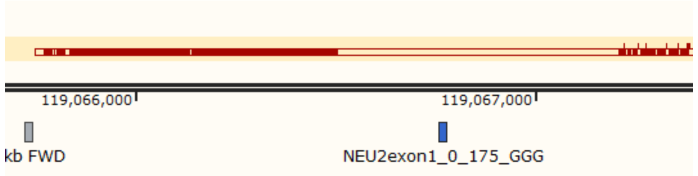

Topo -698bp

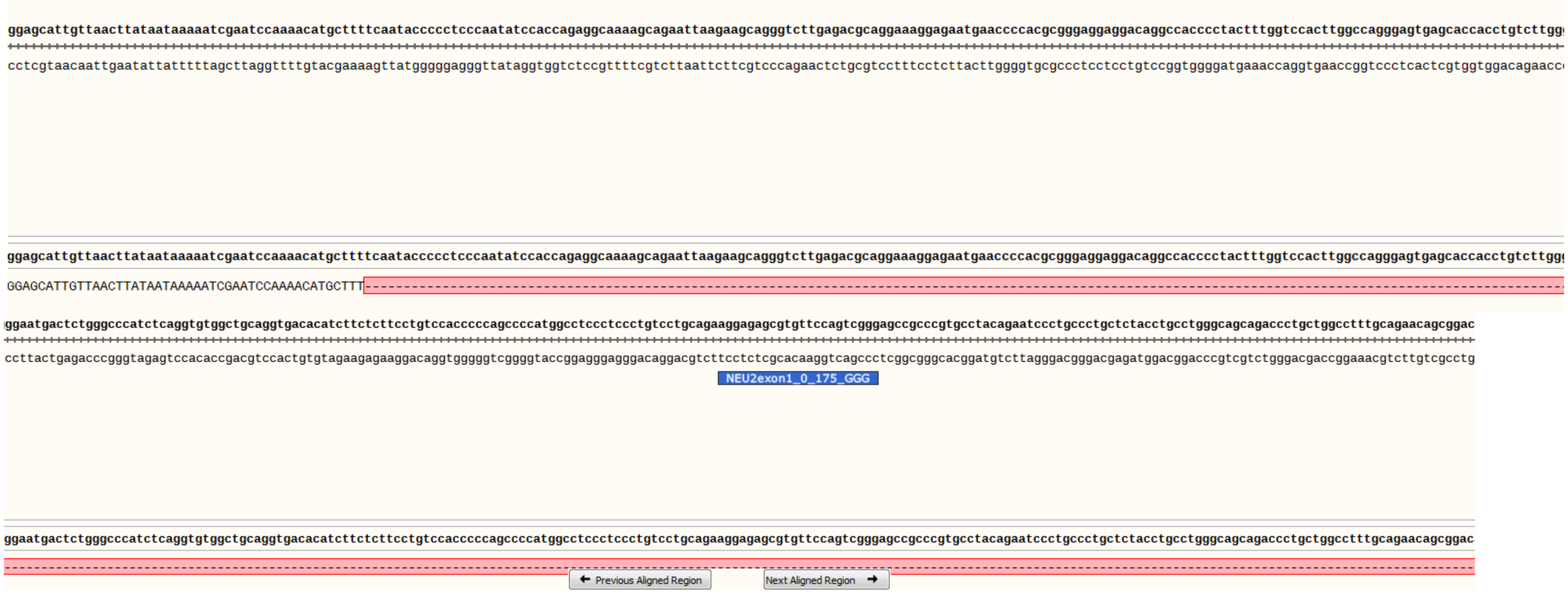

Clone 042R (-22/-698)

NGS 1, 3kb PCR, topo sequencing, fragment analysis, NGS 2 (cell bank)

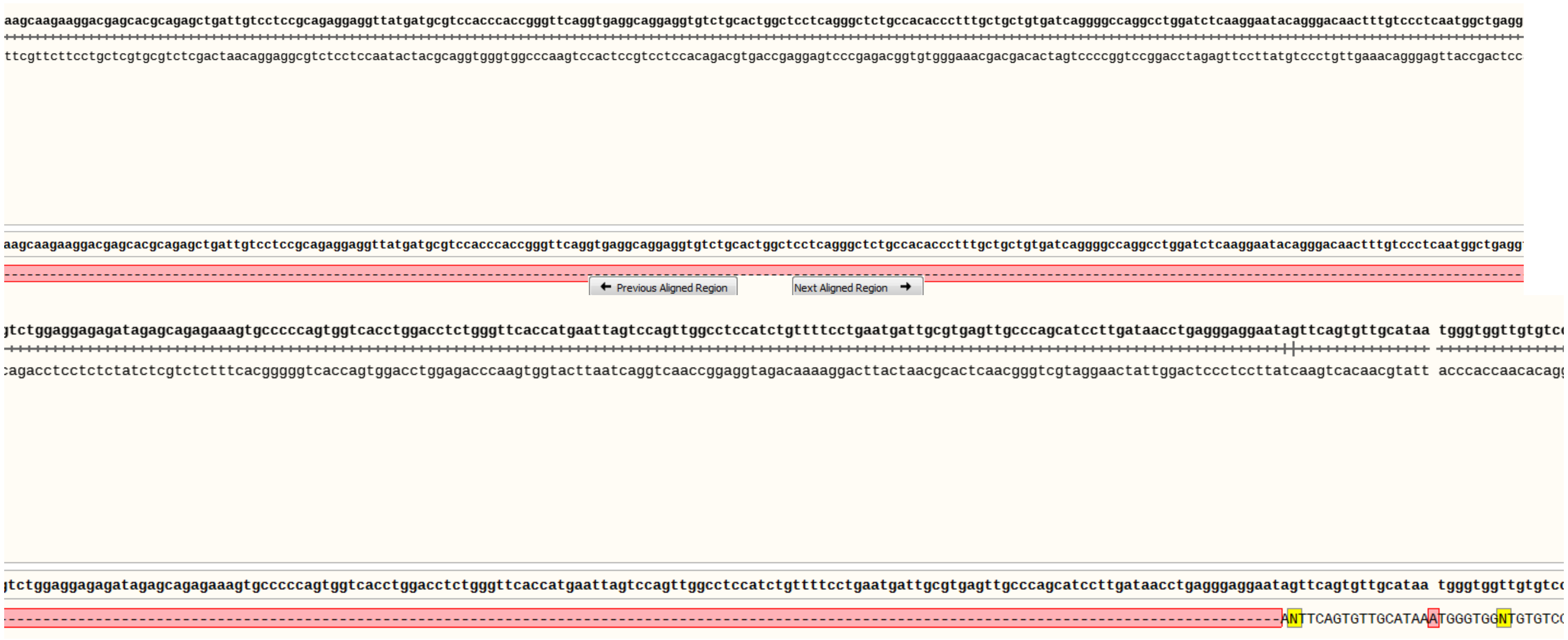

Deleted sequence:

tcaatacccccctcccaatatccaccagaggcaaaagcagaattaagaagcagggtcttgagacgcaggaaaggagaatgaaccccacgcgggaggagga
caggccacccctactttggtccacttggccagggagtgagcaccacctgtcttggggaatgactctgggcccatctcaggtgtggctgcaggtgacacatcttct
cttcctgtccacccccagccccatggcctccctccctgtcctgcagaaggagagcgtgttccagtcgggagccgcccgtgcctacagaatccctgccctgctcta
cctgcctgggcagcagaccctgctggcctttgcagaacagcggacaagcaagaaggacgagcacgcagagctgattgtcctccgcagaggaggttatgatg
cgtccacccaccgggttcaggtgaggcaggaggtgtctgcactggctcctcagggctctgccacaccctttgctgctgtgatcaggggccaggcctggatctca
aggaatacagggacaactttgtccctcaatggctgaggtctggaggagagatagagcagagaaagtgcccccagtggtcacctggacctctgggttcaccat
gaattagtccagttggcctccatctgttttcctgaatgattgcgtgagttgcccagcatccttgataacctgagggaggaat

Clone 042R (-22/-698)

NGS 1, 3kb PCR, topo sequencing, fragment analysis, NGS 2 (cell bank)

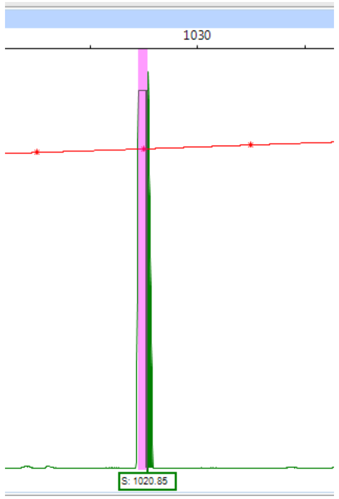

Clone ID	Clone ID	Total # reads	# wt reads(%)	#1-Indel	#1-Reads(%)	#2-Indel	#2-Reads(%)
NEU2 1G8	042R	1382	0 (0.0%)	-22	1346 (97.4%)	-23	31 (2.2%)

Clone 043R (-4/-8)

NGS 1, NGS 2 (cell bank)

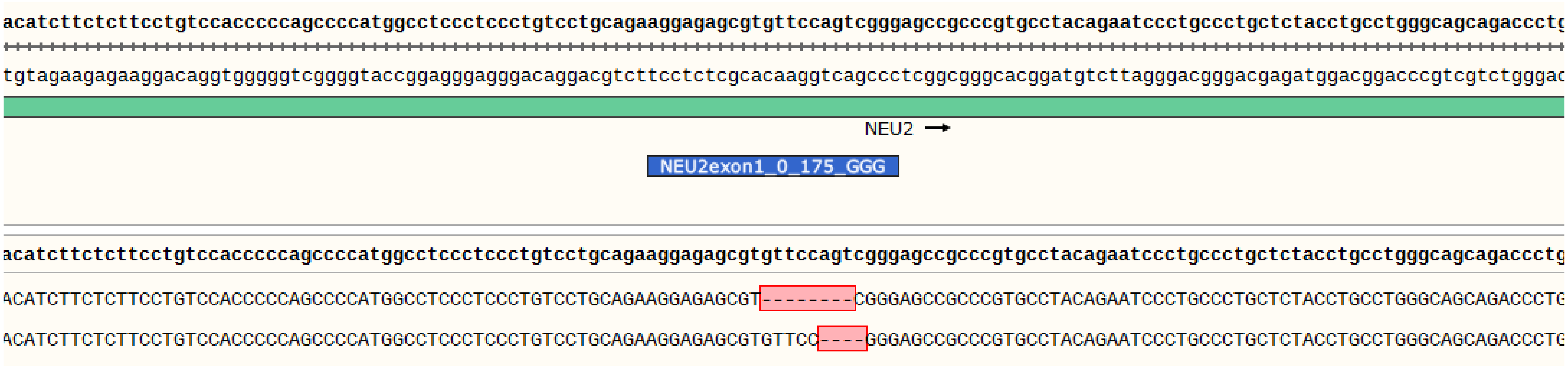

Name	Clone ID	Total # reads	# wt reads(%)	#1-Indel	#1-Reads(%)	#2-Indel	#2-Reads(%)
NEU2 2C2	043R	1568	0 (0.0%)	-8	815 (52.0%)	-4	725 (46.2%)

Clone ID	Clone ID	Total # reads	# wt reads(%)	#1-Indel	#1-Reads(%)	#2-Indel	#2-Reads(%)
NEU2 2C2	043R	1211	0 (0.0%)	-8	680 (56.2%)	-4	504 (41.6%)

Clone 045R (+4/-134)

NGS 1, fragment analysis, topo sequencing, NGS 2 (cell bank)

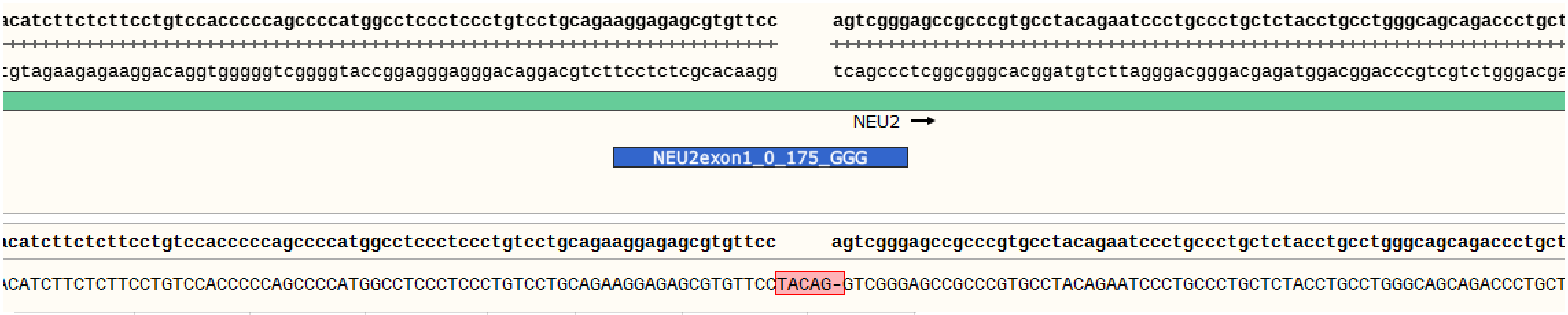

Name	Clone ID	Total # reads	# wt reads(%)	#1-Indel	#1-Reads(%)	#2-Indel	#2-Reads(%)
NEU2 2H3	045R	1077	2 (0.2%)	4	1063 (98.7%)	3	11 (1.0%)

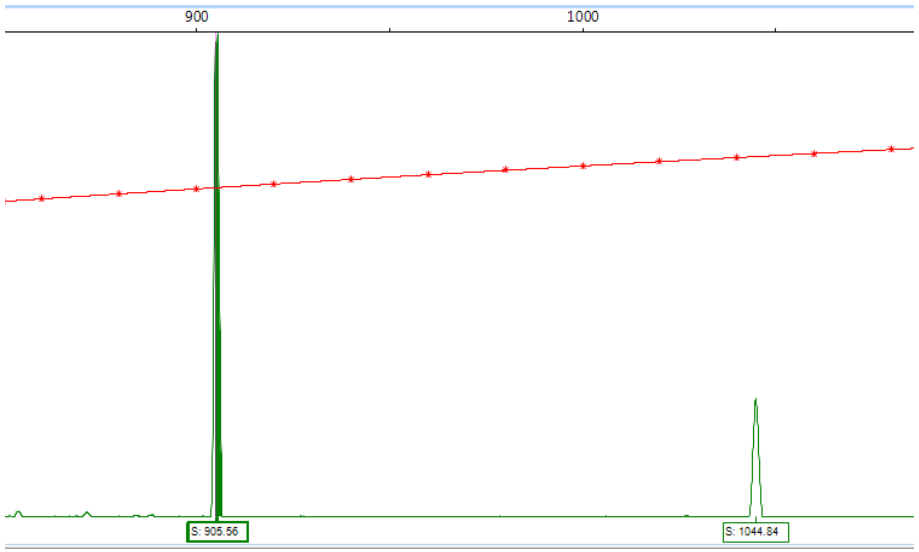

Clone 045R (+4/-134)

NGS 1, fragment analysis, topo sequencing, NGS 2 (cell bank)

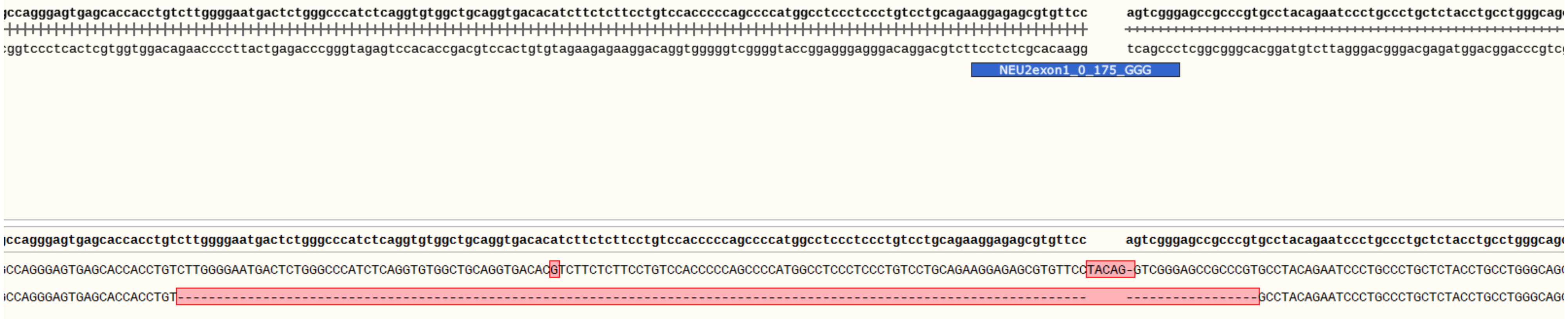

Deleted sequence:

cttggggaatgactctgggcccatctcaggtgtggctgcaggtgacacatcttctcttcctgtccacccccagccccatggcctccctccctgtcctgcagaaggagagcgtgttccagtcggga
gccgcccgt

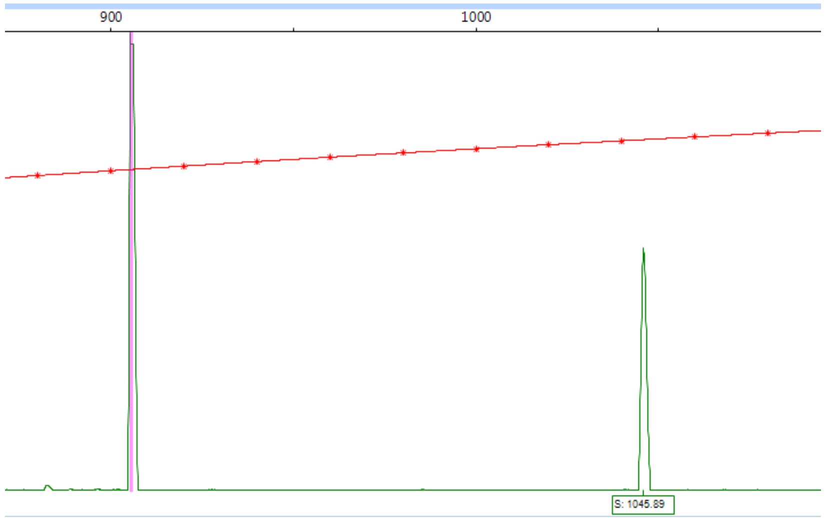

Clone ID	Clone ID	Total # reads	# wt reads(%)	#1-Indel	#1-Reads(%)	#2-Indel	#2-Reads(%)
NEU2 2H3	045R	1005	0 (0.0%)	4	989 (98.4%)	3	15 (1.5%)

Clone 046R (-2/-2)

Fragment analysis, direct sequencing, NGS 2 (cell bank)

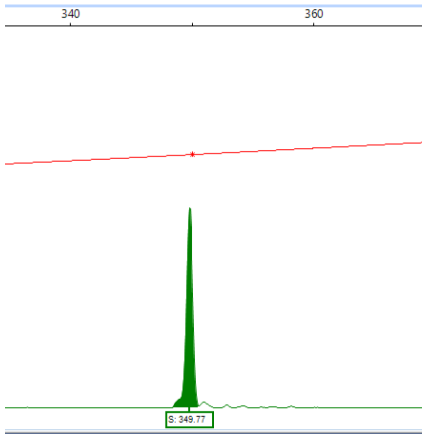

ND NGS, -2 fragment analysis

SNP T/C SNP G/A

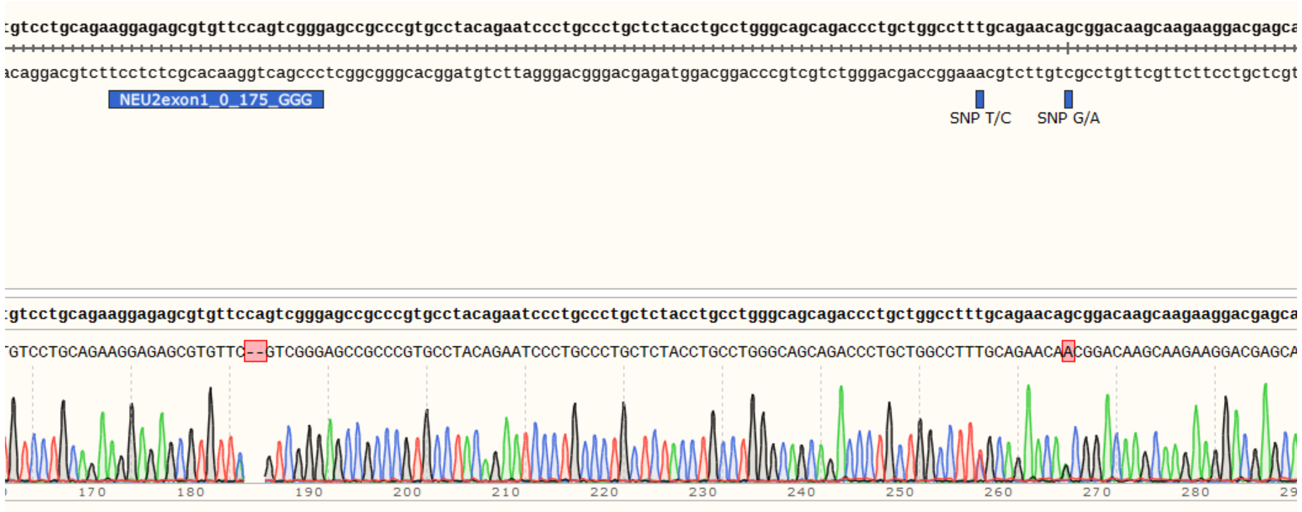

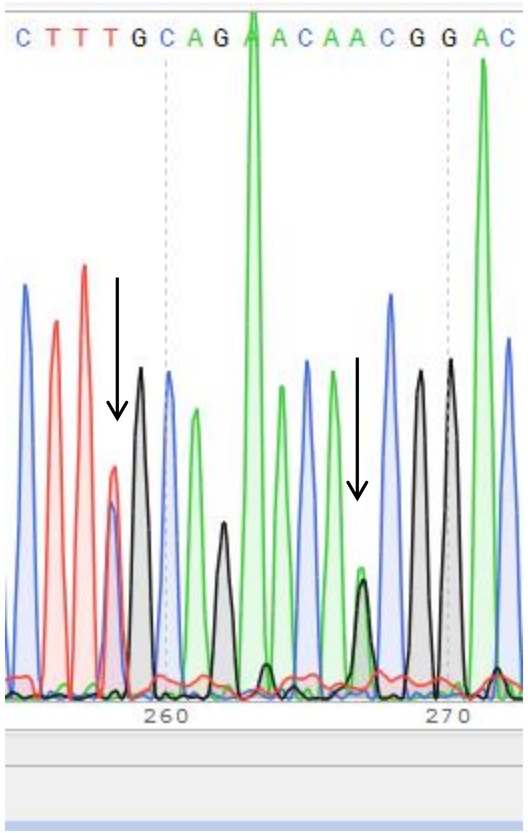

Clone ID	Clone ID	Total # reads	# wt reads(%)	#1-Indel	#1-Reads(%)	#2-Indel	#2-Reads(%)
NEU2 C4	046R	906	0 (0.0%)	-2	886 (97.8%)	-3	19 (2.1%)

Clone 047R (-53/+1)

Fragment analysis, topo sequencing, NGS 2 (cell bank)

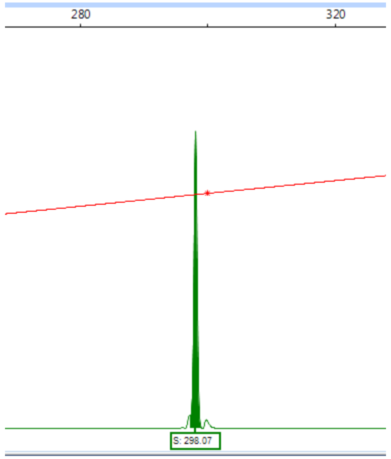

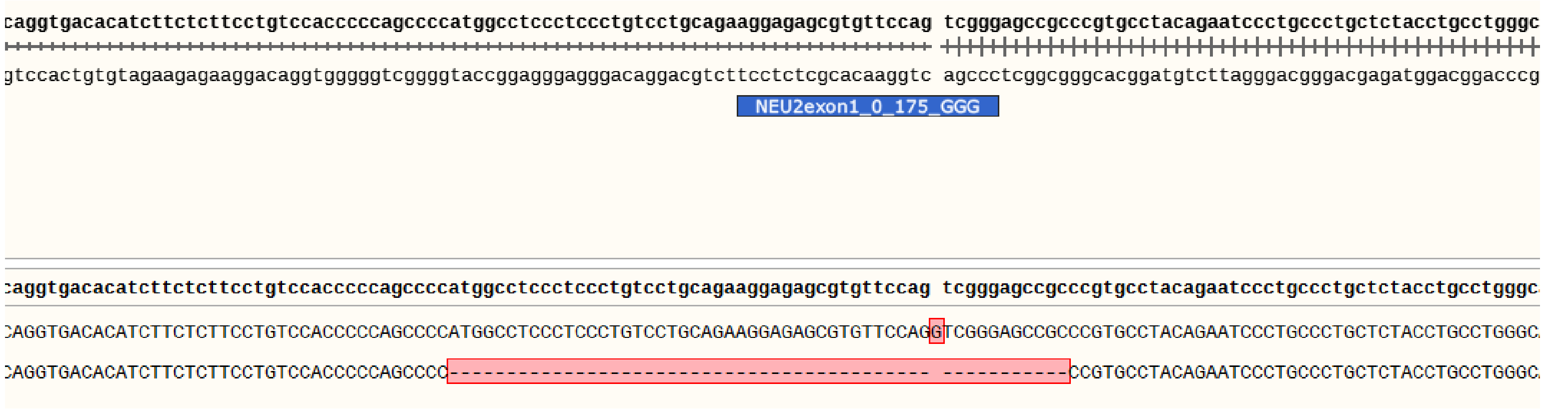

Clone ID	Clone ID	Total # reads	# wt reads(%)	#1-Indel	#1-Reads(%)	#2-Indel	#2-Reads(%)
NEU2 B3	047R	986	0 (0.0%)	-53	598 (60.6%)	1	367 (37.2%)

Clone 063R (+1/-5)

Fragment analysis, topo sequencing, NGS 2 (cell bank)

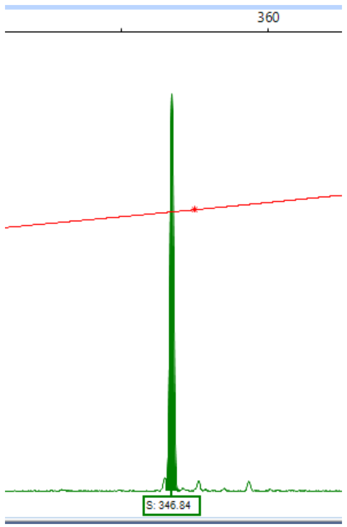

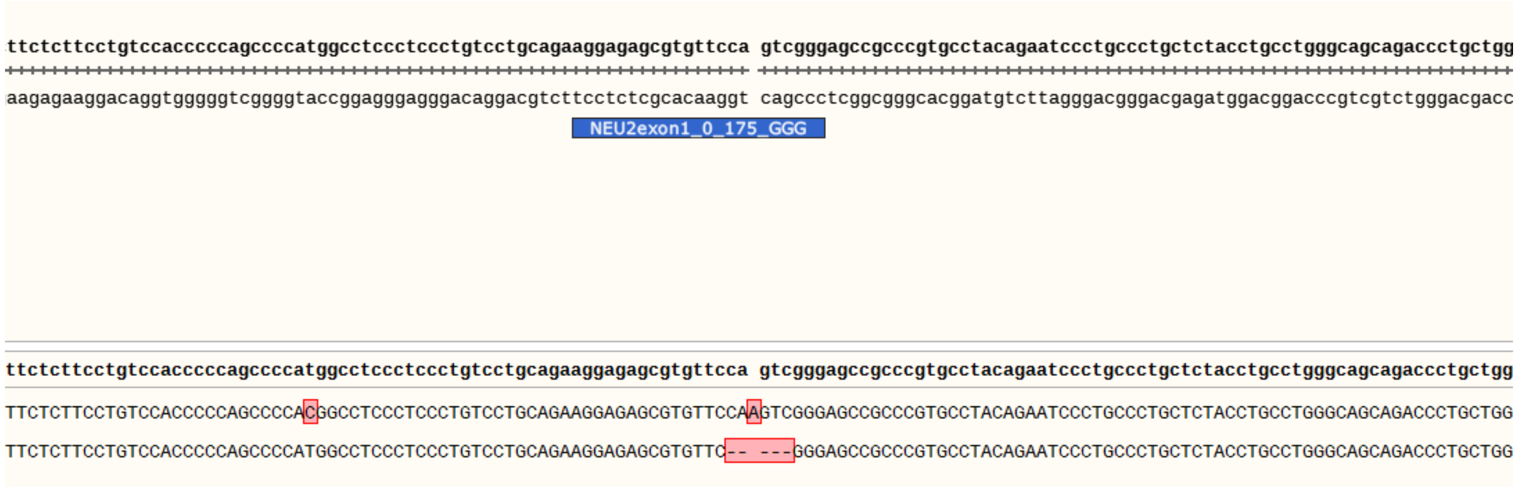

Clone ID	Clone ID	Total # reads	# wt reads(%)	#1-Indel	#1-Reads(%)	#2-Indel	#2-Reads(%)
NEU2 from CNT/EP 4B3 data NEU2		1154	1 (0.1%)	-5	590 (51.1%)	1	544 (47.1%)
NEU2 from CNT/EP 4B3 data EP300	063R	2310	2181 (94.4%)	0	2284 (98.9%)	-1	24 (1.0%)

Clone 065R (-11/-17)

NGS 1, NGS 2 (cell bank)

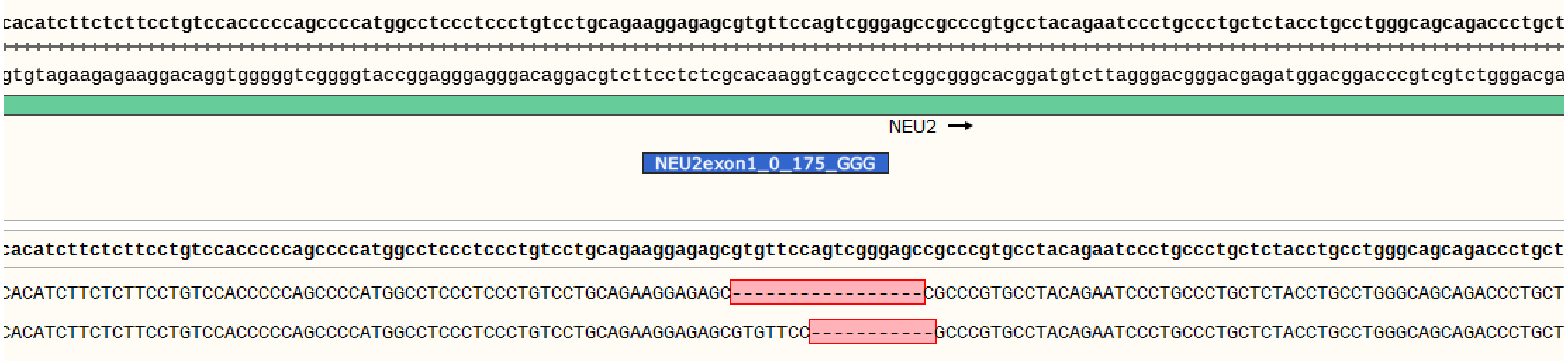

Name	Clone ID	Total # reads	# wt reads(%)	#1-Indel	#1-Reads(%)	#2-Indel	#2-Reads(%)
NEU2 from CNT/NEU 1G8 data NEU2		884	6 (0.7%)	-11	431 (48.8%)	-17	430 (48.6%)
NEU2 from CNT/NEU 1G8 data CNTD2	065R	679	630 (92.8%)	0	656 (96.6%)	-1	22 (3.2%)

Clone ID	Clone ID	Total # reads	# wt reads(%)	#1-Indel	#1-Reads(%)	#2-Indel	#2-Reads(%)
NEU2 from CNT/NEU 1G8 data NEU2		6561	0 (0.0%)	-17	3267 (49.8%)	-11	3164 (48.2%)
NEU2 from CNT/NEU 1G8 data CNTD2	065R	10987	10345 (94.2%)	0	10742 (97.8%)	-1	227 (2.1%)

Clone 066R (-17/-4)

NGS 1, NGS 2 (cell bank)

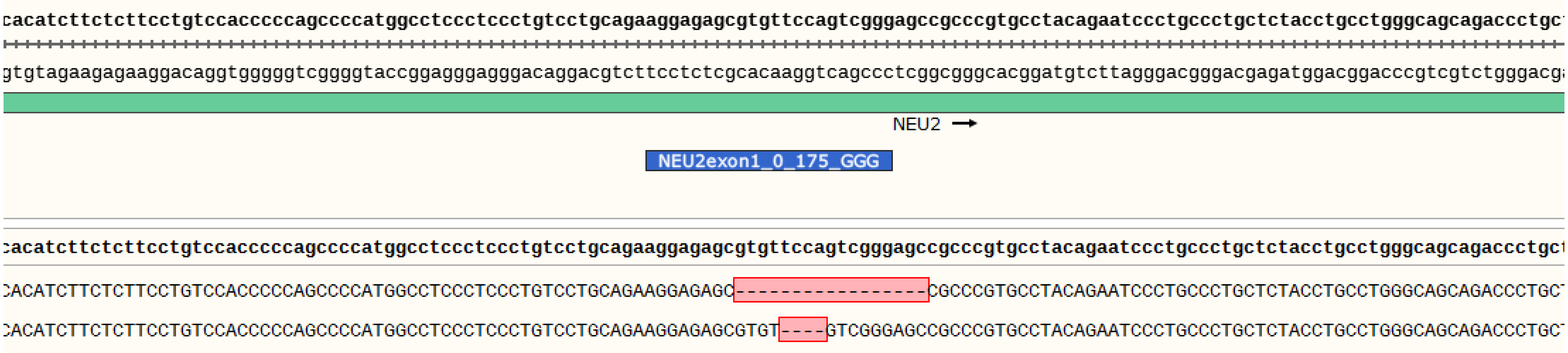

Name	Clone ID	Total # reads	# wt reads(%)	#1-Indel	#1-Reads(%)	#2-Indel	#2-Reads(%)
NEU2 from CNT/NEU 1H2 data NEU2		1207	2 (0.2%)	-17	642 (53.2%)	-4	537 (44.5%)
NEU2 from CNT/NEU 1H2 data CNTD2	066R	1198	1105 (92.2%)	0	1157 (96.6%)	-1	36 (3.0%)

Clone ID	Clone ID	Total # reads	# wt reads(%)	#1-Indel	#1-Reads(%)	#2-Indel	#2-Reads(%)
NEU2 from CNT/NEU 1H2 data NEU2		1186	0 (0.0%)	-17	660 (55.6%)	-4	511 (43.1%)
NEU2 from CNT/NEU 1H2 data CNTD2	066R	13715	12896 (94.0%)	0	13393 (97.7%)	-1	286 (2.1%)

Clone 052R (-2/-11)

NGS 1, NGS 2 (cell bank)

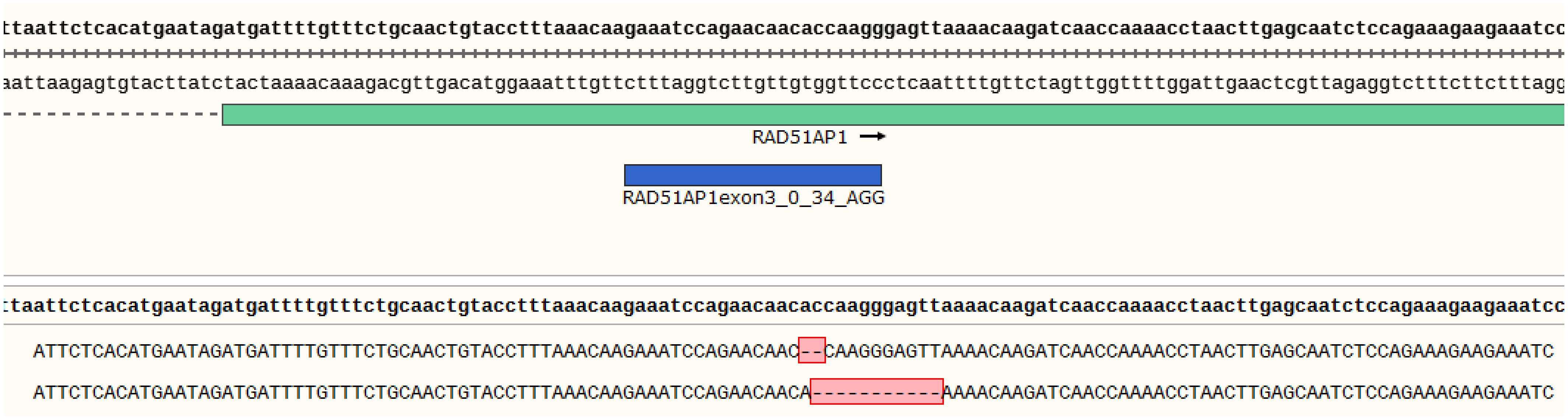

Name	Clone ID	Total # reads	# wt reads(%)	#1-Indel	#1-Reads(%)	#2-Indel	#2-Reads(%)
RAD51SP1 1H4	052R	1589	0 (0.0%)	-2	801 (50.4%)	-11	775 (48.8%)

Clone ID	Clone ID	Total # reads	# wt reads(%)	#1-Indel	#1-Reads(%)	#2-Indel	#2-Reads(%)
RAD51SP1 1H4	052R	4239	0 (0.0%)	-2	2186 (51.6%)	-11	2007 (47.3%)

Clone 054R (-2/-1)

NGS 1, NGS 2 (cell bank)

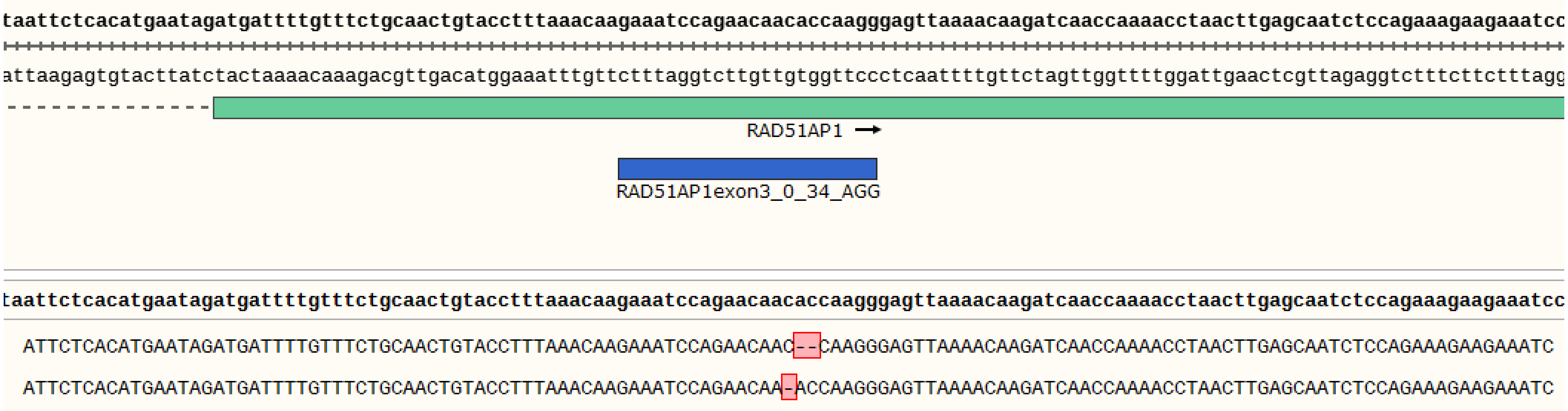

Name	Clone ID	Total # reads	# wt reads(%)	#1-Indel	#1-Reads(%)	#2-Indel	#2-Reads(%)
RAD51SP1 1E2	054R	1059	0 (0.0%)	-2	559 (52.8%)	-1	496 (46.8%)

Clone ID	Clone ID	Total # reads	# wt reads(%)	#1-Indel	#1-Reads(%)	#2-Indel	#2-Reads(%)
RAD51SP1 1E2	054R	2919	0 (0.0%)	-2	1574 (53.9%)	-1	1327 (45.5%)

Clone 055R (-1/+1)

NGS 1, NGS 2 (cell bank)

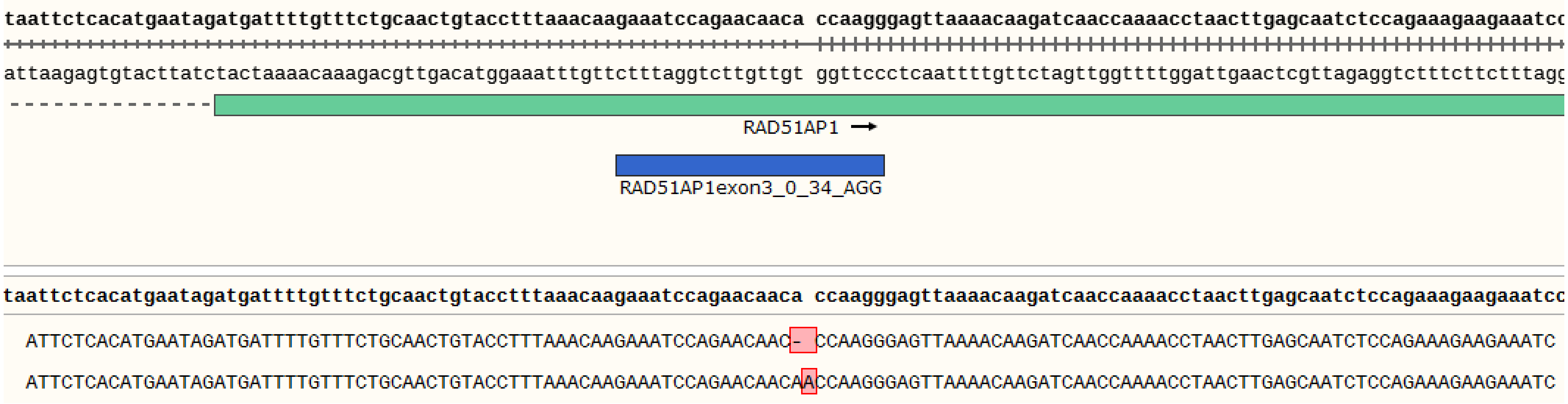

Name	Clone ID	Total # reads	# wt reads(%)	#1-Indel	#1-Reads(%)	#2-Indel	#2-Reads(%)
RAD51SP1 1D5	055R	3937	1 (0.0%)	-1	2069 (52.6%)	1	1820 (46.2%)

Clone ID	Clone ID	Total # reads	# wt reads(%)	#1-Indel	#1-Reads(%)	#2-Indel	#2-Reads(%)
RAD51SP1 1D5	055R	4350	0 (0.0%)	-1	2313 (53.2%)	1	1990 (45.7%)

Clone 056R (-10/+5)

NGS 1, NGS 2 (cell bank)

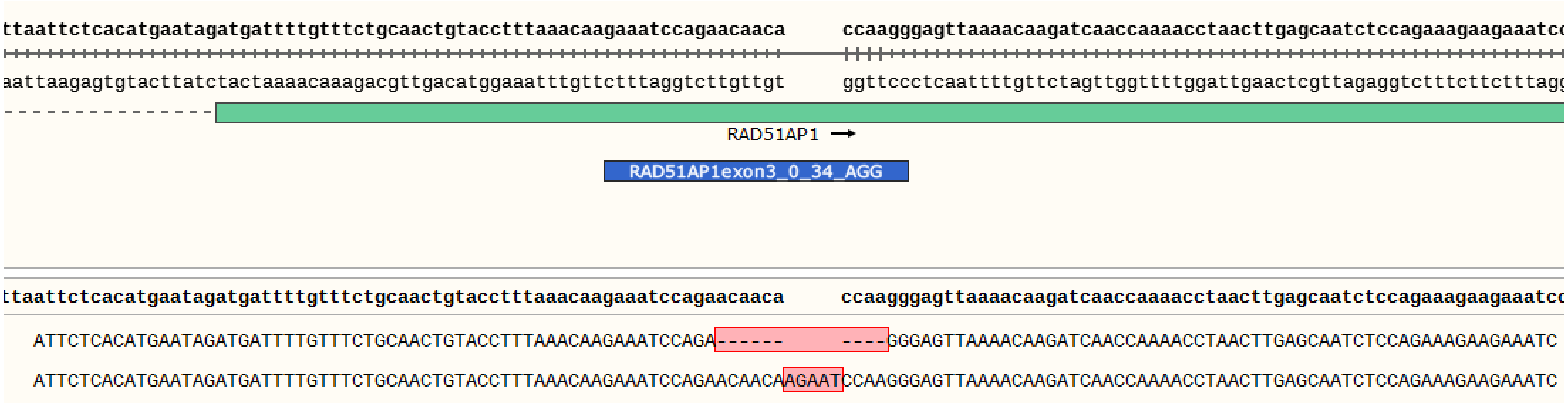

Name	Clone ID	Total # reads	# wt reads(%)	#1-Indel	#1-Reads(%)	#2-Indel	#2-Reads(%)
RAD51SP1 1C10	056R	1913	0 (0.0%)	-10	1075 (56.2%)	5	814 (42.6%)

Clone ID	Clone ID	Total # reads	# wt reads(%)	#1-Indel	#1-Reads(%)	#2-Indel	#2-Reads(%)
RAD51SP1 1C10	056R	3840	0 (0.0%)	-10	2119 (55.2%)	5	1670 (43.5%)

Clone 057R (-2/+1)

NGS 1, NGS 2 (cell bank)

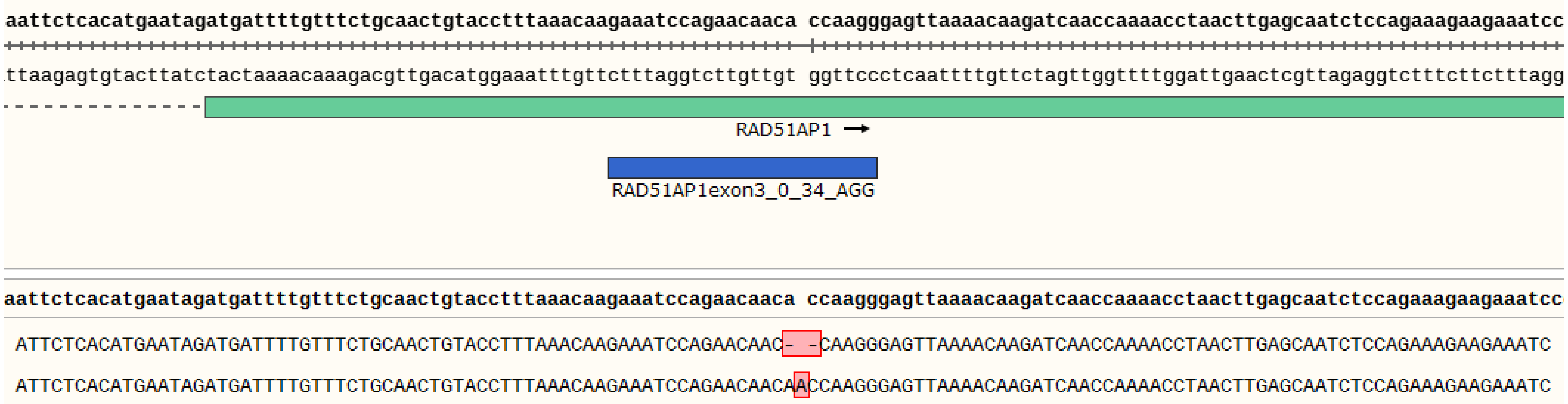

Name	Clone ID	Total # reads	# wt reads(%)	#1-Indel	#1-Reads(%)	#2-Indel	#2-Reads(%)
RAD51SP1 1C7	057R	563	0 (0.0%)	-2	317 (56.3%)	1	238 (42.3%)

Clone ID	Clone ID	Total # reads	# wt reads(%)	#1-Indel	#1-Reads(%)	#2-Indel	#2-Reads(%)
RAD51SP1 1C7	057R	3894	11 (0.3%)	-2	2094 (53.8%)	1	1757 (45.1%)

Clone 058R (-59/+1)

NGS 1, NGS 2 (cell bank)

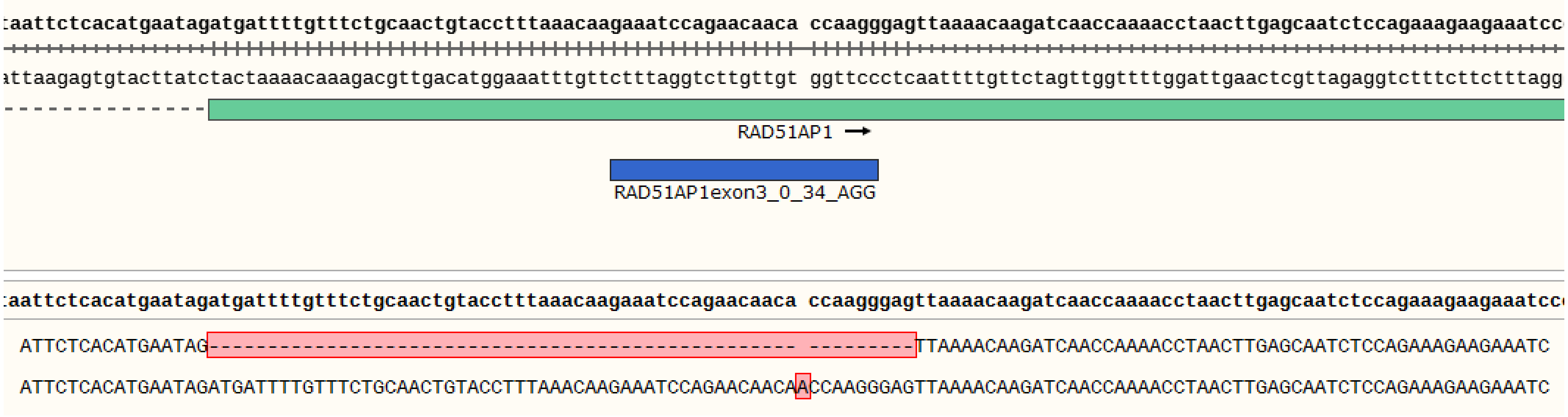

Name	Clone ID	Total # reads	# wt reads(%)	#1-Indel	#1-Reads(%)	#2-Indel	#2-Reads(%)
RAD51SP1 2G4	058R	2541	1 (0.0%)	-59	1600 (63.0%)	1	924 (36.4%)

Clone ID	Clone ID	Total # reads	# wt reads(%)	#1-Indel	#1-Reads(%)	#2-Indel	#2-Reads(%)
RAD51SP1 2G4	058R	4558	0 (0.0%)	-59	2762 (60.6%)	1	1754 (38.5%)

Clone 059R (-22/-11)

NGS 1, NGS 2 (cell bank)

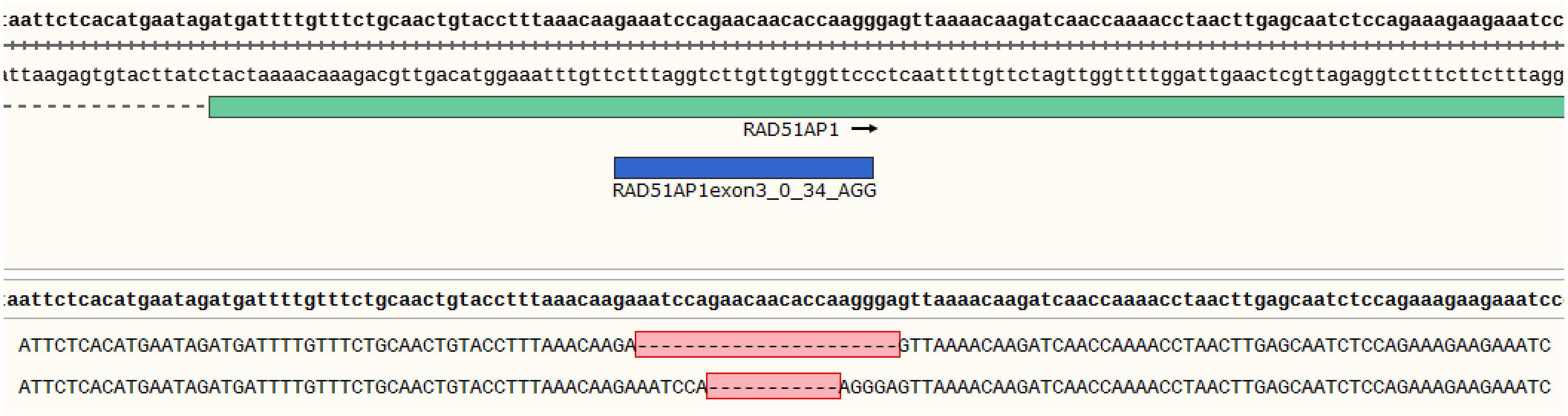

Name	Clone ID	Total # reads	# wt reads(%)	#1-Indel	#1-Reads(%)	#2-Indel	#2-Reads(%)
RAD51SP1 4F10	059R	1627	0 (0.0%)	-22	820 (50.4%)	-11	794 (48.8%)

Clone ID	Clone ID	Total # reads	# wt reads(%)	#1-Indel	#1-Reads(%)	#2-Indel	#2-Reads(%)
RAD51SP1 4F10	059R	4108	0 (0.0%)	-11	2038 (49.6%)	-22	2028 (49.4%)

Clone 060R (+1/+23)

NGS 1, NGS 2 (cell bank)

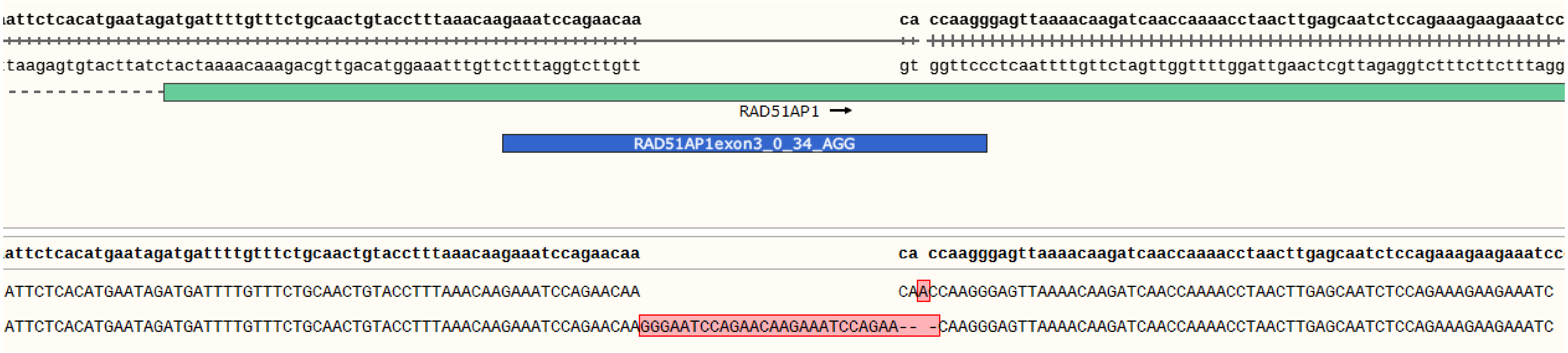

Name	Clone ID	Total # reads	# wt reads(%)	#1-Indel	#1-Reads(%)	#2-Indel	#2-Reads(%)
RAD51SP1 4D5	060R	1508	2 (0.1%)	1	833 (55.2%)	23	658 (43.6%)

Clone ID	Clone ID	Total # reads	# wt reads(%)	#1-Indel	#1-Reads(%)	#2-Indel	#2-Reads(%)
RAD51SP1 4D5	060R	4213	2 (0.0%)	1	2543 (60.4%)	23	1597 (37.9%)

Clone 061R (-2/-32)

NGS 1, NGS 2 (cell bank)

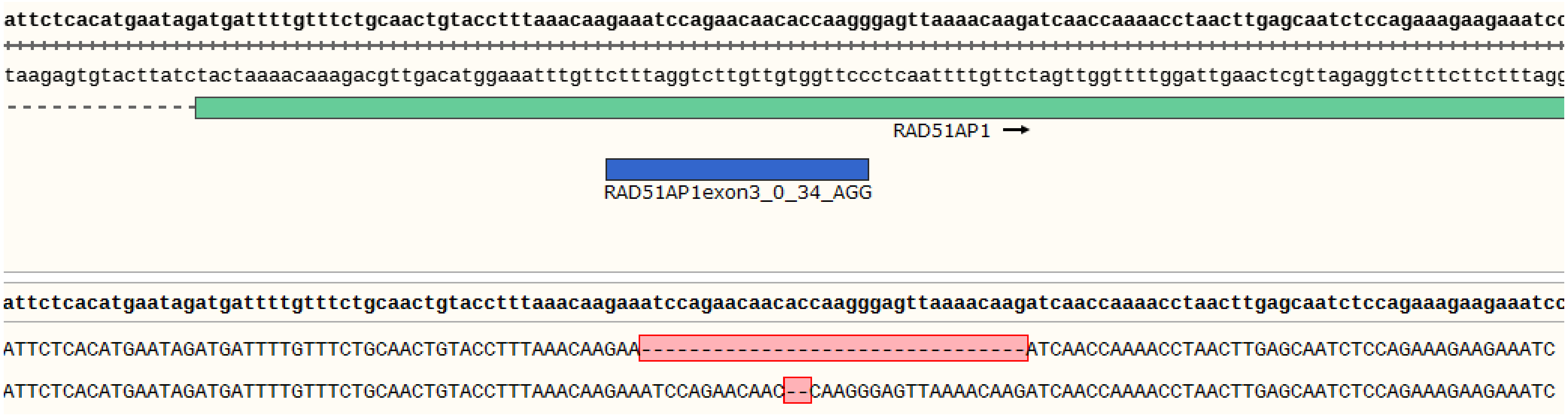

Name	Clone ID	Total # reads	# wt reads(%)	#1-Indel	#1-Reads(%)	#2-Indel	#2-Reads(%)
RAD51SP1 4B8	061R	2905	0 (0.0%)	-2	1459 (50.2%)	-32	1416 (48.7%)

Clone ID	Clone ID	Total # reads	# wt reads(%)	#1-Indel	#1-Reads(%)	#2-Indel	#2-Reads(%)
RAD51SP1 4B8	061R	3219	0 (0.0%)	-32	1718 (53.4%)	-2	1476 (45.9%)

Clone 068R (+1/-7)

NGS 1, NGS 2 (cell bank)

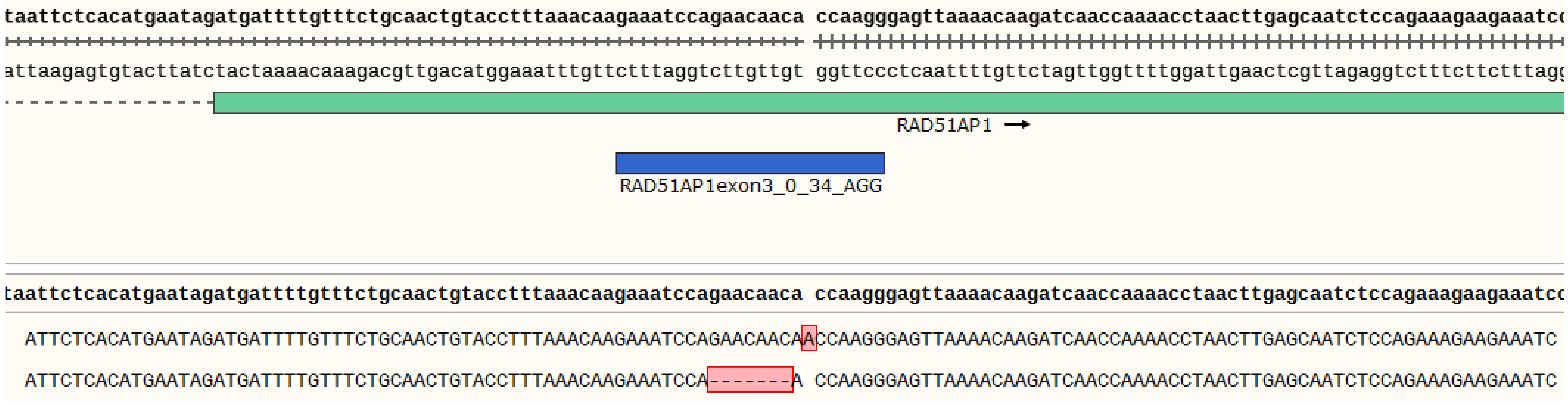

Name	Clone ID	Total # reads	# wt reads(%)	#1-Indel	#1-Reads(%)	#2-Indel	#2-Reads(%)
RAD51AP1 2F10	068R	1611	1 (0.1%)	1	1055 (65.5%)	-7	543 (33.7%)

Clone ID	Clone ID	Total # reads	# wt reads(%)	#1-Indel	#1-Reads(%)	#2-Indel	#2-Reads(%)
RAD51AP1 2F10	068R	3729	1 (0.0%)	1	2578 (69.1%)	-7	1112 (29.8%)

Clone 051R (-23/-23/-5)

NGS 1, NGS 2 (cell bank)

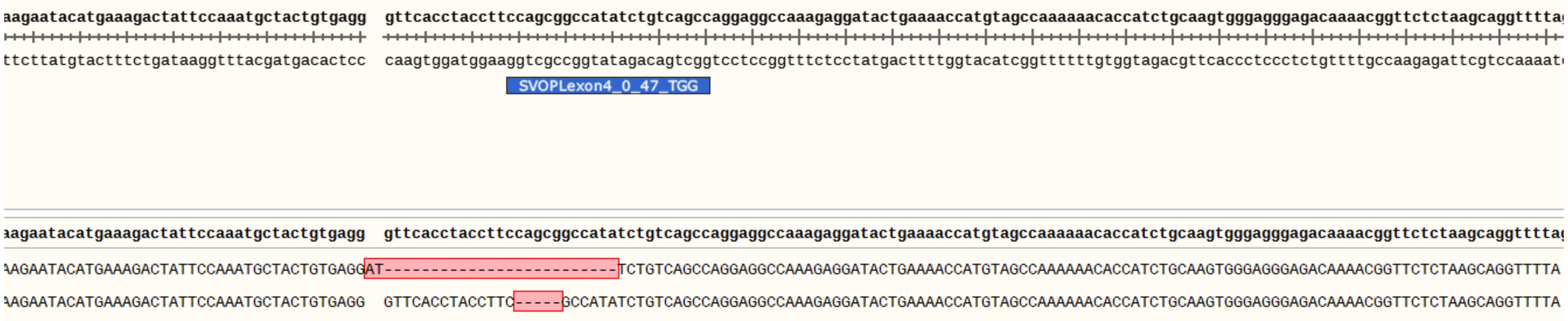

Name	Clone ID	Total # reads	# wt reads(%)	#1-Indel	#1-Reads(%)	#2-Indel	#2-Reads(%)	#3-Indel	#3-Reads(%)	#4-Indel	#4-Reads(%)
SVOPL 2G7	051R	5852	8 (0.1%)	-23	4128 (70.5%)	-5	1598 (27.3%)	-24	77 (1.3%)	-6	27 (0.5%)

Clone ID	Clone ID	Total # reads	# wt reads(%)	#1-Indel	#1-Reads(%)	#2-Indel	#2-Reads(%)
SVOPL 2G7	051R	34231	3 (0.0%)	-23	23809 (69.6%)	-5	10419 (30.5%)

Double knockout clones

EP300/COQ9 double knockout clones

Clone ID	In/dels
001D	-10/-1 EP, -5/+1 COQ
002D	-20/-28 EP, +1/-1 COQ
004D	-16/+1 EP, -5/+1 COQ
015D	-31/-5 EP, +1/+1 COQ9
016D	-11/-149 EP, +1/+1 COQ9
074D	-32/-14 EP, -2/+1 COQ
075D	-7/+1 EP, -19/+1 COQ
076D	-8/-1 EP, -23/-8 COQ
082D	-16/-2 EP, +1/+1 COQ

EP300/NAT9 double knockout clones

Clone ID	In/dels
003D	-17/-61 EP, -89/-2/+1 NAT
005D	-1/-7 EP, -1/-7/-2 NAT
007D	-16/+11 EP, -4/+1 NAT
008D	-16/-62 EP, -14/-2/-326 NAT
071D	-4/-17 EP, -4/-2/-1 NAT
072D	-41/-101 EP, -1/-1/-22 NAT
073D	-5/-187 EP, -1/-1/+1 NAT
079D	-16/+13 EP, -38/-2/+1 NAT

EP300/NEU2 double knockout clones

Clone ID	In/dels
049D	-14/-2 EP, -47/+1 NEU
051D	-32/-10 EP, -22/-19 NEU
052D	-2/-527 EP, -17/+1 NEU
053D	-11/+1 EP, -20/-263 NEU
055D	-203/-416 EP, -31/-8 NEU
058D	-50/-86 EP, -22/-19 NEU

CNTD2/COQ9 double knockout clones

Clone ID	In/dels
029D	-58/-20 CNT, -2/+1 COQ
030D	-8/+1 CNT, -16/-23 COQ
031D	+1/-13 CNT, -23/+1 COQ
036D	-1/-13 CNT, +1/+40 COQ
037D	-13/+25 CNT, +1/+1 COQ
038D	-13/-38 CNT, +1/+1 COQ
039D	-79/-1 CNT, +1/+1 COQ

CNTD2/NAT9 double knockout clones

Clone ID	Indels
025D	-32/-11 CNT, -82/-82/+1 NAT
027D	-13/-11 CNT, -19/-1/+1 NAT
034D	-35/+1 CNT, -1/-2/-328 NAT
035D	+1/-232 CNT, +1/+1/-1 NAT
044D	-268 CNT, -49/-272 NAT
077D	+14/-17 CNT, -7/-13/-1 NAT
078D	-16/-1 CNT, -2/-2/-5 NAT

CNTD2/NEU2 double knockout clones

Clone ID	In/dels
048D	-89/-814 CNT, -22/-128 NEU
059D	-13/-1 CNT, +1/+1 NEU
060D	-38/-1 CNT, +16/-100 NEU
061D	-67/-40 CNT, +1/-1 NEU
062D	-38/+2 CNT, +1/-112 NEU
064D	+1/+2 CNT, -8/+5 NEU
065D	-47/-11 CNT, -17/-1 NEU
066D	+1/+37 CNT, +1/-172 NEU

Clone 001D (-10/-1 EP300; -5/+1 COQ9)

EP300: NGS 1, NGS 2 (cell bank)

COQ9: NGS 1, NGS 2 (cell bank)

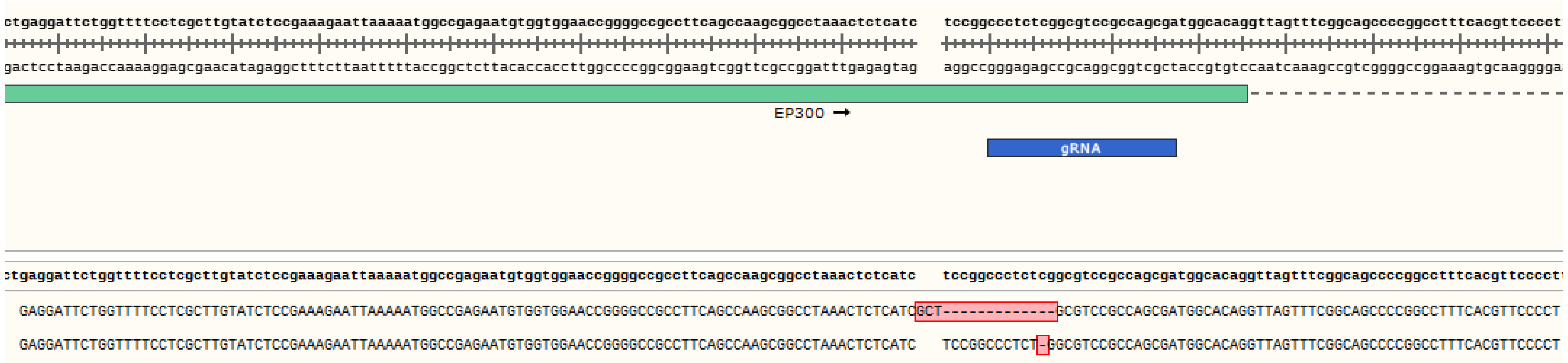

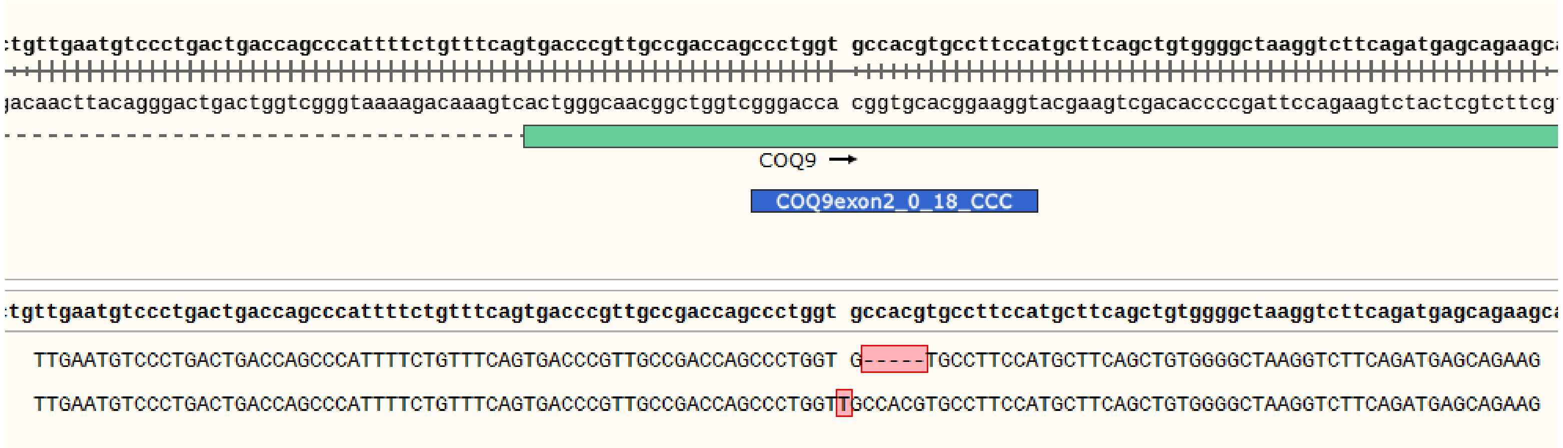

Name	Clone ID	Total # reads	# wt reads(%)	#1-Indel	#1-Reads(%)	#2-Indel	#2-Reads(%)
EPCOQ DKO data EP300 1-F8		1750	0 (0.0%)	-10	865 (49.4%)	-1	827 (47.3%)
EPCOQ DKO data COQ9 1-F8	001D	56	0 (0.0%)	-5	34 (60.7%)	1	21 (37.5%)

Clone ID	Clone ID	Total # reads	# wt reads(%)	#1-Indel	#1-Reads(%)	#2-Indel	#2-Reads(%)
EPCOQ DKO data EP300 1-F8		2642	2 (0.1%)	-10	1359 (51.4%)	-1	1234 (46.7%)
EPCOQ DKO data COQ9 1-F8	001D	2348	0 (0.0%)	-5	1196 (50.9%)	1	1057 (45.0%)

Clone 002D (-20/-28 EP300; +1/-1 COQ9)

EP300: NGS 1, NGS 2 (cell bank)
COQ9: NGS 1, NGS 2 (cell bank)

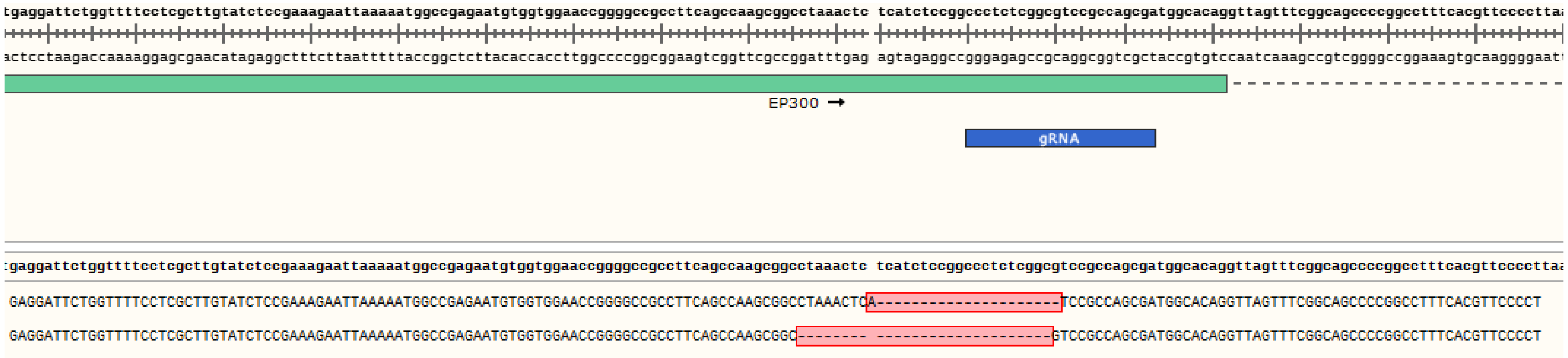

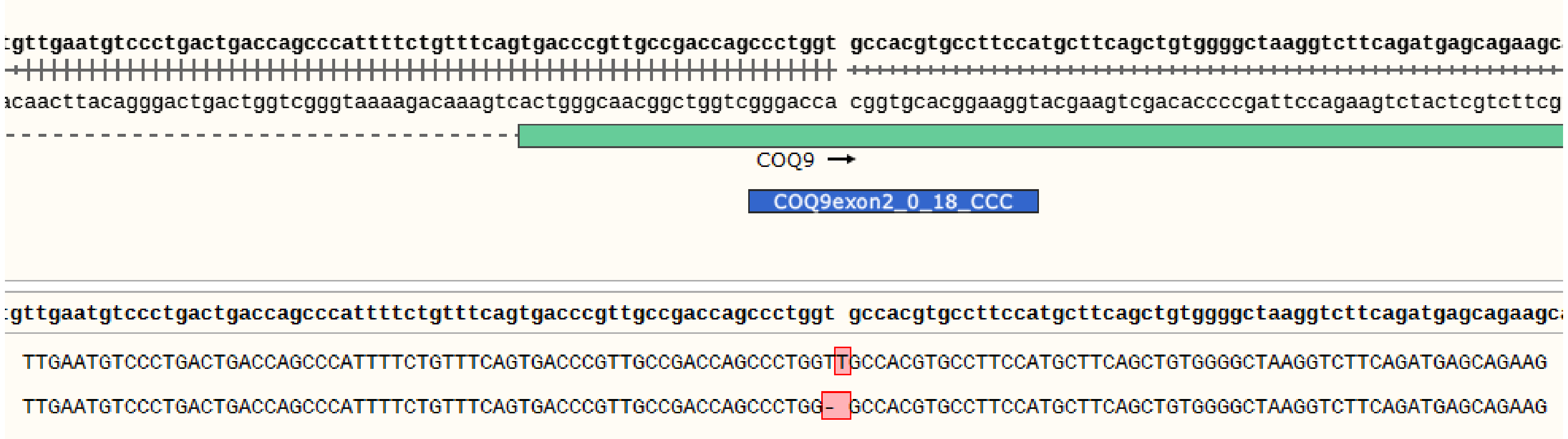

Name	Clone ID	Total # reads	# wt reads(%)	#1-Indel	#1-Reads(%)	#2-Indel	#2-Reads(%)
EPCOQ DKO data EP300 1-G7		1342	0 (0.0%)	-20	686 (51.1%)	-28	615 (45.8%)
EPCOQ DKO data COQ9 1-G7	002D	849	0 (0.0%)	1	525 (61.8%)	-1	314 (37.0%)

Clone ID	Clone ID	Total # reads	# wt reads(%)	#1-Indel	#1-Reads(%)	#2-Indel	#2-Reads(%)
EPCOQ DKO data EP300 1-G7		2734	2 (0.1%)	-20	1403 (51.3%)	-28	1261 (46.1%)
EPCOQ DKO data COQ9 1-G7	002D	2164	0 (0.0%)	-1	1093 (50.5%)	1	999 (46.2%)

Clone 004D (-16/+1 EP300; -5/+1 COQ9)

EP300: NGS 1, NGS 2 (cell bank)
COQ9: NGS 1, NGS 2 (cell bank)

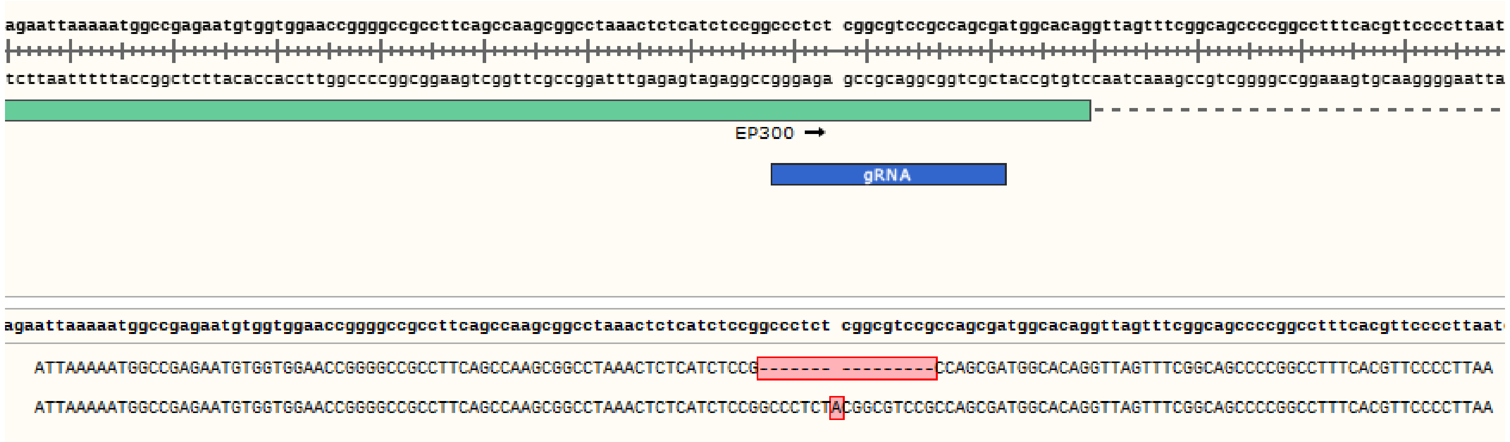

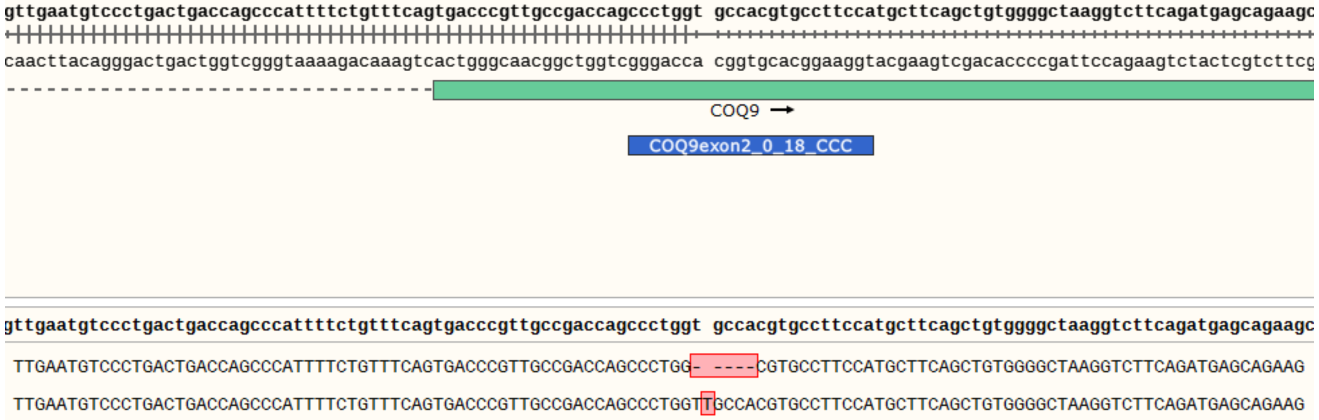

Name	Clone ID	Total # reads	# wt reads(%)	#1-Indel	#1-Reads(%)	#2-Indel	#2-Reads(%)
EPCOQ DKO data EP300 2-C5		2663	0 (0.0%)	-16	1460 (54.8%)	1	1178 (44.2%)
EPCOQ DKO data COQ9 2-C5	004D	1310	0 (0.0%)	-5	700 (53.4%)	1	604 (46.1%)

Clone ID	Clone ID	Total # reads	# wt reads(%)	#1-Indel	#1-Reads(%)	#2-Indel	#2-Reads(%)
EPCOQ DKO data EP300 2-C5		1288	0 (0.0%)	1	541 (42.0%)	-16	525 (40.8%)
EPCOQ DKO data COQ9 2-C5	004D	1740	0 (0.0%)	-5	867 (49.8%)	1	729 (41.9%)

Clone 015D (-31/-5 EP300; +1/+1 COQ9)

EP300: NGS 1, NGS 2 (cell bank)
COQ9: NGS 1, direct sequencing, NGS 2 (cell bank)

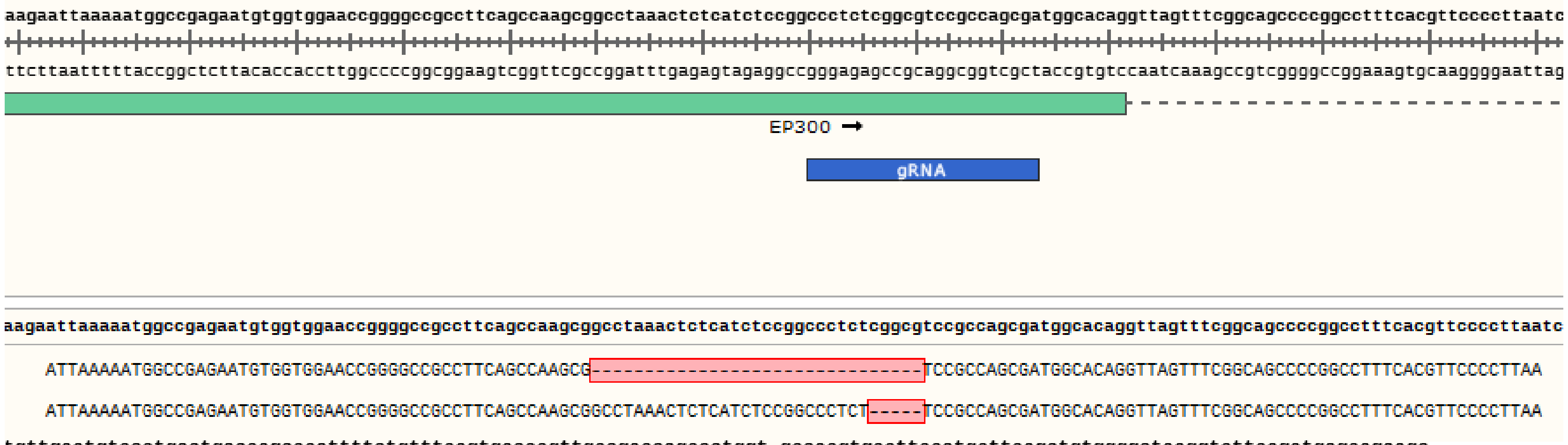

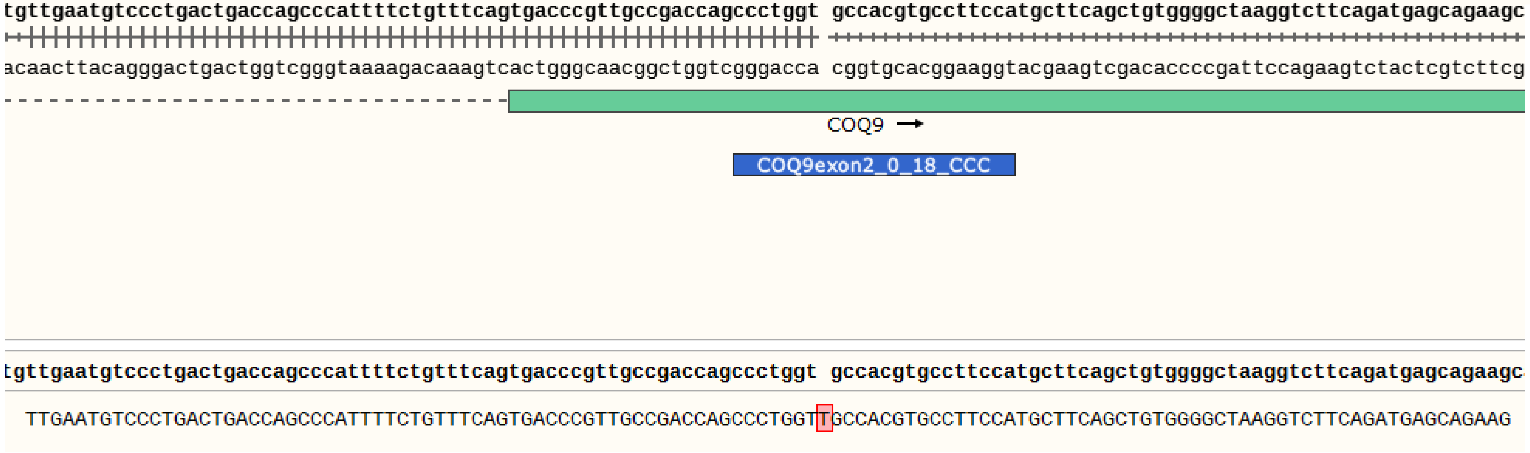

Name	Clone ID	Total # reads	# wt reads(%)	#1-Indel	#1-Reads(%)	#2-Indel	#2-Reads(%)
EPCOQ DKO data EP300 1B6		1295	0 (0.0%)	-31	739 (57.1%)	-5	552 (42.6%)
EPCOQ DKO data COQ9 1B6	015D	1278	0 (0.0%)	1	1253 (98.0%)	0	23 (1.8%)

Clone 015D (-31/-5 EP300; +1/+1 COQ9)

EP300: NGS 1, NGS 2 (cell bank)
COQ9: NGS 1, direct sequencing, NGS 2 (cell bank)

SNP T/A

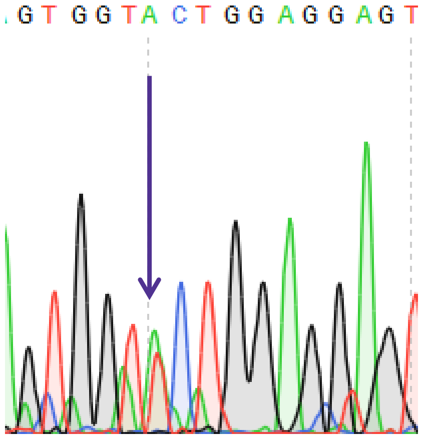

Clone ID	Clone ID	Total # reads	# wt reads(%)	#1-Indel	#1-Reads(%)	#2-Indel	#2-Reads(%)
EPCOQ DKO data EP300 1B6		2718	0 (0.0%)	-31	1512 (55.6%)	-5	1180 (43.4%)
EPCOQ DKO data COQ9 1B6	015D	2734	1 (0.0%)	1	2633 (96.3%)	0	87 (3.2%)

Clone 016D (-11/-149 EP300; +1/+1 COQ9)

EP300: NGS 1, fragment analysis, topo sequencing, NGS 2 (cell bank)
COQ9: NGS 1, direct sequencing, NGS 2 (cell bank)

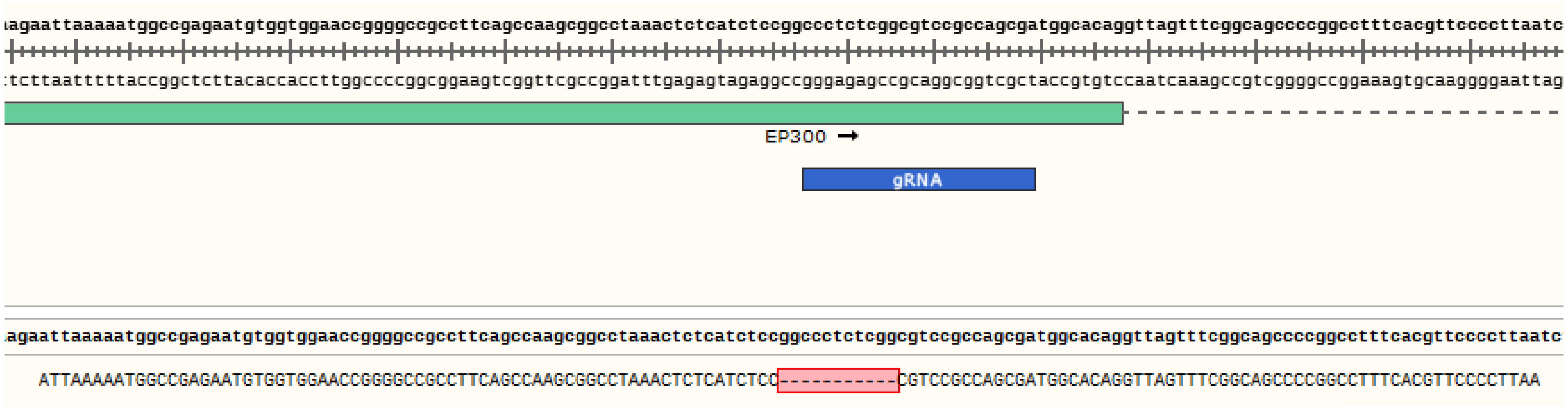

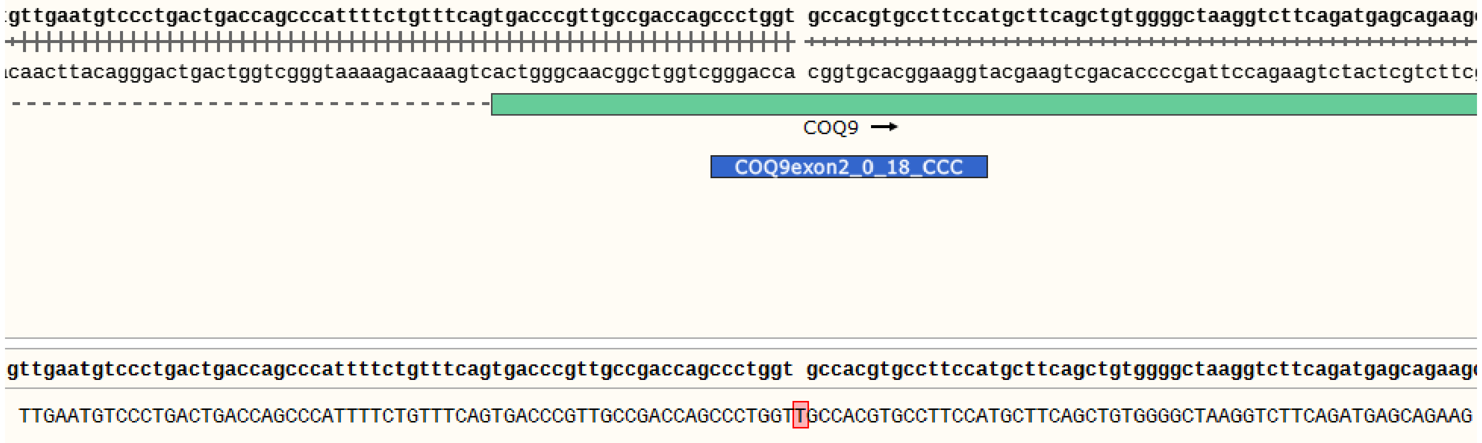

Name	Clone ID	Total # reads	# wt reads(%)	#1-Indel	#1-Reads(%)	#2-Indel	#2-Reads(%)
EPCOQ DKO data EP300 1D11		673	0 (0.0%)	-11	668 (99.3%)	-12	5 (0.7%)
EPCOQ DKO data COQ9 1D11	016D	1903	1 (0.1%)	1	1865 (98.0%)	0	36 (1.9%)

Clone 016D (-11/-149 EP300; +1/+1 COQ9)

EP300: NGS 1, fragment analysis, topo sequencing, NGS 2 (cell bank)
COQ9: NGS 1, direct sequencing, NGS 2 (cell bank)

EP300 fragment analysis -149/-11

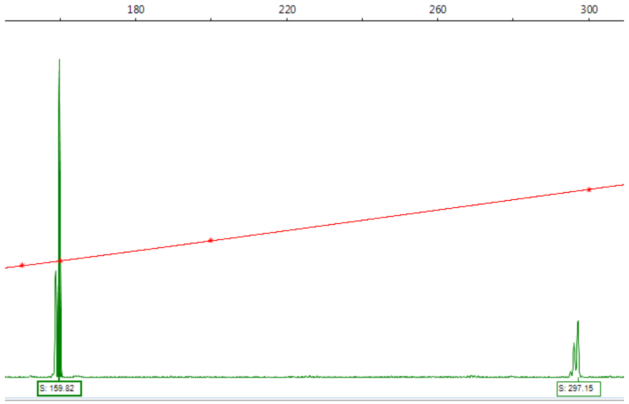

EP300 topo -149/-11

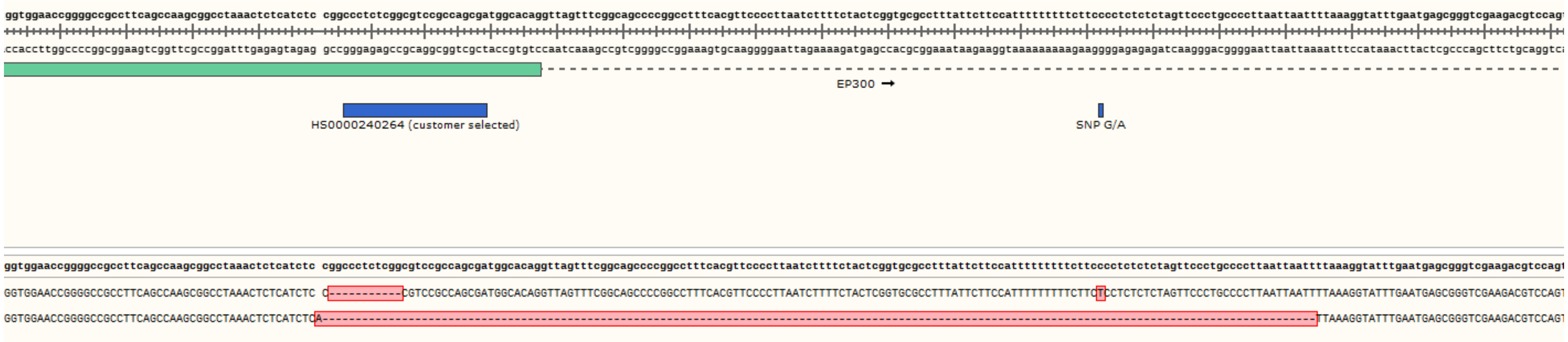

Clone 016D (-11/-149 EP300; +1/+1 COQ9)

EP300: NGS 1, fragment analysis, topo sequencing, NGS 2 (cell bank)
COQ9: NGS 1, direct sequencing, NGS 2 (cell bank)

COQ9 direct sequencing

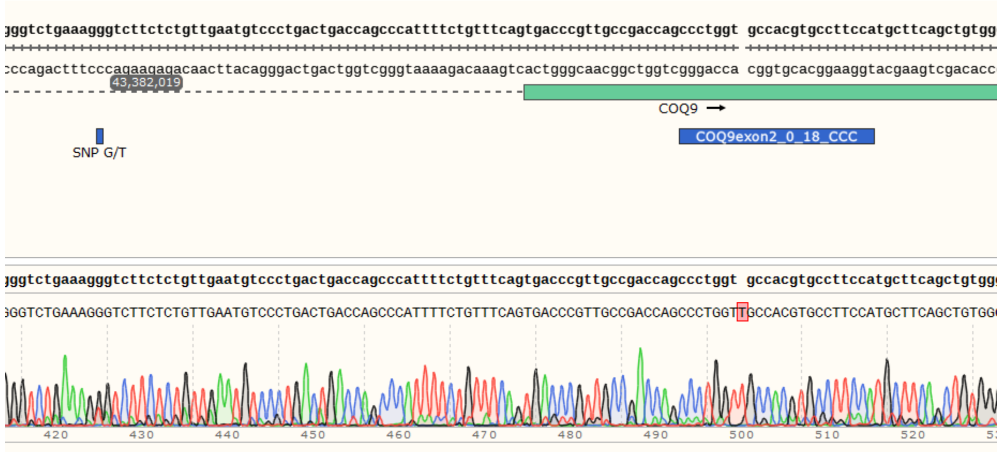

SNP T/A

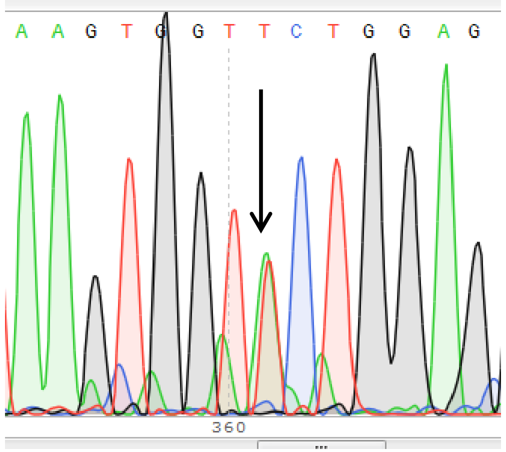

Clone ID	Clone ID	Total # reads	# wt reads(%)	#1-Indel	#1-Reads(%)	#2-Indel	#2-Reads(%)
EPCOQ DKO data EP300 1D11		2229	0 (0.0%)	-11	2198 (98.6%)	-12	28 (1.3%)
EPCOQ DKO data COQ9 1D11	016D	2173	1 (0.0%)	1	2086 (96.0%)	0	72 (3.3%)

Clone 074D (-32/-14 EP300; -2/+1 COQ9)

EP300: NGS 1, NGS 2 (cell bank)
COQ9: NGS 1, NGS 2 (cell bank)

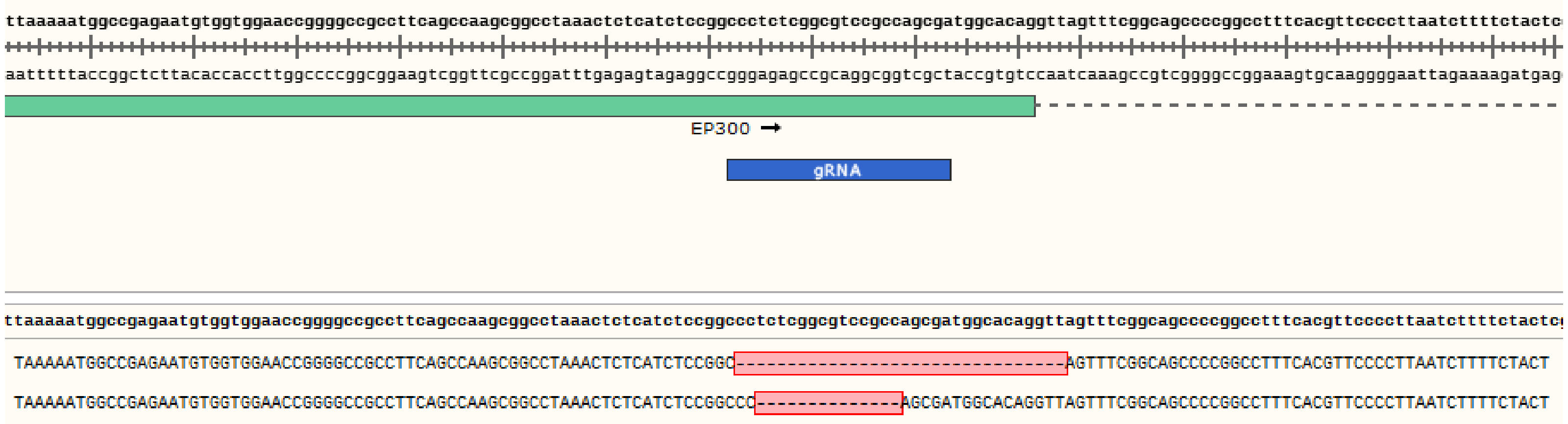

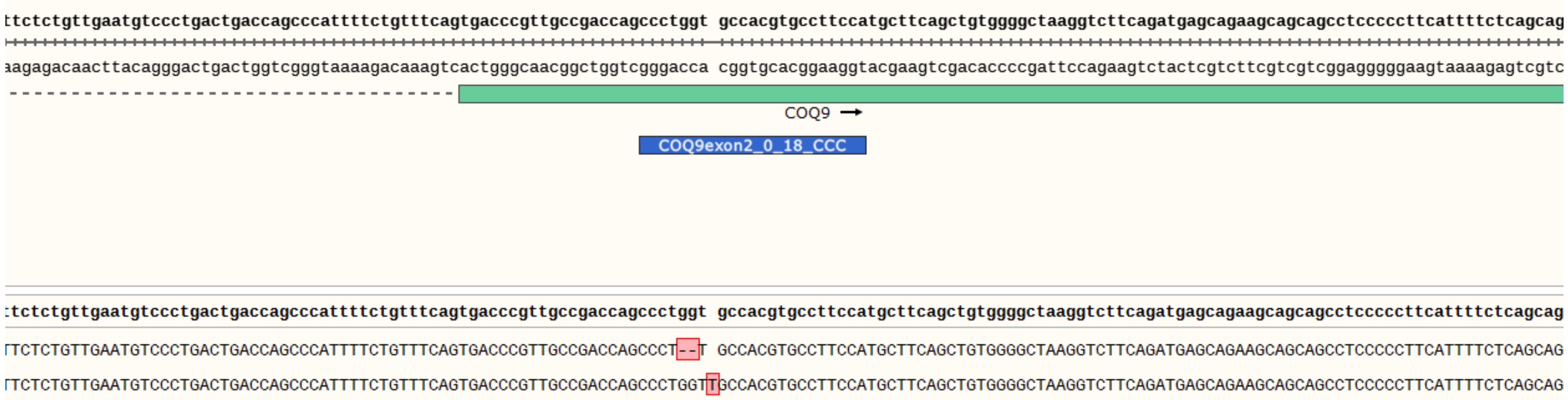

Name	Clone ID	Total # reads	# wt reads(%)	#1-Indel	#1-Reads(%)	#2-Indel	#2-Reads(%)
EPCOQ DKO data EP300 1B4		3932	0 (0.0%)	-32	2010 (51.1%)	-14	1891 (48.1%)
EPCOQ DKO data COQ9 1B4	074D	1834	0 (0.0%)	-2	921 (50.2%)	1	844 (46.0%)

Clone ID	Clone ID	Total # reads	# wt reads(%)	#1-Indel	#1-Reads(%)	#2-Indel	#2-Reads(%)
EPCOQ DKO data EP300 1B4		10606	1 (0.0%)	-32	5638 (53.2%)	-14	4823 (45.5%)
EPCOQ DKO data COQ9 1B4	074D	5110	0 (0.0%)	-2	2478 (48.5%)	1	2474 (48.4%)

Clone 075D (-7/+1 EP300; -19/+1 COQ9)

EP300: NGS 1, NGS 2 (cell bank) WT contamination revealed
COQ9: NGS 1, NGS 2 (cell bank)

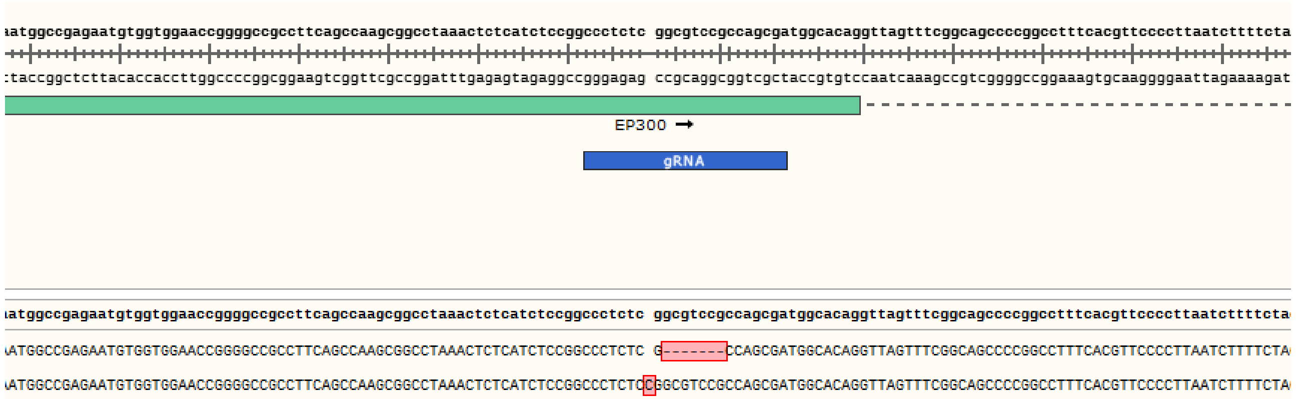

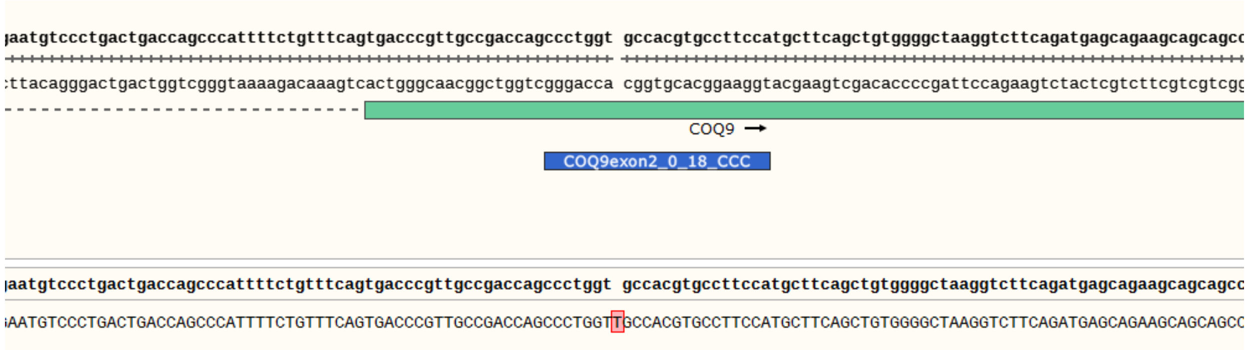

Name	Clone ID	Total # reads	# wt reads(%)	#1-Indel	#1-Reads(%)	#2-Indel	#2-Reads(%)
EPCOQ DKO data EP300 1D5		2812	4 (0.1%)	-7	1433 (51.0%)	1	1346 (47.9%)
EPCOQ DKO data COQ9 1D5	075D	2267	0 (0.0%)	-19	1232 (54.3%)	1	945 (41.7%)

Clone ID	Clone ID	Total # reads	# wt reads(%)	#1-Indel	#1-Reads(%)	#2-Indel	#2-Reads(%)
EPCOQ DKO data EP300 1D5		13476	7966 (59.1%)	0	8304 (61.6%)	1	2637 (19.6%)
EPCOQ DKO data COQ9 1D5	075D	4447	1 (0.0%)	1	3653 (82.1%)	-19	647 (14.5%)

Clone 076D (-8/-1 EP300; -23/-8 COQ9)

EP300: NGS 1, NGS 2 (cell bank)
COQ9: NGS 1, NGS 2 (cell bank)

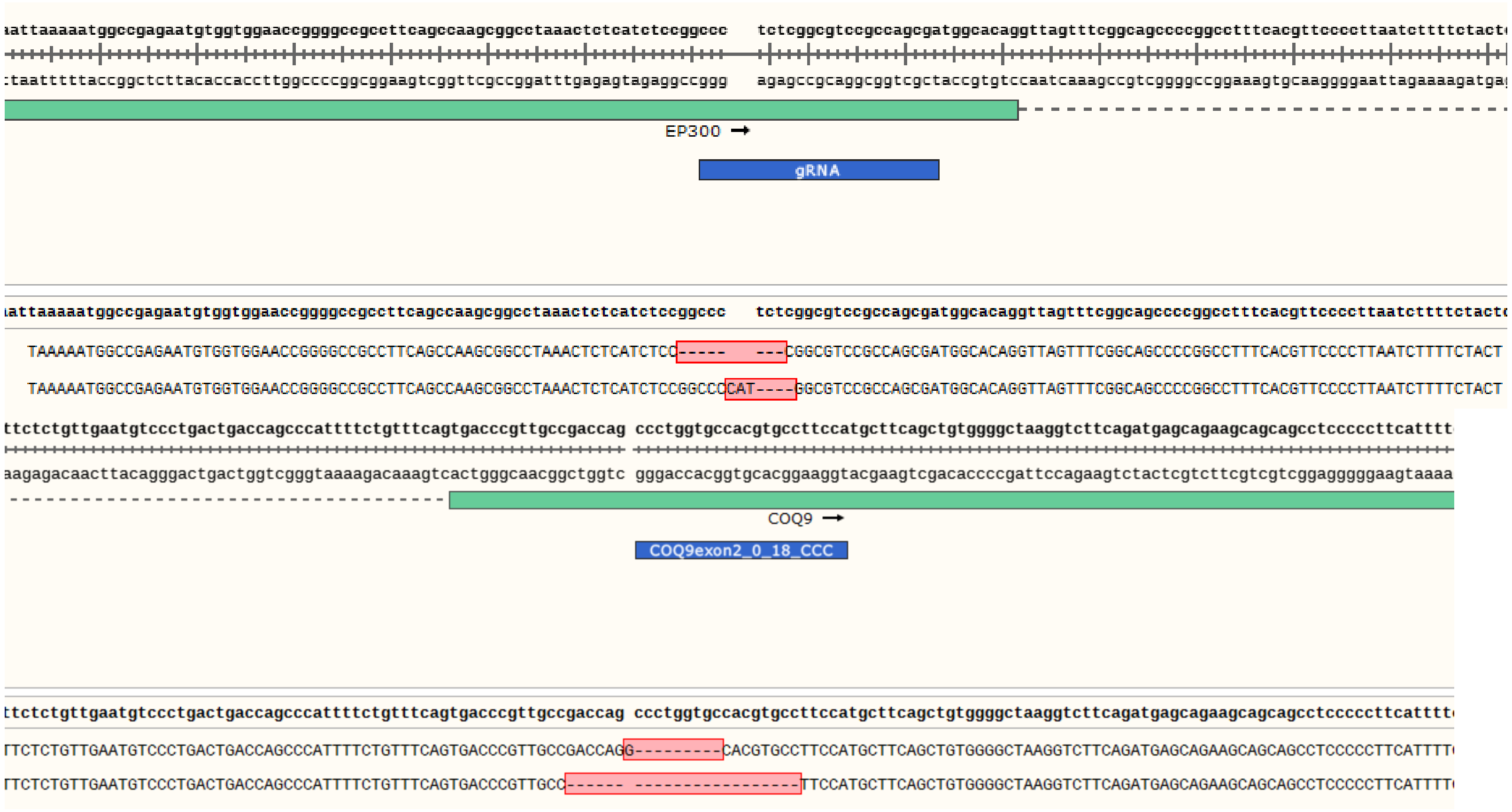

Name	Clone ID	Total # reads	# wt reads(%)	#1-Indel	#1-Reads(%)	#2-Indel	#2-Reads(%)
EPCOQ DKO data EP300 1F12		2486	2 (0.1%)	-8	1266 (50.9%)	-1	1156 (46.5%)
EPCOQ DKO data COQ9 1F12	076D	1528	0 (0.0%)	-23	745 (48.8%)	-8	741 (48.5%)

Clone ID	Clone ID	Total # reads	# wt reads(%)	#1-Indel	#1-Reads(%)	#2-Indel	#2-Reads(%)
EPCOQ DKO data EP300 1F12		14142	36 (0.3%)	-1	6954 (49.2%)	-8	6937 (49.1%)
EPCOQ DKO data COQ9 1F12	076D	5051	11 (0.2%)	-8	2451 (48.5%)	-23	2405 (47.6%)

Clone 082D (-16/-2 EP300; +1/+1 COQ9)

EP300: NGS 1, NGS 2 (cell bank)
COQ9: NGS 1, direct sequencing, NGS 2 (cell bank)

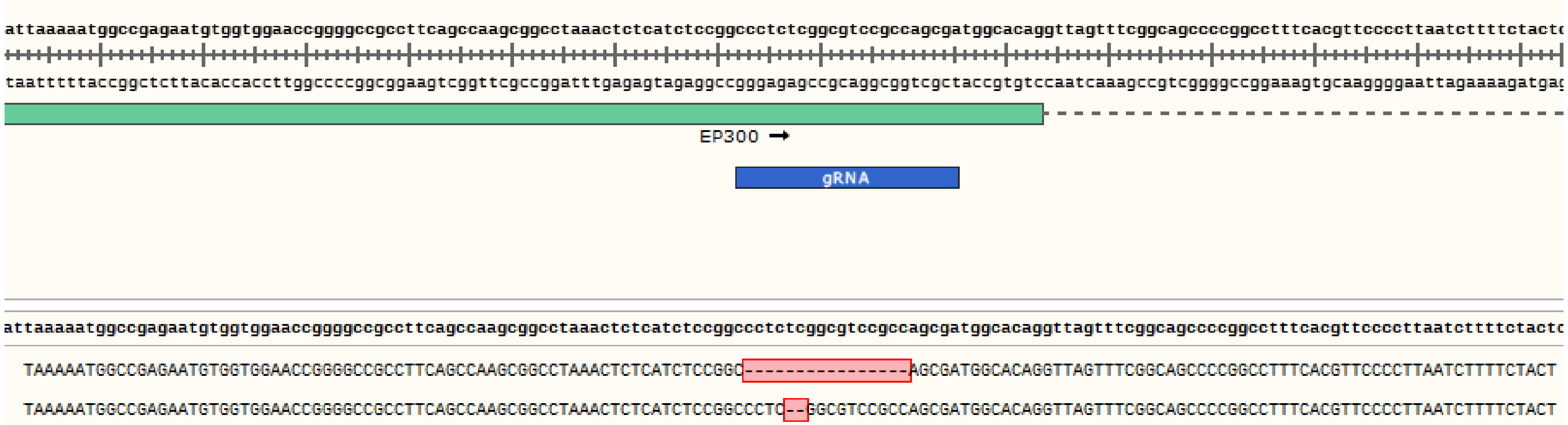

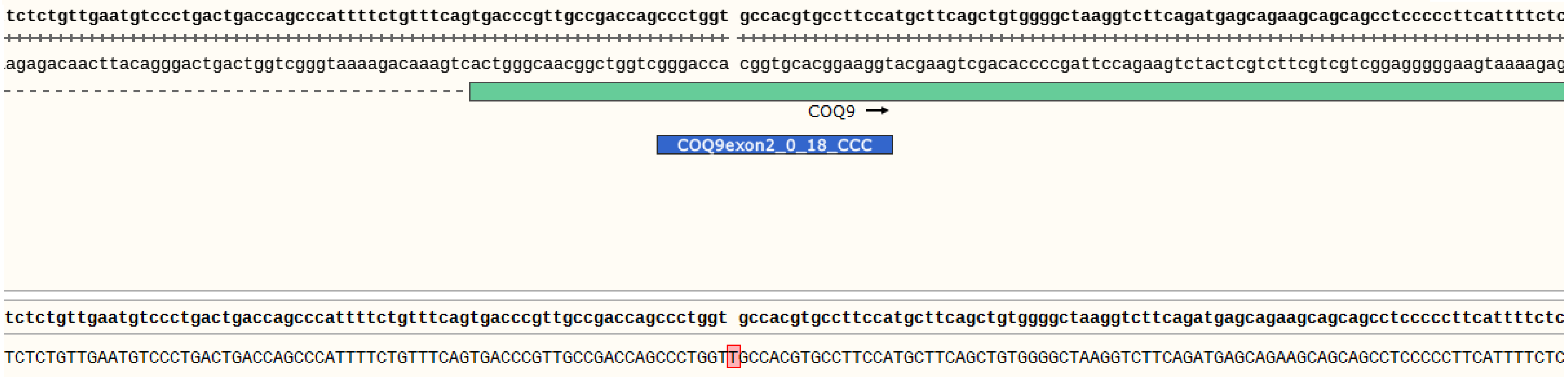

Name	Clone ID	Total # reads	# wt reads(%)	#1-Indel	#1-Reads(%)	#2-Indel	#2-Reads(%)
EPCOQ DKO data EP300 2G2		2384	1 (0.0%)	-16	1234 (51.8%)	-2	1127 (47.3%)
EPCOQ DKO data COQ9 2G2	082D	2346	1 (0.0%)	1	2265 (96.5%)	0	61 (2.6%)

Clone 082D (-16/-2 EP300; +1/+1 COQ9)

EP300: NGS 1, NGS 2 (cell bank)
COQ9: NGS 1, direct sequencing, NGS 2 (cell bank)

SNP T/A

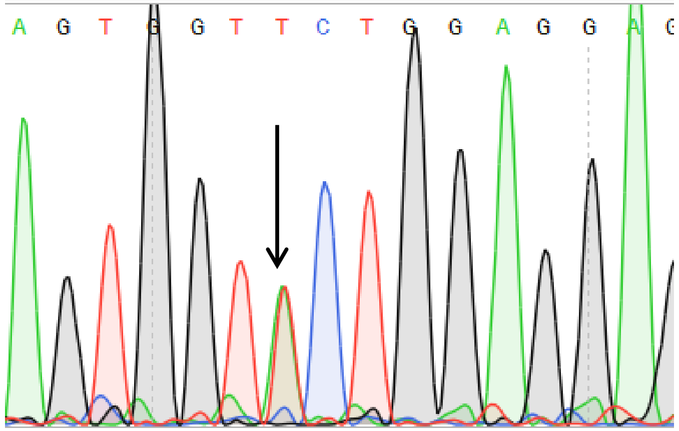

Clone ID	Clone ID	Total # reads	# wt reads(%)	#1-Indel	#1-Reads(%)	#2-Indel	#2-Reads(%)
EPCOQ DKO data EP300 2G2		9916	6 (0.1%)	-16	4977 (50.2%)	-2	4797 (48.4%)
EPCOQ DKO data COQ9 2G2	082D	5336	1 (0.0%)	1	5172 (96.9%)	0	135 (2.5%)

Clone 003D (-17/-61 EP300; -89/-2/+1 NAT9)

EP300: NGS 1, NGS 2 (cell bank)

NAT9: NGS 1, NGS 2 (cell bank)

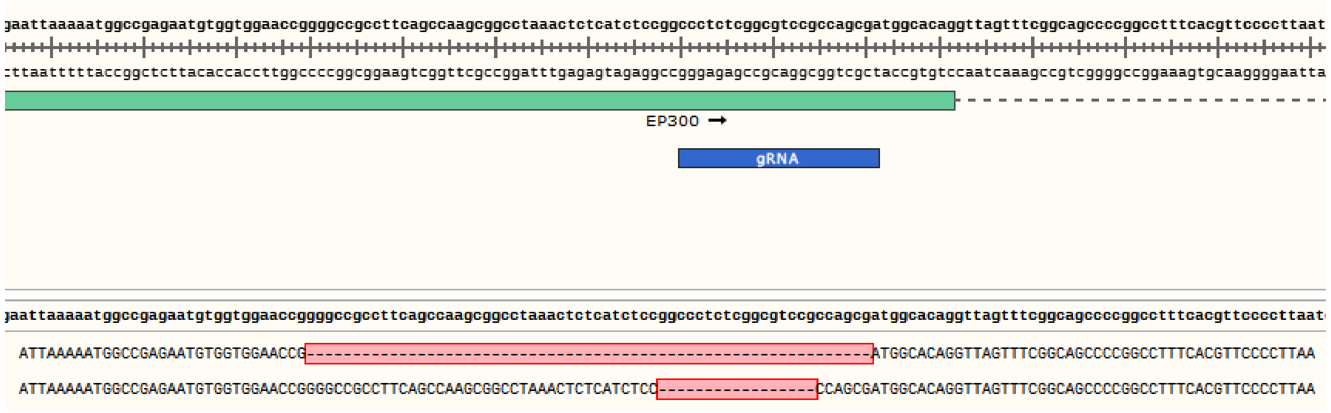

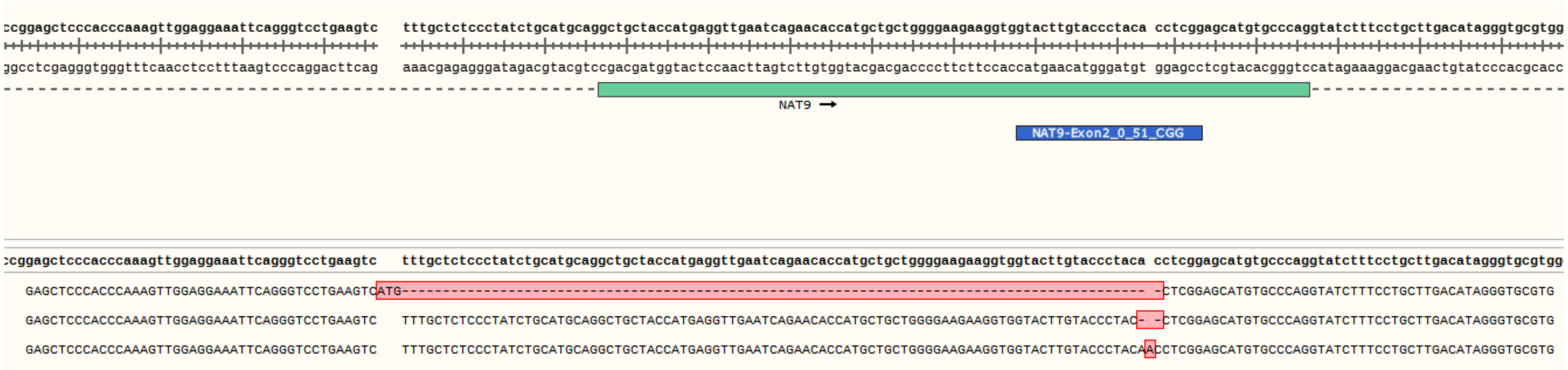

Name	Clone ID	Total # reads	# wt reads(%)	#1-Indel	#1-Reads(%)	#2-Indel	#2-Reads(%)	#3-Indel	#3-Reads(%)
EPNAT DKO data EP300 1-H8		231	0 (0.0%)	-17	124 (53.7%)	-61	107 (46.3%)	NA	
EPNAT DKO data NAT9 1-H8	003D	1413	1 (0.1%)	-89	712 (50.4%)	-2	388 (27.5%)	1	297 (21.0%)

Clone ID	Clone ID	Total # reads	# wt reads(%)	#1-Indel	#1-Reads(%)	#2-Indel	#2-Reads(%)	#3-Indel	#3-Reads(%)
EPNAT DKO data EP300 1-H8		3350	0 (0.0%)	-61	1920 (57.3%)	-17	1380 (41.2%)	-18	22 (0.7%)
EPNAT DKO data NAT9 1-H8	003D	1692	0 (0.0%)	-89	984 (58.2%)	-2	353 (20.9%)	1	325 (19.2%)

Clone 005D (-1/-7 EP300; -1/-7/-2 NAT9)

EP300: NGS 1, NGS 2 (cell bank)
NAT9: NGS 1, NGS 2 (cell bank)

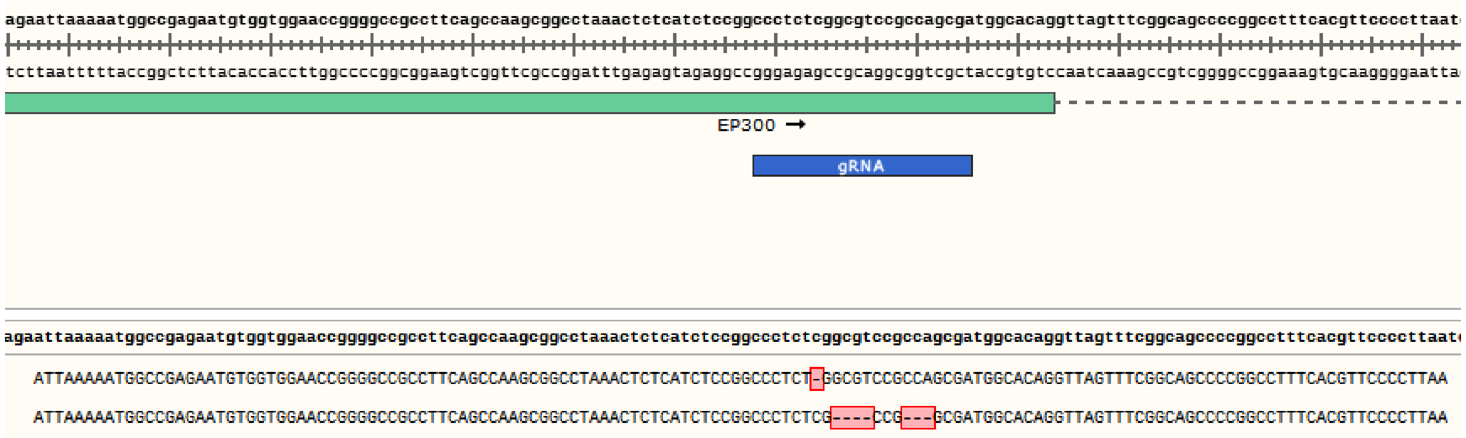

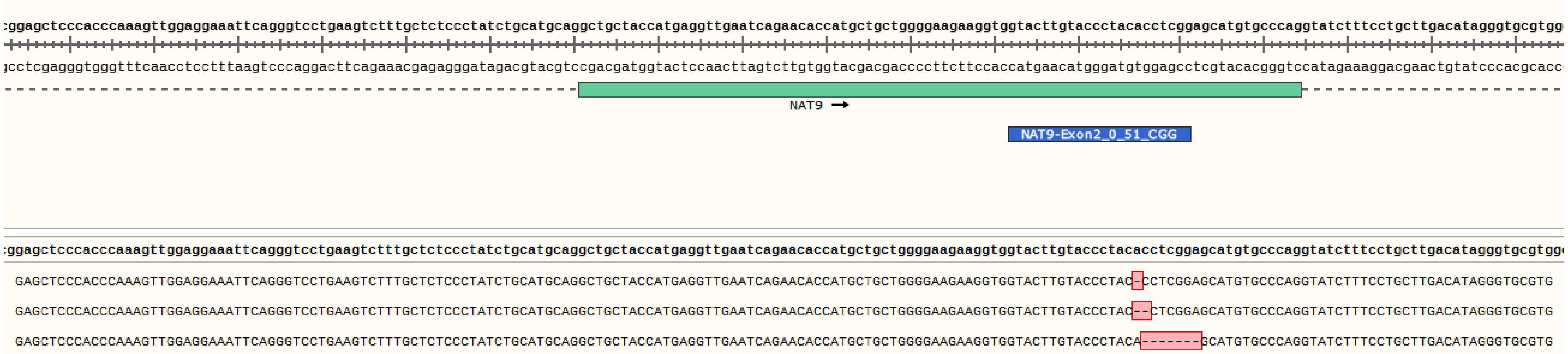

Name	Clone ID	Total # reads	# wt reads(%)	#1-Indel	#1-Reads(%)	#2-Indel	#2-Reads(%)	#3-Indel	#3-Reads(%)
EPNAT DKO data EP300 2-G1		677	0 (0.0%)	-1	430 (63.5%)	-7	238 (35.2%)	-8	5 (0.7%)
EPNAT DKO data NAT9 2-G1	005D	1417	0 (0.0%)	-1	613 (43.3%)	-7	405 (28.6%)	-2	390 (27.5%)

Clone ID	Clone ID	Total # reads	# wt reads(%)	#1-Indel	#1-Reads(%)	#2-Indel	#2-Reads(%)	#3-Indel	#3-Reads(%)
EPNAT DKO data EP300 2-G1		2723	22 (0.8%)	-1	1391 (51.1%)	-7	1227 (45.1%)	-57	36 (1.3%)
EPNAT DKO data NAT9 2-G1	005D	1317	0 (0.0%)	-1	441 (33.5%)	-7	439 (33.3%)	-2	409 (31.1%)

Clone 007D (-16/+11 EP300; -4/+1 NAT9)

EP300: NGS 1, NGS 2 (cell bank)
NAT9: NGS 1, NGS 2 (cell bank)

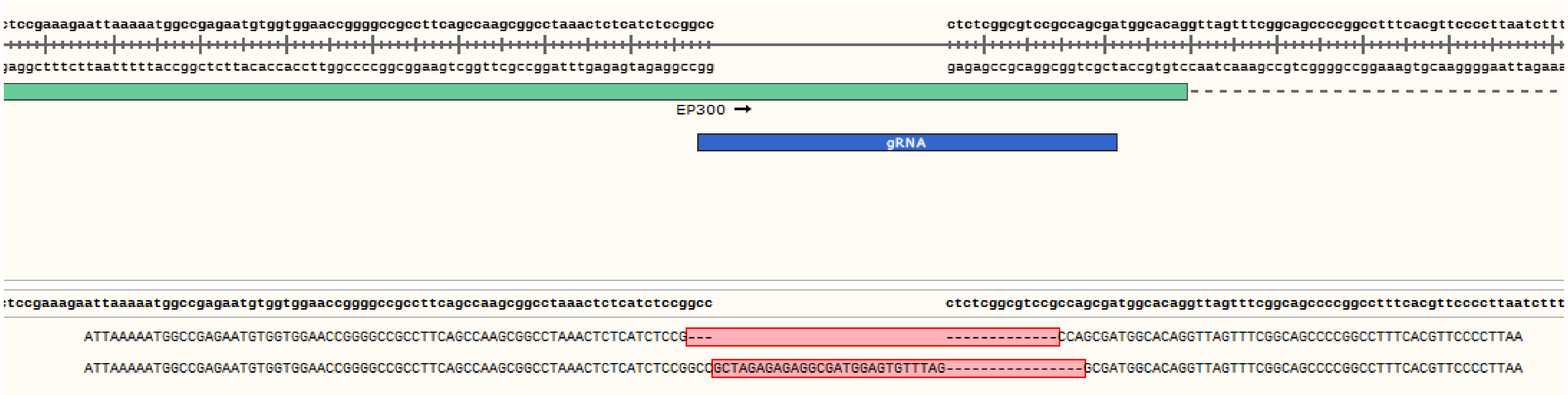

NAT9

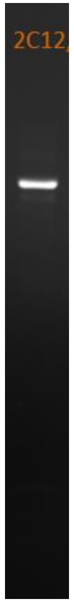

Name	Clone ID	Total # reads	# wt reads(%)	#1-Indel	#1-Reads(%)	#2-Indel	#2-Reads(%)	#3-Indel	#3-Reads(%)
EPNAT DKO data EP300 2C12		393	0 (0.0%)	-16	213 (54.2%)	11	178 (45.3%)	10	1 (0.3%)
EPNAT DKO data NAT9 2C12	007D	1846	2 (0.1%)	-4	950 (51.5%)	1	870 (47.1%)	0	12 (0.7%)

Clone ID	Clone ID	Total # reads	# wt reads(%)	#1-Indel	#1-Reads(%)	#2-Indel	#2-Reads(%)	#3-Indel	#3-Reads(%)
EPNAT DKO data EP300 2C12		3624	3 (0.1%)	-16	1958 (54.0%)	11	1617 (44.6%)	-17	24 (0.7%)
EPNAT DKO data NAT9 2C12	007D	1156	0 (0.0%)	-4	578 (50.0%)	1	555 (48.0%)	0	14 (1.2%)

Clone 008D (-16/-62 EP300; -14/-2/-326 NAT9)

EP300: NGS 1, fragment analysis, topo sequencing, NGS 2 (cell bank)
NAT9: NGS 1, agarose gel, topo sequencing, NGS 2 (cell bank)

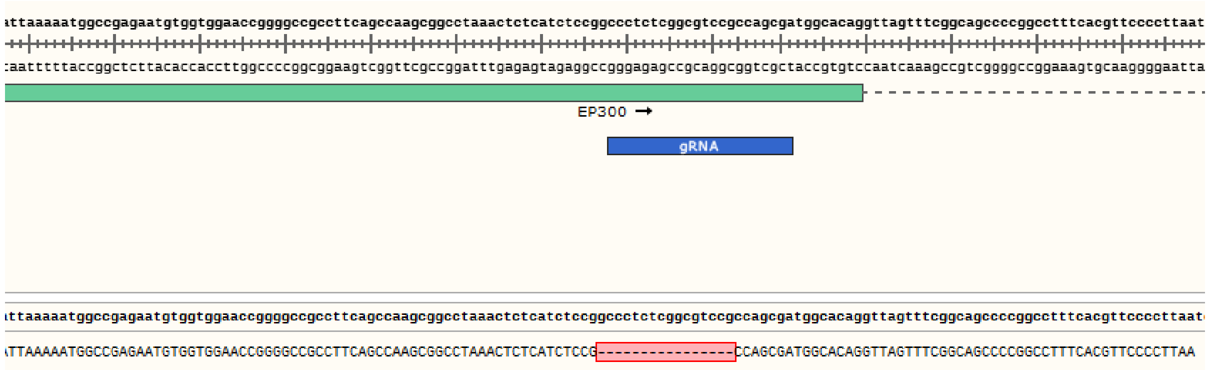

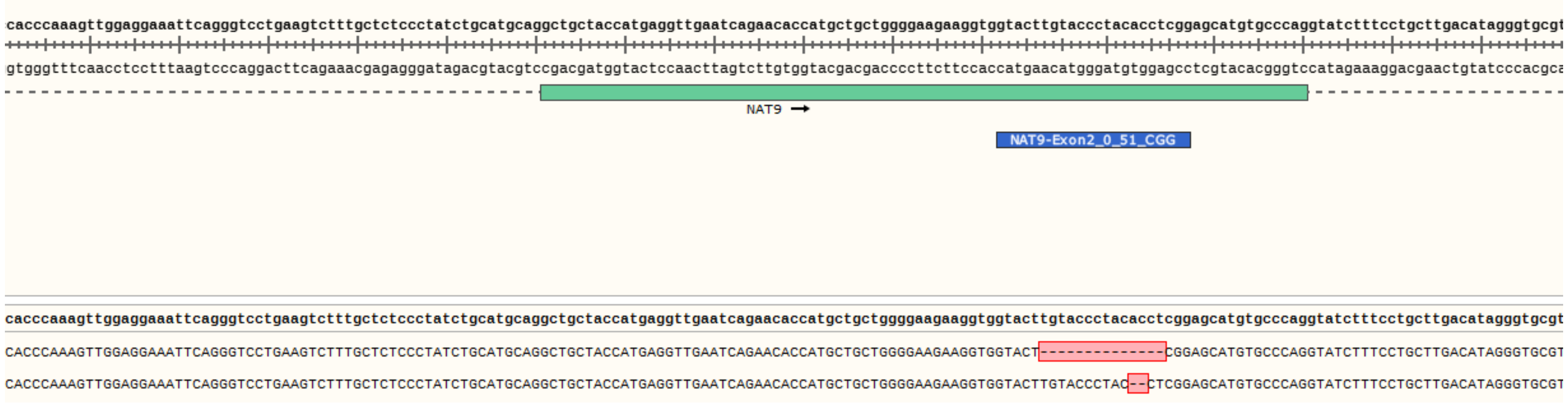

Name	Clone ID	Total # reads	# wt reads(%)	#1-Indel	#1-Reads(%)	#2-Indel	#2-Reads(%)	#3-Indel	#3-Reads(%)
EPNAT DKO data EP300 1C2		1358	2 (0.1%)	-16	1346 (99.1%)	-17	7 (0.5%)	0	2 (0.1%)
EPNAT DKO data NAT9 1C2	008D	2475	1 (0.0%)	-14	1252 (50.6%)	-2	1188 (48.0%)	-3	18 (0.7%)

Clone 008D (-16/-62 EP300; -14/-2/-326 NAT9)

EP300: NGS 1, fragment analysis, topo sequencing, NGS 2 (cell bank)
NAT9: NGS 1, agarose gel, topo sequencing, NGS 2 (cell bank)

EP300 fragment analysis -16/-62

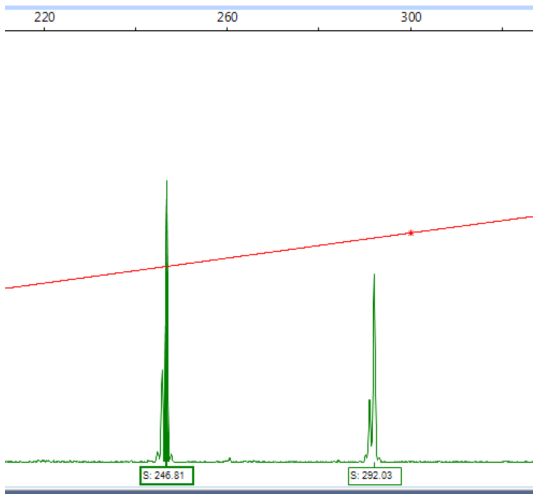

EP300 topo sequencing -16/-62

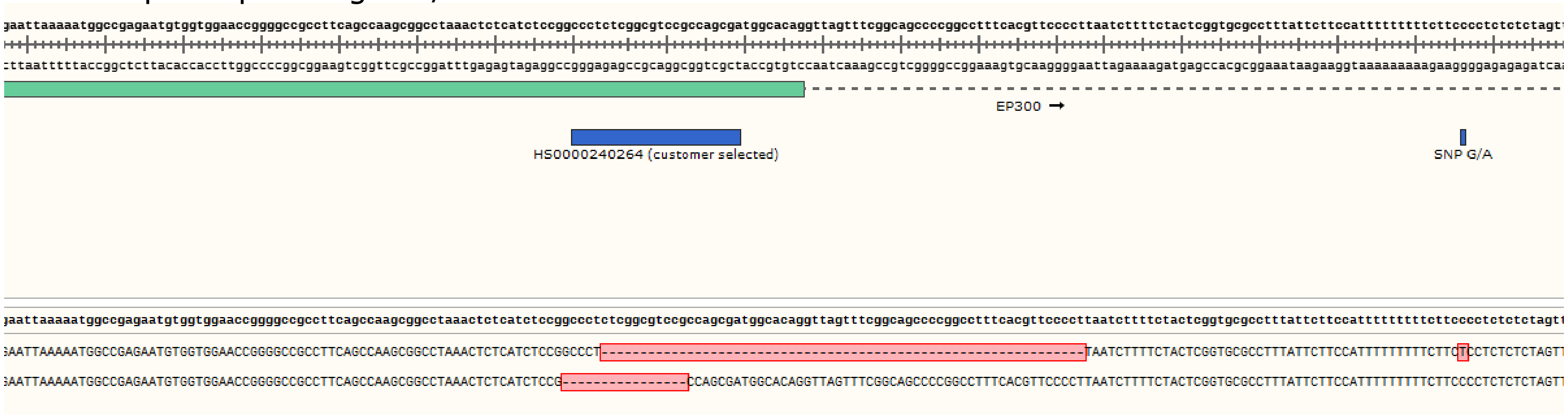

Clone 008D (-16/-62 EP300; -14/-2/-326 NAT9)

EP300: NGS 1, fragment analysis, topo sequencing, NGS 2 (cell bank)
NAT9: NGS 1, agarose gel, topo sequencing, NGS 2 (cell bank)

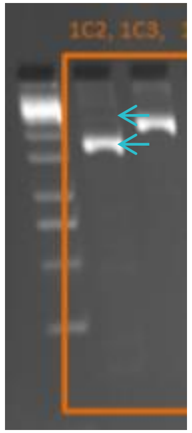

Agarose gel NAT9- smaller band indicates large deletion

Topo NAT9 deleted sequence -326bp:

accctacacctcggagcatgtgcccaggtatctttcctgcttgacatagggtgcgtggcagcatccagagctgtgtaaggtacagccccagtctcaggg
ctgtttcccctaagaagaaacattggttgcgagaaataactacatatgagggggaaaaaaaaggctgggtaccgtggctcacgcctgtaatcccag
gactttgggaggctgtggcaggaggatcgcttgagcccaggagttcgagaccagcttgggtaacgtagcgagacctcgtctctacaaaaaatataaa
aattagtcaggcatggtggtgcacagctgtagt

Clone 008D (-16/-62 EP300; -14/-2/-326 NAT9)

EP300: NGS 1, fragment analysis, topo sequencing, NGS 2 (cell bank)
NAT9: NGS 1, agarose gel, topo sequencing, NGS 2 (cell bank)

Topo sequence alignment NAT9

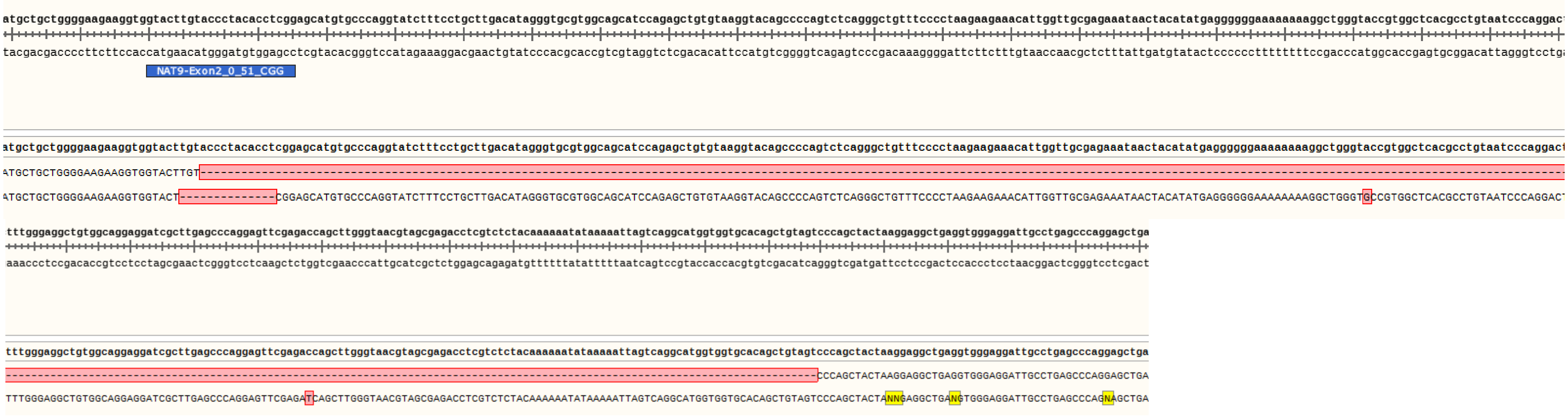

Clone ID	Clone ID	Total # reads	# wt reads(%)	#1-Indel	#1-Reads(%)	#2-Indel	#2-Reads(%)	#3-Indel	#3-Reads(%)
EPNAT DKO data EP300 1C2		3119	0 (0.0%)	-62	1628 (52.2%)	-16	1451 (46.5%)	-17	19 (0.6%)
EPNAT DKO data NAT9 1C2	008D	1556	0 (0.0%)	-14	783 (50.3%)	-2	744 (47.8%)	-3	14 (0.9%)

Clone 071D (-4/-17 EP300; -4/-2/-1 NAT9)

EP300: NGS 1, NGS 2 (cell bank)
NAT9: NGS 1, NGS 2 (cell bank)

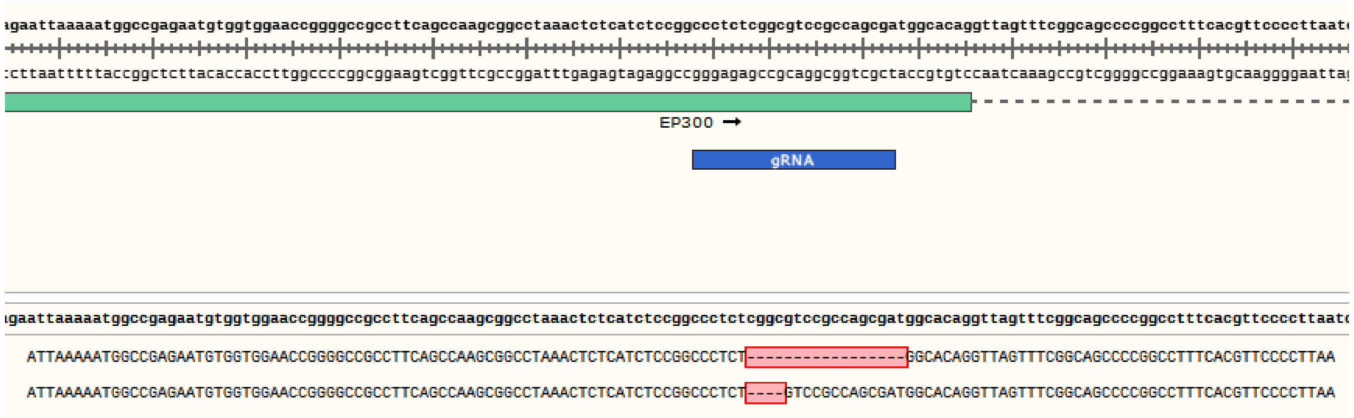

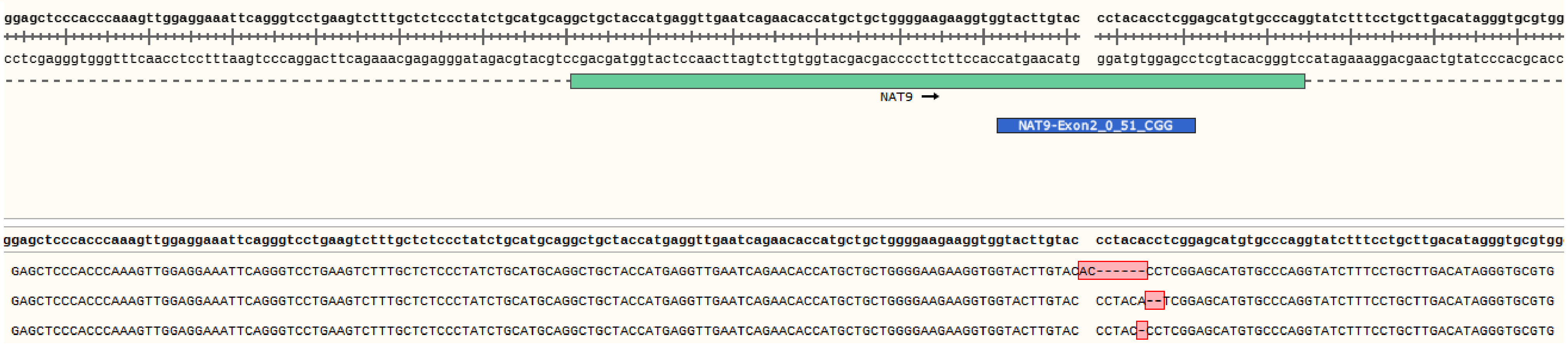

Name	Clone ID	Total # reads	# wt reads(%)	#1-Indel	#1-Reads(%)	#2-Indel	#2-Reads(%)	#3-Indel	#3-Reads(%)
EPNAT DKO data EP300 1E11		6020	1 (0.0%)	-4	3017 (50.1%)	-17	2904 (48.2%)	-18	43 (0.7%)
EPNAT DKO data NAT9 1E11	071D	5769	1 (0.0%)	-4	1992 (34.5%)	-2	1879 (32.6%)	-1	1834 (31.8%)

Clone ID	Clone ID	Total # reads	# wt reads(%)	#1-Indel	#1-Reads(%)	#2-Indel	#2-Reads(%)	#3-Indel	#3-Reads(%)
EPNAT DKO data EP300 1E11		11131	47 (0.4%)	-17	5601 (50.3%)	-4	5315 (47.7%)	-18	80 (0.7%)
EPNAT DKO data NAT9 1E11	071D	7461	0 (0.0%)	-2	2508 (33.6%)	-1	2401 (32.2%)	-4	2400 (32.2%)

Clone 072D (-41/-101 EP300; -1/-1/-22 NAT9)

EP300: NGS 1, PCR agarose gel, topo sequencing, NGS 2 (cell bank)
NAT9: NGS 1, NGS 2 (cell bank)

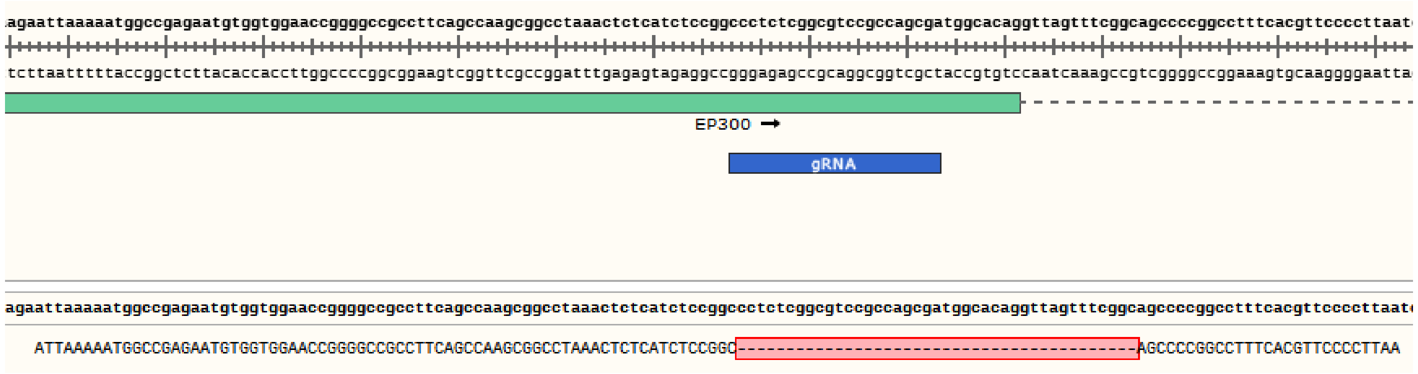

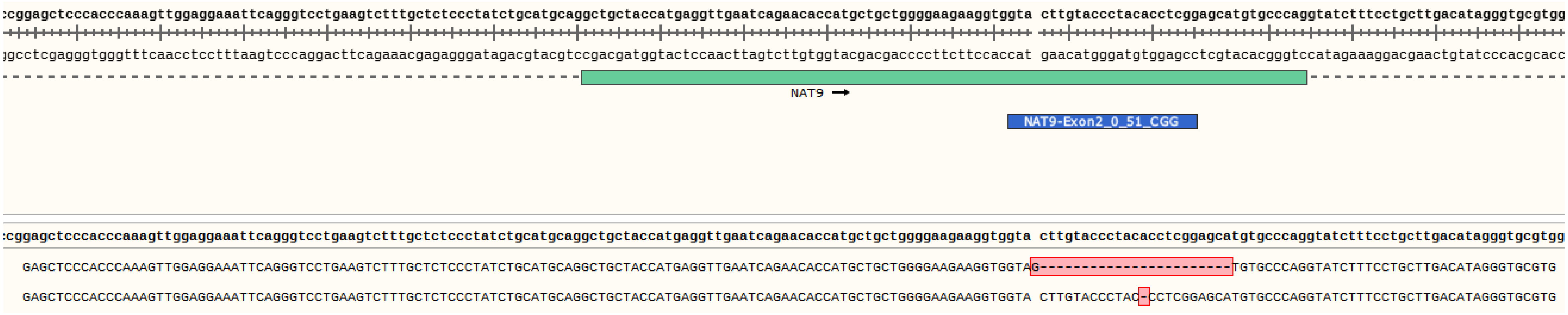

Name	Clone ID	Total # reads	# wt reads(%)	#1-Indel	#1-Reads(%)	#2-Indel	#2-Reads(%)	#3-Indel	#3-Reads(%)
EPNAT DKO data EP300 1F7		10200	15 (0.1%)	-41	10052 (98.5%)	-42	80 (0.8%)	-12	27 (0.3%)
EPNAT DKO data NAT9 1F7	072D	6818	1 (0.0%)	-1	4231 (62.1%)	-22	2478 (36.3%)	-2	59 (0.9%)

Clone 072D (-41/-101 EP300; -1/-1/-22 NAT9)

EP300: NGS 1, fragment analysis, topo sequencing, NGS 2 (cell bank)
NAT9: NGS 1, NGS 2 (cell bank)

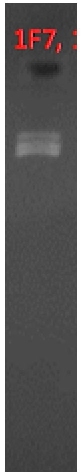

EP300 2 bands

EP300 topo sequencing -41/-101

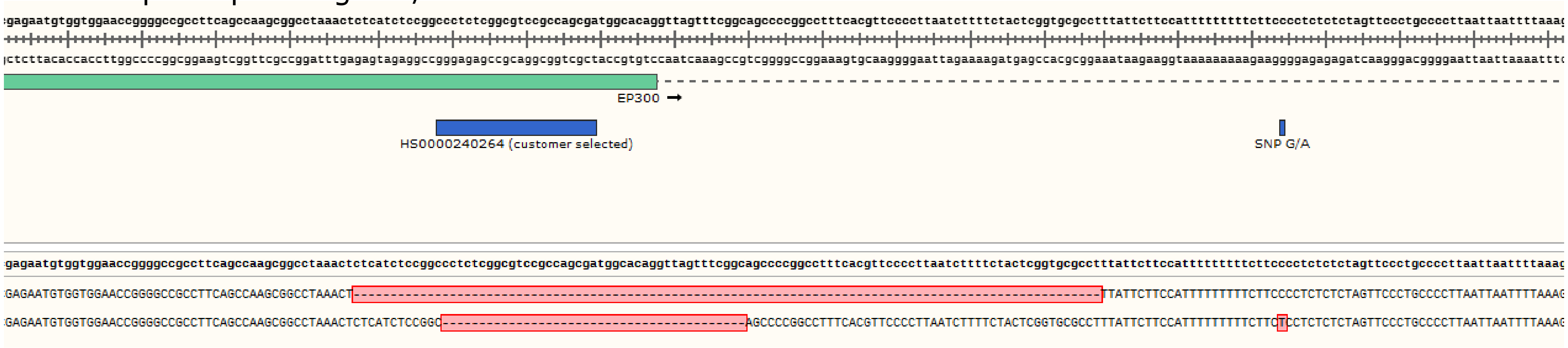

Clone ID	Clone ID	Total # reads	# wt reads(%)	#1-Indel	#1-Reads(%)	#2-Indel	#2-Reads(%)	#3-Indel	#3-Reads(%)
EPNAT DKO data EP300 1F7		12930	0 (0.0%)	-41	12754 (98.6%)	-42	146 (1.1%)	-1	10 (0.1%)
EPNAT DKO data NAT9 1F7	072D	5888	0 (0.0%)	-1	3781 (64.2%)	-22	1973 (33.5%)	-2	82 (1.4%)

Clone 073D (-5/-187 EP300; -1/-1/+1 NAT9)

EP300: NGS 1, topo sequencing, NGS 2 (cell bank)
NAT9: NGS 1, NGS 2 (cell bank)

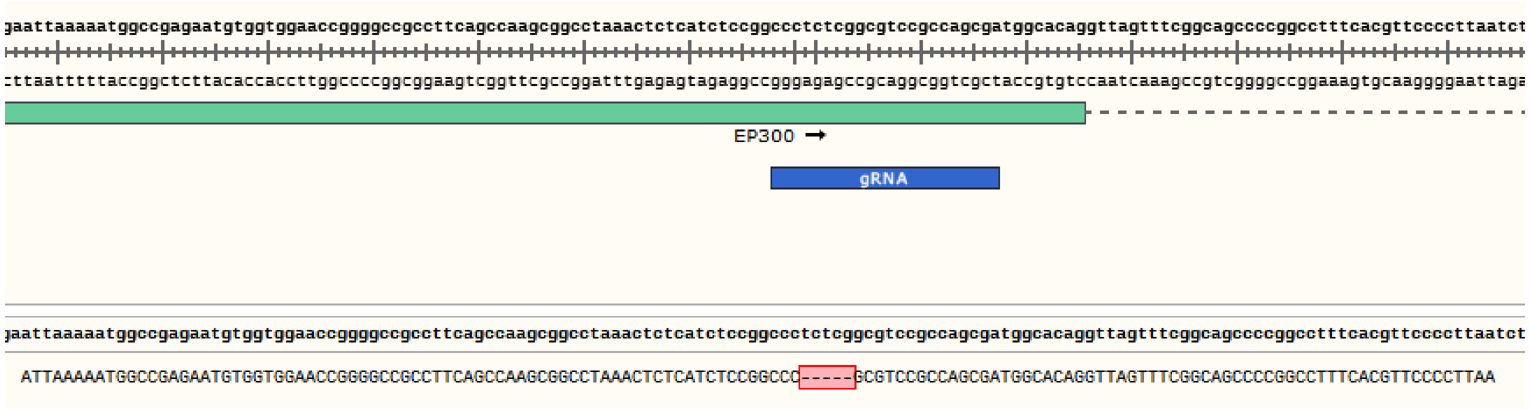

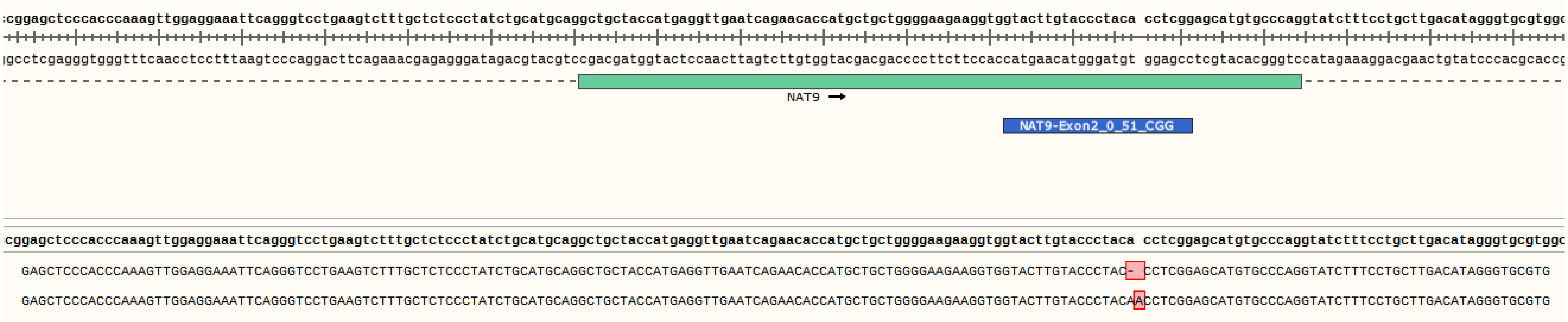

Name	Clone ID	Total # reads	# wt reads(%)	#1-Indel	#1-Reads(%)	#2-Indel	#2-Reads(%)	#3-Indel	#3-Reads(%)
EPNAT DKO data EP300 1H11		5832	14 (0.2%)	-5	5643 (96.8%)	-6	81 (1.4%)	-47	32 (0.5%)
EPNAT DKO data NAT9 1H11	073D	7925	0 (0.0%)	-1	5228 (66.0%)	1	2594 (32.7%)	-2	65 (0.8%)

Clone 073D (-5/-187 EP300; -1/-1/+1 NAT9)

EP300: NGS 1, topo sequencing, NGS 2 (cell bank)
NAT9: NGS 1, NGS 2 (cell bank)

EP300 topo sequencing -5/-187

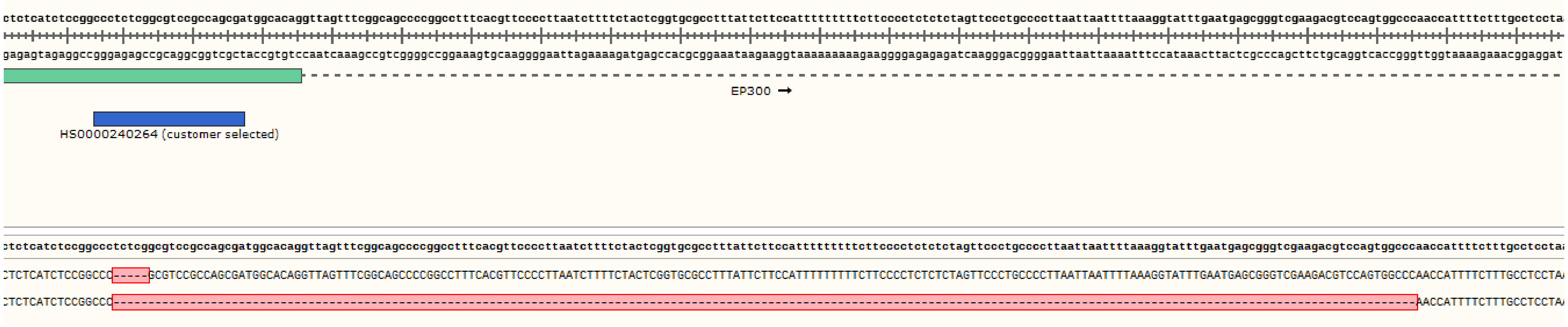

Clone ID	Clone ID	Total # reads	# wt reads(%)	#1-Indel	#1-Reads(%)	#2-Indel	#2-Reads(%)	#3-Indel	#3-Reads(%)
EPNAT DKO data EP300 1H11		9973	0 (0.0%)	-5	9838 (98.6%)	-6	121 (1.2%)	-7	4 (0.0%)
EPNAT DKO data NAT9 1H11	073D	7246	0 (0.0%)	-1	4615 (63.7%)	1	2475 (34.2%)	-2	90 (1.2%)

Clone 079D (-16/+13 EP300; -38/-2/+1 NAT9)

EP300: NGS 1, NGS 2 (cell bank)
NAT9: NGS 1, NGS 2 (cell bank)

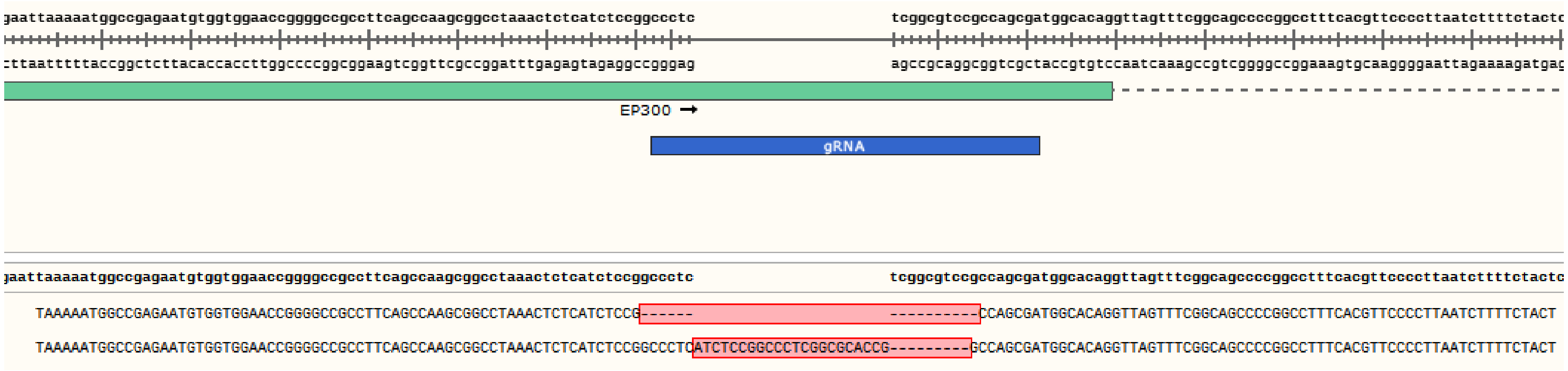

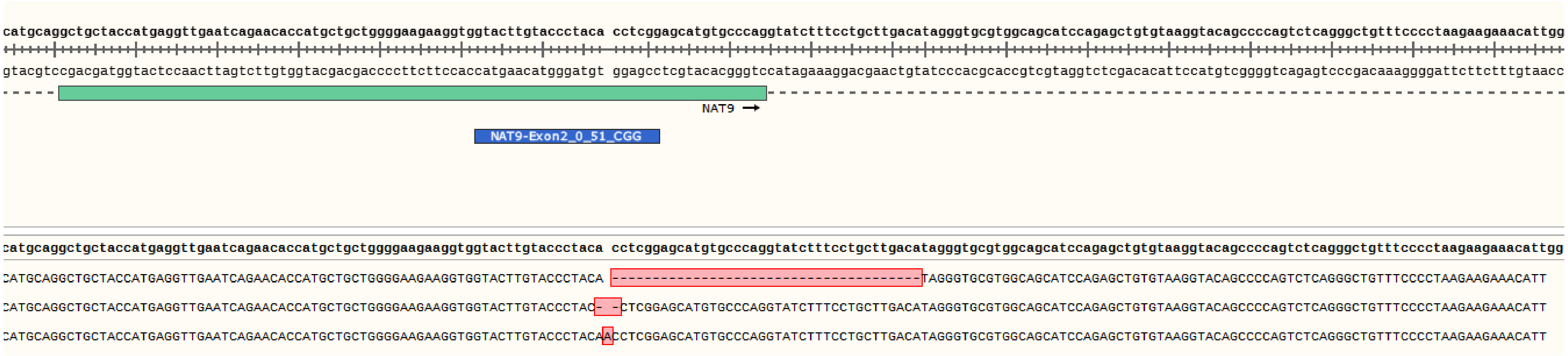

Name	Clone ID	Total # reads	# wt reads(%)	#1-Indel	#1-Reads(%)	#2-Indel	#2-Reads(%)	#3-Indel	#3-Reads(%)
EPNAT DKO data EP300 3C6		2143	0 (0.0%)	-16	1175 (54.8%)	13	951 (44.4%)	12	8 (0.4%)
EPNAT DKO data NAT9 3C6	079D	1777	0 (0.0%)	-38	707 (39.8%)	-2	541 (30.4%)	1	472 (26.6%)

Clone ID	Clone ID	Total # reads	# wt reads(%)	#1-Indel	#1-Reads(%)	#2-Indel	#2-Reads(%)	#3-Indel	#3-Reads(%)
EPNAT DKO data EP300 3C6		5434	33 (0.6%)	-16	3227 (59.4%)	13	2039 (37.5%)	-17	52 (1.0%)
EPNAT DKO data NAT9 3C6	079D	18479	1 (0.0%)	-38	7343 (39.7%)	-2	5076 (27.5%)	1	4896 (26.5%)

Clone 049D (-14/-2 EP300; -47/+1 NEU2)

EP300: NGS 1, NGS 2 (cell bank)

NEU2: NGS 1, NGS 2 (cell bank)

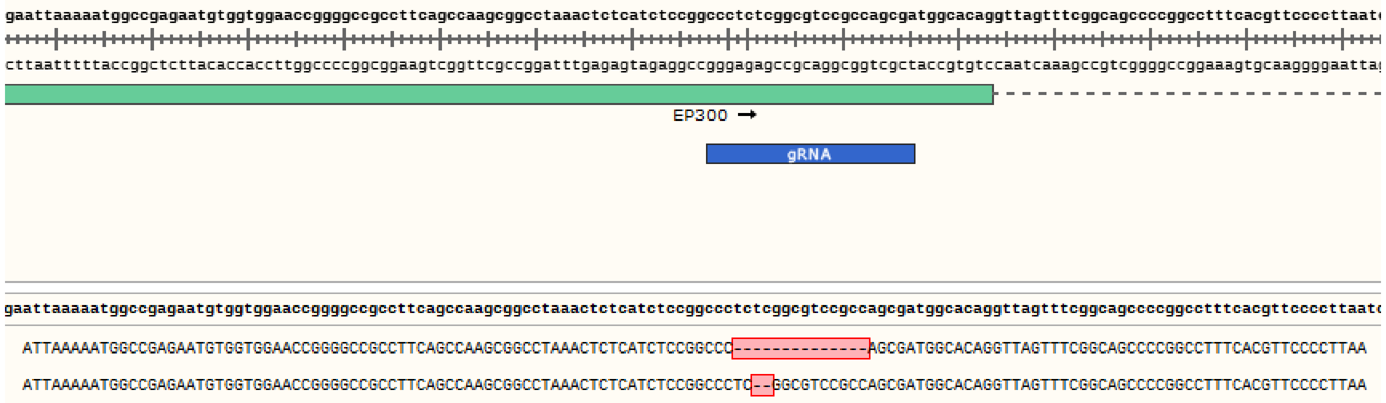

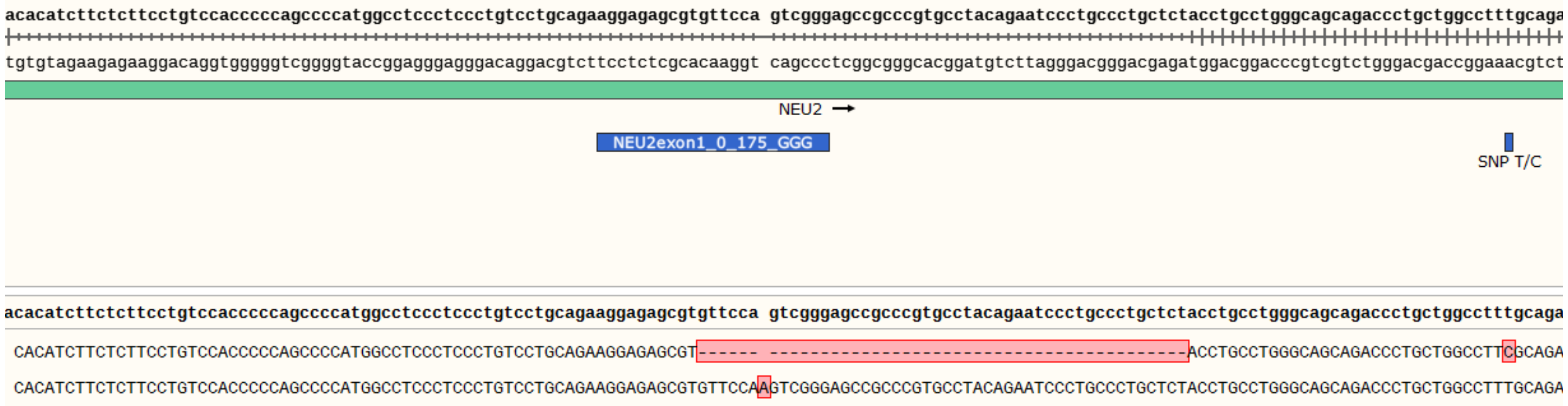

Name	Clone ID	Total # reads	# wt reads(%)	#1-Indel	#1-Reads(%)	#2-Indel	#2-Reads(%)
EPNEU DKO data EP300 1H11		2789	0 (0.0%)	-14	1413 (50.7%)	-2	1353 (48.5%)
EPNEU DKO data NEU2 1H11	049D	2069	0 (0.0%)	-47	1336 (64.6%)	1	692 (33.4%)

Clone ID	Clone ID	Total # reads	# wt reads(%)	#1-Indel	#1-Reads(%)	#2-Indel	#2-Reads(%)
EPNEU DKO data EP300 1H11		5707	9 (0.2%)	-2	3021 (52.9%)	-14	2587 (45.3%)
EPNEU DKO data NEU2 1H11	049D	6149	1 (0.0%)	-47	3751 (61.0%)	1	2257 (36.7%)

Clone 051D (-32/-10 EP300; -22/-19 NEU2)

EP300: NGS 1, NGS 2 (cell bank)

NEU2: NGS 1, NGS 2 (cell bank)

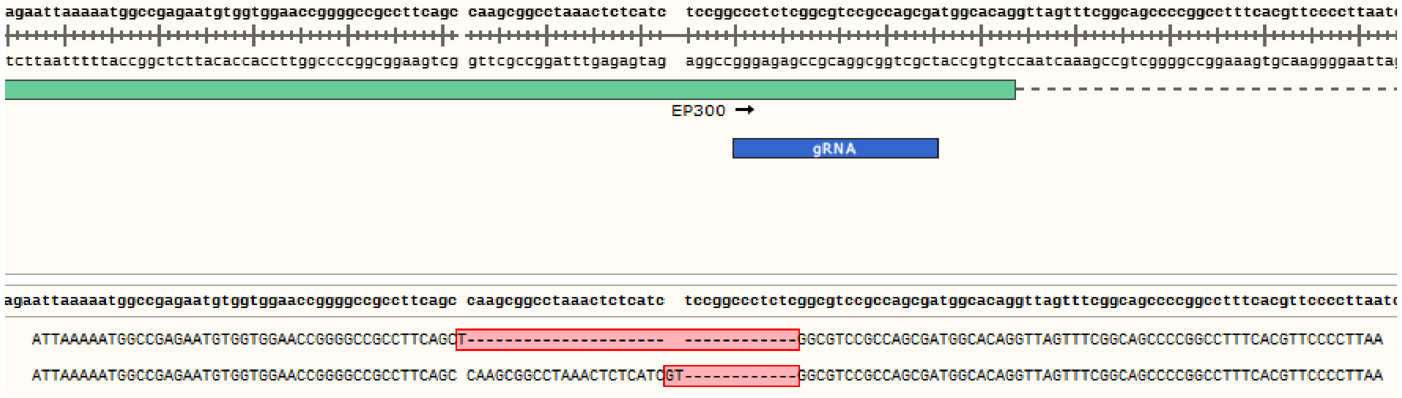

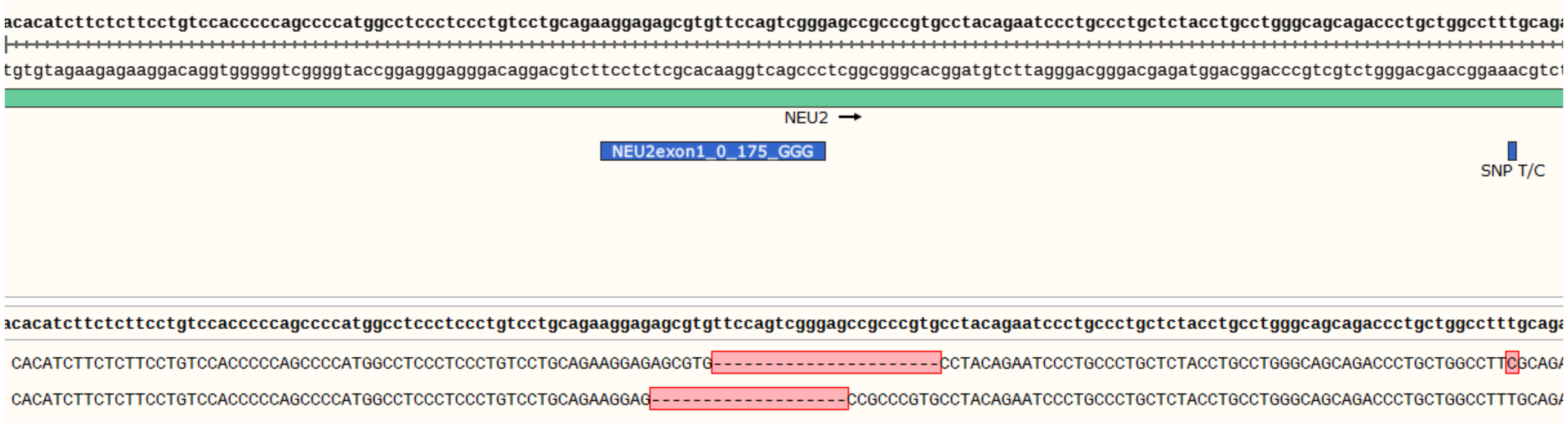

Name	Clone ID	Total # reads	# wt reads(%)	#1-Indel	#1-Reads(%)	#2-Indel	#2-Reads(%)
EPNEU DKO data EP300 3B3		2588	0 (0.0%)	-32	1328 (51.3%)	-10	1249 (48.3%)
EPNEU DKO data NEU2 3B3	051D	1657	1 (0.1%)	-22	843 (50.9%)	-19	787 (47.5%)

Clone ID	Clone ID	Total # reads	# wt reads(%)	#1-Indel	#1-Reads(%)	#2-Indel	#2-Reads(%)
EPNEU DKO data EP300 3B3		4357	0 (0.0%)	-32	2206 (50.6%)	-10	2098 (48.2%)
EPNEU DKO data NEU2 3B3	051D	5554	0 (0.0%)	-19	2613 (47.0%)	-22	2380 (42.9%)

Clone 052D (-2/-527 EP300; -17/+1 NEU2)

EP300: NGS 1, agarose gel PCR, sequencing, NGS 2 (cell bank)

NEU2: NGS 1, NGS 2 (cell bank)

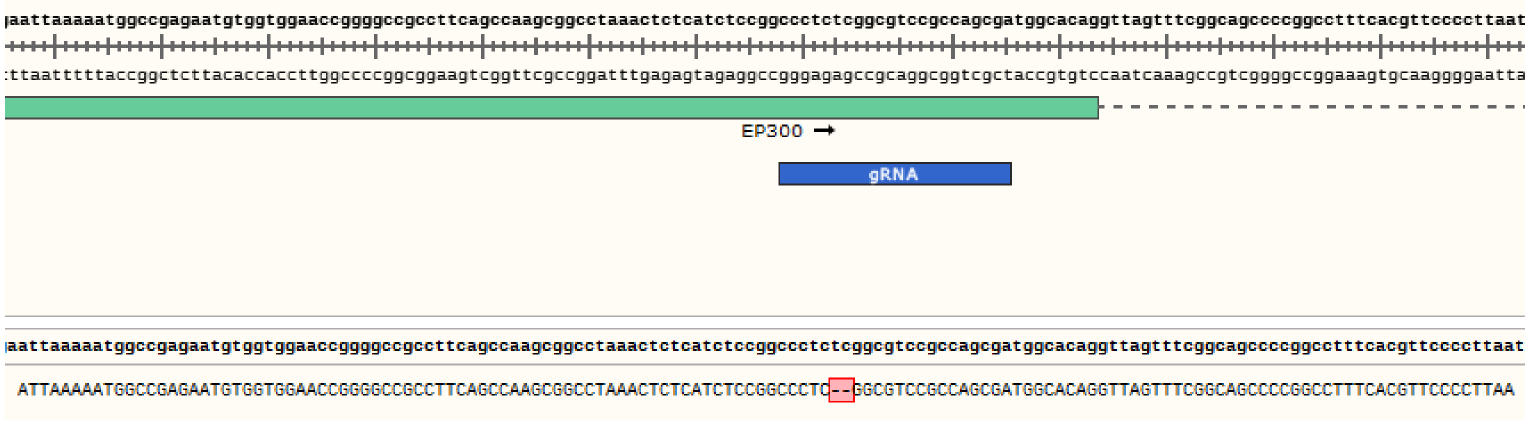

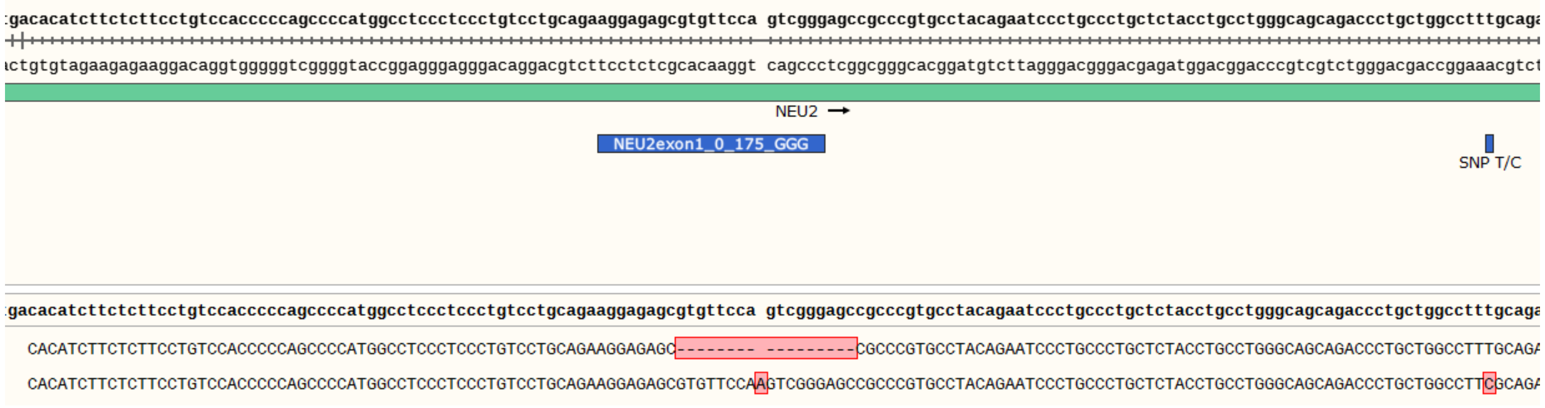

Name	Clone ID	Total # reads	# wt reads(%)	#1-Indel	#1-Reads(%)	#2-Indel	#2-Reads(%)
EPNEU DKO data EP300 4E8		2791	0 (0.0%)	-2	2772 (99.3%)	-3	18 (0.6%)
EPNEU DKO data NEU2 4E8	052D	1894	0 (0.0%)	-17	1098 (58.0%)	1	770 (40.7%)

Clone 052D (-2/-527 EP300; -17/+1 NEU2)

EP300: NGS 1, agarose gel PCR, sequencing, NGS 2 (cell bank)

NEU2: NGS 1, NGS 2 (cell bank)

EP300

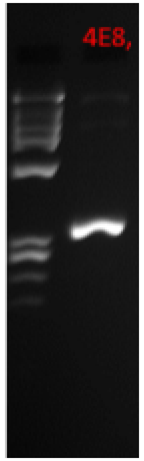

EP300 sequencing -527bp

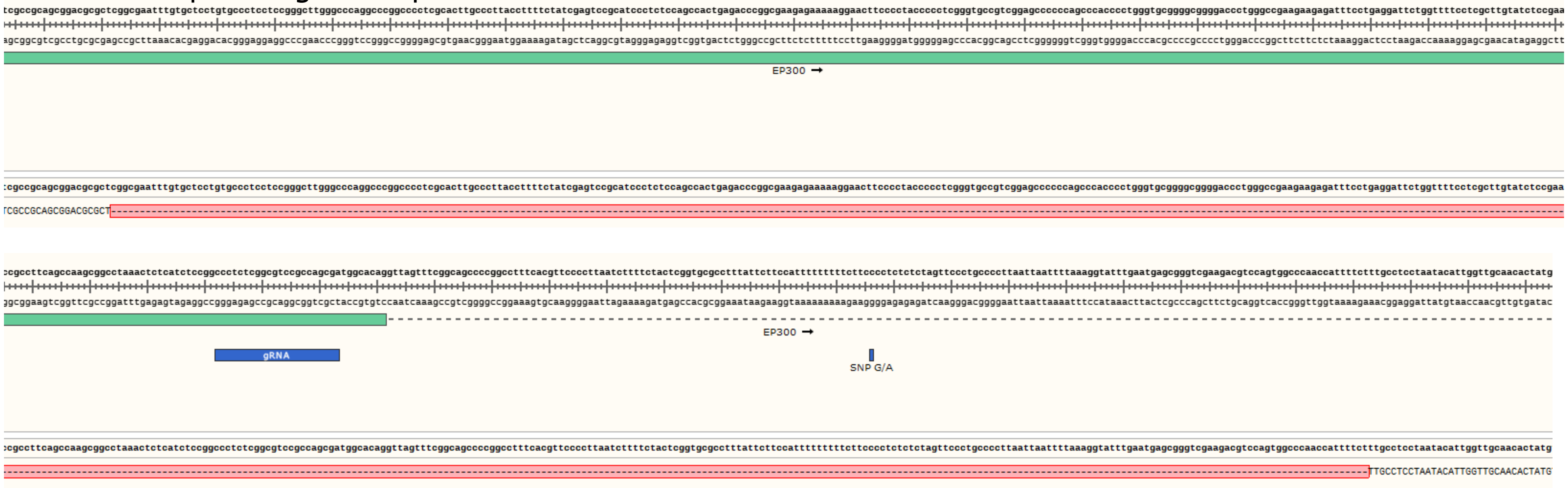

EP300 deleted sequence:

cggcgaatttgtgctcctgtgccctcctccgggcttgggcccaggcccggcccctcgcacttgcccttaccttttctatcgagtccgcatccctctccagccactgagacccggcgaag
agaaaaaggaacttcccctacccccctcgggtgccgtcggagcccccagcccaccccctgggtgcggggcggggaccctgggccgaagaagagatttcctgaggattctggttttc
ctcgcttgtatctccgaaagaattaaaaatggccgagaatgtggtggaaccggggccgccttcagccaagcggcctaaactctcatctccggccctctcggcgtccgccagcgatg
gcacaggttagtttcggcagccccggcctttcacgttccccttaatcttttctactcggtgcgcctttattcttccattttttttcttcccctctctctagttccctgcccttaattaattttaa
aggtatttgaatgagcgggtcgaagacgtccagtggcccaaccattttct

Clone ID	Clone ID	Total # reads	# wt reads(%)	#1-Indel	#1-Reads(%)	#2-Indel	#2-Reads(%)
EPNEU DKO data EP300 4E8		4098	0 (0.0%)	-2	4022 (98.1%)	-3	72 (1.8%)
EPNEU DKO data NEU2 4E8	052D	4815	1 (0.0%)	-17	2304 (47.9%)	1	2105 (43.7%)

Clone 053D (-11/+1 EP300; -20/-263 NEU2)

EP300: NGS 1, NGS 2 (cell bank)

NEU2: NGS 1, fragment analysis, topo sequencing, NGS 2 (cell bank)

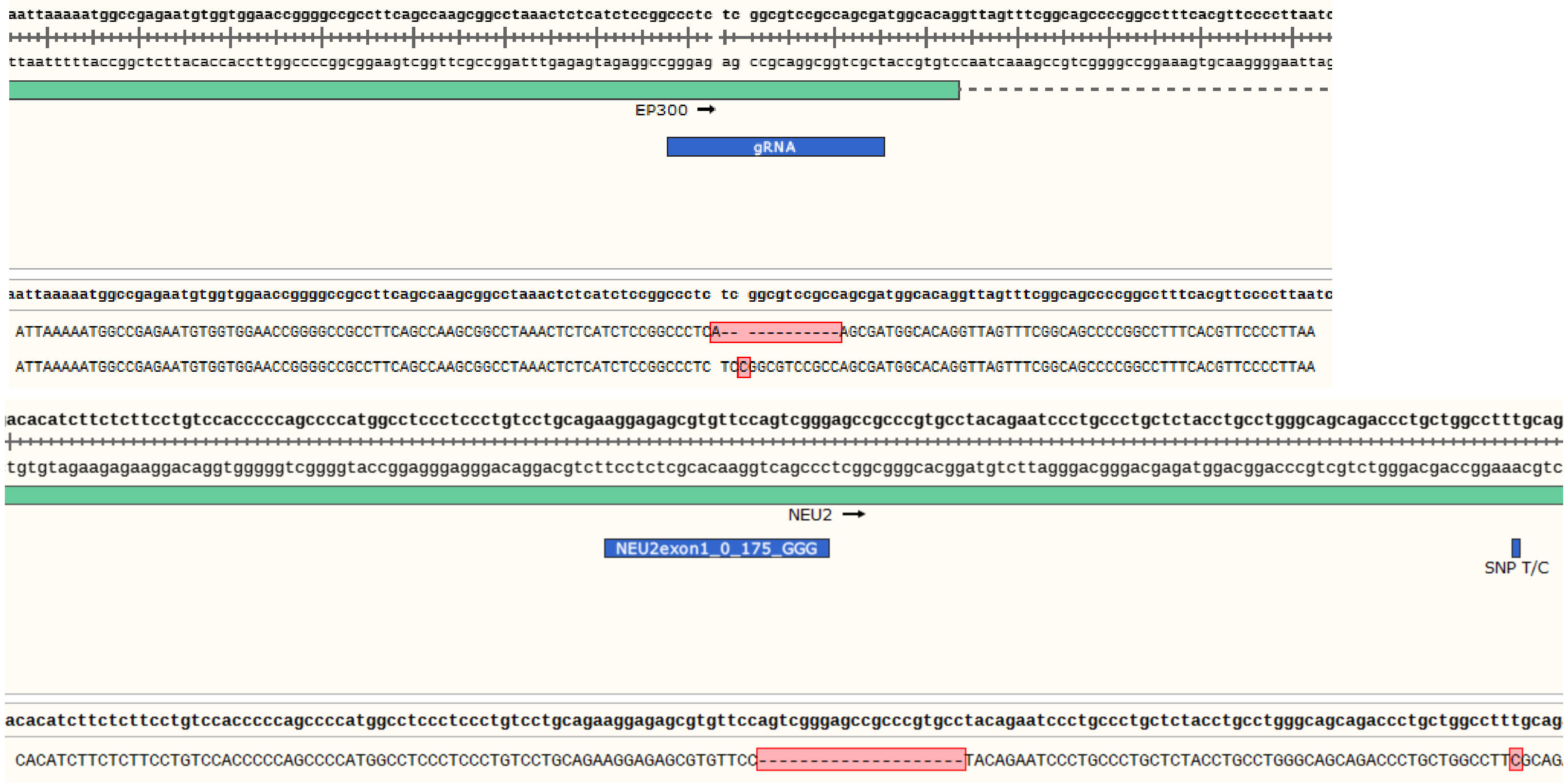

Name	Clone ID	Total # reads	# wt reads(%)	#1-Indel	#1-Reads(%)	#2-Indel	#2-Reads(%)
EPNEU DKO data EP300 4G7		2631	0 (0.0%)	-11	1400 (53.2%)	1	1206 (45.8%)
EPNEU DKO data NEU2 4G7	053D	1599	0 (0.0%)	-20	1569 (98.1%)	-21	28 (1.8%)

Clone 053D (-11/+1 EP300; -20/-263 NEU2)

EP300: NGS 1, NGS 2 (cell bank)

NEU2: NGS 1, fragment analysis, topo sequencing, NGS 2 (cell bank)

NEU2 fragment analysis

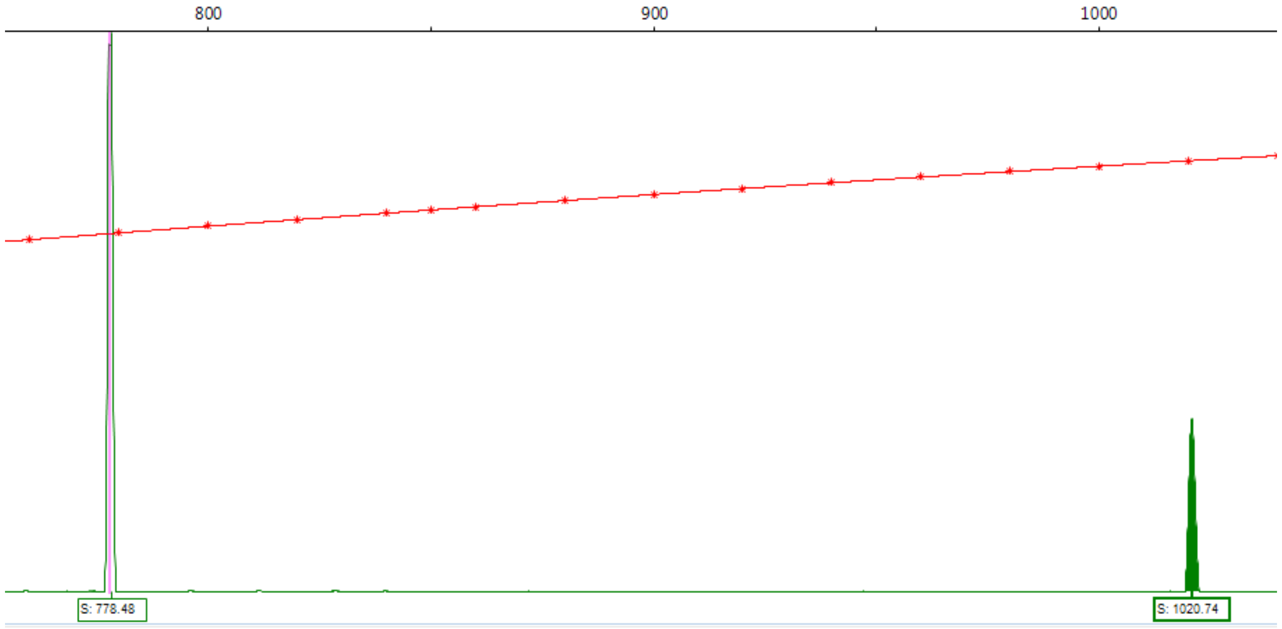

Clone 053D (-11/+1 EP300; -20/-263 NEU2)

EP300: NGS 1, NGS 2 (cell bank)

NEU2: NGS 1, fragment analysis, topo sequencing, NGS 2 (cell bank)

NEU2 sequencing -263bp

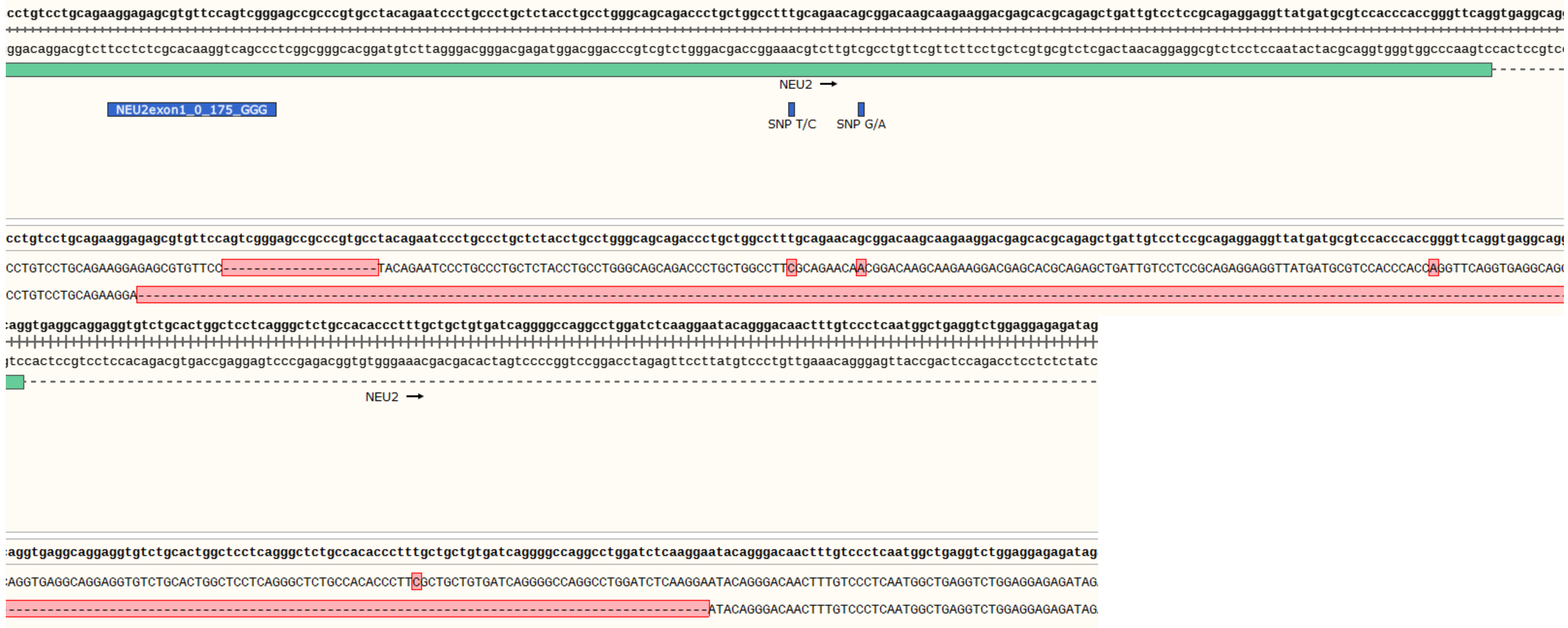

NEU2 deleted sequence:

gagcgtgttccagtcgggagccgcccgtgcctacagaatccctgccctgctctacctgcctgggcagcagaccctgctggcctttgcagaacagcggacaagcaagaaggacgagcacgc
agagctgattgtcctccgcagaggaggttatgatgcgtccacccaccgggttcaggtgaggcaggaggtgtctgcactggctcctcagggctctgccacaccctttgctgctgtgatcagggg
ccaggcctggatctcaagga

Clone ID	Clone ID	Total # reads	# wt reads(%)	#1-Indel	#1-Reads(%)	#2-Indel	#2-Reads(%)
EPNEU DKO data EP300 4G7		5382	9 (0.2%)	-11	2733 (50.8%)	1	2585 (48.0%)
EPNEU DKO data NEU2 4G7	053D	7130	0 (0.0%)	-20	6200 (87.0%)	-100	553 (7.8%)

Clone 055D (-203/-416 EP300; -31/-8 NEU2)

EP300: NGS 1, fragment analysis, topo sequencing, NGS 2 (cell bank)

NEU2: NGS 1, NGS 2 (cell bank)

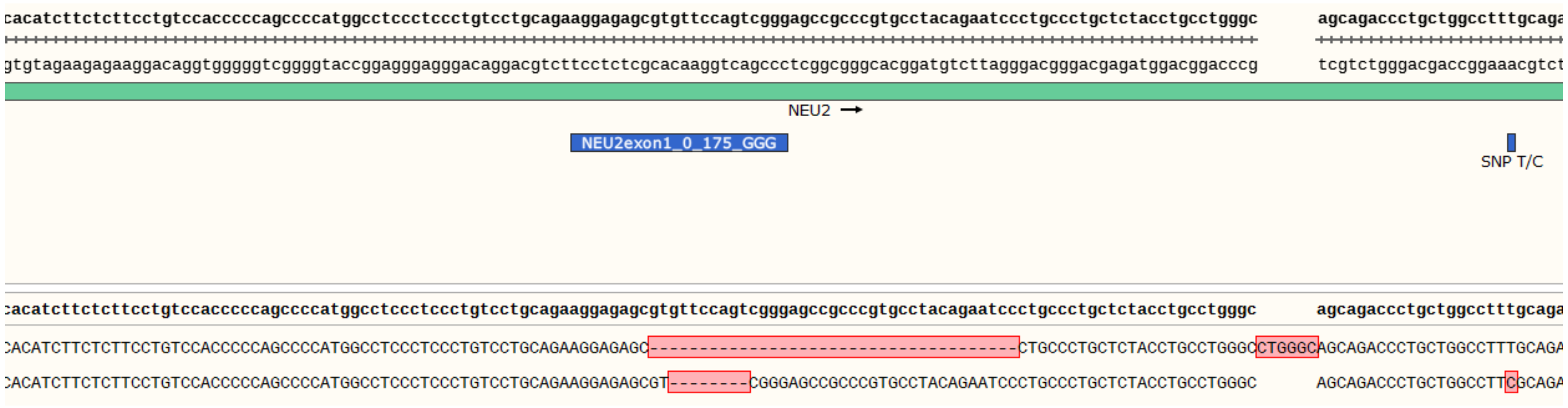

Name	Clone ID	Total # reads	# wt reads(%)	#1-Indel	#1-Reads(%)	#2-Indel	#2-Reads(%)
EPNEU DKO data EP300 2F6		no data					
EPNEU DKO data NEU2 2F6	055D	2330	0 (0.0%)	-31	1360 (58.4%)	-8	907 (38.9%)

Clone 055D (-203/-416 EP300; -31/-8 NEU2)

EP300: NGS 1, fragment analysis, topo sequencing, NGS 2 (cell bank)

NEU2: NGS 1, NGS 2 (cell bank)

EP300 fragment analysis

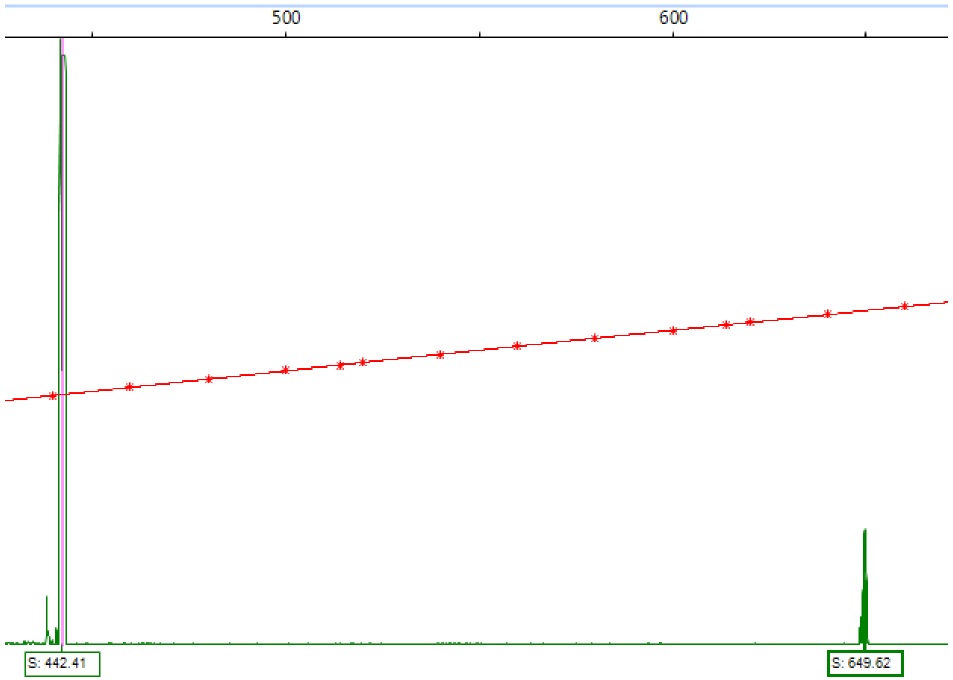

Clone 055D (-203/-416 EP300; -31/-8 NEU2)

EP300: NGS 1, fragment analysis, topo sequencing, NGS 2 (cell bank)

NEU2: NGS 1, NGS 2 (cell bank)

EP300 sequencing -203/-416

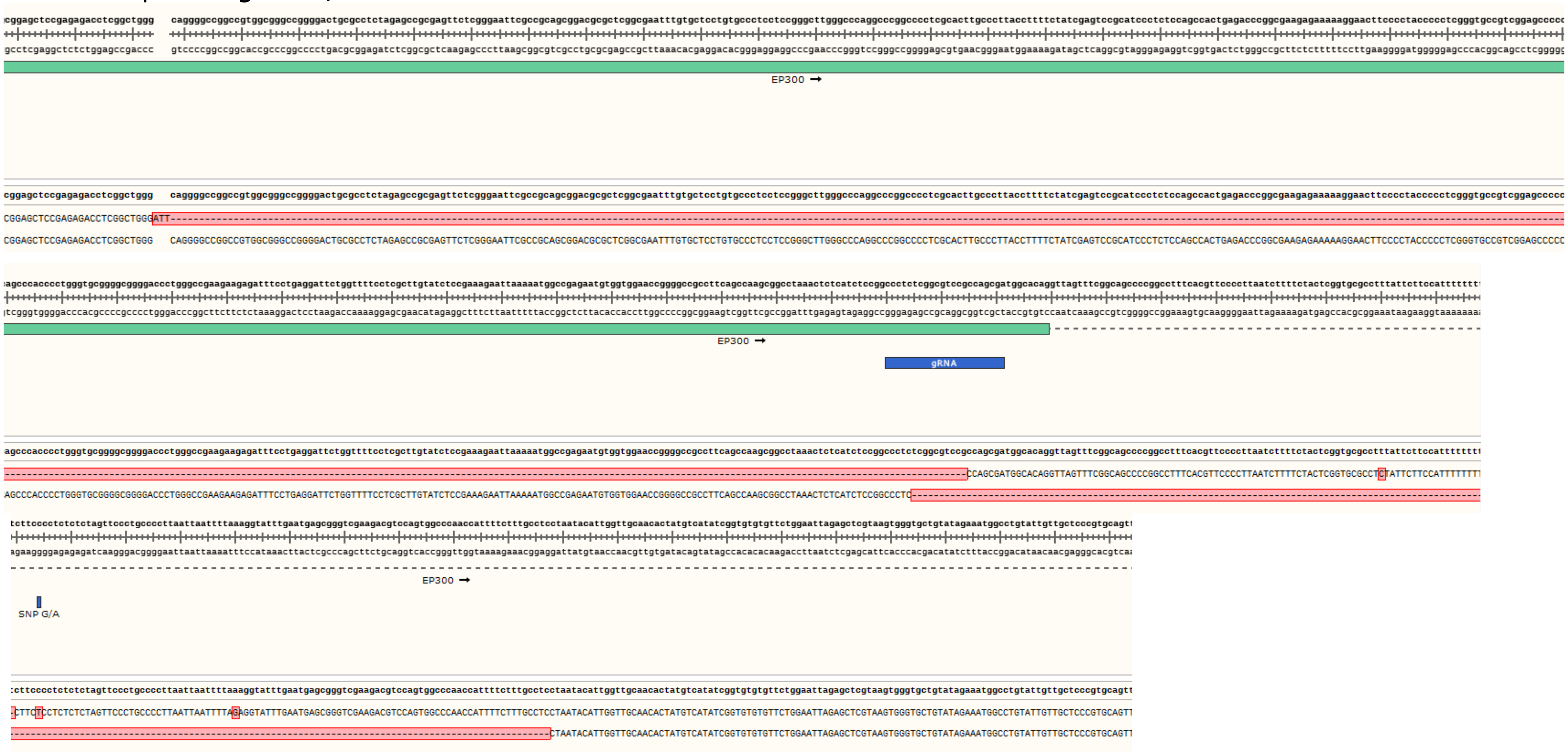

Clone 055D (-203/-416 EP300; -31/-8 NEU2)

EP300: NGS 1, fragment analysis, topo sequencing, NGS 2 (cell bank)

NEU2: NGS 1, NGS 2 (cell bank)

EP300 deleted sequences:

taggggccggccgtggcgggccggggactgcgcctctagagccgcgagttctcgggaattcgccgcagcggacgcgctcggcg
aatttgtgctcctgtgccctcctccgggcttgggcccaggcccggcccctcgcacttgcccttaccttttctatcgagtccgcatccctct
ccagccactgagacccggcgaagagaaaaggaacttcccctacccctcgggtgccgtcggagcccccagcccacccctgggt
gcggggcggggaccctgggccgaagaagagatttcctgaggattctggttttcctcgcttgtatctccgaaagaattaaaaatggc
cgagaatgtggtggaaccggggccgccttcagccaagcggcctaaactctcatctccggccctctcggcgt

tcggcgtccgccagcgatggcacaggttagtttcggcagccccggcctttcacgttccccttaatcttttctactcggtgcgcctttatt
cttccattttttttttcttcccctctctctagttccctgccccttaattaattttaaaggtatttgaatgagcgggtcgaagacgtccagtgg
cccaaccattttctttgcctc

Clone ID	Clone ID	Total # reads	# wt reads(%)	#1-Indel	#1-Reads(%)	#2-Indel	#2-Reads(%)
EPNEU DKO data EP300 2F6		not possible, large deletions					
EPNEU DKO data NEU2 2F6	055D	1121	0 (0.0%)	-31	629 (56.1%)	-8	374 (33.4%)

Clone 058D (-50/-86 EP300; -22/-19 NEU2)

EP300: NGS 1, fragment analysis, topo sequencing NGS 2 (cell bank)

NEU2: NGS 1, NGS 2 (cell bank)

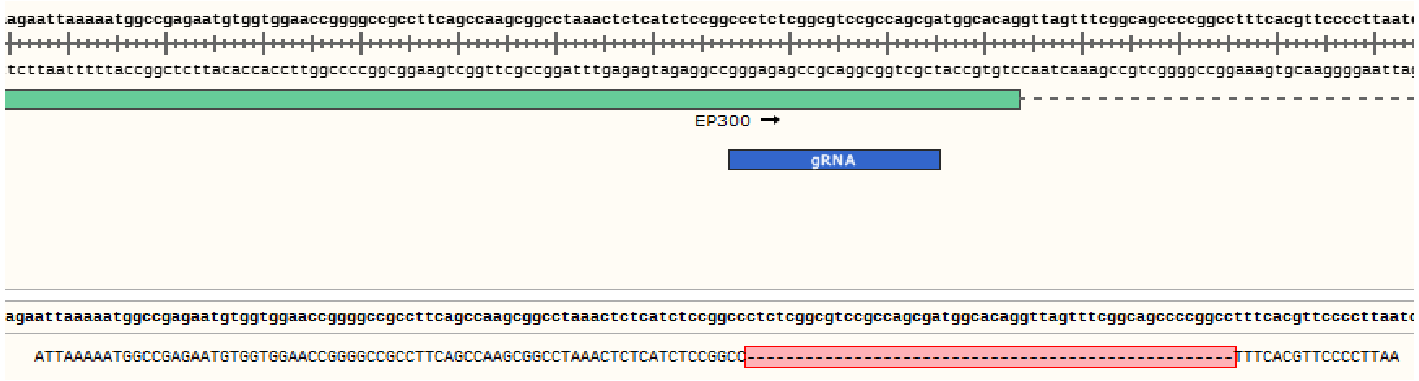

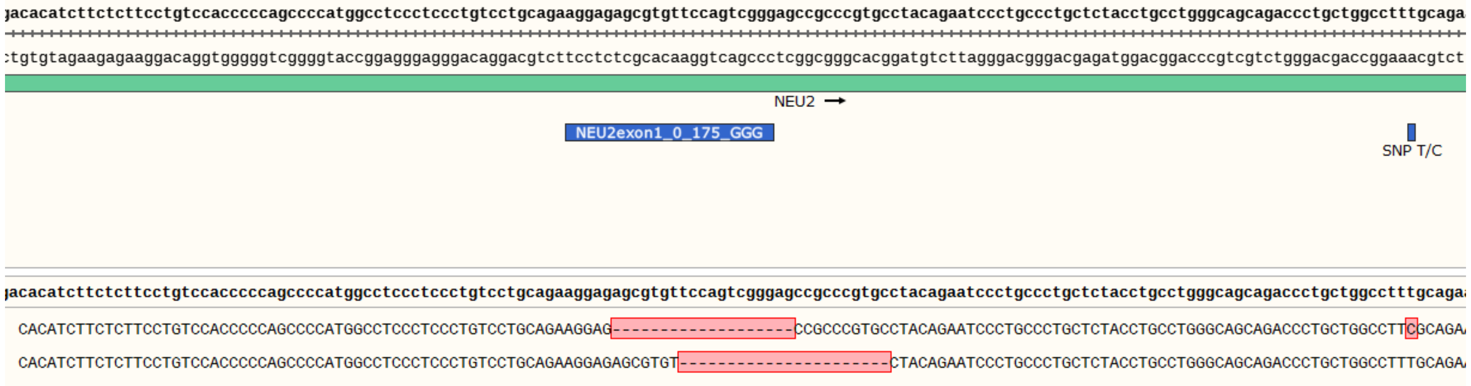

Name	Clone ID	Total # reads	# wt reads(%)	#1-Indel	#1-Reads(%)	#2-Indel	#2-Reads(%)
EPNEU DKO data EP300 1G5		1970	0 (0.0%)	-50	1959 (99.4%)	-51	10 (0.5%)
EPNEU DKO data NEU2 1G5	058D	1911	0 (0.0%)	-22	957 (50.1%)	-19	922 (48.2%)

Clone 058D (-50/-86 EP300; -22/-19 NEU2)

EP300: NGS 1, fragment analysis, topo sequencing NGS 2 (cell bank)

NEU2: NGS 1, NGS 2 (cell bank)

EP300 fragment analysis

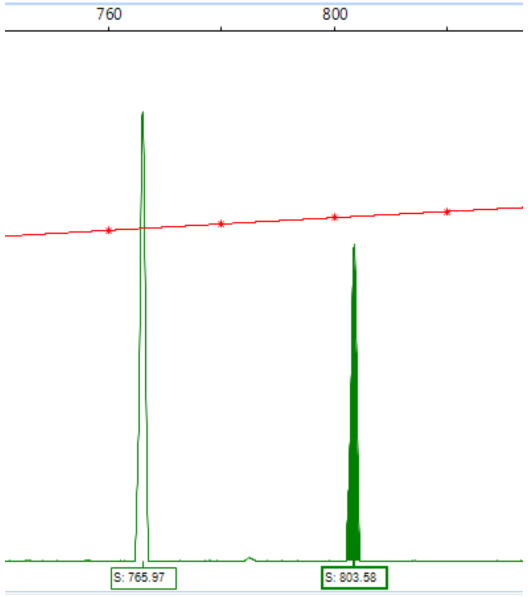

EP300 sequencing

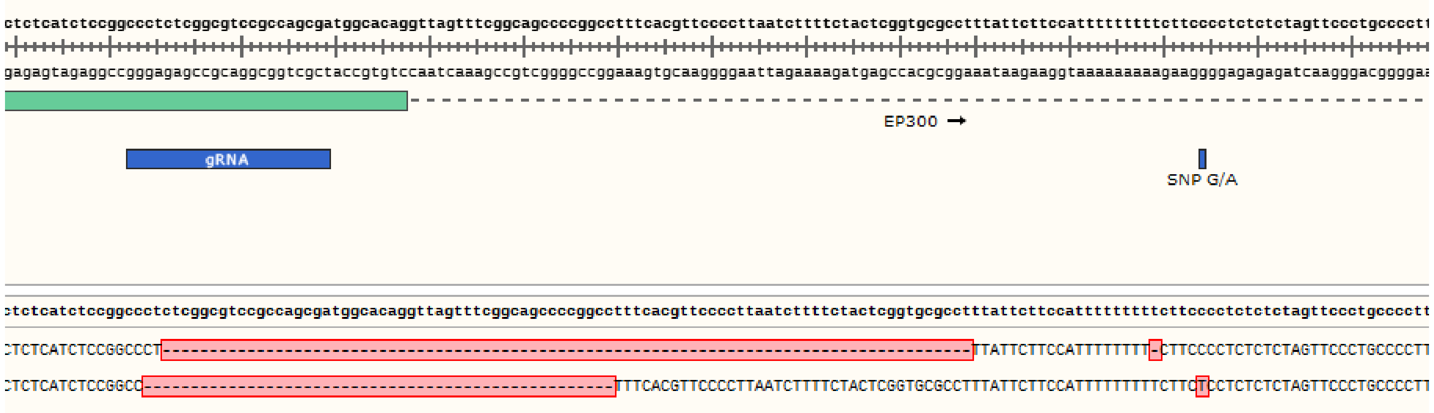

Clone ID	Clone ID	Total # reads	# wt reads(%)	#1-Indel	#1-Reads(%)	#2-Indel	#2-Reads(%)
EPNEU DKO data EP300 1G5		3585	1 (0.0%)	-50	3546 (98.9%)	-51	38 (1.1%)
EPNEU DKO data NEU2 1G5	058D	6427	0 (0.0%)	-19	3183 (49.5%)	-22	2530 (39.4%)

Clone 029D (-58/-20 CNTD2; -2/+1 COQ9)

CNTD2: NGS 1, NGS 2 (cell bank)

COQ9: NGS 1, NGS 2 (cell bank)

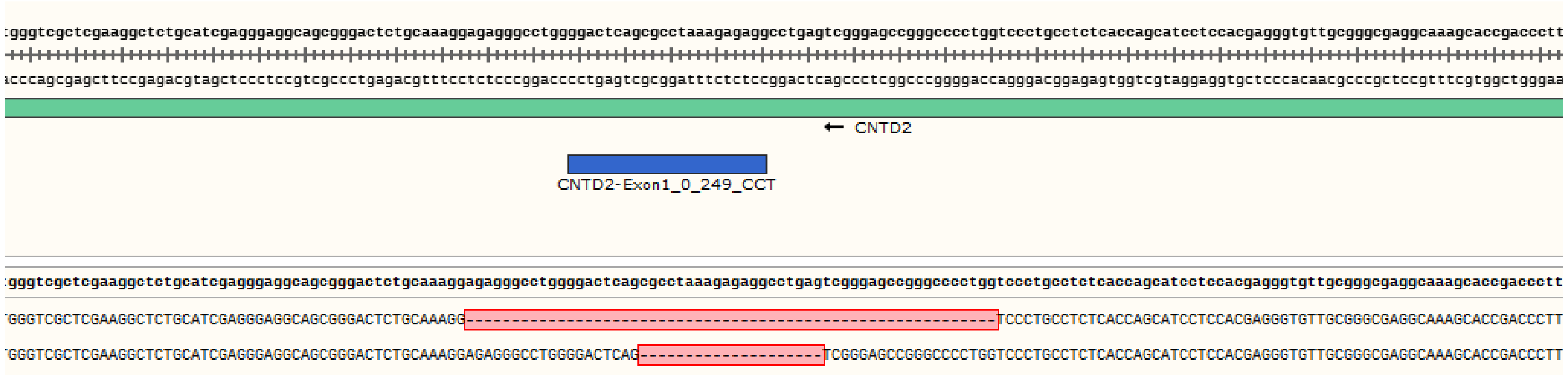

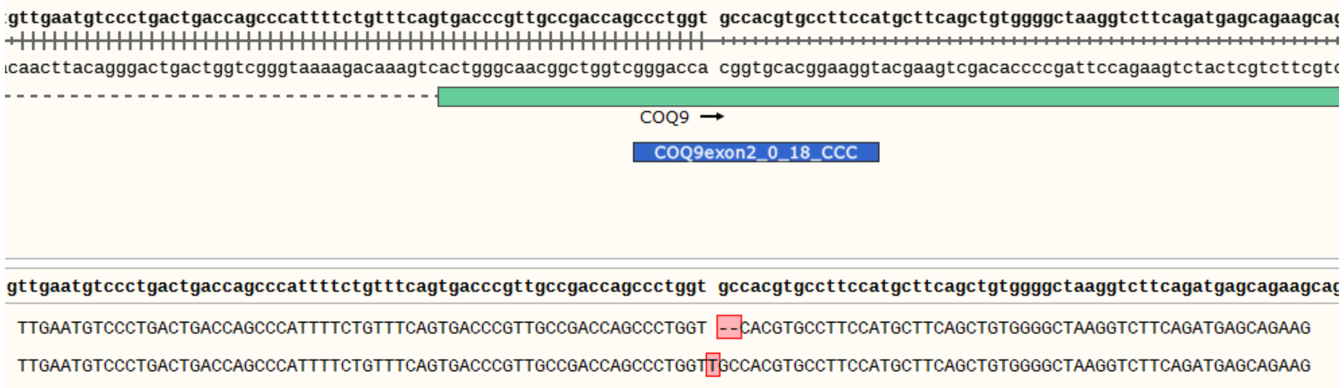

Name	Clone ID	Total # reads	# wt reads(%)	#1-Indel	#1-Reads(%)	#2-Indel	#2-Reads(%)
CNTCOQ DKO data CNTD2 2F12		1820	2 (0.1%)	-58	1028 (56.5%)	-20	756 (41.5%)
CNTCOQ DKO data COQ9 2F12	029D	1979	0 (0.0%)	-2	1013 (51.2%)	1	937 (47.3%)

Clone ID	Clone ID	Total # reads	# wt reads(%)	#1-Indel	#1-Reads(%)	#2-Indel	#2-Reads(%)
CNTCOQ DKO data CNTD2 2F12		1538	0 (0.0%)	-20	893 (58.1%)	-58	617 (40.1%)
CNTCOQ DKO data COQ9 2F12	029D	5310	1 (0.0%)	-2	2694 (50.7%)	1	2446 (46.1%)

Clone 030D (-8/+1 CNTD2; -16/-23 COQ9)

CNTD2: NGS 1, NGS 2 (cell bank)

COQ9: NGS 1, NGS 2 (cell bank)

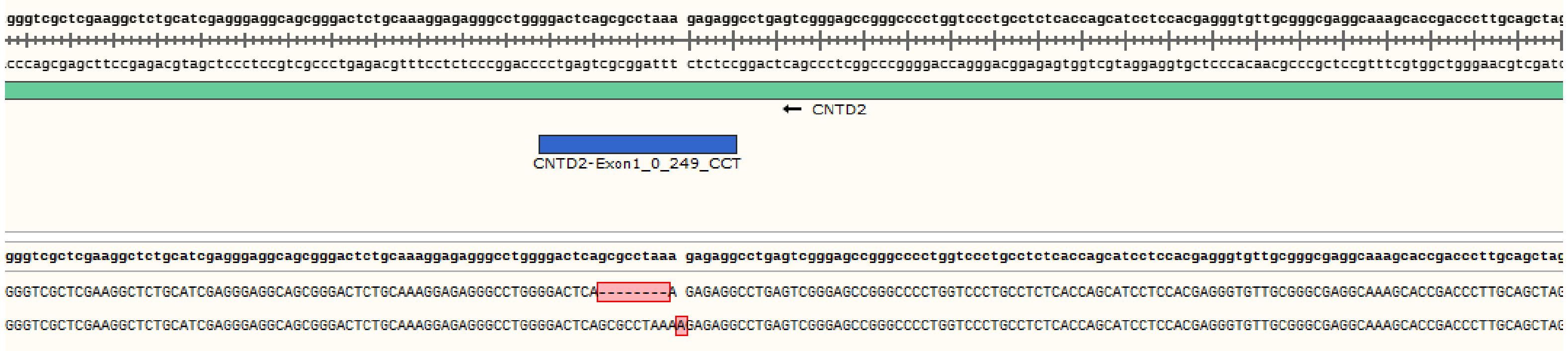

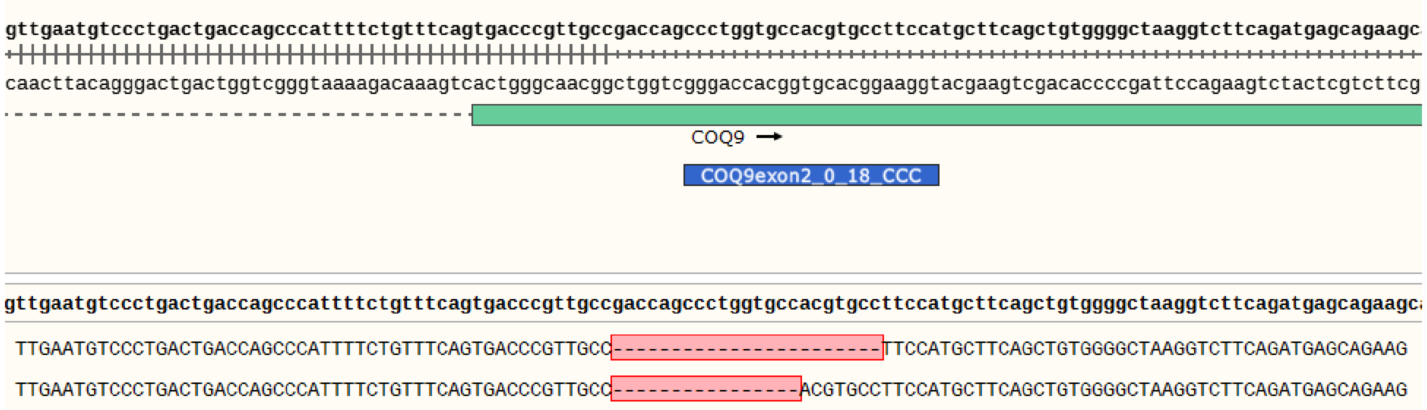

Name	Clone ID	Total # reads	# wt reads(%)	#1-Indel	#1-Reads(%)	#2-Indel	#2-Reads(%)
CNTCOQ DKO data CNTD2 2H5		1327	3 (0.2%)	-8	688 (51.8%)	1	604 (45.5%)
CNTCOQ DKO data COQ9 2H5	030D	1849	2 (0.1%)	-16	924 (50.0%)	-23	891 (48.2%)

Clone ID	Clone ID	Total # reads	# wt reads(%)	#1-Indel	#1-Reads(%)	#2-Indel	#2-Reads(%)
CNTCOQ DKO data CNTD2 2H5		1347	1 (0.1%)	-8	720 (53.5%)	1	604 (44.8%)
CNTCOQ DKO data COQ9 2H5	030D	5768	0 (0.0%)	-23	2861 (49.6%)	-16	2741 (47.5%)

Clone 031D (+1/-13 CNTD2; -23/+1 COQ9)

CNTD2: NGS 1, NGS 2 (cell bank)

COQ9: NGS 1, NGS 2 (cell bank)

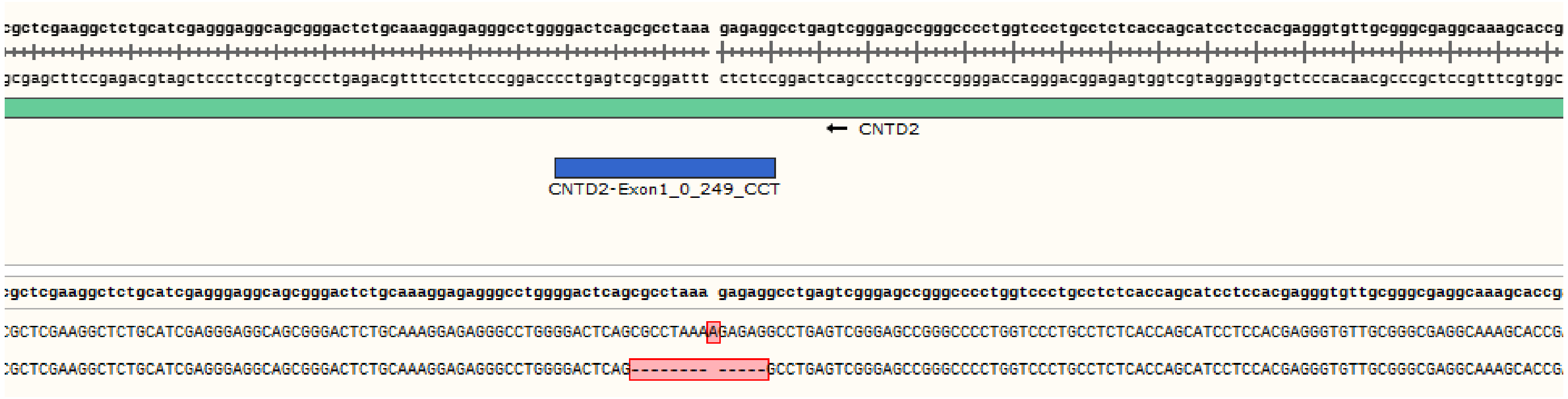

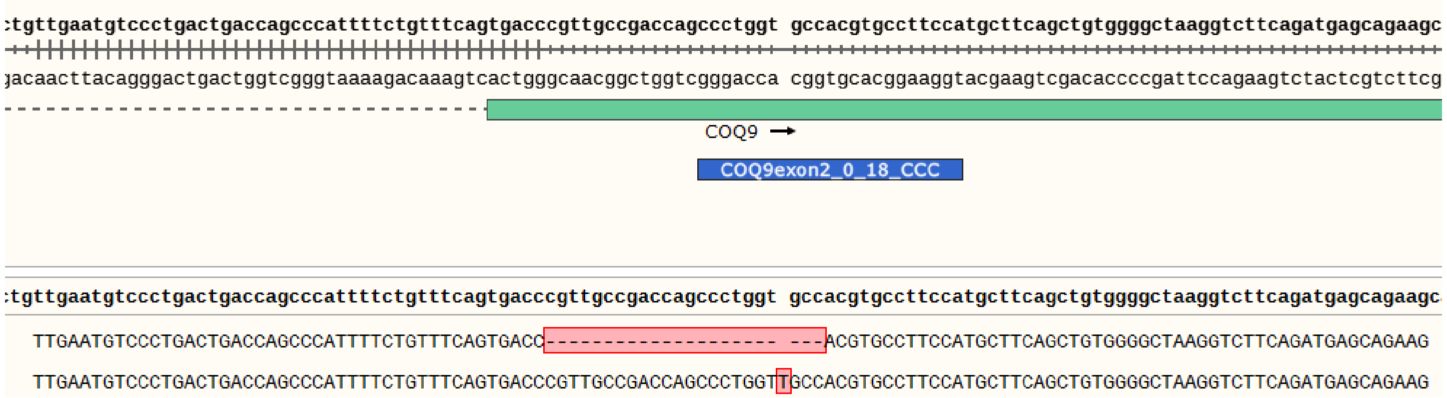

Name	Clone ID	Total # reads	# wt reads(%)	#1-Indel	#1-Reads(%)	#2-Indel	#2-Reads(%)
CNTCOQ DKO data CNTD2 3C12		109	0 (0.0%)	1	70 (64.2%)	-13	37 (33.9%)
CNTCOQ DKO data COQ9 3C12	031D	701	1 (0.1%)	1	361 (51.5%)	-23	335 (47.8%)

Clone ID	Clone ID	Total # reads	# wt reads(%)	#1-Indel	#1-Reads(%)	#2-Indel	#2-Reads(%)
CNTCOQ DKO data CNTD2 3C12		1571	2 (0.1%)	1	837 (53.3%)	-13	709 (45.1%)
CNTCOQ DKO data COQ9 3C12	031D	4551	0 (0.0%)	-23	2490 (54.7%)	1	1940 (42.6%)

Clone 036D (-1/-13 CNTD2; +1/+40 COQ9)

CNTD2: NGS 1, NGS 2 (cell bank)

COQ9: NGS 1, NGS 2 (cell bank)

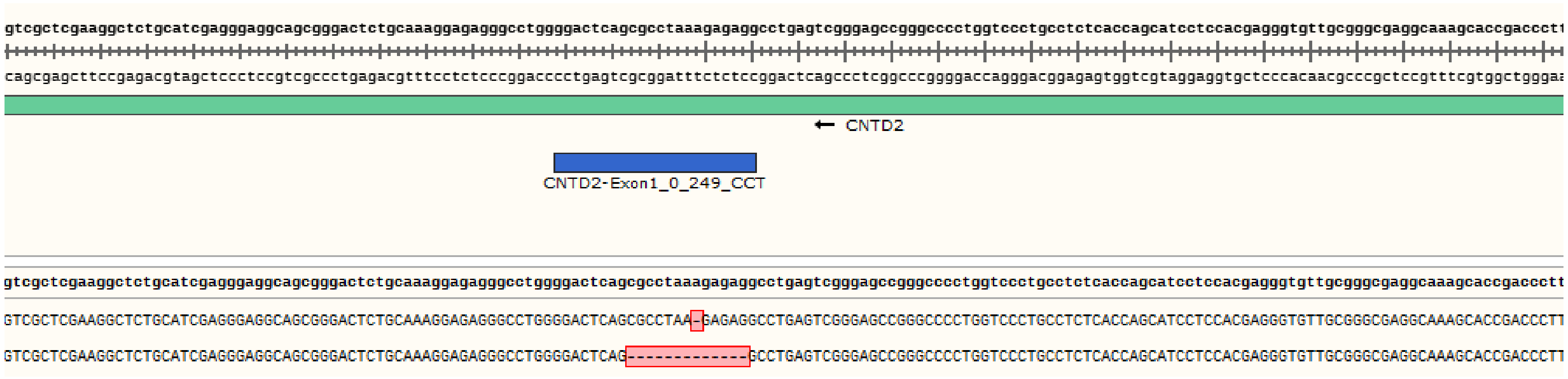

COQ9 +1

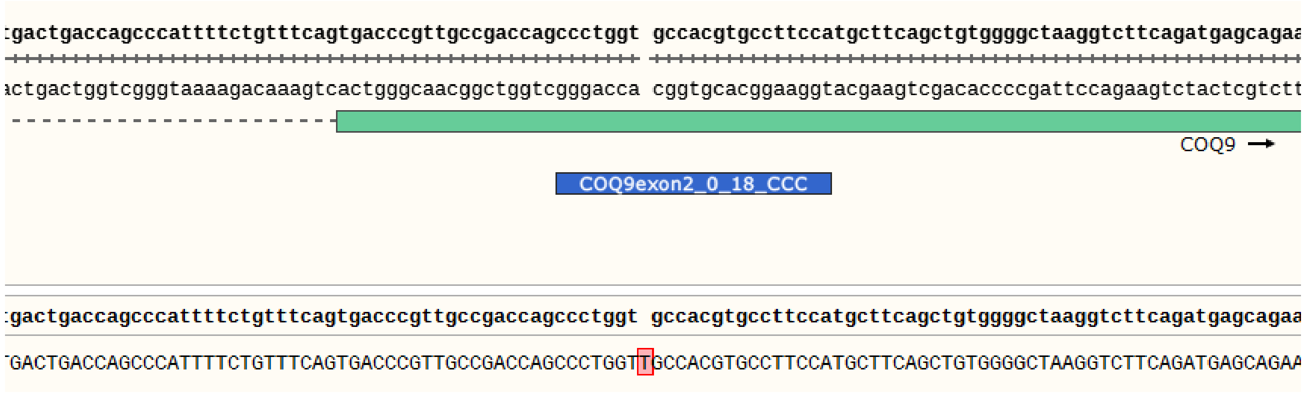

Clone 036D (-1/-13 CNTD2; +1/+40 COQ9)

CNTD2: NGS 1, NGS 2 (cell bank)

COQ9: NGS 1, NGS 2 (cell bank)

COQ9 +40 (illustrated in two lines due to alignment software inability to recognize and align the repetitive insertion)

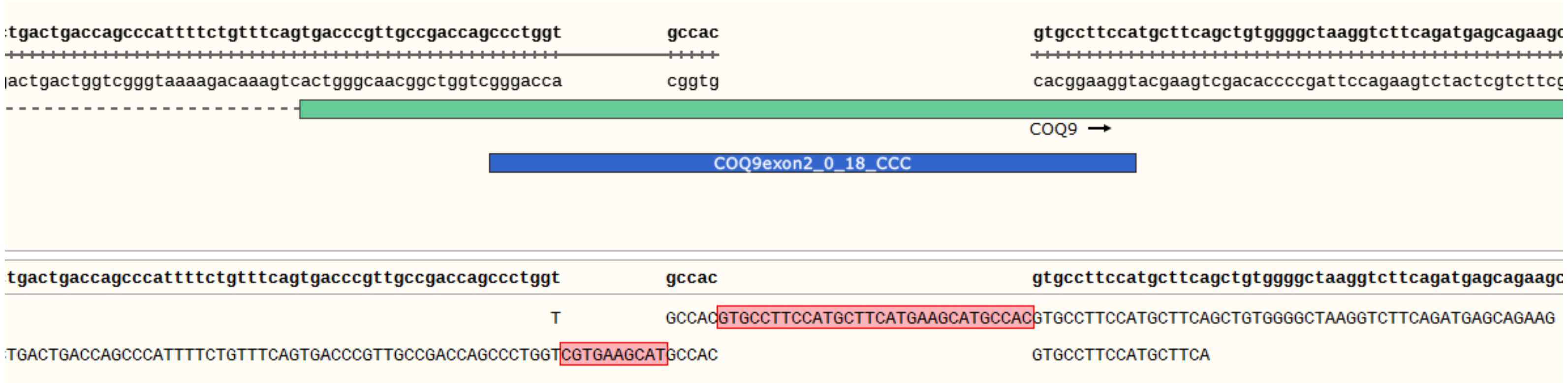

Name	Clone ID	Total # reads	# wt reads(%)	#1-Indel	#1-Reads(%)	#2-Indel	#2-Reads(%)
CNTCOQ DKO data CNTD2 1G4		975	1 (0.1%)	-1	466 (47.8%)	-13	464 (47.6%)
CNTCOQ DKO data COQ9 1G4	036D	686	0 (0.0%)	1	366 (53.4%)	40	310 (45.2%)

Clone ID	Clone ID	Total # reads	# wt reads(%)	#1-Indel	#1-Reads(%)	#2-Indel	#2-Reads(%)
CNTCOQ DKO data CNTD2 1G4		1307	0 (0.0%)	-1	651 (49.8%)	-13	632 (48.4%)
CNTCOQ DKO data COQ9 1G4	036D	3761	0 (0.0%)	1	1937 (51.5%)	40	1659 (44.1%)

Clone 037D (-13/+25 CNTD2; +1/+1 COQ9)

CNTD2: NGS 1, NGS 2 (cell bank)

COQ9: NGS 1, direct sequencing, NGS 2 (cell bank)

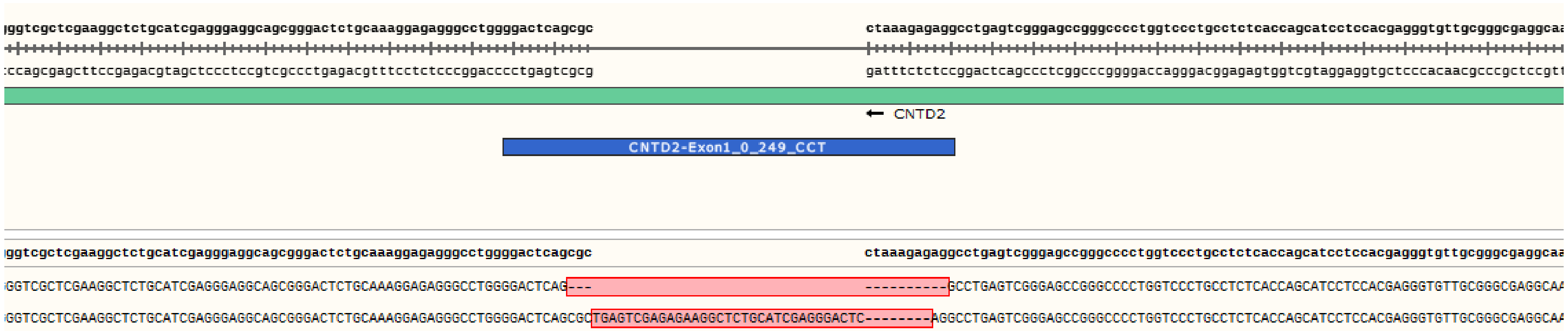

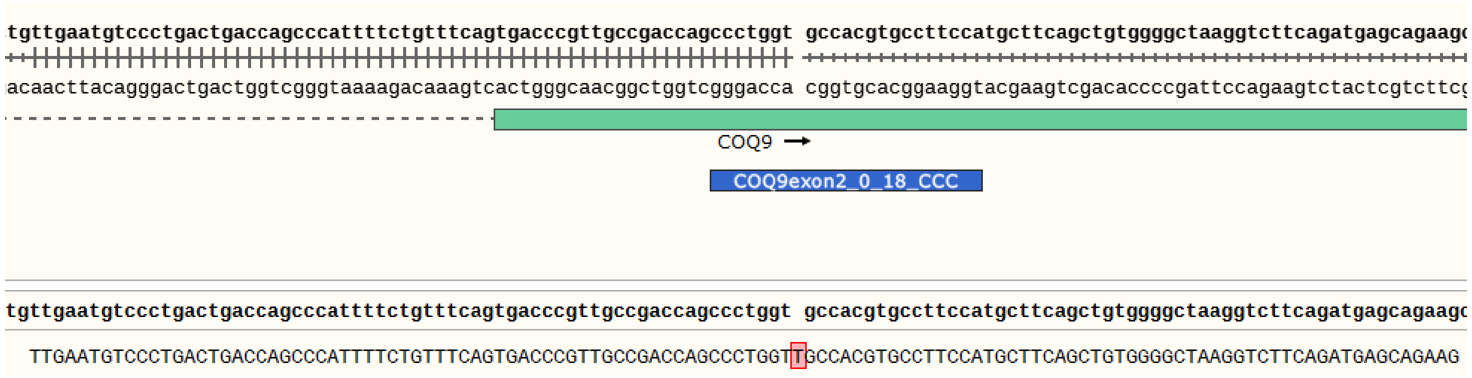

Name	Clone ID	Total # reads	# wt reads(%)	#1-Indel	#1-Reads(%)	#2-Indel	#2-Reads(%)
CNTCOQ DKO data CNTD2 2B10		1371	0 (0.0%)	-13	720 (52.5%)	25	620 (45.2%)
CNTCOQ DKO data COQ9 2B10	037D	1606	0 (0.0%)	1	1586 (98.8%)	0	18 (1.1%)

Clone 037D (-13/+25 CNTD2; +1/+1 COQ9)

CNTD2: NGS 1, NGS 2 (cell bank)

COQ9: NGS 1, direct sequencing, NGS 2 (cell bank)

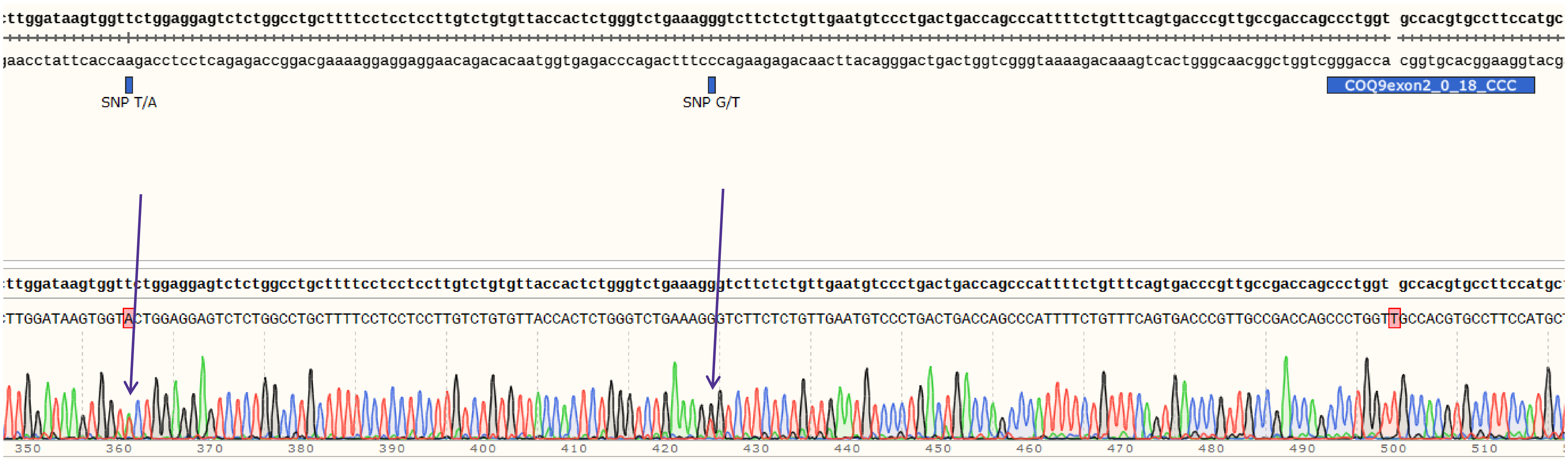

Clone ID	Clone ID	Total # reads	# wt reads(%)	#1-Indel	#1-Reads(%)	#2-Indel	#2-Reads(%)
CNTCOQ DKO data CNTD2 2B10		1495	0 (0.0%)	-13	785 (52.5%)	25	674 (45.1%)
CNTCOQ DKO data COQ9 2B10	037D	4076	0 (0.0%)	1	3917 (96.1%)	0	125 (3.1%)

Clone 038D (-13/-38 CNTD2; +1/+1 COQ9)

CNTD2: NGS 1, NGS 2 (cell bank)

COQ9: NGS 1, direct sequencing, NGS 2 (cell bank)

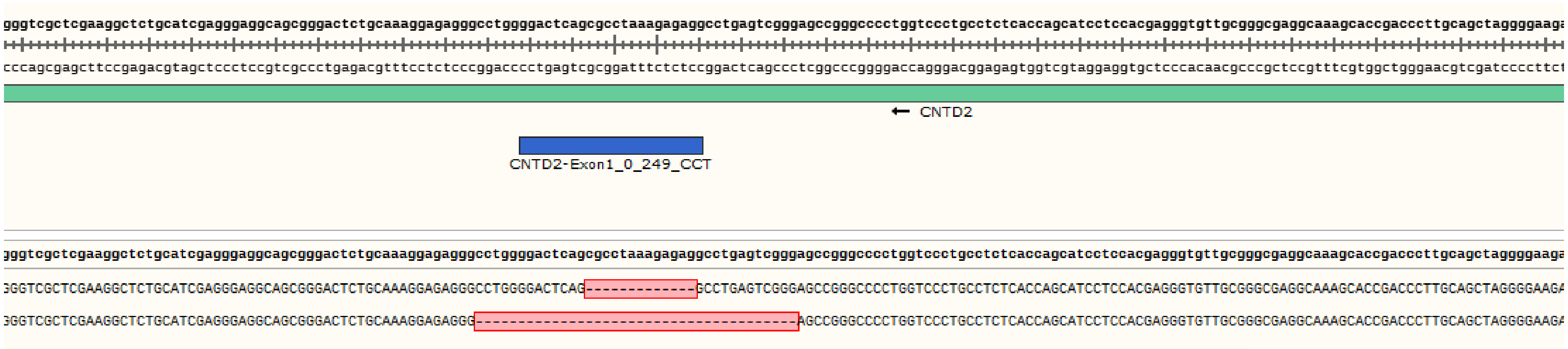

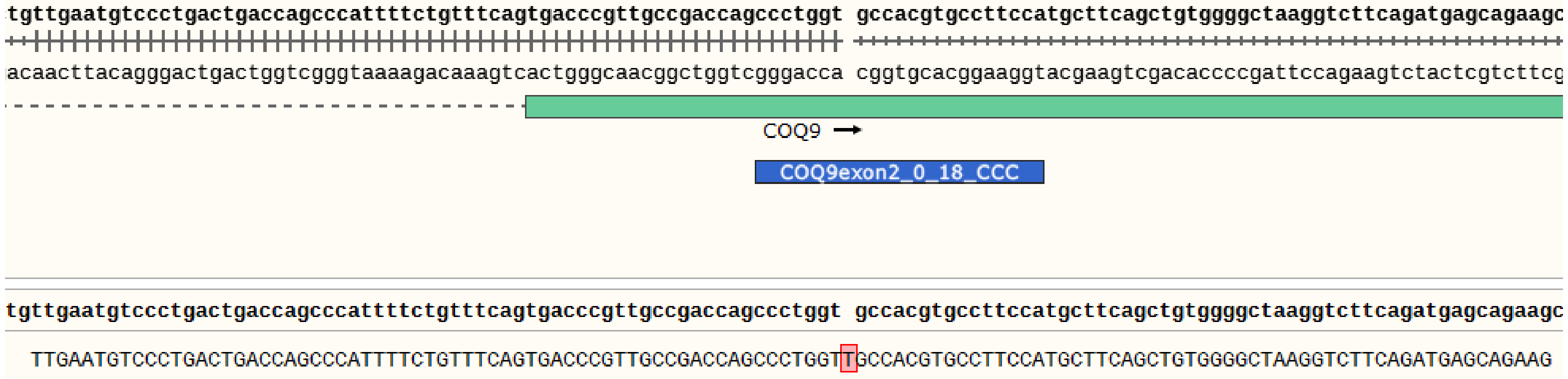

Name	Clone ID	Total # reads	# wt reads(%)	#1-Indel	#1-Reads(%)	#2-Indel	#2-Reads(%)
CNTCOQ DKO data CNTD2 2D11		1285	0 (0.0%)	-13	659 (51.3%)	-38	602 (46.8%)
CNTCOQ DKO data COQ9 2D11	038D	1809	0 (0.0%)	1	1780 (98.4%)	0	26 (1.4%)

Clone 038D (-13/-38 CNTD2; +1/+1 COQ9)

CNTD2: NGS 1, NGS 2 (cell bank)

COQ9: NGS 1, direct sequencing, NGS 2 (cell bank)

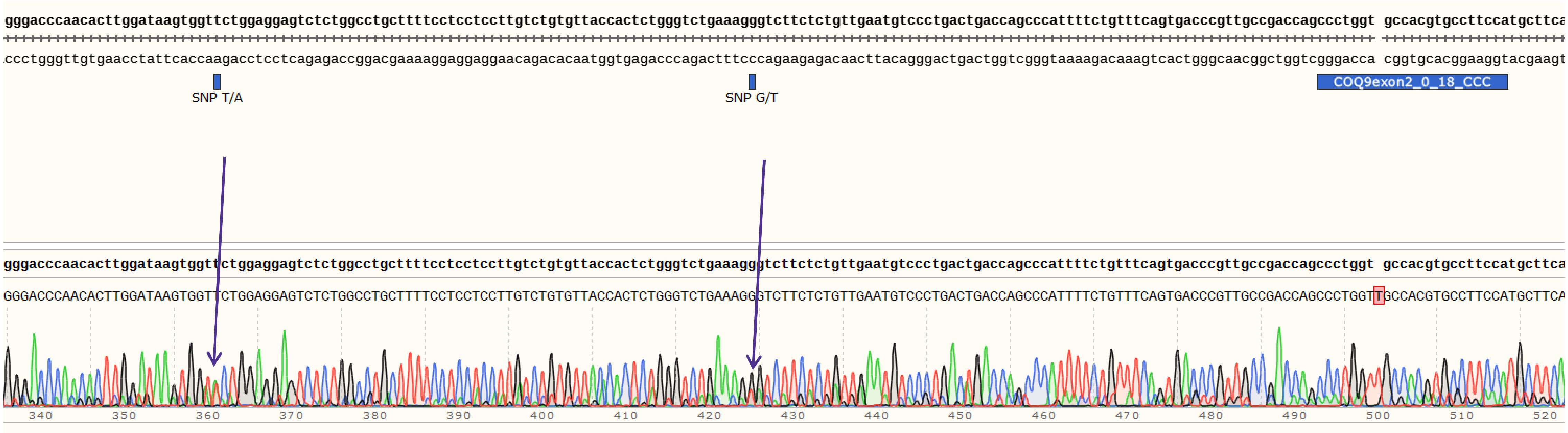

Clone ID	Clone ID	Total # reads	# wt reads(%)	#1-Indel	#1-Reads(%)	#2-Indel	#2-Reads(%)
CNTCOQ DKO data CNTD2 2D11		1572	0 (0.0%)	-38	794 (50.5%)	-13	759 (48.3%)
CNTCOQ DKO data COQ9 2D11	038D	5566	0 (0.0%)	1	5368 (96.4%)	0	163 (2.9%)

Clone 039D (-79/-1 CNTD2; +1/+1 COQ9)

CNTD2: NGS 1, NGS 2 (cell bank)

COQ9: NGS 1, direct sequencing, NGS 2 (cell bank)

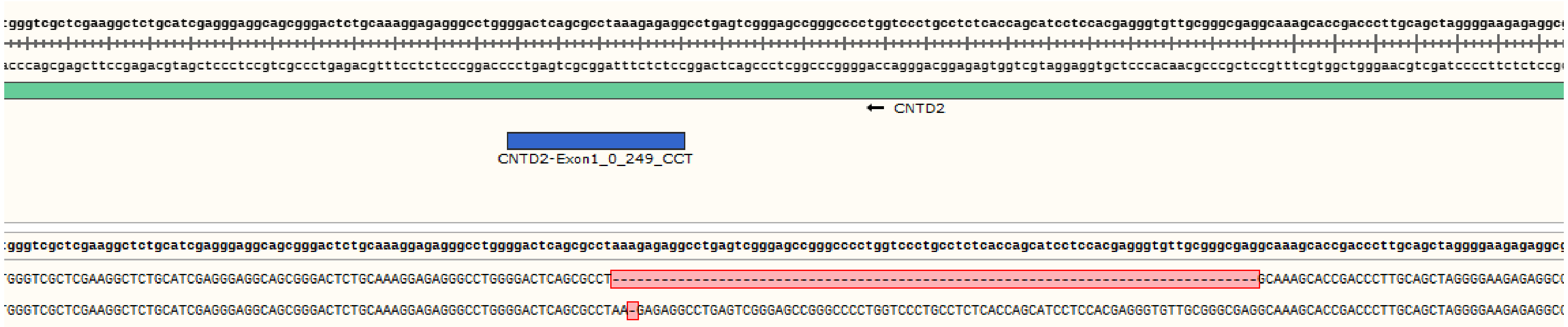

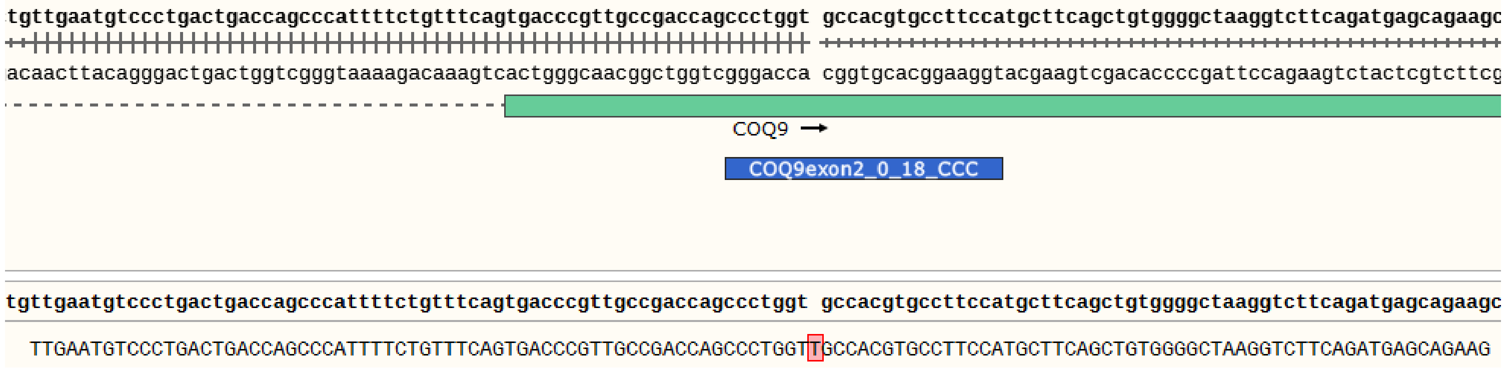

Name	Clone ID	Total # reads	# wt reads(%)	#1-Indel	#1-Reads(%)	#2-Indel	#2-Reads(%)
CNTCOQ DKO data CNTD2 3B11		1766	0 (0.0%)	-79	1036 (58.7%)	-1	686 (38.8%)
CNTCOQ DKO data COQ9 3B11	039D	1348	0 (0.0%)	1	1326 (98.4%)	0	21 (1.6%)

Clone 039D (-79/-1 CNTD2; +1/+1 COQ9)

CNTD2: NGS 1, NGS 2 (cell bank)

COQ9: NGS 1, direct sequencing, NGS 2 (cell bank)

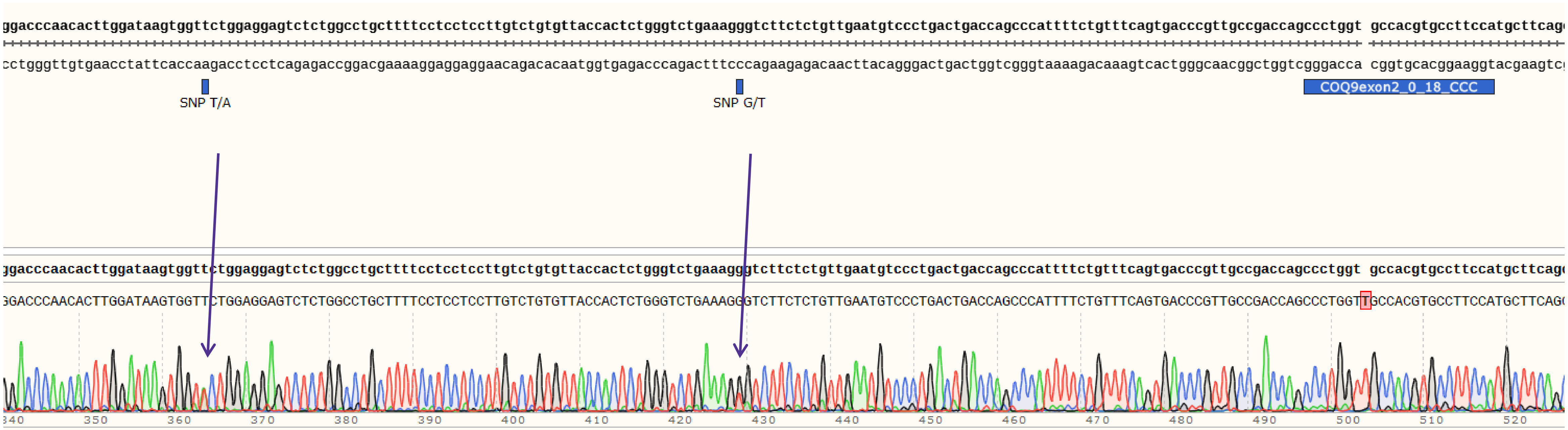

Clone ID	Clone ID	Total # reads	# wt reads(%)	#1-Indel	#1-Reads(%)	#2-Indel	#2-Reads(%)
CNTCOQ DKO data CNTD2 3B11		537	0 (0.0%)	-1	526 (98.0%)	-2	9 (1.7%)
CNTCOQ DKO data COQ9 3B11	039D	3806	0 (0.0%)	1	3682 (96.7%)	0	110 (2.9%)

Note: the NGS provider used different primers for CNTD2 from NGS run 1 to NGS run 2. The -79bp overlaps the 2nd primer pair and therefore was not resolved in NGS run 2, however all reads were -1 as expected with this primer pair.

Clone 025D (-32/-11 CNTD2; -82/-82/+1 NAT9)

CNTD2: NGS 1, NGS 2 (cell bank)

NAT9: NGS 1, NGS 2 (cell bank)

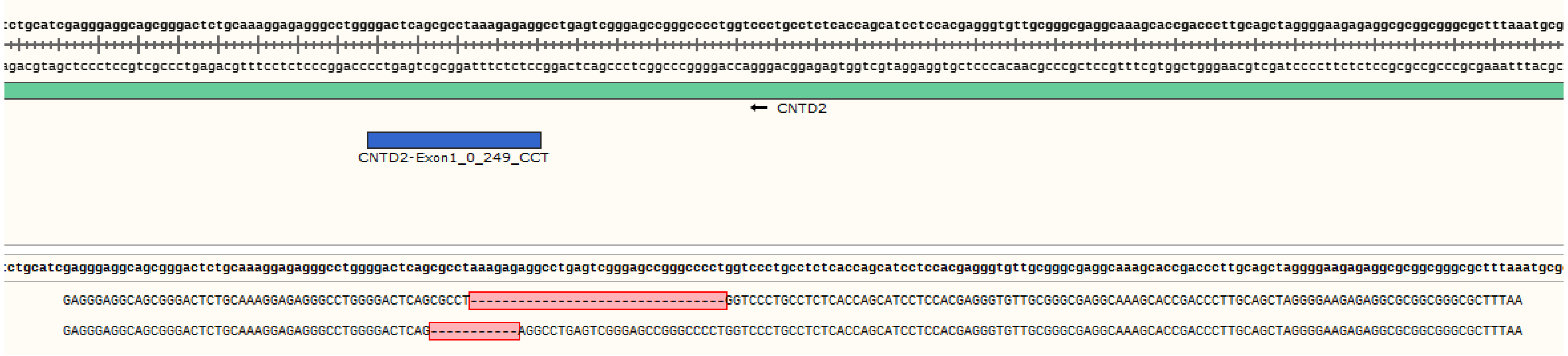

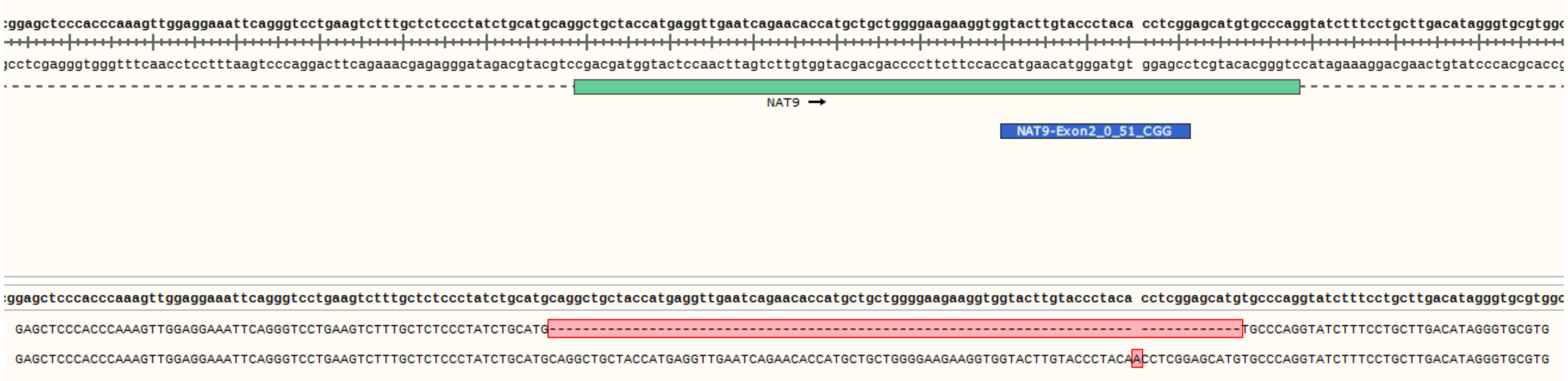

Name	Clone ID	Total # reads	# wt reads(%)	#1-Indel	#1-Reads(%)	#2-Indel	#2-Reads(%)	#3-Indel	#3-Reads(%)
CNTNAT DKO data CNTD2 1C6		376	0 (0.0%)	-32	222 (59.0%)	-11	147 (39.1%)	-12	5 (1.3%)
CNTNAT DKO data NAT9 1C6	025D	1319	2 (0.2%)	-82	801 (60.7%)	1	502 (38.1%)	0	9 (0.7%)

Clone ID	Clone ID	Total # reads	# wt reads(%)	#1-Indel	#1-Reads(%)	#2-Indel	#2-Reads(%)	#3-Indel	#3-Reads(%)
CNTNAT DKO data CNTD2 1C6		1318	0 (0.0%)	-32	752 (57.1%)	-11	551 (41.8%)	-12	11 (0.8%)
CNTNAT DKO data NAT9 1C6	025D	1813	0 (0.0%)	-82	1197 (66.0%)	1	577 (31.8%)	-83	24 (1.3%)

Clone 027D (-13/-11 CNTD2; -19/-1/+1 NAT9)

CNTD2: NGS 1, NGS 2 (cell bank)

NAT9: NGS 1, NGS 2 (cell bank)

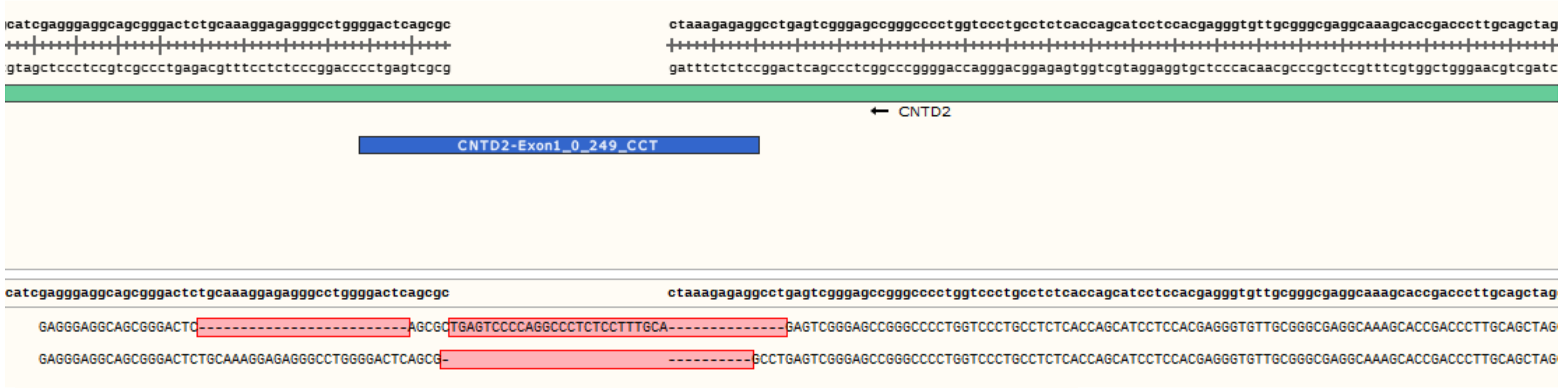

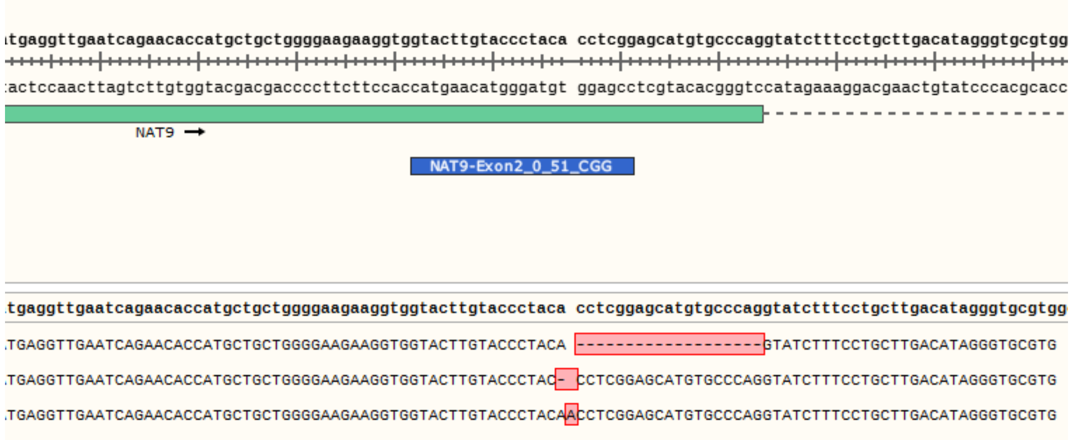

Name	Clone ID	Total # reads	# wt reads(%)	#1-Indel	#1-Reads(%)	#2-Indel	#2-Reads(%)	#3-Indel	#3-Reads(%)
CNTNAT DKO data CNTD2 3C11		212	0 (0.0%)	-13	107 (50.5%)	-11	98 (46.2%)	-12	5 (2.4%)
CNTNAT DKO data NAT9 3C11	027D	1530	0 (0.0%)	-19	541 (35.4%)	-1	488 (31.9%)	1	480 (31.4%)

Clone ID	Clone ID	Total # reads	# wt reads(%)	#1-Indel	#1-Reads(%)	#2-Indel	#2-Reads(%)	#3-Indel	#3-Reads(%)
CNTNAT DKO data CNTD2 3C11		1421	0 (0.0%)	-13	767 (54.0%)	-11	633 (44.5%)	-12	10 (0.7%)
CNTNAT DKO data NAT9 3C11	027D	1530	0 (0.0%)	-19	613 (40.1%)	1	448 (29.3%)	-1	433 (28.3%)

Clone 034D (-35/+1 CNTD2; -1/-2/-328 NAT9)

CNTD2: NGS 1, NGS 2 (cell bank)

NAT9: NGS 1, fragment analysis, topo sequencing, NGS 2 (cell bank)

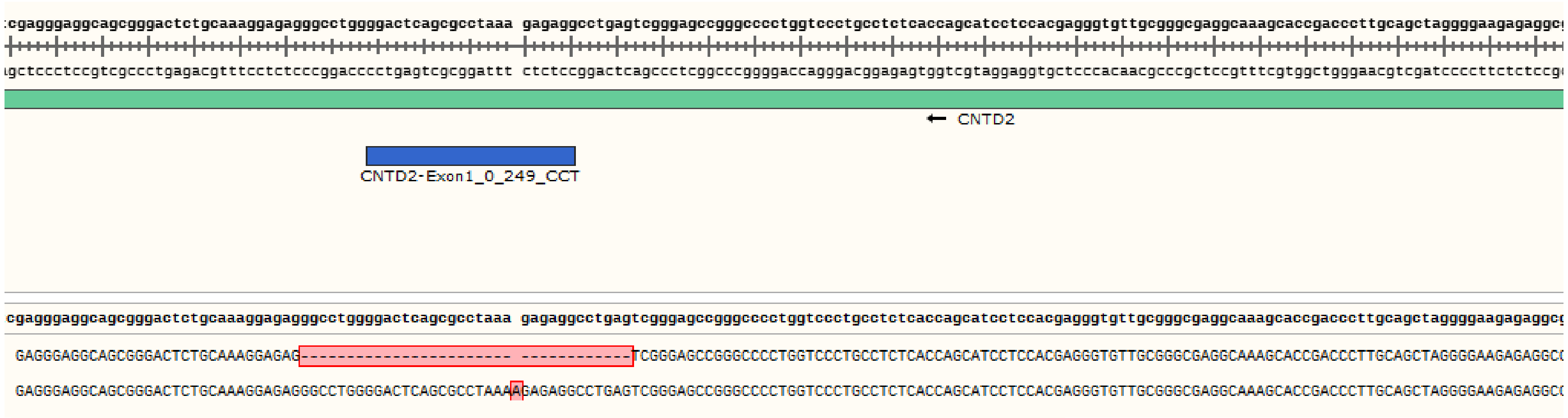

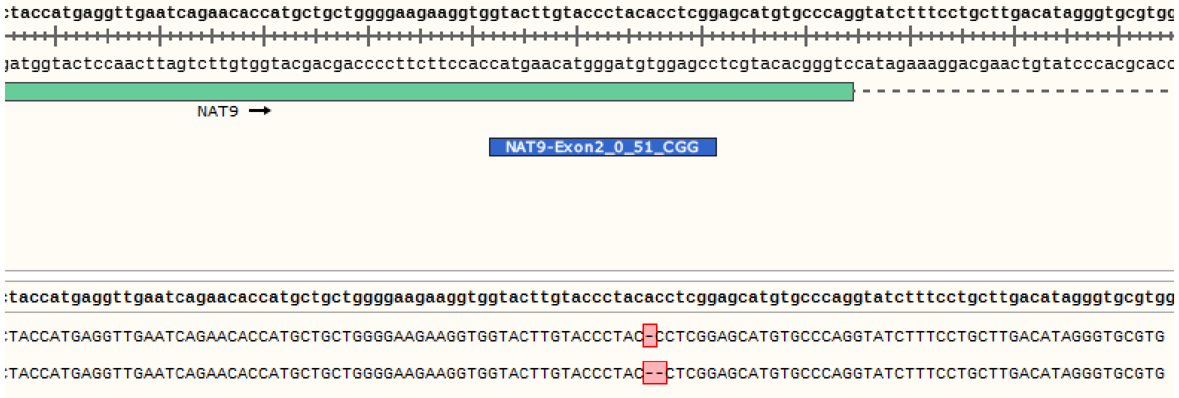

Name	Clone ID	Total # reads	# wt reads(%)	#1-Indel	#1-Reads(%)	#2-Indel	#2-Reads(%)	#3-Indel	#3-Reads(%)
CNTNAT DKO data CNTD2 1H2		91	0 (0.0%)	-35	52 (57.1%)	1	36 (39.6%)	0	1 (1.1%)
CNTNAT DKO data NAT9 1H2	034D	871	0 (0.0%)	-1	460 (52.8%)	-2	405 (46.5%)	-3	6 (0.7%)

Clone 034D (-35/+1 CNTD2; -1/-2/-328 NAT9)

CNTD2: NGS 1, NGS 2 (cell bank)

NAT9: NGS 1, fragment analysis, topo sequencing, NGS 2 (cell bank)

NAT9 fragment analysis

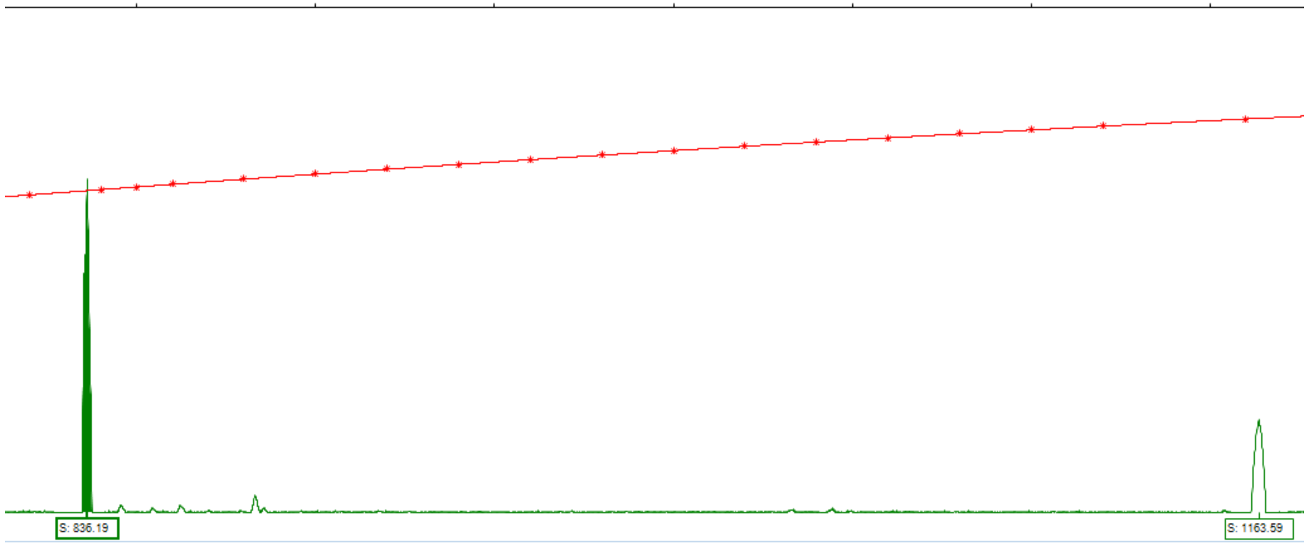

Clone 034D (-35/+1 CNTD2; -1/-2/-328 NAT9)

CNTD2: NGS 1, NGS 2 (cell bank)

NAT9: NGS 1, fragment analysis, topo sequencing, NGS 2 (cell bank)

NAT9 -328bp

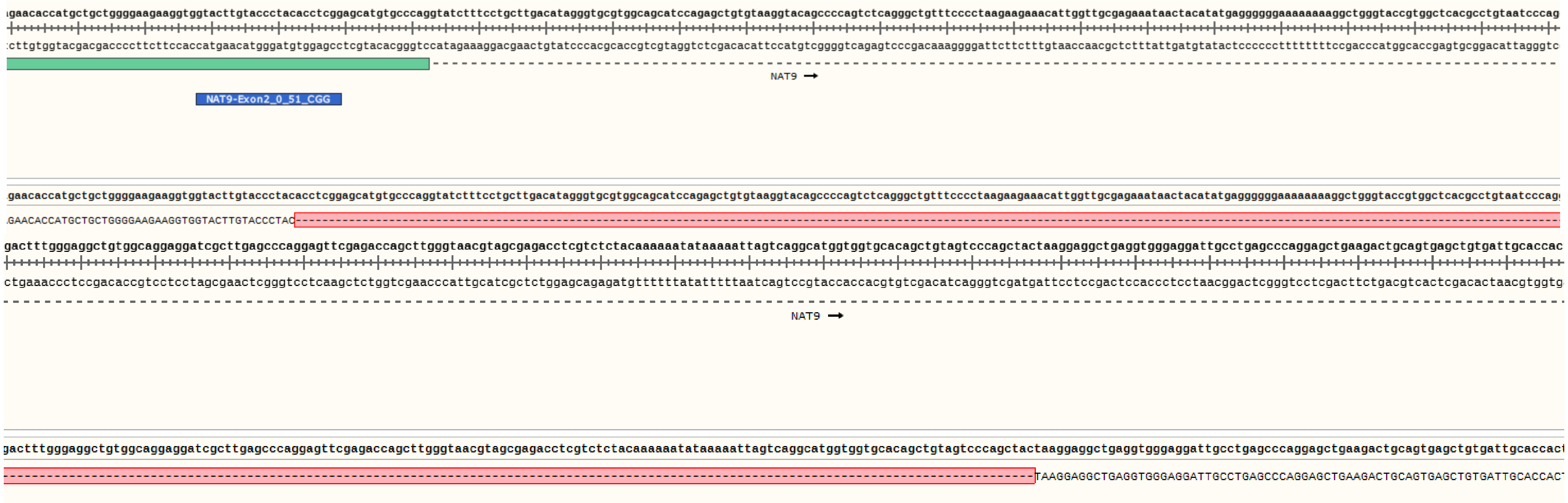

NAT9 deleted sequence:

acctcggagcatgtgcccaggtatctttcctgcttgacatagggtgcgtggcagcatccagagctgtgtaaggtacagccccagtctcagggctgtttcccctaagaagaaac
attggttgcgagaaataactacatatgagggggaaaaaaaaggctgggtaccgtggctcacgcctgtaatcccaggactttgggaggctgtggcaggaggatcgcttga
gcccaggagttcgagaccagcttgggtaacgtagcgagacctcgtctctacaaaaaatataaaaattagtcaggcatggtggtgcacagctgtagtcccagctac

Clone ID	Clone ID	Total # reads	# wt reads(%)	#1-Indel	#1-Reads(%)	#2-Indel	#2-Reads(%)	#3-Indel	#3-Reads(%)
CNTNAT DKO data CNTD2 1H2		1783	5 (0.3%)	-35	1006 (56.4%)	1	737 (41.3%)	0	20 (1.1%)
CNTNAT DKO data NAT9 1H2	034D	1299	1 (0.1%)	-2	673 (51.8%)	-1	603 (46.4%)	-3	18 (1.4%)

Clone 035D (+1/-232 CNTD2; +1/+1/-1 NAT9)

CNTD2: NGS 1, fragment analysis, topo sequencing, NGS 2 (cell bank)

NAT9: NGS 1, NGS 2 (cell bank)

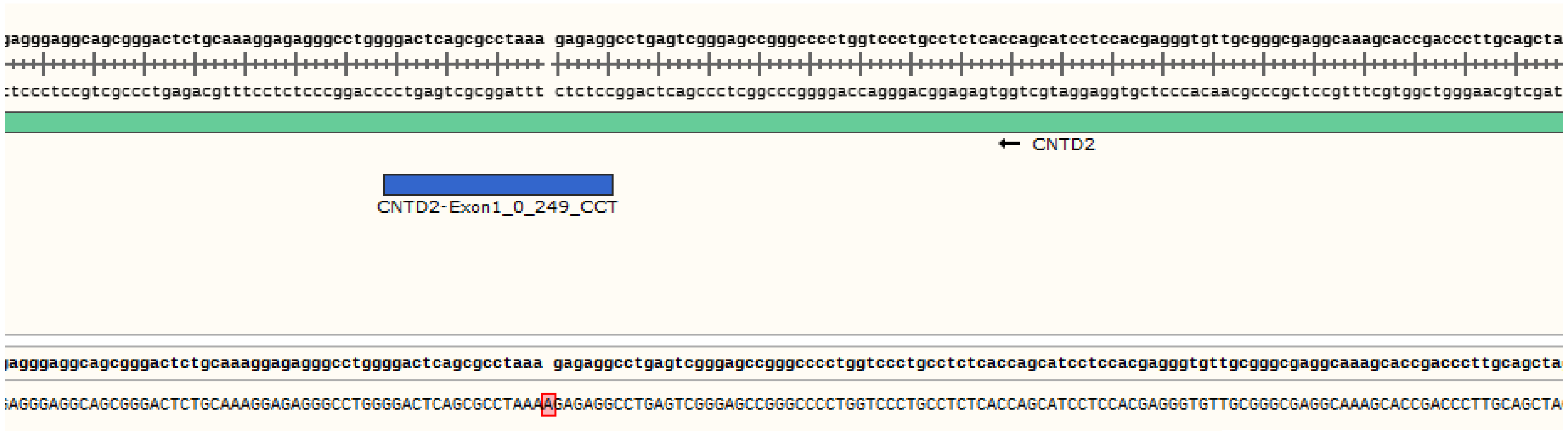

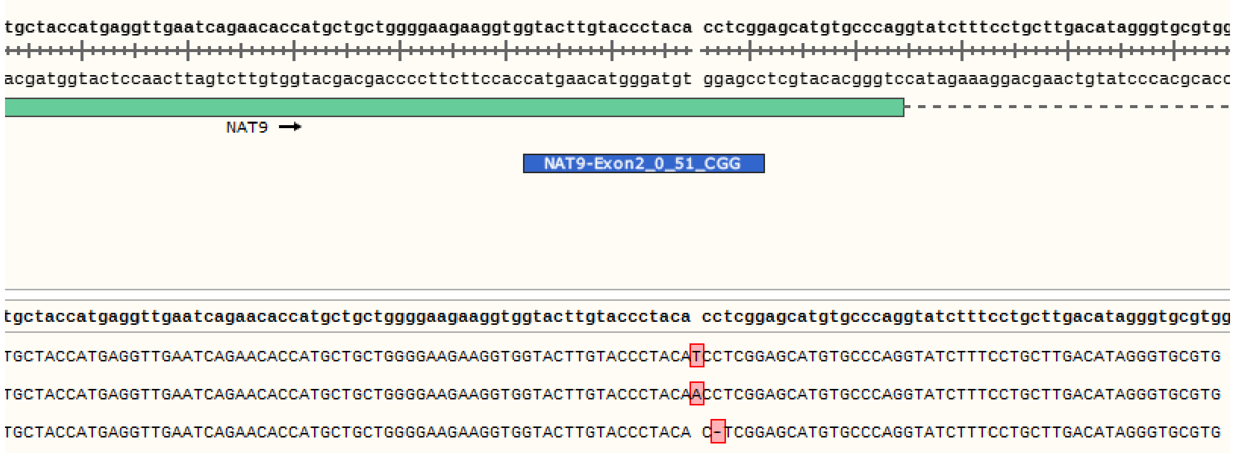

Name	Clone ID	Total # reads	# wt reads(%)	#1-Indel	#1-Reads(%)	#2-Indel	#2-Reads(%)	#3-Indel	#3-Reads(%)
CNTNAT DKO data CNTD2 1H9		83	1 (1.2%)	1	82 (98.8%)	0	1 (1.2%)	NA	
CNTNAT DKO data NAT9 1H9	035D	1503	0 (0.0%)	1	1000 (66.5%)	-1	474 (31.5%)	0	20 (1.3%)

Clone 035D (+1/-232 CNTD2; +1/+1/-1 NAT9)

CNTD2: NGS 1, fragment analysis, topo sequencing, NGS 2 (cell bank)

NAT9: NGS 1, NGS 2 (cell bank)

CNTD2 fragment analysis

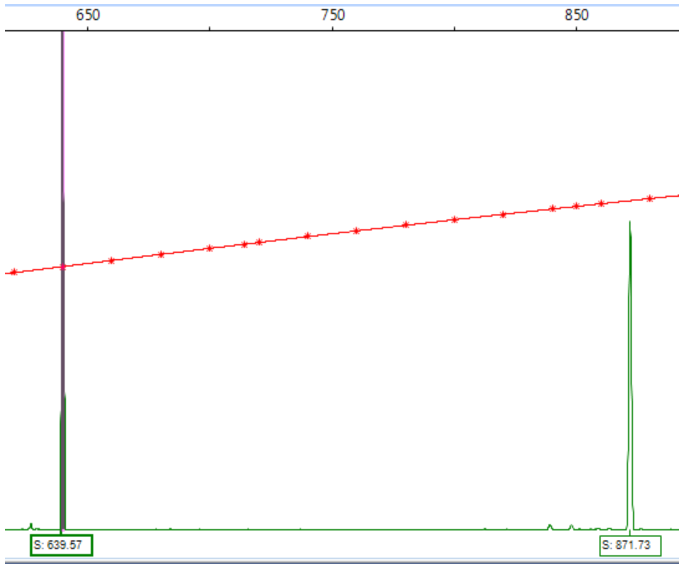

Clone 035D (+1/-232 CNTD2; +1/+1/-1 NAT9)

CNTD2: NGS 1, fragment analysis, topo sequencing, NGS 2 (cell bank)

NAT9: NGS 1, NGS 2 (cell bank)

CNTD2 -232

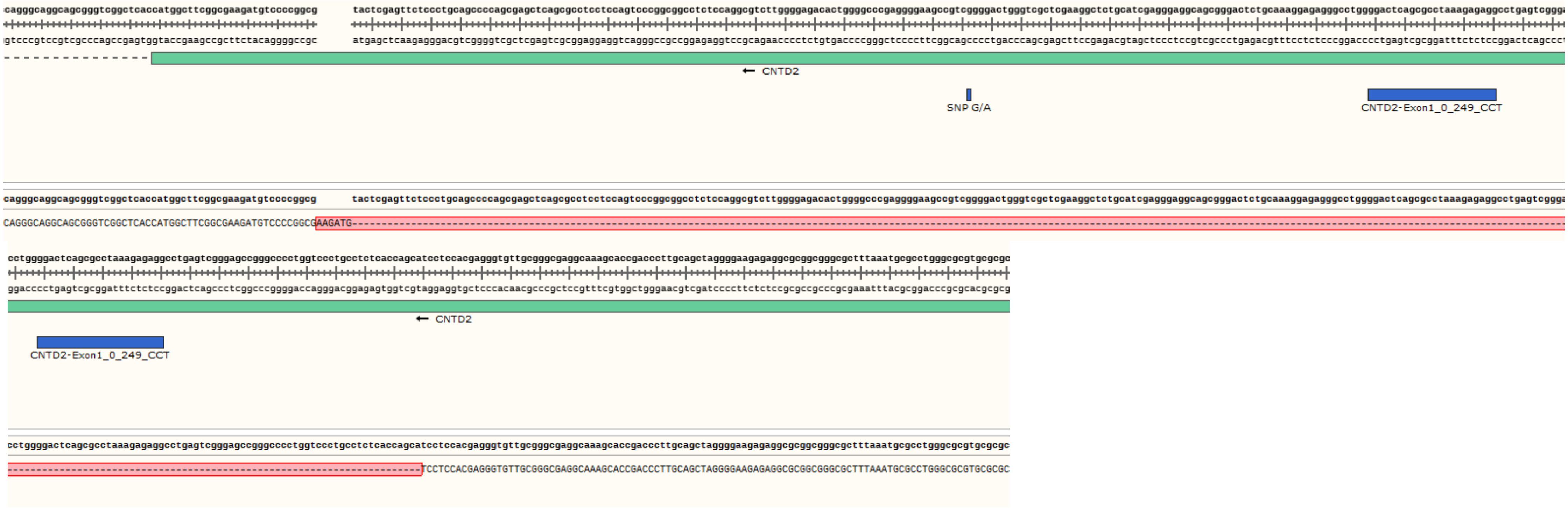

CNTD2 deleted sequence:

tactcgagttctccctgcagccccagcgagctcagcgcctcctccagtcccggcggcctctccaggcgtcttggggagacactggggcccga
ggggaagccgtcggggactgggtcgctcgaaggctctgcatcgagggaggcagcgggactctgcaaaggagagggcctggggactca
gcgcctaaagagaggcctgagtcgggagccgggcccctggtccctgcctctcaccagca

Clone ID	Clone ID	Total # reads	# wt reads(%)	#1-Indel	#1-Reads(%)	#2-Indel	#2-Reads(%)	#3-Indel	#3-Reads(%)
CNTNAT DKO data CNTD2 1H9		1247	1 (0.1%)	1	1209 (97.0%)	0	34 (2.7%)	2	2 (0.2%)
CNTNAT DKO data NAT9 1H9	035D	1179	0 (0.0%)	1	765 (64.9%)	-1	384 (32.6%)	0	15 (1.3%)

Clone 044D (-268 CNTD2; -49/-272 NAT9)

CNTD2: NGS 1, sequencing, NGS 2
NAT9: NGS 1, fragment analysis, 3kb PCR, topo sequencing, NGS 2

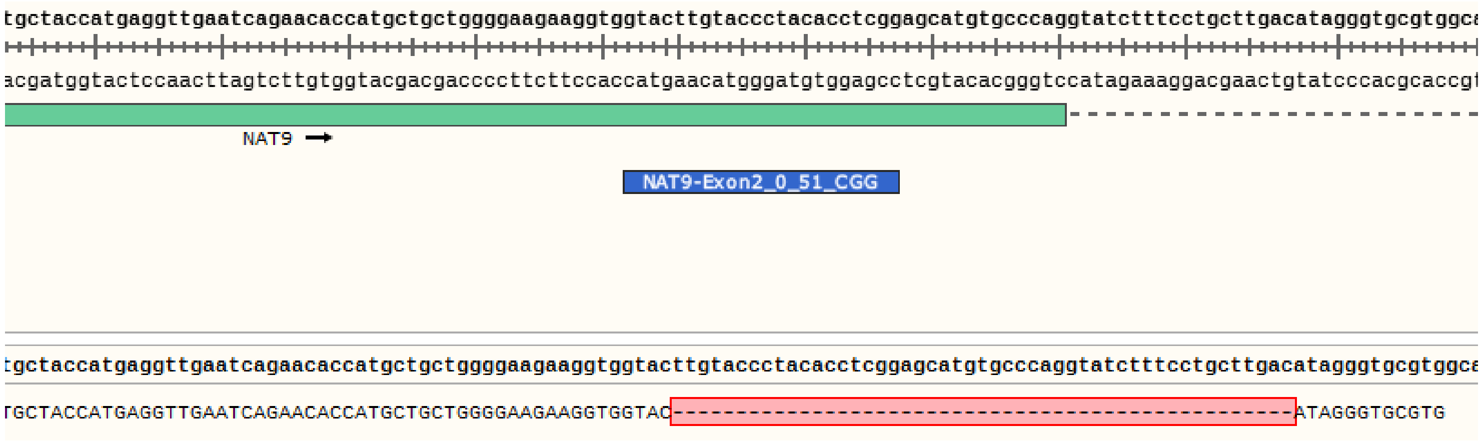

Name	Clone ID	Total # reads	# wt reads(%)	#1-Indel	#1-Reads(%)	#2-Indel	#2-Reads(%)	#3-Indel	#3-Reads(%)
CNTNAT DKO data CNTD2 2B4		no data							
CNTNAT DKO data NAT9 2B4	044D	496	0 (0.0%)	-49	489 (98.6%)	1	3 (0.6%)	-50	3 (0.6%)

Clone 044D (-268 CNTD2; -49/-272 NAT9)

CNTD2: NGS 1, sequencing, NGS 2
NAT9: NGS 1, fragment analysis, 3kb PCR, topo sequencing, NGS 2

CNTD2 sequencing -268

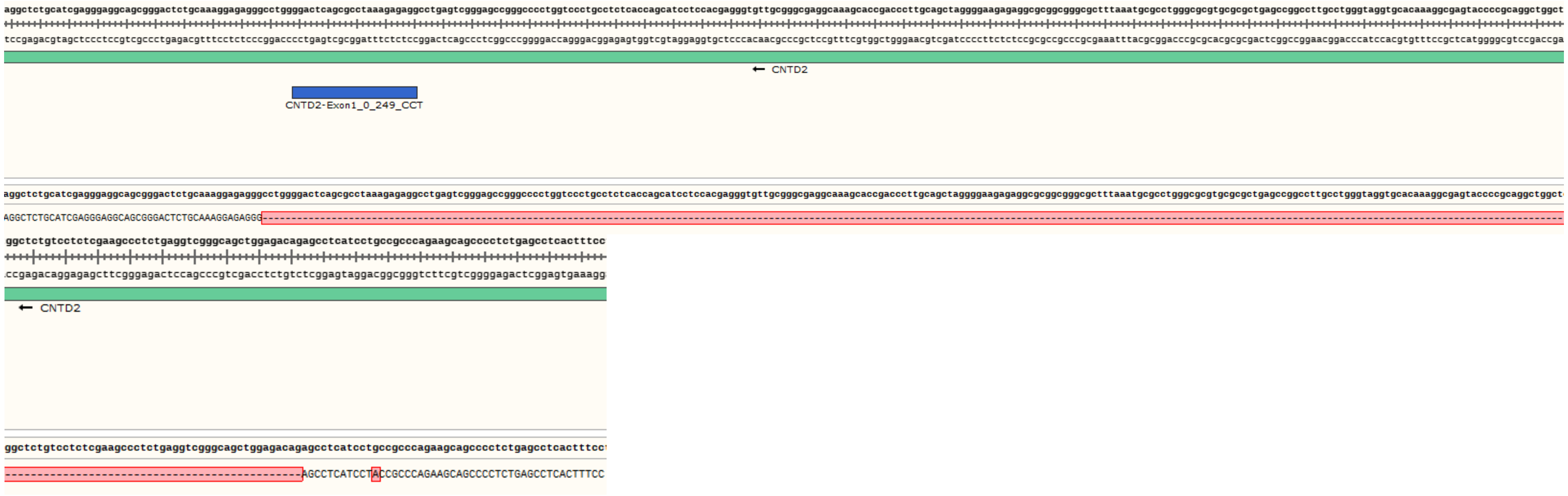

CNTD2 deleted sequence

cctggggactcagcgcctaaagagaggcctgagtcgggagccgggcccctggtccctgcctctcaccagcatcctccacgagggtgttgcgggcgaggcaaagcaccgacccttgcagctaggggaagaga
ggcgcggcgggcgctttaaatgcgcctgggcgcgtgcgcgctgagccggccttgcctgggtaggtgcacaaaggcgagtaccccgcaggctggctctgtcctctcgaagccctctgaggtcgggcagctgga
gacag

Clone 044D (-268 CNTD2; -49/-272 NAT9)

CNTD2: NGS 1, sequencing, NGS 2
NAT9: NGS 1, fragment analysis, 3kb PCR, topo sequencing, NGS 2

NAT9 fragment analysis

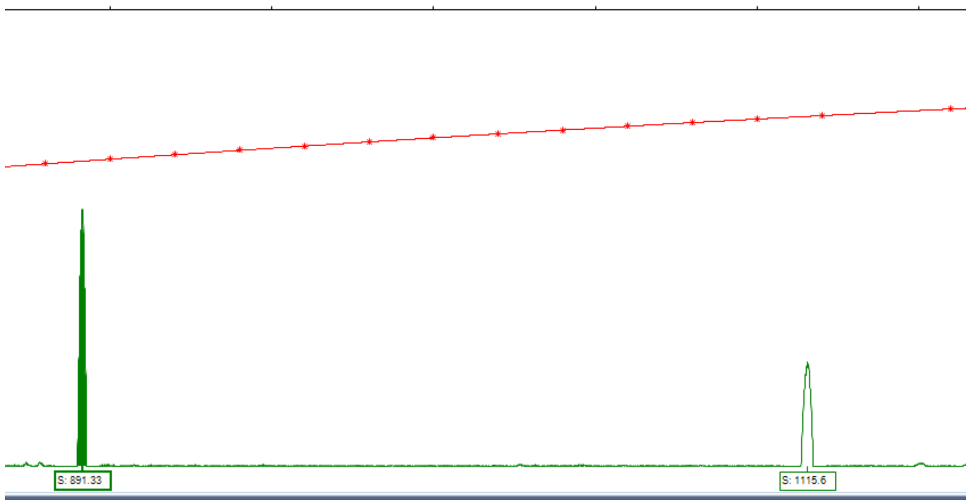

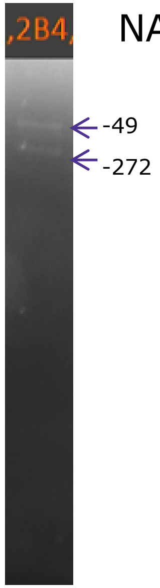

NAT9 3kb PCR

Clone 044D (-268 CNTD2; -49/-272 NAT9)

CNTD2: NGS 1, sequencing, NGS 2
NAT9: NGS 1, fragment analysis, 3kb PCR, topo sequencing, NGS 2

NAT9 sequencing -272

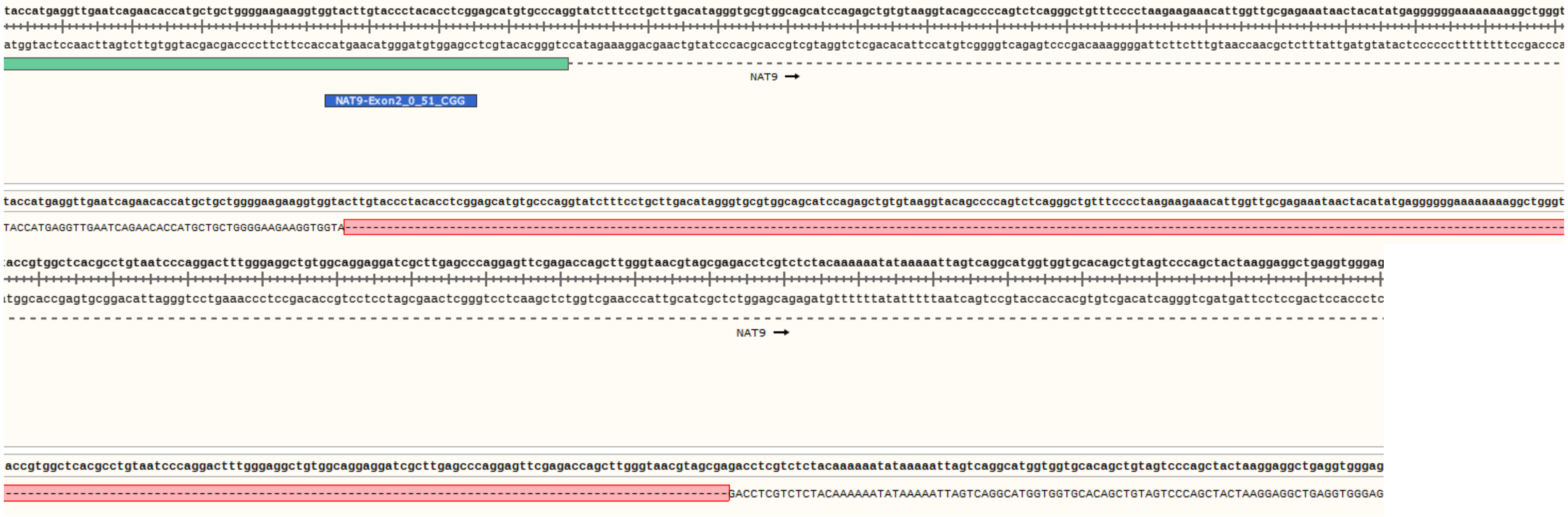

NAT9 deleted sequence

cttgtaccctacacctcggagcatgtgcccaggtatctttcctgcttgacatagggtgcgtggcagcatccagagctgtgtaaggtacagccccagtctcagggctgtttcccctaagaagaaa
cattggttgcgagaaataactacatatgagggggaaaaaaaaggctgggtaccgtggctcacgcctgtaatcccaggactttgggaggctgtggcaggaggatcgcttgagcccaggag
ttcgagaccagcttgggtaacgtagcga

Clone ID	Clone ID	Total # reads	# wt reads(%)	#1-Indel	#1-Reads(%)	#2-Indel	#2-Reads(%)	#3-Indel	#3-Reads(%)
CNTNAT DKO data CNTD2 2B4		no data - large deletions							
CNTNAT DKO data NAT9 2B4	044D	1820	0 (0.0%)	-49	1776 (97.6%)	-50	39 (2.1%)	-48	3 (0.2%)

Clone 077D (+14/-17 CNTD2; -7/-13/-1 NAT9)

CNTD2: NGS 1, NGS 2 (cell bank)

NAT9: NGS 1, NGS 2 (cell bank)

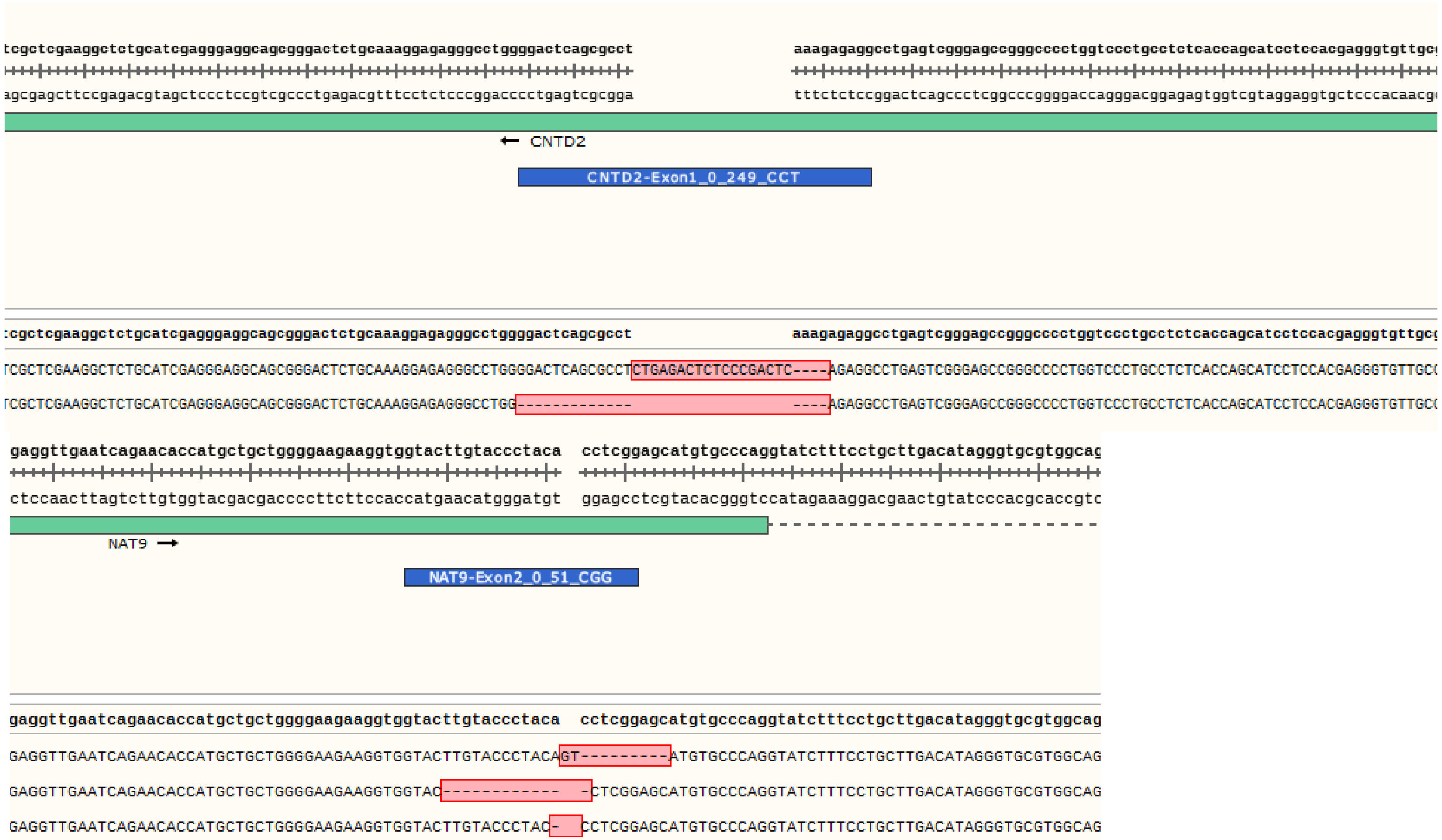

Name	Clone ID	Total # reads	# wt reads(%)	#1-Indel	#1-Reads(%)	#2-Indel	#2-Reads(%)	#3-Indel	#3-Reads(%)
CNTNAT DKO data CNTD2 1D11		3176	8 (0.3%)	14	1633 (51.4%)	-17	1482 (46.7%)	13	26 (0.8%)
CNTNAT DKO data NAT9 1D11	077D	2115	3 (0.1%)	-7	776 (36.7%)	-13	691 (32.7%)	-1	582 (27.5%)

Clone ID	Clone ID	Total # reads	# wt reads(%)	#1-Indel	#1-Reads(%)	#2-Indel	#2-Reads(%)	#3-Indel	#3-Reads(%)
CNTNAT DKO data CNTD2 1D11		11251	21 (0.2%)	-17	6048 (53.8%)	14	4901 (43.6%)	-18	138 (1.2%)
CNTNAT DKO data NAT9 1D11	077D	4356	0 (0.0%)	-13	1551 (35.6%)	-7	1380 (31.7%)	-1	1300 (29.8%)

Clone 078D (-16/-1 CNTD2; -2/-2/-5 NAT9)

CNTD2: NGS 1, NGS 2 (cell bank)

NAT9: NGS 1, NGS 2 (cell bank)

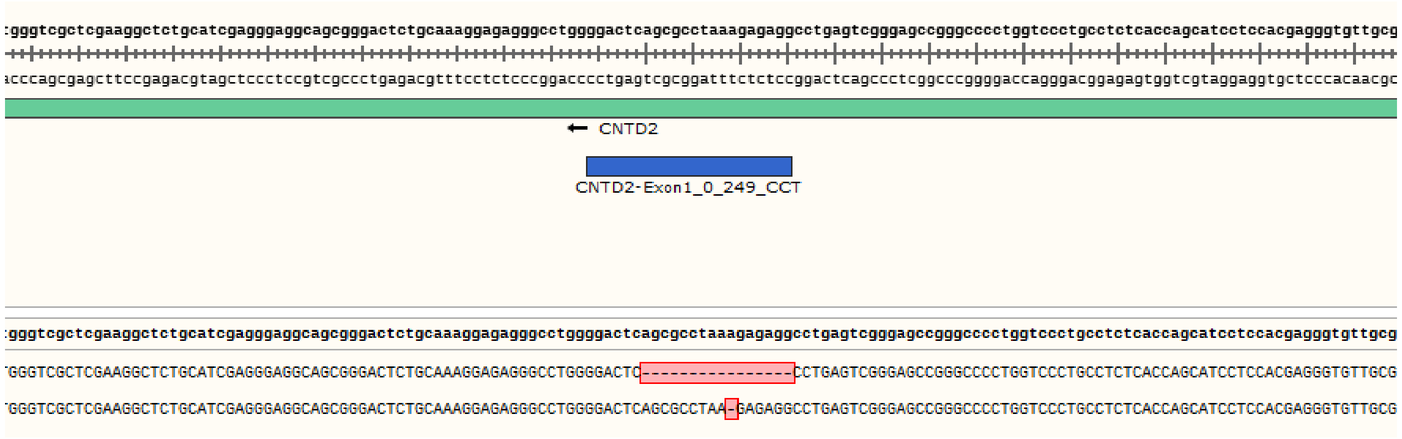

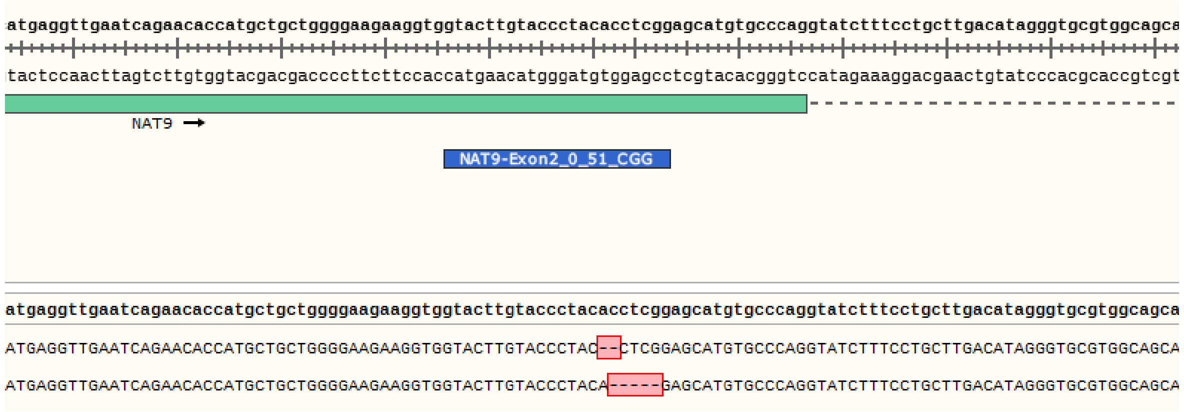

Name	Clone ID	Total # reads	# wt reads(%)	#1-Indel	#1-Reads(%)	#2-Indel	#2-Reads(%)	#3-Indel	#3-Reads(%)
CNTNAT DKO data CNTD2 2D6		3008	0 (0.0%)	-16	1464 (48.7%)	-1	1446 (48.1%)	-17	45 (1.5%)
CNTNAT DKO data NAT9 2D6	078D	2049	1 (0.0%)	-2	1307 (63.8%)	-5	672 (32.8%)	-3	39 (1.9%)

Clone ID	Clone ID	Total # reads	# wt reads(%)	#1-Indel	#1-Reads(%)	#2-Indel	#2-Reads(%)	#3-Indel	#3-Reads(%)
CNTNAT DKO data CNTD2 2D6		13302	0 (0.0%)	-1	6580 (49.5%)	-16	6428 (48.3%)	-2	137 (1.0%)
CNTNAT DKO data NAT9 2D6	078D	6016	0 (0.0%)	-2	3844 (63.9%)	-5	2013 (33.5%)	-3	80 (1.3%)

Clone 048D (-89/-814 CNTD2; -22/-128 NEU2)

CNTD2: NGS 1, 3kb PCR, sequencing, NGS 2 (cell bank)

NEU2: NGS 1, topo sequencing, NGS 2 (cell bank)

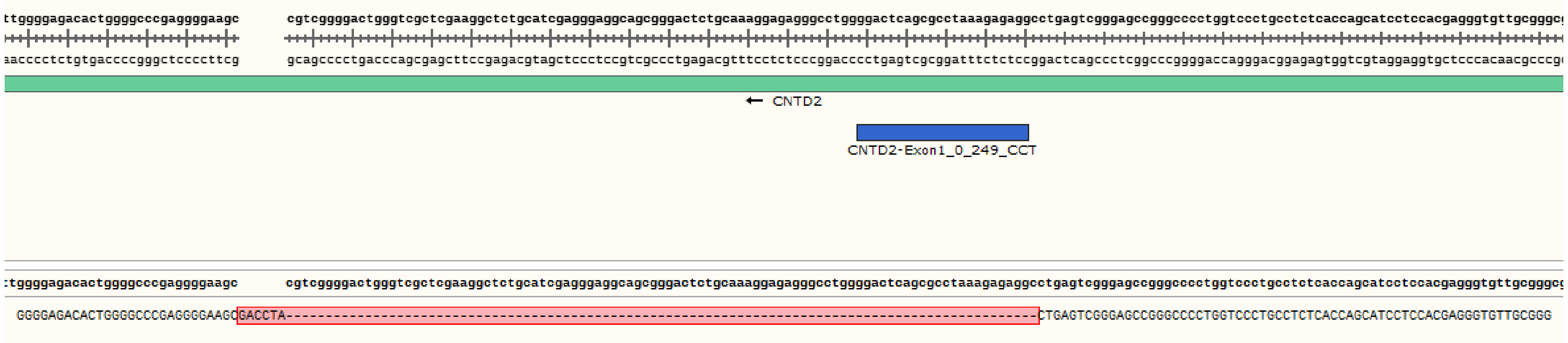

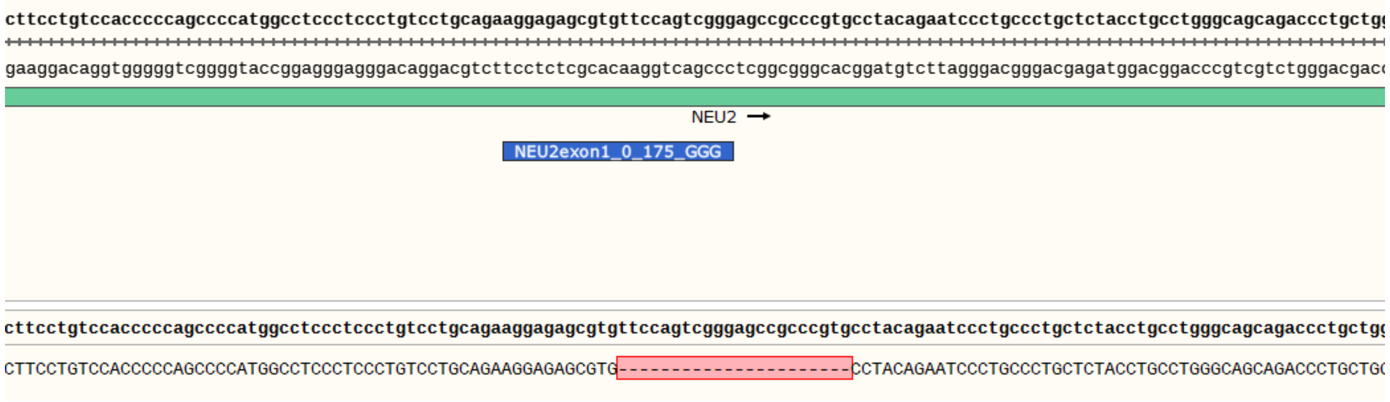

Name	Clone ID	Total # reads	# wt reads(%)	#1-Indel	#1-Reads(%)	#2-Indel	#2-Reads(%)
CNTNEU DKO data CNTD2 2B6		1620	0 (0.0%)	-89	1604 (99.0%)	-90	13 (0.8%)
CNTNEU DKO data NEU2 2B6	048D	778	5 (0.6%)	-22	750 (96.4%)	-23	20 (2.6%)

Clone 048D (-89/-814 CNTD2; -22/-128 NEU2)

CNTD2: NGS 1, 3kb PCR, sequencing, NGS 2 (cell bank)

NEU2: NGS 1, topo sequencing, NGS 2 (cell bank)

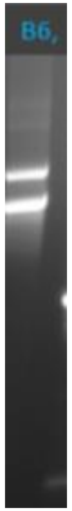

CNTD2 3kb PCR

Clone 048D (-89/-814 CNTD2; -22/-128 NEU2)

CNTD2: NGS 1, 3kb PCR, sequencing, NGS 2 (cell bank)

NEU2: NGS 1, topo sequencing, NGS 2 (cell bank)

CNTD2 -814

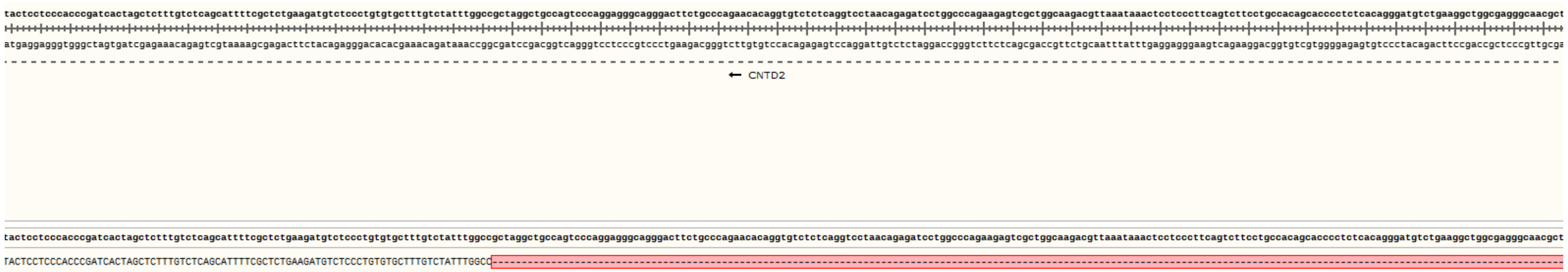

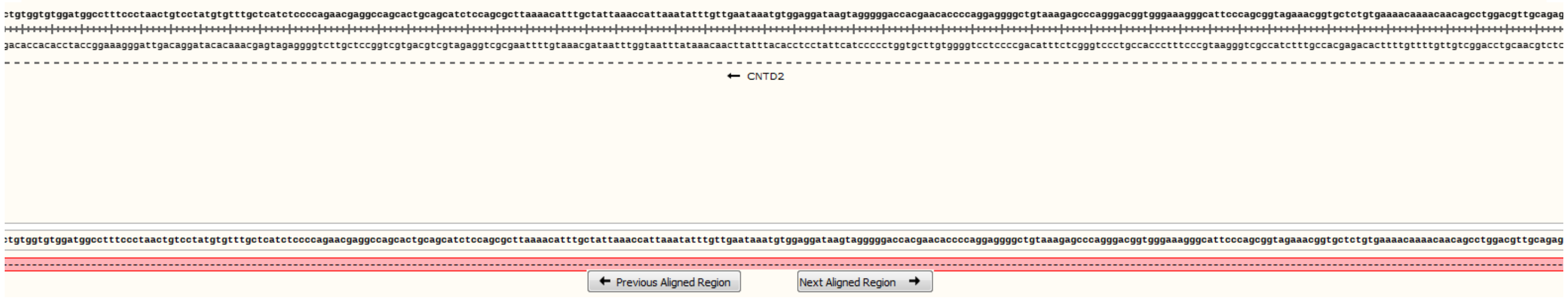

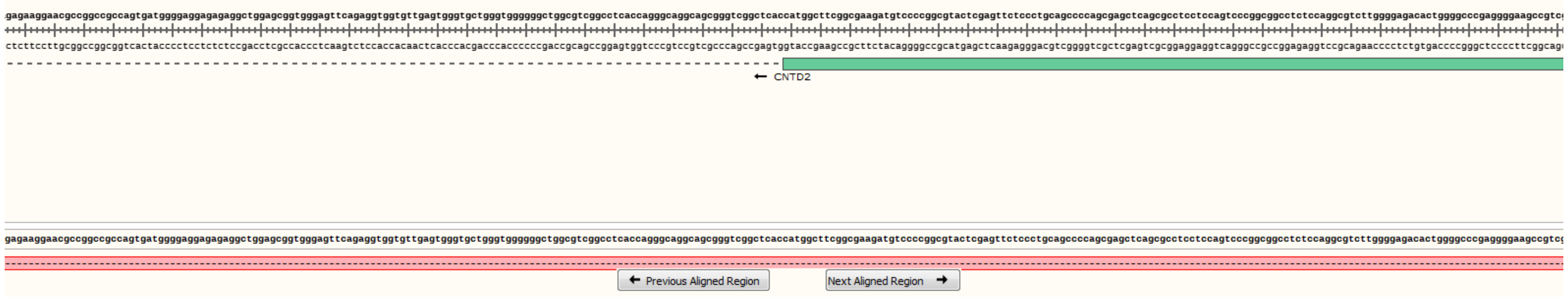

Clone 048D (-89/-814 CNTD2; -22/-128 NEU2)

CNTD2: NGS 1, 3kb PCR, sequencing, NGS 2 (cell bank)

NEU2: NGS 1, topo sequencing, NGS 2 (cell bank)

CNTD2 -814 continued

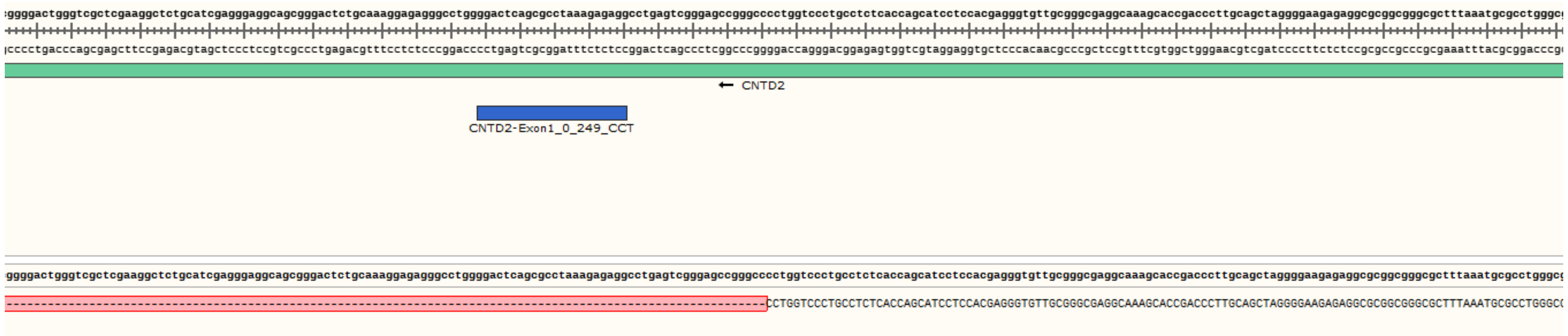

CNTD2 deleted sequence:

gctaggctgccagtcccaggagggcagggacttctgcccagaacacaggtgtctctcaggtcctaacagagatcctggcccagaagagtcgctggcaagacgttaaataaactcctcccttcagtcttcctgccacagcacccctctcaca
gggatgtctgaaggctggcgagggcaacgctgtggtgtggatggcctttccctaactgtcctatgtgtttgctcatctccccagaacgaggccagcactgcagcatctccagcgcttaaaacatttgctattaaaccattaaatatttgttgaat
aaatgtggaggataagtaggggggaccacgaacaccccaggaggggctgtaaagagcccagggacggtgggaaagggcattcccagcggtagaaacggtgctctgtgaaaacaaaacaacagcctggacgttgcagagaaggaac
gccggccgccagtgatggggaggagagaggctggagcggtgggagttcagaggtggtgttgagtgggtgctgggtggggggctggcgtcggcctcaccagggcaggcagcgggtcggctcaccatggcttcggcgaagatgtcccc
ggcgtactcgagttctccctgcagccccagcgagctcagcgcctcctccagtcccggcggcctctccaggcgtcttggggagacactggggcccgaggggaagccgtcggggactgggtcgctcgaaggctctgcatcgagggaggca
gcgggactctgcaaaggagagggcctggggactcagcgcctaaagagaggcctgagtcgggagccgggcc

Clone 048D (-89/-814 CNTD2; -22/-128 NEU2)

CNTD2: NGS 1, 3kb PCR, sequencing, NGS 2 (cell bank)

NEU2: NGS 1, topo sequencing, NGS 2 (cell bank)

NEU2 -128

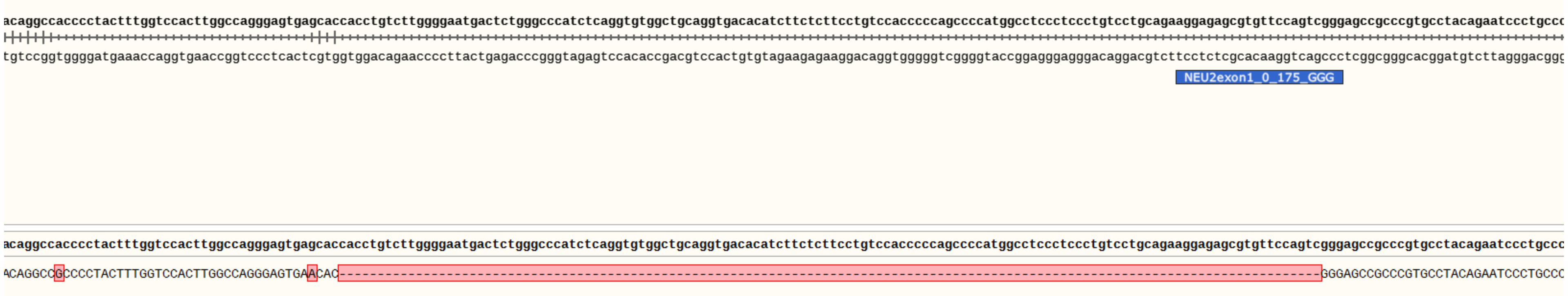

NEU2 deleted sequence:

cacctgtcttggggaatgactctgggcccatctcaggtgtggctgcaggtgacacatcttctcttcctgtccacccccagccccatggcctccctccctgtcctgcag
aaggagagcgtgttccagtc

Clone ID	Clone ID	Total # reads	# wt reads(%)	#1-Indel	#1-Reads(%)	#2-Indel	#2-Reads(%)
CNTNEU DKO data CNTD2 2B6		1281	0 (0.0%)	-89	1272 (99.3%)	-90	8 (0.6%)
CNTNEU DKO data NEU2 2B6	048D	863	0 (0.0%)	-22	844 (97.8%)	-23	15 (1.7%)

Clone 059D (-13/-1 CNTD2; +1/+1 NEU2)

CNTD2: NGS 1, NGS 2 (cell bank)

NEU2: NGS 1, direct sequencing, NGS 2 (cell bank)

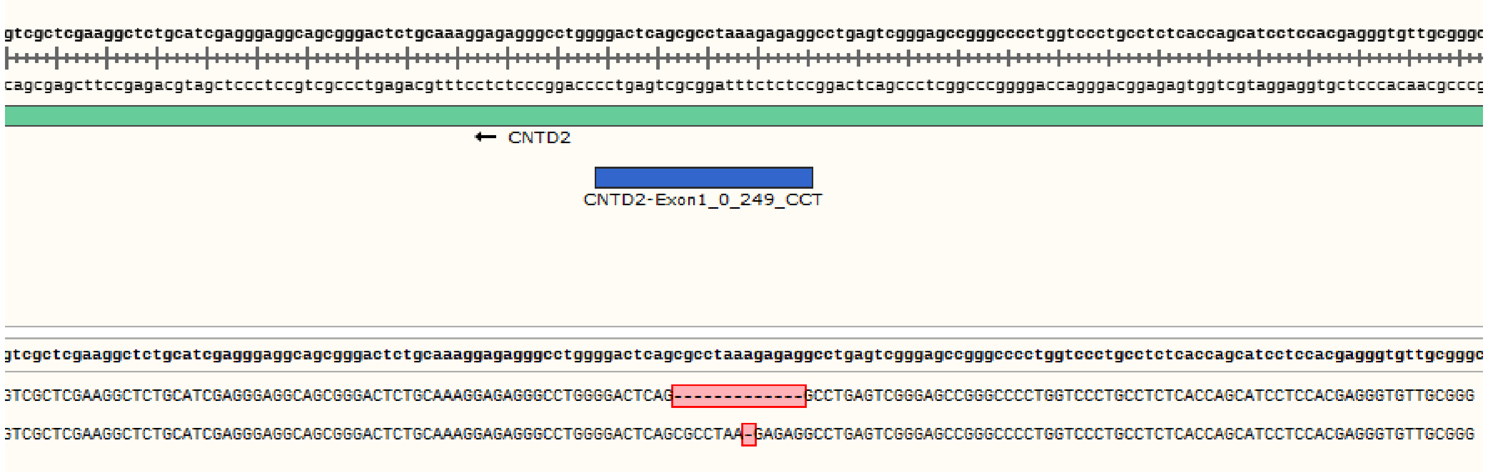

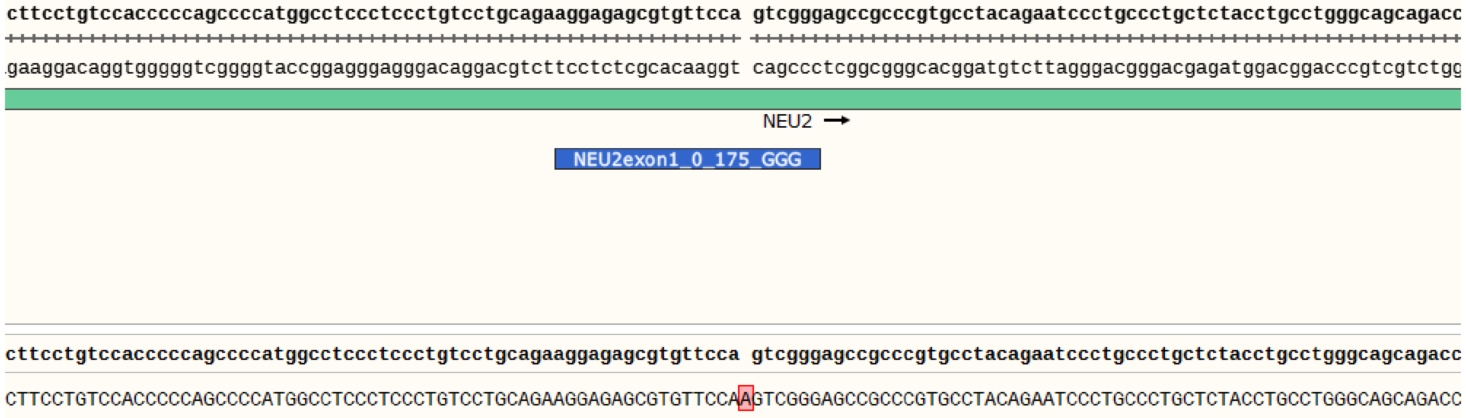

Name	Clone ID	Total # reads	# wt reads(%)	#1-Indel	#1-Reads(%)	#2-Indel	#2-Reads(%)
CNTNEU DKO data CNTD2 1B8		1641	0 (0.0%)	-13	881 (53.7%)	-1	714 (43.5%)
CNTNEU DKO data NEU2 1B8	059D	1363	0 (0.0%)	1	1337 (98.1%)	0	25 (1.8%)

Clone 059D (-13/-1 CNTD2; +1/+1 NEU2)

CNTD2: NGS 1, NGS 2 (cell bank)

NEU2: NGS 1, direct sequencing, NGS 2 (cell bank)

NEU2 direct sequence

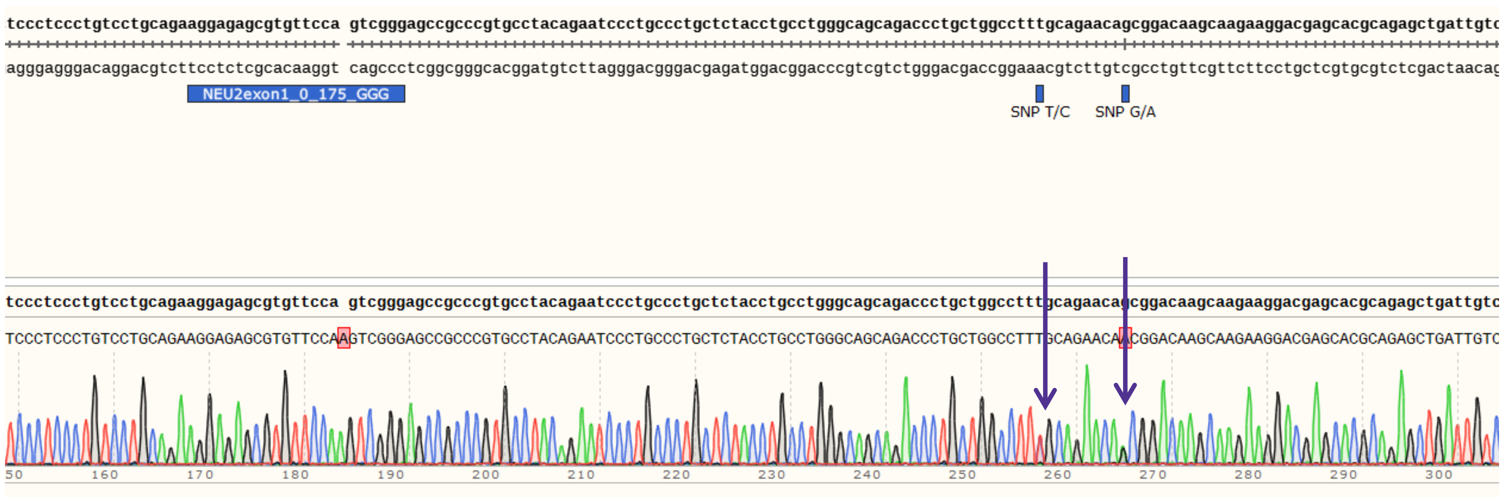

Magnified

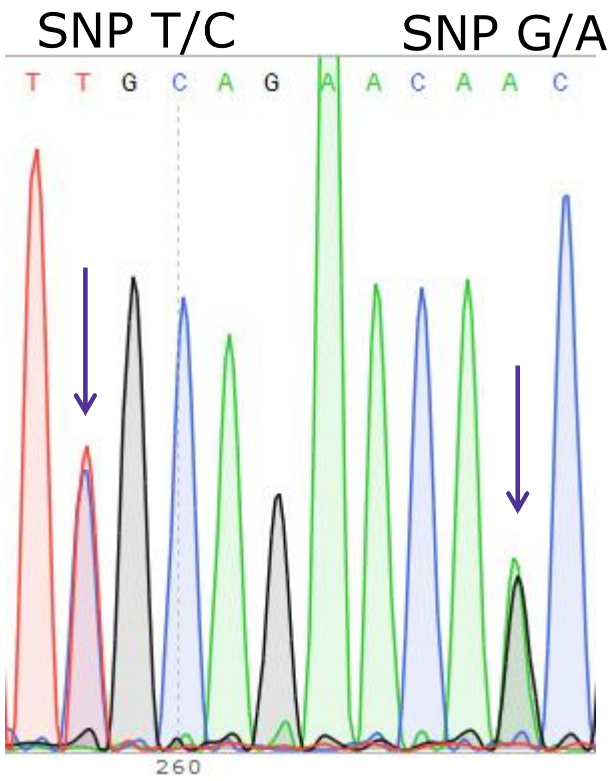

Clone ID	Clone ID	Total # reads	# wt reads(%)	#1-Indel	#1-Reads(%)	#2-Indel	#2-Reads(%)
CNTNEU DKO data CNTD2 1B8		1362	0 (0.0%)	-13	733 (53.8%)	-1	604 (44.3%)
CNTNEU DKO data NEU2 1B8	059D	4170	0 (0.0%)	1	3916 (93.9%)	-100	138 (3.3%)

Clone 060D (-38/-1 CNTD2; +16/-100 NEU2)

CNTD2: NGS 1, NGS 2 (cell bank)

NEU2: NGS 1, topo sequencing, NGS 2 (cell bank)

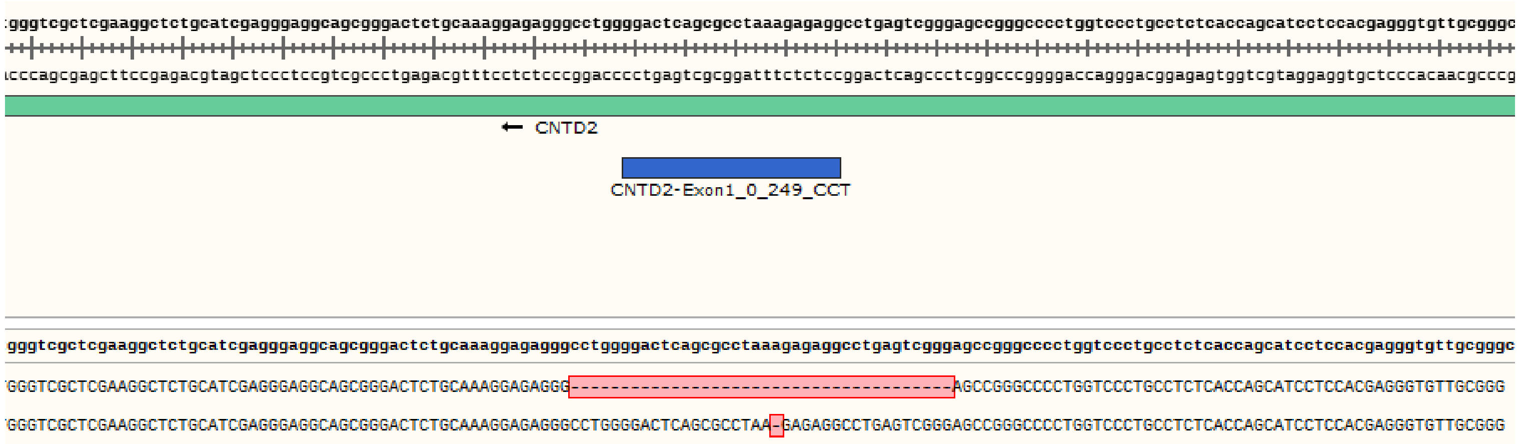

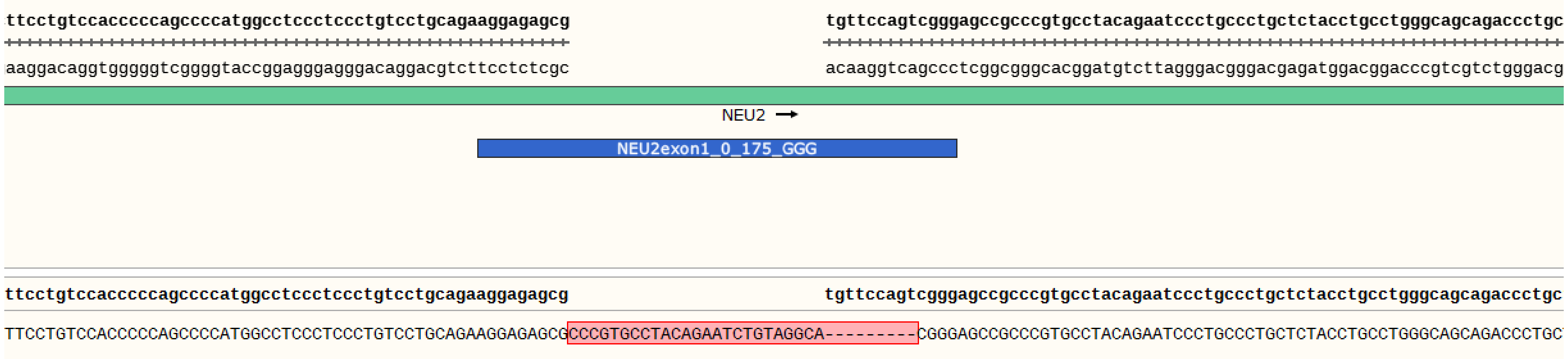

Name	Clone ID	Total # reads	# wt reads(%)	#1-Indel	#1-Reads(%)	#2-Indel	#2-Reads(%)
CNTNEU DKO data CNTD2 1D10		1579	0 (0.0%)	-38	899 (56.9%)	-1	660 (41.8%)
CNTNEU DKO data NEU2 1D10	060D	587	1 (0.2%)	16	569 (96.9%)	15	8 (1.4%)

Clone 060D (-38/-1 CNTD2; +16/-100 NEU2)

CNTD2: NGS 1, NGS 2 (cell bank)

NEU2: NGS 1, topo sequencing, NGS 2 (cell bank)

NEU2 sequence -100

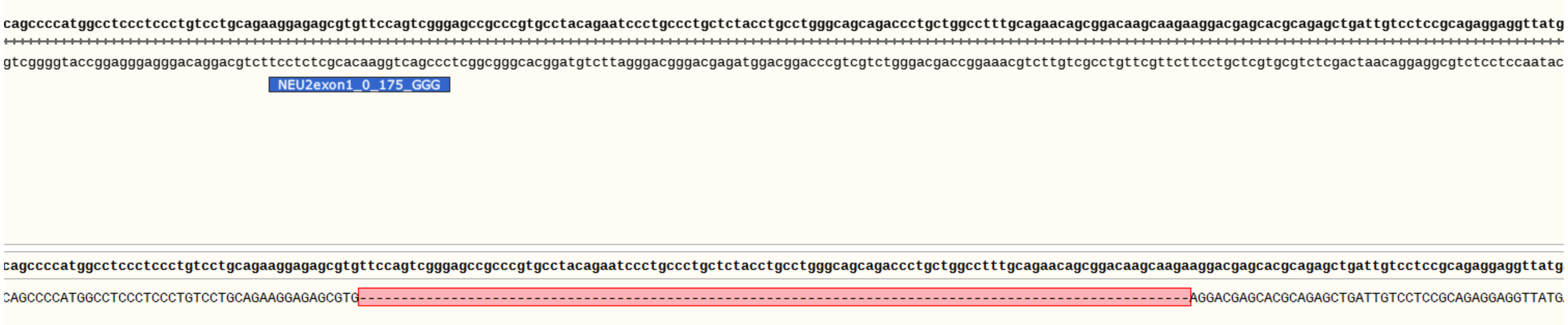

Clone ID	Clone ID	Total # reads	# wt reads(%)	#1-Indel	#1-Reads(%)	#2-Indel	#2-Reads(%)
CNTNEU DKO data CNTD2 1D10		1614	0 (0.0%)	-38	892 (55.3%)	-1	702 (43.5%)
CNTNEU DKO data NEU2 1D10	060D	1816	0 (0.0%)	-100	1190 (65.5%)	16	588 (32.4%)

Clone 061D (-67/-40 CNTD2; +1/-1 NEU2)

CNTD2: NGS 1, NGS 2 (cell bank)

NEU2: NGS 1, NGS 2 (cell bank)

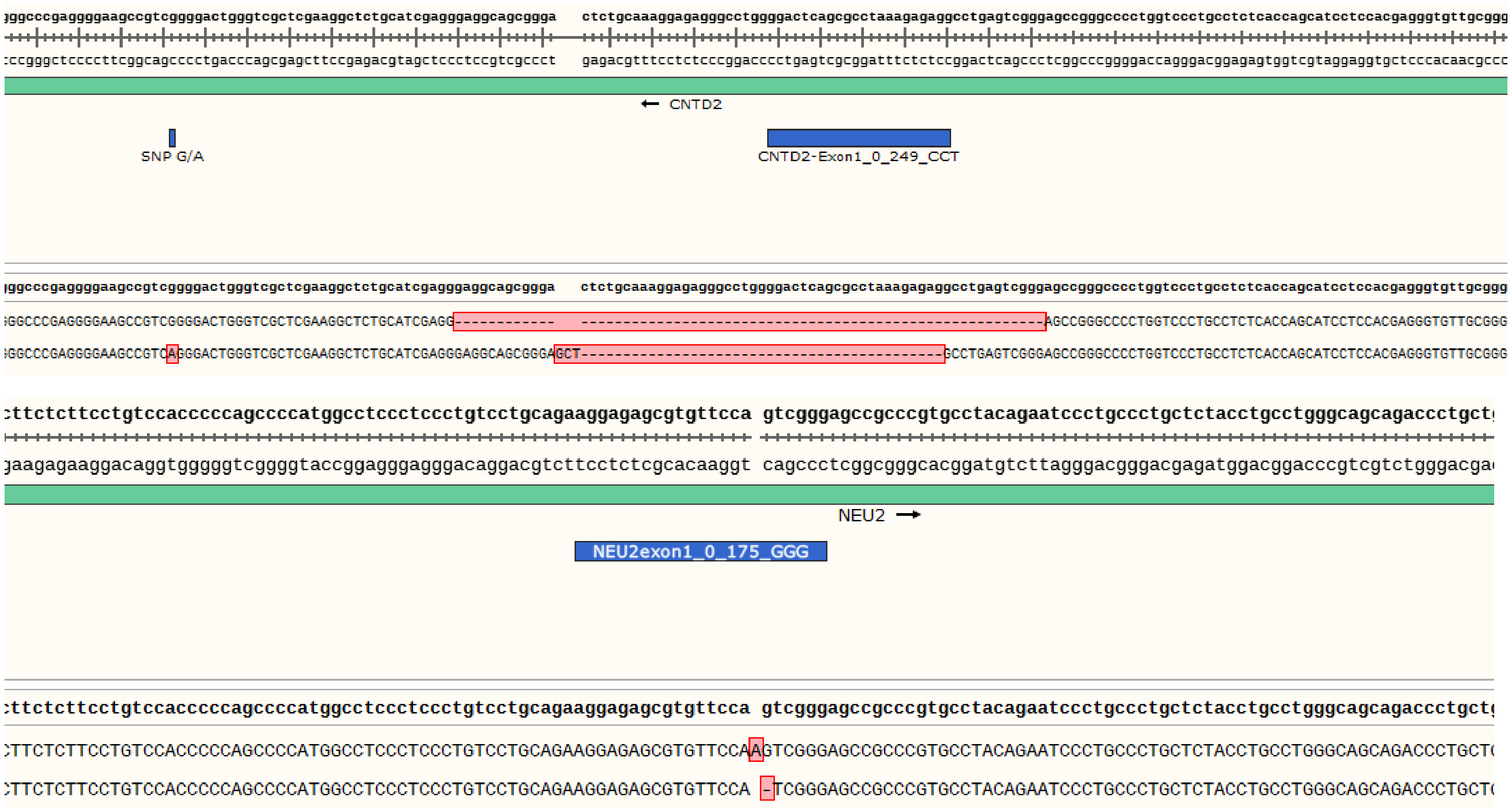

Name	Clone ID	Total # reads	# wt reads(%)	#1-Indel	#1-Reads(%)	#2-Indel	#2-Reads(%)
CNTNEU DKO data CNTD2 1F10		1889	0 (0.0%)	-67	997 (52.8%)	-40	863 (45.7%)
CNTNEU DKO data NEU2 1F10	061D	1229	0 (0.0%)	1	605 (49.2%)	-1	600 (48.8%)

Clone ID	Clone ID	Total # reads	# wt reads(%)	#1-Indel	#1-Reads(%)	#2-Indel	#2-Reads(%)
CNTNEU DKO data CNTD2 1F10		1866	0 (0.0%)	-40	993 (53.2%)	-67	852 (45.7%)
CNTNEU DKO data NEU2 1F10	061D	5500	1 (0.0%)	-1	2709 (49.3%)	1	2520 (45.8%)

Clone 062D (-38/+2 CNTD2; +1/-112 NEU2)

CNTD2: NGS 1, NGS 2 (cell bank)

NEU2: NGS 1, topo sequencing, NGS 2 (cell bank)

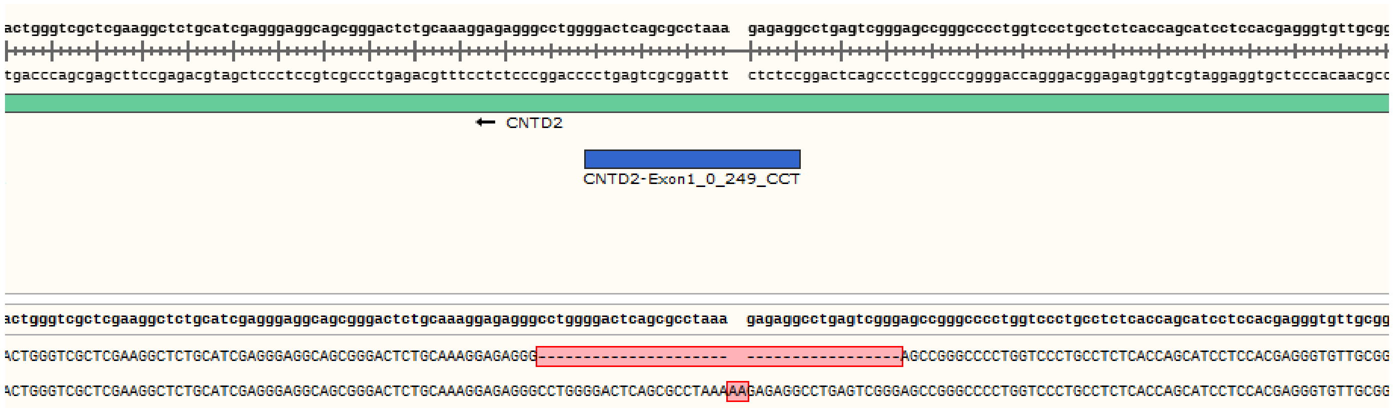

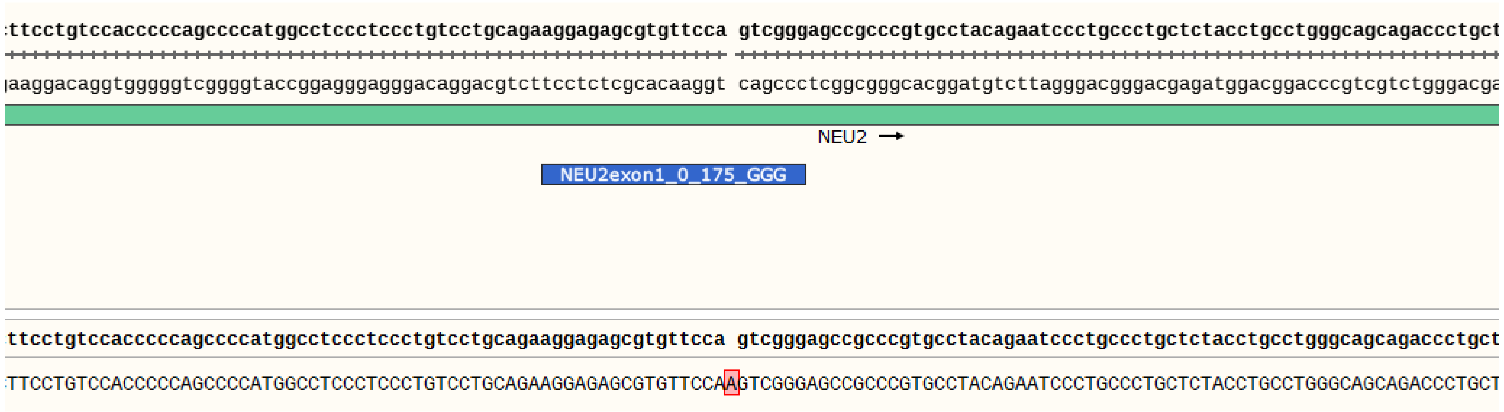

Name	Clone ID	Total # reads	# wt reads(%)	#1-Indel	#1-Reads(%)	#2-Indel	#2-Reads(%)
CNTNEU DKO data CNTD2 1G5		1164	0 (0.0%)	-38	678 (58.2%)	2	471 (40.5%)
CNTNEU DKO data NEU2 1G5	062D	477	1 (0.2%)	1	465 (97.5%)	0	7 (1.5%)

Clone 062D (-38/+2 CNTD2; +1/-112 NEU2)

CNTD2: NGS 1, NGS 2 (cell bank)

NEU2: NGS 1, topo sequencing, NGS 2 (cell bank)

NEU2 sequence -112

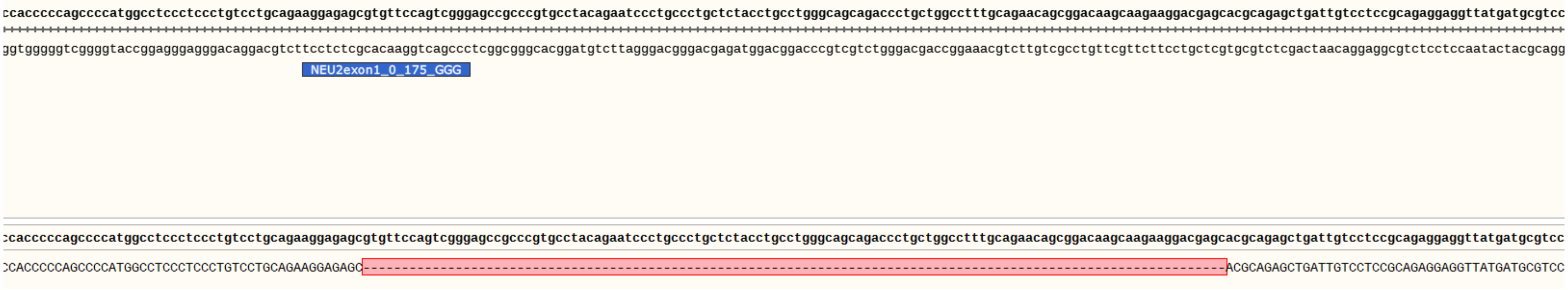

Clone ID	Clone ID	Total # reads	# wt reads(%)	#1-Indel	#1-Reads(%)	#2-Indel	#2-Reads(%)
CNTNEU DKO data CNTD2 1G5		1631	0 (0.0%)	-38	897 (55.0%)	2	707 (43.3%)
CNTNEU DKO data NEU2 1G5	062D	1313	1 (0.1%)	-112	957 (72.9%)	1	290 (22.1%)

Clone 064D (+1/+2 CNTD2; -8/+5 NEU2)

CNTD2: NGS 1, NGS 2 (cell bank)

NEU2: NGS 1, NGS 2 (cell bank)

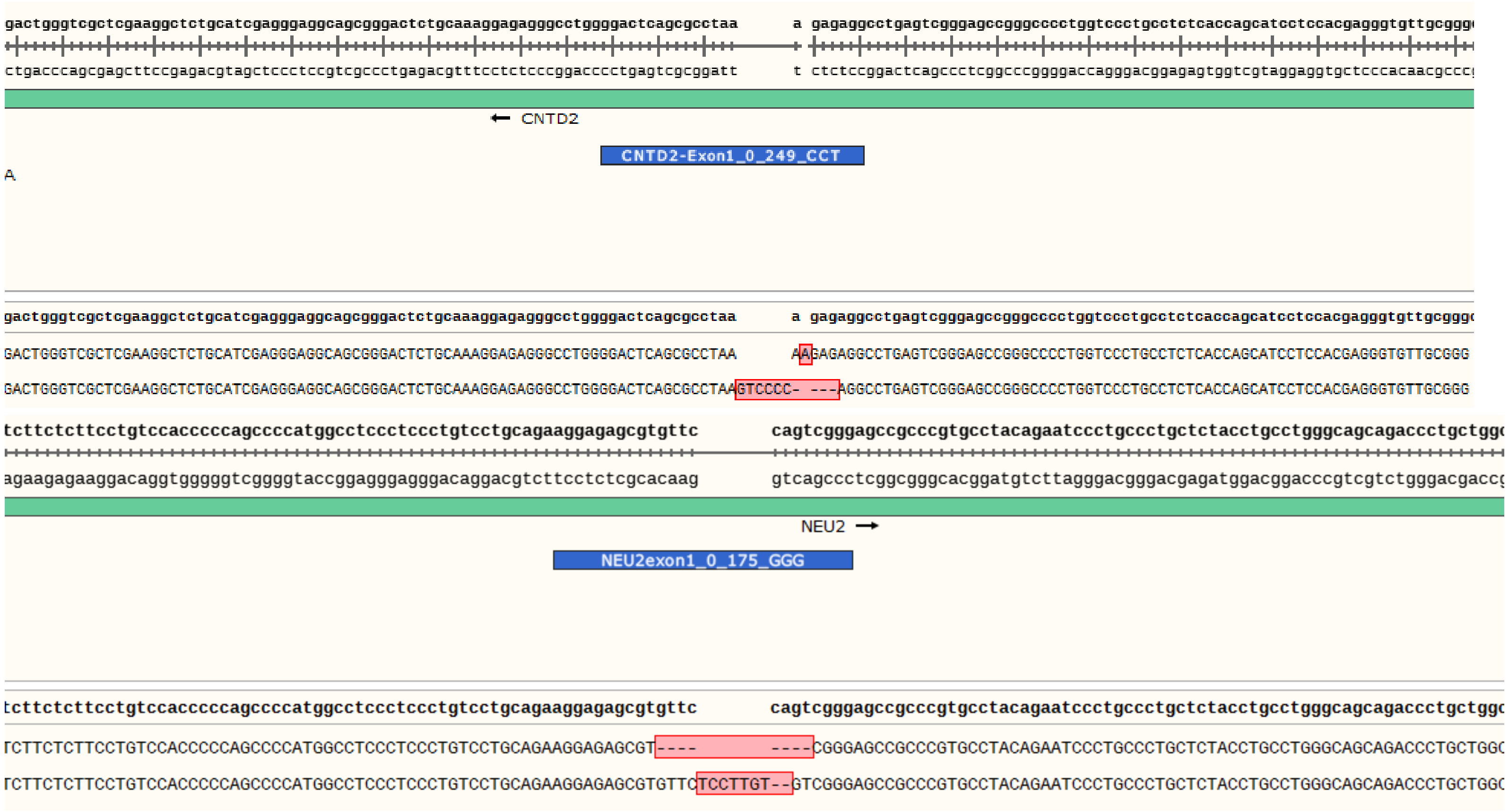

Name	Clone ID	Total # reads	# wt reads(%)	#1-Indel	#1-Reads(%)	#2-Indel	#2-Reads(%)
CNTNEU DKO data CNTD2 2H5		808	3 (0.4%)	1	510 (63.1%)	2	277 (34.3%)
CNTNEU DKO data NEU2 2H5	064D	727	3 (0.4%)	-8	379 (52.1%)	5	325 (44.7%)

Clone ID	Clone ID	Total # reads	# wt reads(%)	#1-Indel	#1-Reads(%)	#2-Indel	#2-Reads(%)
CNTNEU DKO data CNTD2 2H5		13532	20 (0.1%)	1	7603 (56.2%)	2	5699 (42.1%)
CNTNEU DKO data NEU2 2H5	064D	5559	4 (0.1%)	-8	2967 (53.4%)	5	2482 (44.6%)

Clone 065D (-47/-11 CNTD2; -17/-1 NEU2)

CNTD2: NGS 1, NGS 2 (cell bank)

NEU2: NGS 1, NGS 2 (cell bank)

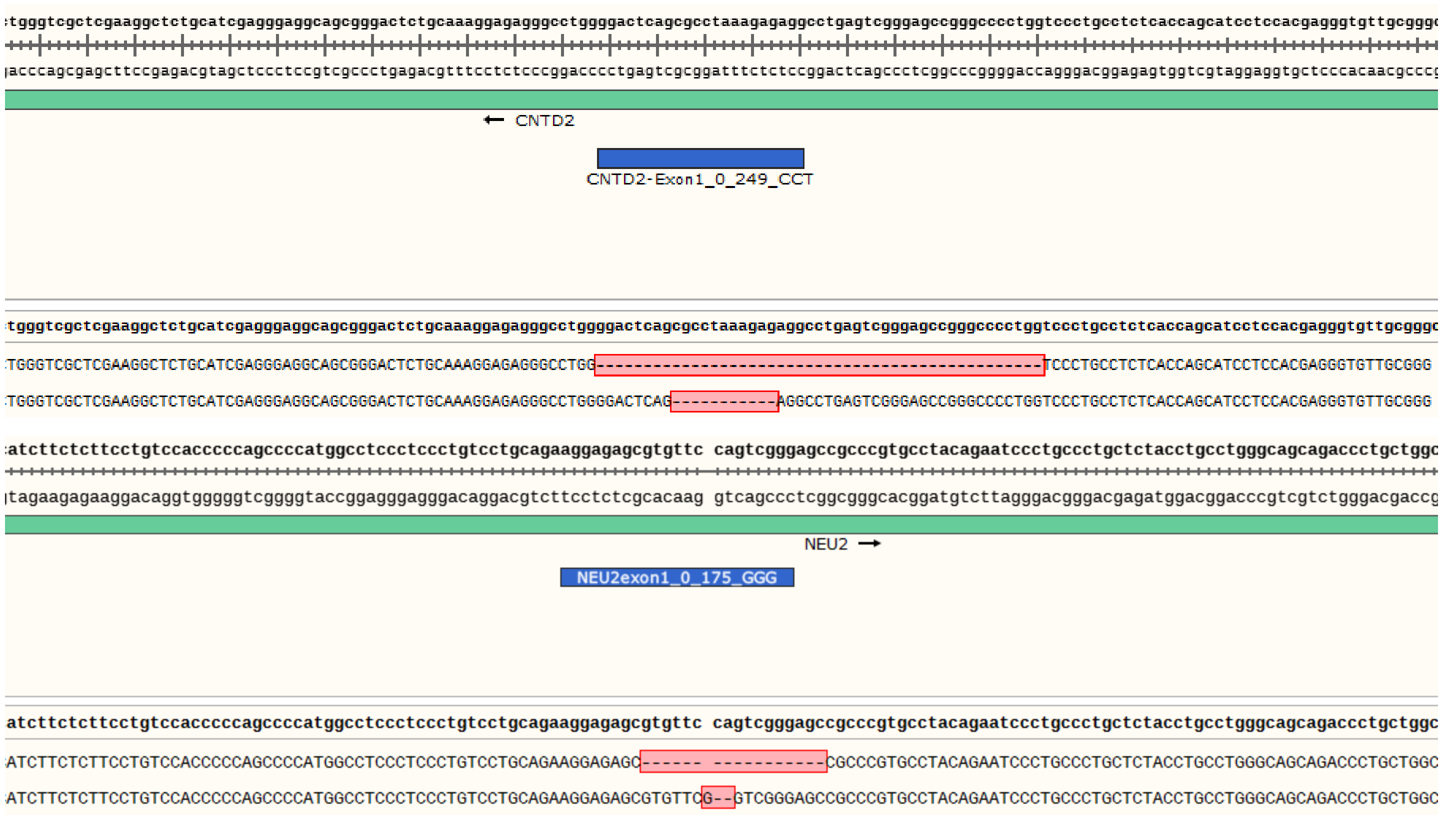

Name	Clone ID	Total # reads	# wt reads(%)	#1-Indel	#1-Reads(%)	#2-Indel	#2-Reads(%)
CNTNEU DKO data CNTD2 3C3		18	0 (0.0%)	-47	12 (66.7%)	-11	6 (33.3%)
CNTNEU DKO data NEU2 3C3	065D	703	2 (0.3%)	-17	392 (55.8%)	-1	300 (42.7%)

Clone ID	Clone ID	Total # reads	# wt reads(%)	#1-Indel	#1-Reads(%)	#2-Indel	#2-Reads(%)
CNTNEU DKO data CNTD2 3C3		1519	0 (0.0%)	-47	878 (57.8%)	-11	626 (41.2%)
CNTNEU DKO data NEU2 3C3	065D	6346	0 (0.0%)	-17	3116 (49.1%)	-1	2892 (45.6%)

Clone 066D (+1/+37 CNTD2; +1/-172 NEU2)

CNTD2: NGS 1, NGS 2 (cell bank)

NEU2: NGS 1, topo sequencing, NGS 2 (cell bank)

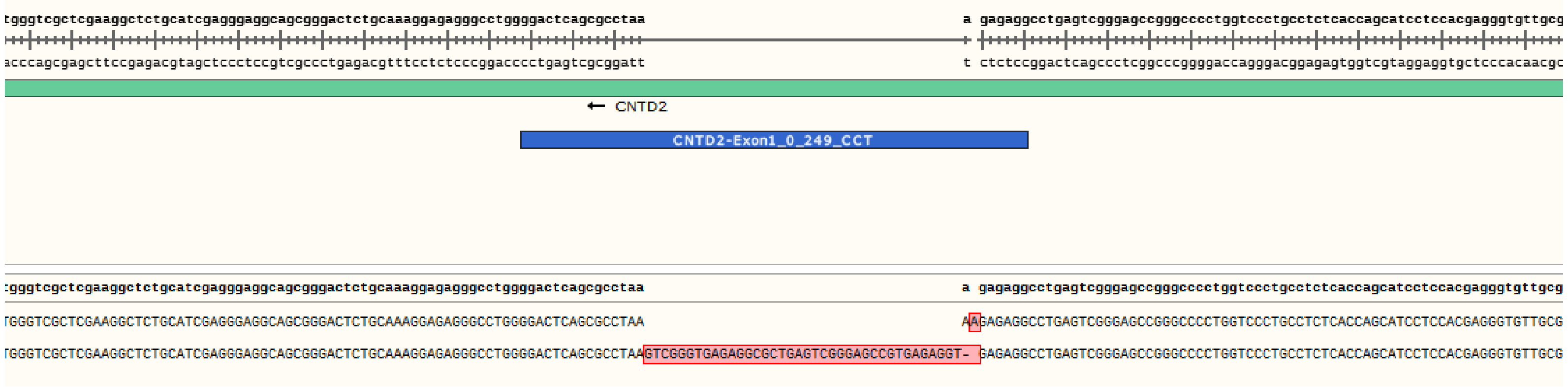

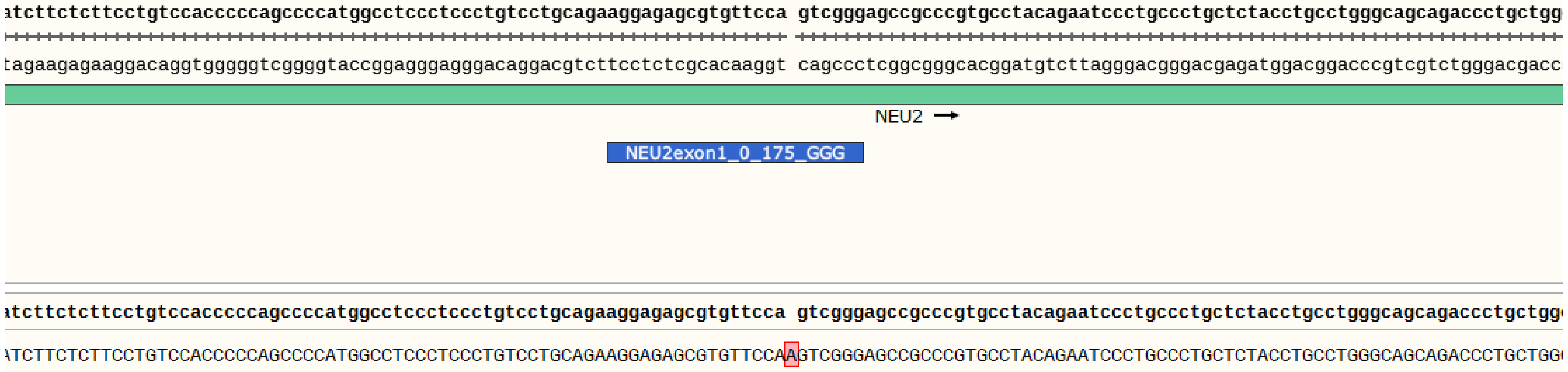

Name	Clone ID	Total # reads	# wt reads(%)	#1-Indel	#1-Reads(%)	#2-Indel	#2-Reads(%)
CNTNEU DKO data CNTD2 3G6		1007	3 (0.3%)	1	607 (60.3%)	37	371 (36.8%)
CNTNEU DKO data NEU2 3G6	066D	825	0 (0.0%)	1	807 (97.8%)	0	16 (1.9%)

Clone 066D (+1/+37 CNTD2; +1/-172 NEU2)

CNTD2: NGS 1, NGS 2 (cell bank)

NEU2: NGS 1, topo sequencing, NGS 2 (cell bank)

NEU2 sequence -172

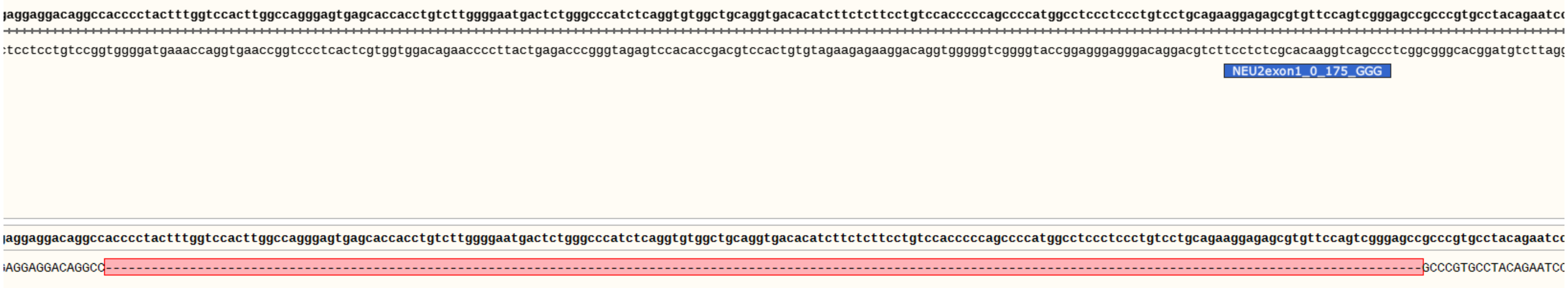

NEU2 deleted sequence

acccctactttggtccacttggccagggagtgagcaccacctgtcttggggaatgactctgggcccatctcaggtgtggctgcaggtgacacatcttctcttcctgtccacccccagccccatggcctccctccctgtcctgcag aaggagagcgtgttccagtcgggagcc

Clone ID	Clone ID	Total # reads	# wt reads(%)	#1-Indel	#1-Reads(%)	#2-Indel	#2-Reads(%)
CNTNEU DKO data CNTD2 3G6		1408	2 (0.1%)	1	764 (54.3%)	37	614 (43.6%)
CNTNEU DKO data NEU2 3G6	066D	4702	2 (0.0%)	1	4272 (90.9%)	-100	243 (5.2%)
